# Predicting the molecular mechanism-driven progression of breast cancer through comprehensive network pharmacology and molecular docking approach

**DOI:** 10.1038/s41598-023-40684-7

**Published:** 2023-08-22

**Authors:** Bharti Vyas, Sunil Kumar, Ratul Bhowmik, Mymoona Akhter

**Affiliations:** 1 School of Interdisciplinary Science and Technology, Jamia Hamdard, New Delhi, India; 2 ICAR-Indian Institute of Farming System Research, Modipuram, Meerut, 250110 India; 3 Department of Pharmaceutical Chemistry, School of Pharmaceutical Education and Research, Jamia Hamdard, New Delhi, 110062 India

**Keywords:** Computational biology and bioinformatics, Drug discovery, Diseases

## Abstract

Identification of key regulators is a critical step toward discovering biomarker that participate in BC. A gene expression dataset of breast cancer patients was used to construct a network identifying key regulators in breast cancer. Overexpressed genes were identified with BioXpress, and then curated genes were used to construct the BC interactome network. As a result of selecting the genes with the highest degree from the BC network and tracing them, three of them were identified as novel key regulators, since they were involved at all network levels, thus serving as the backbone. There is some evidence in the literature that these genes are associated with BC. In order to treat BC, drugs that can simultaneously interact with multiple targets are promising. When compared with single-target drugs, multi-target drugs have higher efficacy, improved safety profile, and are easier to administer. The haplotype and LD studies of the *FN1* gene revealed that the identified variations rs6707530 and rs1250248 may both cause TB, and endometriosis respectively. Interethnic differences in SNP and haplotype frequencies might explain the unpredictability in association studies and may contribute to predicting the pharmacokinetics and pharmacodynamics of drugs using *FN1*.

## Introduction

Breast cancer is a malignant tumor of the glandular milk duct epithelial cells or of the breast lobule. It is a multifaceted disease and the most common type of cancer among women. In 2020, there were 2,261,419 newly diagnosed cases and 684,996 deaths due to BC^1,2^. The overall survival rate for BC is very low, but with proper diagnosis and treatment, there are good chances of recovery^3^. Risk factors for BC progression generally fall into two categories causal and non-causal. As a causal risk factor, breast cancer is influenced by a mutation in genes and non-causal risk factors (age, drinking alcohol, hormonal imbalance, obesity, and abnormal menstruation, some genetic or epigenetic factors) have an indirect effect on BC progression. Causal factors are directly responsible for BC progression and accurate identification of these risk factors is therefore crucial for early diagnosis, prognosis, and treatment of breast cancer. The molecular mechanisms and pathogenic processes involved in BC progression are not clearly understood yet therefore understanding the molecular mechanisms and pathogenetic processes of BC requires the identification of both causal and non-causal genetic risk factors. Since genetic factors play a crucial role in the disease the identification of disease-causing key genes will help understand the disease better. Transcriptomics analysis has gained much popularity as a platform for study of key genes responsible for various diseases including cancer as indicated by various reports in the literature^4–13^. Various therapeutic strategies adopted for the intervention of disease, such as targeted therapy, have led to a decline in global breast cancer death rates. Therefore, identification of key genes involved in the development of diseases like BC will help in the understanding of prognosis and development of appropriate treatment for the disease.

Although the development of molecular targets for breast cancer prognosis and treatment have improved^14,15^ but at the same time resistance to several anticancer therapies has also been reported thereby a continuous demand for search for newer interventions for the disease. A number of individual studies have been reported with several sets of key genes responsible for BC based on single transcriptomic dataset analysis^4–13^, however no common key gene could be identified from these studies. To obtain stable and precise results researchers usually use multiple datasets for their study that may or may not be normalized. We have used normalized dataset (BioXpress) for our study to minimize the errors using bioinformatic techniques to screen potential biomarkers for breast cancer. BioXpress v3.0 (https://hive.biochemistry.gwu.edu/bioxpress)^16^ database enables researchers to select appropriate profiles of mRNA expression data of BC. DAVID was used to perform functional and pathway enrichment analyses on the identified DEGs, STRING^17^ to construct PPI networks and Cytoscape to visualize them^18,19^. The genotype data of Han Chinese (CHB) was used to examine the identified biomarkers and their association with other diseases.

Drug repurposing (DR) process identifies new therapeutic uses from existing drugs (Provised drugs, failed/investigational drugs, marketed drugs, etc.). Comparatively to traditional and de-novo drug discovery, DR is safer, more time-efficient, less time-consuming, and less expensive therefore, using approved drugs as a starting point to discover new therapeutics for other diseases is a promising strategy. In the past decade, the DR strategy has grown in popularity between researchers and pharmaceutical companies with considerable success. In order to determine the structural binding affinity between drugs and targets, molecular docking analysis was used in several studies. The main objectives of this study are as follows: (i) Identification of common BC-causing biomarkers and their associated disease. (ii) Investigating drugs guided by biomarkers for treating BC, and (iii) validation of identified drugs molecules by docking.

## Result

### Construction of network and identification of biomarker by multiple overlapping closed curves

**Figure 1:**
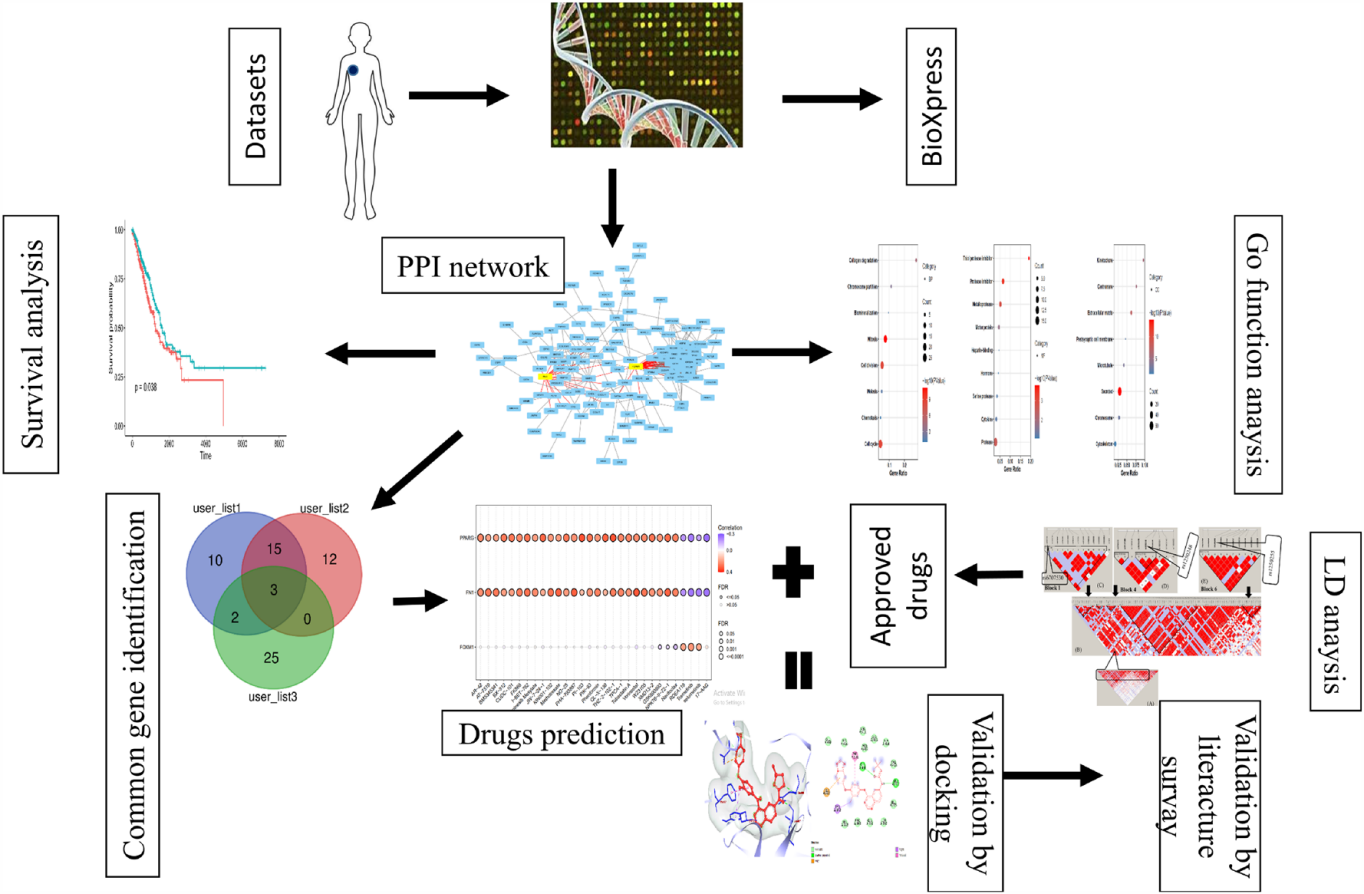
Schematic diagram summarizing the study.

**Figure 2:**
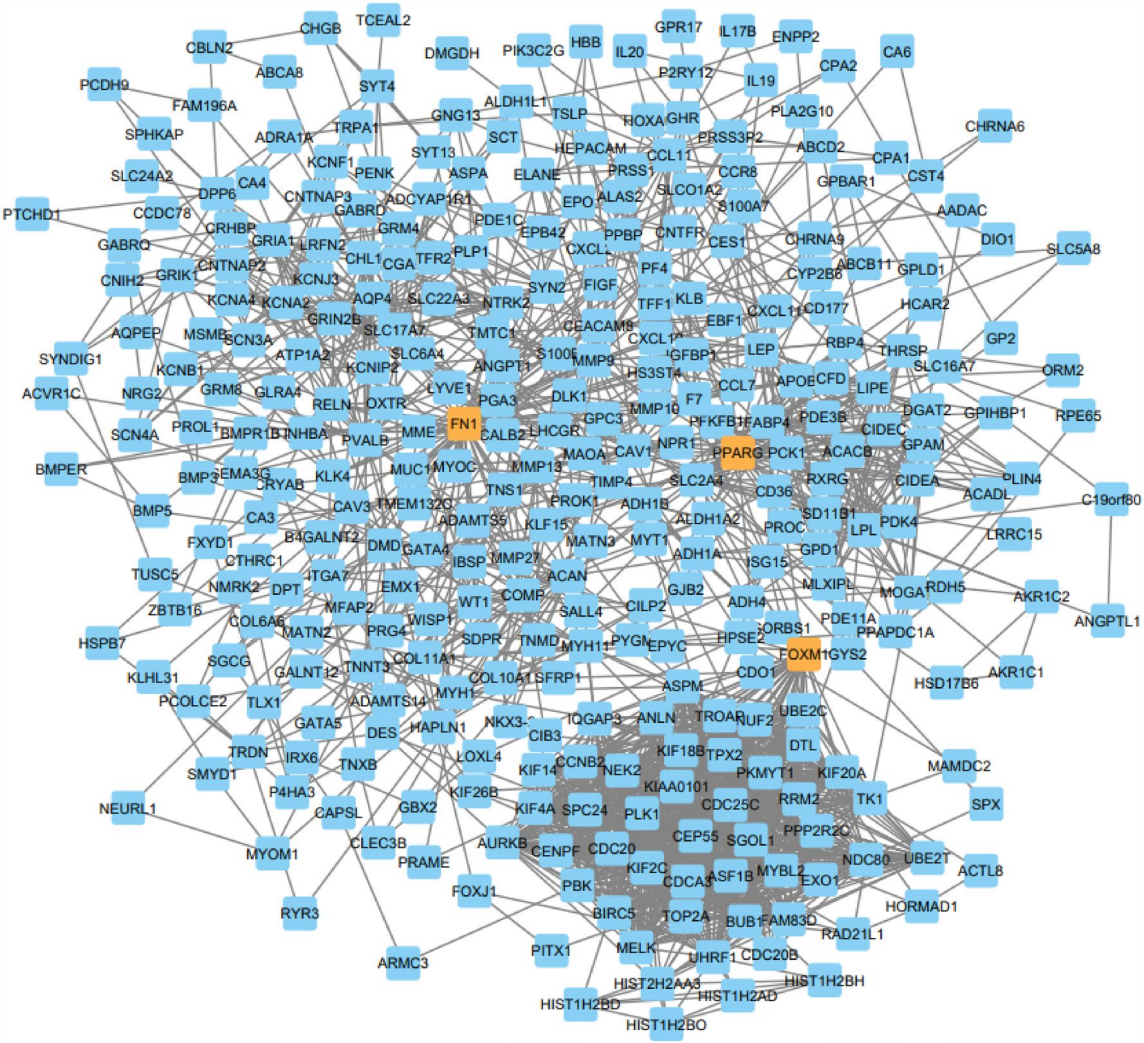
Network of the up regulated gene in breast cancer.

**Figure 3:**
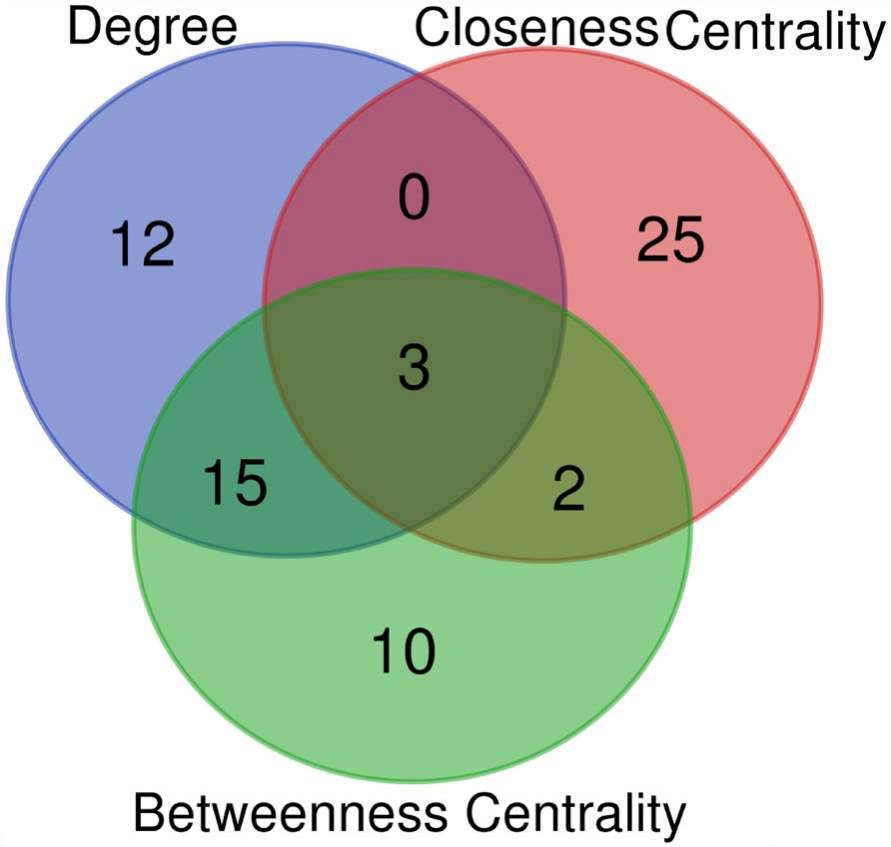
Venn Diagram according to the degree, betweenness, and closeness centrality list of gene.

The STRING server was employed to prepare the network of the selected BC target. The selected list (5557 genes, table S1) of the target was submitted in the STRING to generate the network. The output file showed the interaction between the targets obtained from BioExpress database. A schematic diagram provides a summary of all methodological techniques in a schematic diagram (Fig. 1). STRING has a default feature that does not carry duplicate genes; those genes are not officially mention. STRING was generated the network 532 out of 5556 genes (Fig. 2). STRING was generated the network 532 out of 5556 genes that was take up as input in Cytoscape to generate the node file. The results obtained were summited to Cytoscape and the node file was generated using the network analyzer to obtain the details of degree, betweenness, and closeness centrality (Table S2). On the basis of these results, 30 targets were selected and a Venn diagram was generated using the Venn Diagram tool. The Venn diagram result revealed that 3 genes have a close relationship with each other and the target disease (Fig. 3, Table 1).

**Table 1:** result obtained in terms of list of genes name with closenesscentrality value, degree value and betweennesscentrality value by using cytoscape.

S. no.	List of closeness centrality value with gene name		List of degree value with gene name		List of betweenness centrality value with gene name	
	Closeness centrality value	Gene name	Degree value	Gene name	Betweenness centrality value	Gene name
1.	0.390058972	FN1	55	FN1	0.20955087	FN1
2.	0.380756579	MMP9	47	ASPM	0.111268893	MMP9
3.	0.373990307	GFAP	47	PLK1	0.099078669	GFAP
4.	0.358914729	PPARG	45	HJURP	0.073683881	PPARG
5.	0.35506135	LEP	44	FOXM1	0.069789183	FOXM1
6.	0.35506135	CAV1	44	PBK	0.063945787	PVALB
7.	0.351023503	PVALB	44	AURKB	0.054494125	CAV1
8.	0.342962963	ACAN	44	KIF2C	0.050132032	AQP4
9.	0.342202513	AQP4	44	UBE2C	0.049865671	ASPM
10.	0.340942563	S100B	44	BUB1	0.043633496	DPP6
11.	0.340691685	ADIPOQ	43	KIF20A	0.04099145	LEP
12.	0.334537572	CXCL10	43	KIF4A	0.040050006	GATA4
13.	0.334296029	GATA4	43	TPX2	0.036809687	PBK
14.	0.331899642	NTRK2	43	CDC20	0.03589432	APOB
15.	0.331187411	MMP13	42	PPARG	0.030337315	CES1
16.	0.331187411	RELN	42	TOP2A	0.030289124	ACAN
17.	0.328136074	MYH11	41	EXO1	0.029325479	IBSP
18.	0.327208481	DMD	41	RRM2	0.028388592	CAV3
19.	0.324684432	IGFBP1	41	CCNB2	0.028324107	S100B
20.	0.324229692	APOB	41	BIRC5	0.027015506	CAPSL
21.	0.324229692	CALB2	41	CENPF	0.026273232	MYH11
22.	0.323549965	IBSP	41	UHRF1	0.025703679	DMD
23.	0.323324022	ITGA7	41	NEK2	0.024780402	LPL
24.	0.323098395	FOXM1	41	NDC80	0.024613117	WT1
25.	0.323098395	TIMP4	40	ASF1B	0.024271394	EPO
26.	0.321527778	SLC17A7	40	MELK	0.02321294	SLC2A4
27.	0.32130465	WT1	40	CEP55	0.022544861	PRSS1
28.	0.320637119	COMP	40	NUF2	0.02236115	TK1
29.	0.319972357	GRIN2B	39	MYBL2	0.021729956	NEURL1
30.	0.318870523	EPO	39	CDC25C	0.020696107	SLC6A4

### Identification of BC-related pathways and biological processes

**Figure 4:**
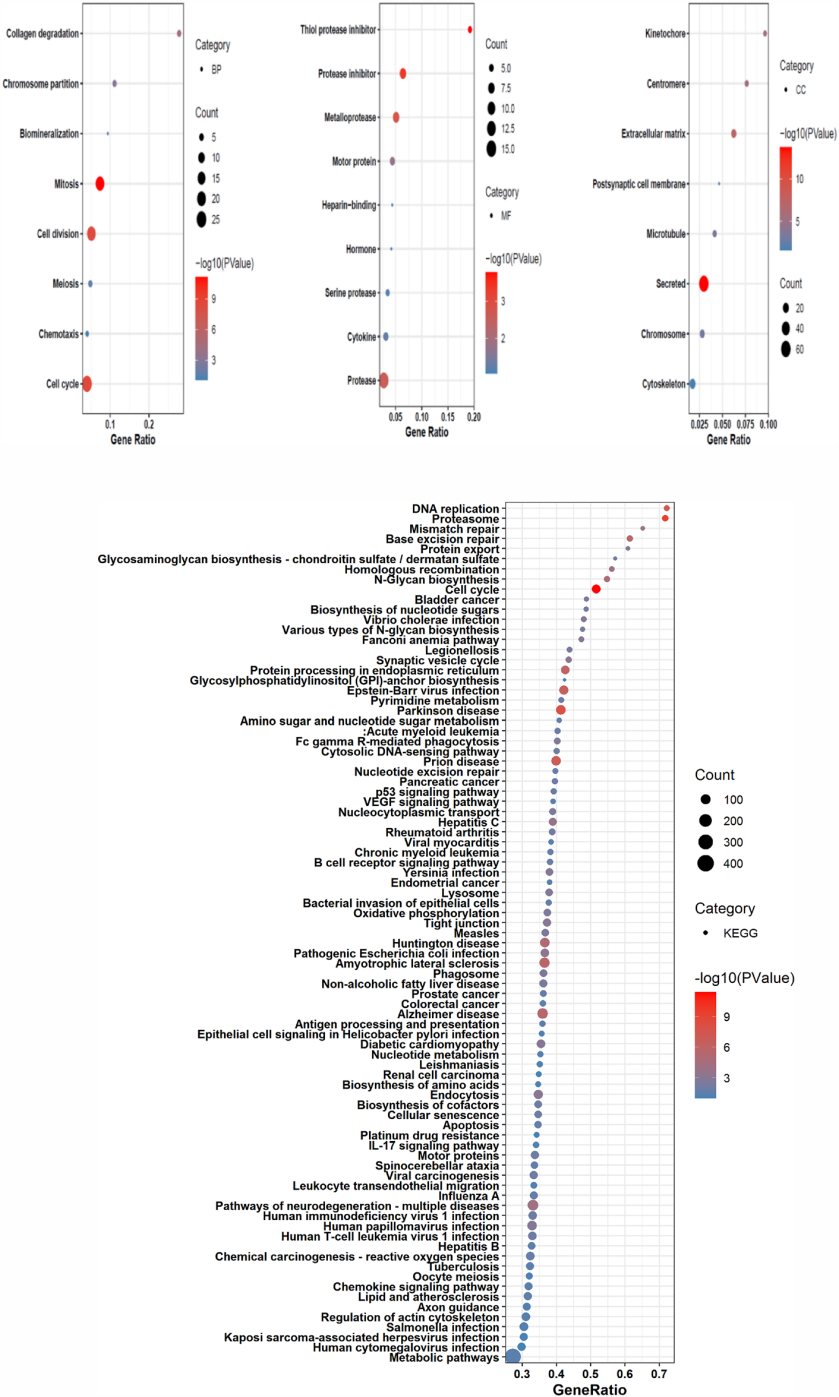
^a^Plots illustrating the correlation between the biological processes (BPs), cellular components (CCs), molecular function (MF) and KEGG pathways with the genes identified by DAVID.

DAVID was used to analyze the Enrichment analysis with Gene Ontology (GO) biological processes for selected genes after generated the network related to BC. GO has several levels of terms like GO biological processes, cellular components, and molecular function, and all three were used for analysis of selected genes. The genes were found related to 15 biological processes, 8 cellular components, and 10 molecular function processes associated with BC (Fig. 4). Three plots were generated using R to illustrate the correlation between the biological processes, cellular components, and molecular function with the genes identified. The results indicated that the genes involved with the developmental proteins and oxidoreductase will make a good target for the design and development of anti-BC agents.

### Identification of hub genes associated BC

**Figure 5:**
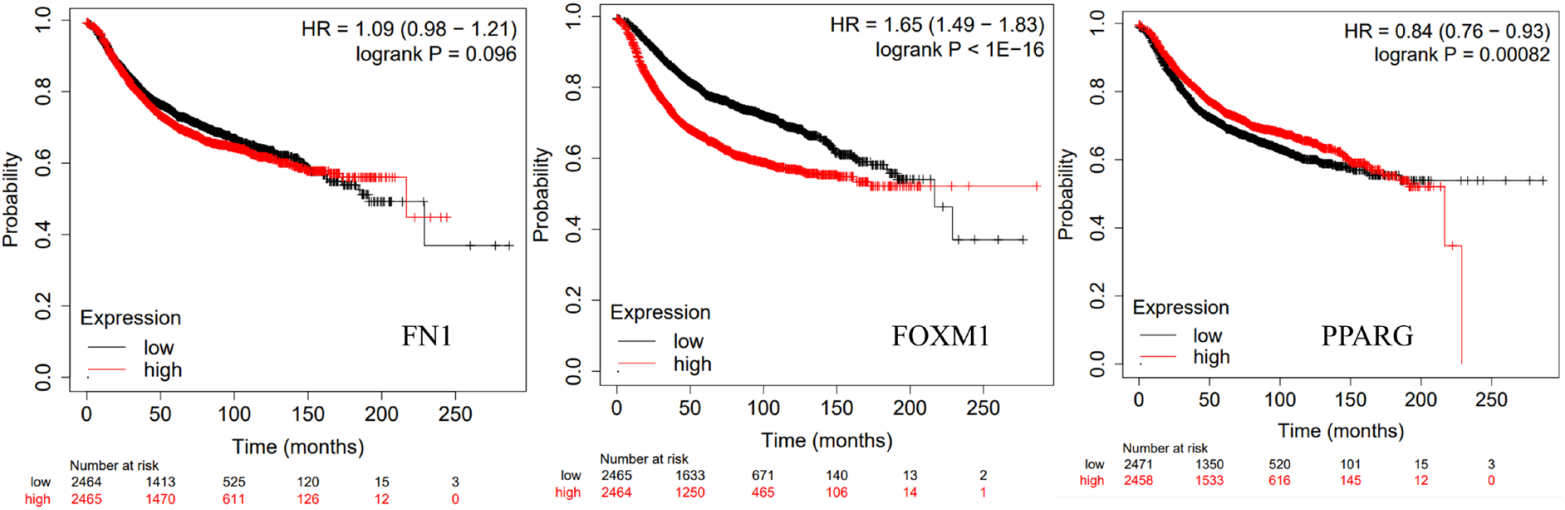
Kaplan–Meier curve of key regulators FN1, FOXM1 and PPARG. *p* values were calculated using the log rank test to evaluate the overall survival analysis between low expression (black) and high expression (red) of key regulator genes of patients.

Hub genes tend to exert core functions in a closely related network. Of the 532 genes within the module, 30 most relevant genes were selected as the candidate hub genes (*FN1, MMP9, GFAP, PPARG, FOXM1, PVALB, CAV1, AQP4, ASPM, DPP6, LEP, GATA4, PBK, APOB, CES1, ACAN, IBSP, CAV3, S100B, CAPSL, MYH11, DMD, LPL, WT1, EPO, SLC2A4, PRSS1, TK1, NEURL1 and SLC6A4*). Three genes (*FN1, FOXM1, and PPARG*) out of 30 genes were significantly associated with BC. The prognosis results of these three genes adjusted for stage and grade also indicated significance (Fig. 5).

### Study of LD and haplotype

**Figure 6:**
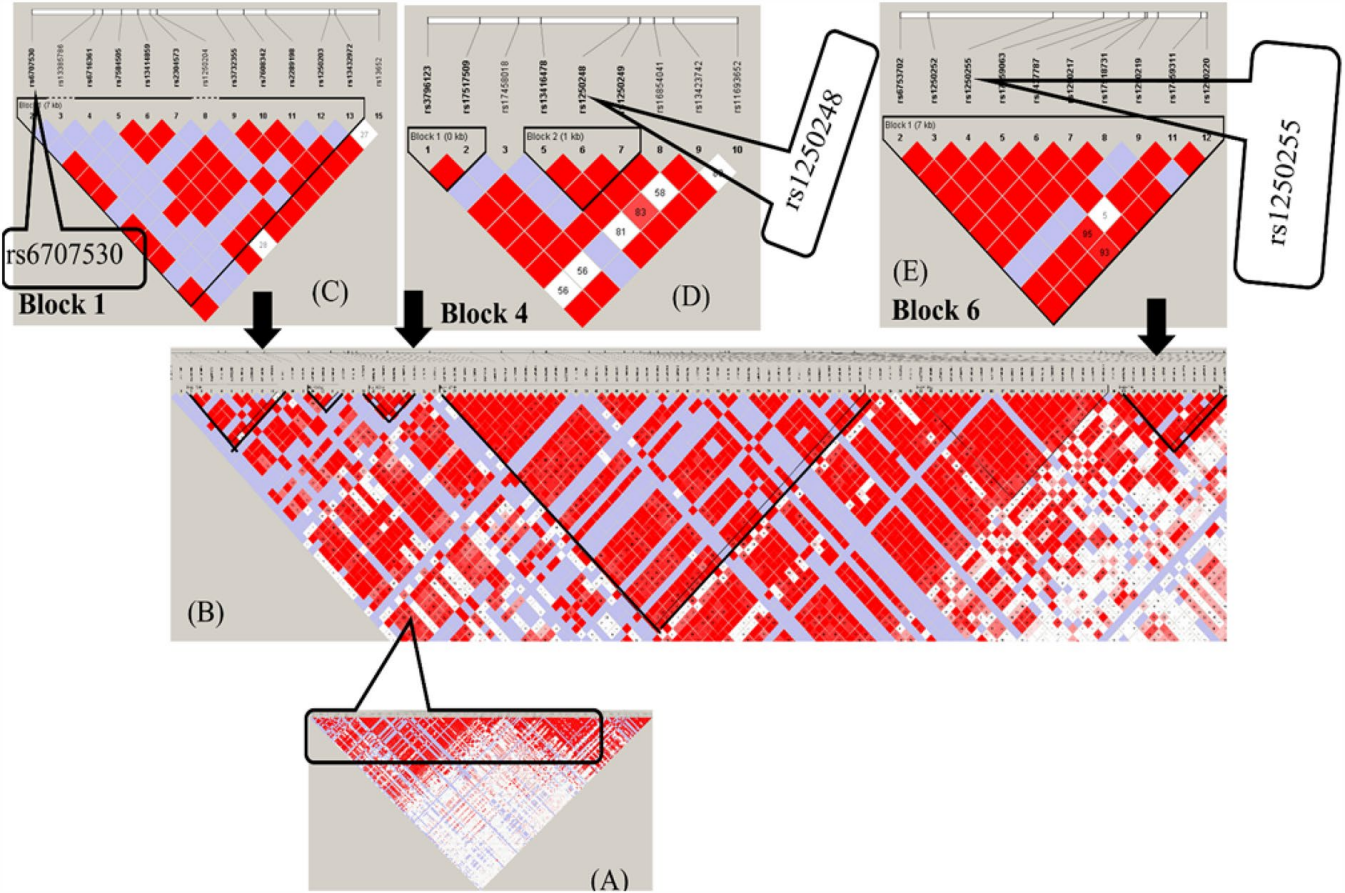
LD structure of FN1 gene: (**A, B**) LD structure of maximum number of SNPs, (**C, D, E**) LD structure of FN1 with minimum block size.

Genotype data of CHB (Han Chinese) were retrieved from the International Hapmap Project for studies of the LD and haplotypes to examine the genetic parameter of the identified *FN1* biomarker. Data about the susceptibility of many diseases due to genetic variants has been evaluated as the information helps to find the vital biomarkers for functional associations with different diseases and drug resistance due to SNPs. The LD and haplotype study revealed significant blocks in the *FN1* gene, having nonrandom associations as represented in Fig. 6, Table S3. Two SNPs (rs1250248, and rs6707530) were identified and found to role in different diseases and drug resistance. These 2 SNPs (rs1250248, and rs6707530) with minor allele frequencies and r2 ≥ 0.8 showed high correlation between the loci. Linkage disequilibrium has been reported between the common polymorphism found on *FN1* at positions 215,360,440 and 215,436,073. The analysis revealed that of the nsSNPs identified, only two nsSNPs occurred and were linked in Han Chinese individuals. The results also indicated that only MAF (minor allele frequency) values of 0.263, and 0.233 showed relatively strong linkage disequilibrium. The genotype of rs6707530 with the *FN1* gene increased the risk of occurrence of TB^20^. The genetic variant of *FN1* rs1250248 was also found to be significantly associated with endometriosis. This study demonstrates a potential connection between breast cancer, TB and endometriosis, and in this paper reported genetic variants of *FN1* were identified as chemoresistance. These results provide an evidence of alteration leading to chemoresistance in several diseases. LD and haplotype information would be beneficial in drug development and in understanding the genetic associations of *FN1* with adverse drug effects.

### Biomarker guided drug repositioning and validation

**Figure 7:**
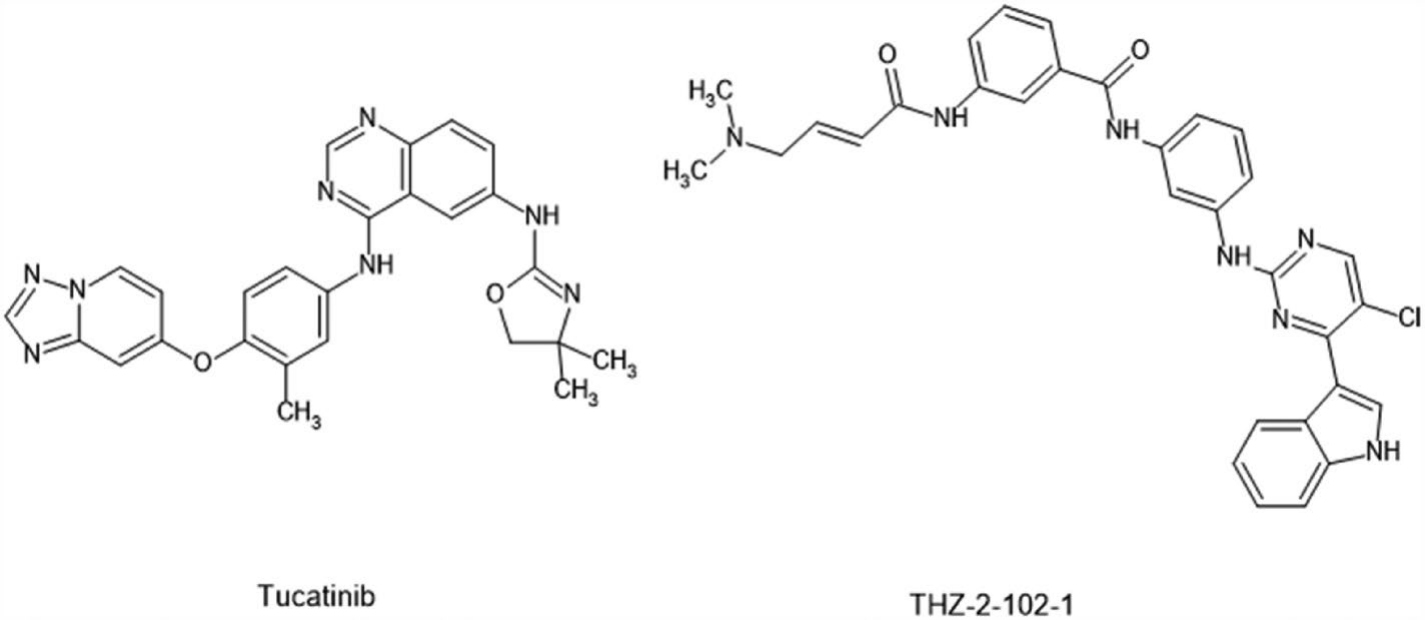
The structure of anti-breast cancer inhibitors of our targets.

**Figure 8:**
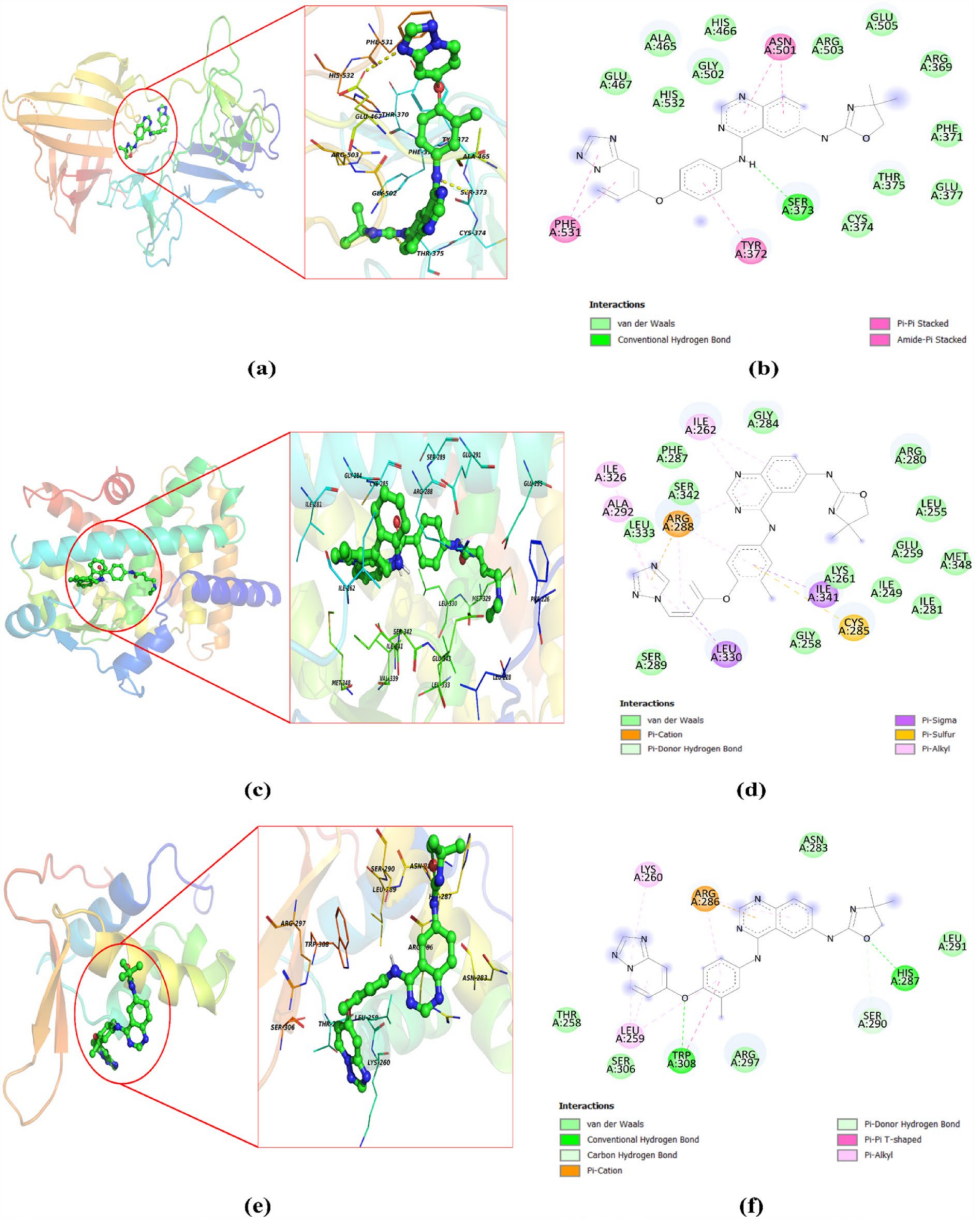

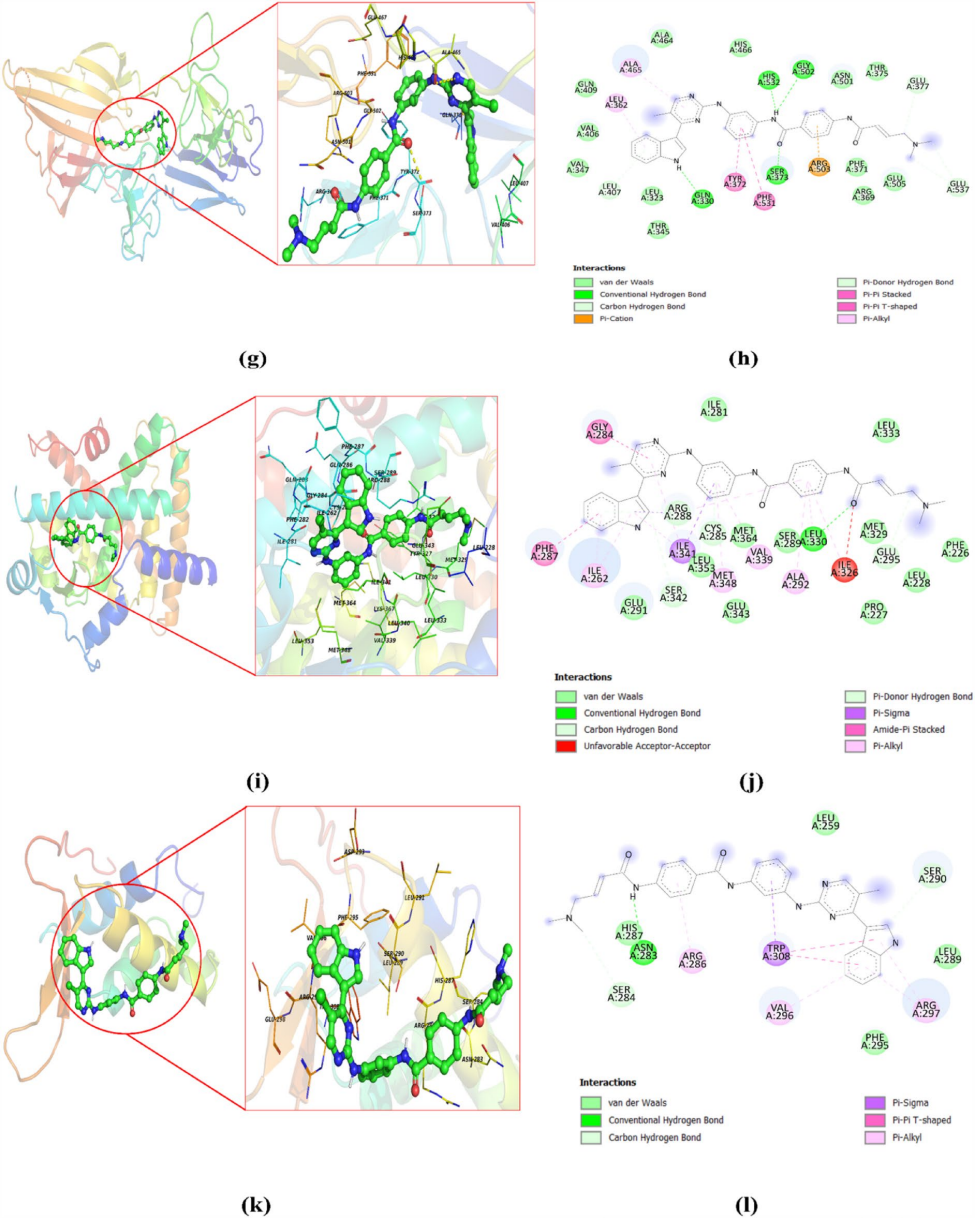
(**a**) Binding of Tucatinib against the FN1 (3M7P) protein. (**b**) 2D interaction of Tucatinib against FN1 (3M7P). (**c**) Binding of Tucatinib against the PPARG (4Y29). (**d**) 2D interaction of Tucatinib against PPARG (4Y29). (**e**) Binding of Tucatinib against FOXM1 (3G73) protein. (**f**) 2D interaction of Tucatinib against FOXM1 (3G73) protein. (**g**) Binding of THZ-2-102-1 against FN1 (3M7P) protein. (**h**) 2D interaction of THZ-2-102-1 against FN1 (3M7P). (i) Binding of THZ-2-102-1 against PPARG (4Y29). (**j**) 2D interaction of THZ-2-102-1 against PPARG (4Y29). (**k**) Binding of THZ-2-102-1 against FOXM1 (3G73) protein. (**l**) 2D interaction of THZ-2-102-1 against FOXM1 (3G73) protein.

To support identification of a drug for targeted therapy, the analysis of drug sensitivity of identified biomarkers by two approaches i.e. LD and GSCA was carried out. Further that the results of LD and haplotype study revealed the relationship between *FN1* (key gene in breast cancer) with endometriosis, therefore FDA approved drugs used in breast cancer and endometriosis were selected for validation of the biomarkers using docking procedure by Autodock vina. In addition GSCA online server results obtained as output was used to study interaction of drugs with three selected genes (PPARG, FOXM1 and FN1). The results of GSCA showed PPARG and FN1 are sensitive towards 23 drugs (AR-42, AT-7519, BMS345541, BX-912, CUDC-101, FK866, I-BET-762, Ispinesib, Mesylate, JW-7-24-1, KIN001-102, Methotrexate, NG-25, PHA-793887, PI-103, PIK-93, Phenformin, QL-X-138, THZ-2-102-1, TPCA-1, TubastatinA, Vorinostat, WZ3105, XMD13-2, GSK690693, and NPK76-II-72-1) whereas FOXM1 was least sensitive towards them but had sensitivity towards RDEA119, Trametinib and selumetinib drugs. The forty-four drugs obtained from the GSCA and LD analysis were subjected for docking studies against all the three proteins related to the biomarkers for validation. The results showed that the drugs THZ-2-102-1, navitoclax, ng25, and trametinib have good binding affinity with all the three proteins whereas vorinostat had least binding affinity with all the three targets which was found to be inconsistent to the GSCA result. This inconsistency in results may be to the difference in the inputs made as the GSCA uses gene information whereas docking is study of protein ligand interaction. Since docking is robust technique we relied on docking results for further study (Table 2). The results revealed that 18 drugs have good binding affinity with proteins pdb id 3M7P, 4Y29, 3G73 associated with *FN1, PPARG,* and *FOXM1* (Figs. 7, 8 Table 2). Docking result showed that most of the drugs have good affinity for *PPARG*, followed by *FN1* and *FOXM1*. For PPARG biomarker (protein pdb id 4Y29), Tucatinib, THZ-2-102-1, Olaparib, Navitoclax, ng25, Trametinib Methotrexate, Abemaciclib, Alpelisib drugs showed best docking score of -11.7, -11.0, -10.6, -10.5, -10.3, -9.3, -9.8, -9.2, -9.6; for FN1 (protein pdb id 3m7p), Tucatinib, THZ-2-102-1, Olaparib, Navitoclax, ng25, Trametinib, Methotrexate, Abemaciclib, Alpelisib, Ribociclib showed best docking score of -10.1, -7.7, -8.7, -7.9, -7.4, -7.0, -9, -9, -8.9, -9.4 and FOXM1 (protein pdb id 3G73,) Tucatinib, THZ-2-102-1, Olaparib, Navitoclax, ng25, Trametinib,Methotrexate, Abemaciclib, Alpelisib, Ribociclib showed best docking score of -7.8, -10.8, -7, -10.6, -10.0, -8.9, -6.9, -6.8, -6.5, -6.5. However, it was observed that five drugs viz. Tucatinib, THZ-2-102-1, Olaparib, Navitoclax, ng25, Trametinib, Methotrexate, Abemaciclib showed good binding affinity against all three target protein.

**Table 2:** Molecular docking analysis results of ligand selected from GSCA and FDA approved drugs for endometriosis and breast cancer against the three targets FN1, FOXM1, and PPARG.

S. no.	Drugs name	3G73 (binding affinity (FOXM1))	3m7p (Binding affinity (FN1))	4y29 (binding affinity (PPARG))
1.	Tucatinib	− 7.8	− 10.1	− 11.7
2.	THZ-2-102-1	− 10.8	− 7.7	− 11.0
3.	Olaparib	− 7	− 8.7	− 10.6
4.	Navitoclax	− 10.6	− 7.9	− 10.5
5.	ng25	− 10.0	− 7.4	− 10.3
6.	Methotrexate	− 6.9	− 9	− 9.8
7.	WZ3105	− 8.8	− 6.6	− 9.6
8.	Alpelisib	− 6.5	− 8.9	− 9.6
9.	FK866	− 7.6	− 6.3	− 9.5
10.	Trametinib	− 8.9	− 7.0	− 9.3
11.	GSK690693	− 8.3	− 6.2	− 9.3
12.	XMD13-2	− 8.2	− 6.8	− 9.3
13.	KIN001-102	− 8.1	− 6.2	− 9.2
14.	Abemaciclib	− 6.8	− 9	− 9.2
15.	PI-103	− 8.6	− 6.7	− 9.0
16.	Raloxifene	− 6.6	− 8.3	− 8.9
17.	I-BET-762	− 7.9	− 6.3	− 8.8
18.	BX-912	− 7.6	− 5.9	− 8.8
19.	Toremifene	− 5.5	− 6.9	− 8.6
20.	Cudc-101	− 8.3	− 6.3	− 8.5
21.	TPCA-1	− 7.3	− 5.8	− 8.5
22.	Fulvestrant	− 4.6	− 7.8	− 8.4
23.	AR-42	− 7.3	− 5.8	− 8.3
24.	Trastuzumab	− 6.7	− 8.8	− 8.3
25.	JW-7-24-1	− 8.5	− 6.2	− 8.2
26.	PHA-793887	− 7.7	− 6.2	− 8.2
27.	Palbociclib	− 6.5	− 8.5	− 8.2
28.	AT-7519	− 7.3	− 5.9	− 7.9
29.	PIK-93	− 7.7	− 5.3	− 7.8
30.	Letrozole	− 5.2	− 6.6	− 7.8
31.	RDEA119	− 8.4	− 5.9	− 7.6
32.	QL-X-138	− 8.5	− 6.9	− 7.5
33.	Ixabepilone	− 6.5	− 8.1	− 7.5
34.	Anastrozole	− 5.4	− 6.6	− 7.5
35.	NPK76-II-72-1	− 8.5	− 6.7	− 7.4
36.	Selumitinib	− 8.4	− 5.6	− 7.3
37.	BMS345541	− 7.0	− 5.8	− 7.3
38.	Capecitabine	− 6.1	− 8	− 7.3
39.	Phenformin	− 6.6	− 5.6	− 7.1
40.	Ribociclib	− 6.5	− 9.4	− 7
41.	vorinostat	− 6.4	− 4.7	− 7.0
42.	Cyclophosphamide	− 4	− 4.9	− 5.7
43.	Fluorouracil	− 4.3	− 5.2	− 5.3
44.	Thiotepa	− 3.7	− 3.8	− 3.7

The binding interactions of Tucatinib and THZ-2-102-1 within active pockets of 3M7P, 3G73, and 4Y29, is shown in Fig. 8. Tucatinib showed hydrogen bond interactions with GLN409 and LEU407; ARG286, HIS287 and CYS285, SER289; pi -pi stacking with PHE531; LEU259, GLY284 in addition to Vander Waal and other interactions in 3M7P, 3G73 and 4Y29 respectively. THZ-2-102-1showed hydrogen bond interactions with ARG286, SER290, HIS287; ARG503, ARG369, HIS532, ALA465; CYS285; pi -pi stacking with ARG286; PHE371; GLN 283 and other interactions with 3M7P, 3G73 and 4Y29 respectively.

## Discussion

This study demonstrates how predicted biomarkers play an integral role in the progression of BC with the support of literature and tried to identify the potential small molecules that can be developed for its treatment. To study the key genes involved in the breast cancer and their association with other disease network biology based approach was taken up in addition to the use of other *in-silico* tools. The network-analyzed approach with breast cancer datasets form Bioexpress database and an independent validation set with 5557 genes was used to generate the genes network. A node file of 532 genes was created out of 5557 genes by using the network analyzer. The module preservation analysis invalidating set revealed that the identified modules were reliable, as all of their summary statistics were above 30. A venn diagram was generated using the Venn Diagram tool based on the results obtained and it could be concluded that three genes (*FN1, FOXM1,* and *PPARG*) are closely related to the target disease. The literature study revealed that the expression of these biomarkers along with the protein related is enhanced in BC.

The involvement of identified overexpresses biomarkers in related biological processes (BPs), molecular functions (MFs), and cellular components (CCs) was studied using network based and integrated statistical methods. It was felt that biological processes, cellular components, and molecular function like protease, serine protease, hormone, motor protein, protease inhibitor, and cell division process would make a good target for the design and development of anti-BC agents.

The expression level of *FN1* has been correlated to an advanced stage of breast cancer and poor clinical outcomes. *FN1* were identified for the first time as cancer stromal key genes associated with breast cancer invasion and metastasis^21^. The *FN1* gene encodes fibronectin 1, an important extracellular matrix glycoprotein that plays a pivotal role in occurrence and development of various tumors. It binds to several members of the family of integrin receptors^22^ thereby activating the breast cancer's PI3K/Akt pathway. Furthermore, *FN1* has been shown to promote cell proliferation and migration in cancers such as esophageal, oral, nasopharyngeal, colorectal, ovarian, renal, and thyroid^23,24^.

The Forkhead Box M1 encoded by *FOXM1* gene is a transcriptional activator involved in cell proliferation and encodes protein which is phosphorylated in M phase and as a result regulates the expression of several cell cycle genes, such as cyclin B1 and cyclin D1^25^. The *FOXM1* gene has been reported to overexpress in aggressive, therapy-resistant variants of hormone receptor-positive and triple-negative breast tumors and correlated with a poor prognosis in a variety of cancers, such as breast cancer and colorectal cancer^25–27^.

The Peroxisome Proliferator-Activated Receptor-Gamma (*PPARG*) is a ligand-activated nuclear hormone receptor that regulates lipid metabolism and insulin sensitivity^28^. By synthesizing high amounts of lipids, ERBB2-positive breast cancer cells generate palmitate-induced lipotoxicity^41^. PPAR is a nuclear hormone transcription factor that regulates the expression of several genes involved in adipogenesis, energy metabolism, proliferation, and growth of tumours. PPARG is the predominant subtype of its family that is expressed in the mammary gland and in primary and metastatic breast cancer, according to references^28–35^.

PPARG is indirectly involved in development of breast cancer through ERBB2 signaling^36^ pathway. It has been reported that inhibition of the PPARG pathway reduces the aldehyde dehydrogenase positive population in ERBB2-positive breast cancer cells supporting its role in development of BC^36^. In numerous malignancies, including colorectal cancer, hepatocellular carcinoma, lung cancer, glioma, and leukaemia, the effects of PPAR activity in CSCs (Cancer stem cells) has been investigated^36^. All these genes are reported overexpressed along with their target proteins in cells. This study was aimed to find out specific inhibitor to control the overexpression of these proteins in the cancer cells. Lately FDA has been approving the single to multi-target drugs in cancer therapy and this move is look at a different scenario, where a new generation of anticancer drugs is capable to prevent more. There is major paradigm shift in drug design and discovery due to number of reasons^26,37^ and one such approach that is gaining fast acceptability is development of multi-target drug. The advantages of multi-target drugs over their single-target counterparts include improved efficacy, a safer profile, simpler administration and patient compliance. Also polypharmacology-guided drug design has been an approach used by scientist for drug development in particularly for repurposing of drugs and multi-targeting.

Taking the concept of multitarget approach the docking of FDA approved anticancer (anti BC and antiendometriasis) agents and drugs identified in GSCA analysis was carried out against the three selected targets. The results obtained presented in Table 1. It could be observed that THZ-2-102-1 and tucatinib showed best binding affinities with all the three target receptors (3M7P, 3G73 and 4Y29) followed by navitoclax, ng25, trametinib, olaparib, and methotrexate contrary to this thiopeta had least binding score with all the target receptors. Interaction analyses of the docked poses of tucatinib^38^ showed that the drug is a persuasive inhibitor of 3M7P, 3G73 and 4Y29. Tucatinib and THZ-2-102-1 could be further taken as lead for development of multitarget drug for BC treatment. Trametinib is already approved by the FDA in May 2013 for the treatment of metastatic melanomas, as well as lung cancer, renal cancer, thyroid cancer, cholangiocarcinoma, and breast cancer^39–42^. These reports further validated our results that the biomarker identified and drug for multiple target can be “the approach” for the disease.

It could be further mentioned that these drugs would be more beneficial in cases suffering from TB as the Linkage studies identified the FN1 gene as a susceptibility for TB^20^, endometriosis^39^. These drugs are not clinical approved against to our targets and may be expanded upon for wet lab studies.

## Materials and methods

The databases and techniques used for identification, analysis and prediction of target biomarker of BC include BioXpress (https://hive.biochemistry.gwu.edu/bioxpress), DAVID's functional annotation tool (https://david.ncifcrf.gov/), Cytoscape (https://cytoscape.org/), KM plotter database (https://kmplot.com/analysis/), International Hapmap Project database (https://ftp.ncbi.nlm.nih.gov/), Haploview tool (https://www.broadinstitute.org/haploview/haploview) and Venn Diagram tool (https://bioinformatics.psb.ugent.be/webtools/Venn/).

### Identification and selection of BC-associated genes

BioXpress is a differential expression database for cancer where RNA-seq and miRNA-derived read counts have been evaluated for differential expression. The current version of BioXpress incorporates mRNA and miRNA-derived expression from TCGA and ICGC^16^. BioExpress database have filter feature like search type, cancer type, feature type, trend with significance cutoff to retrieve cancer dataset. To retrieve cancer dataset from the database, filters like cancer type, breast cancer, mRNA with adjusted p-value were used and the output file obtained was further used for the study.

### Analysis of functional association

The Database for Annotation, Visualization and Integrated Discovery (DAVID) provides a comprehensive set of functional annotation tools for investigators to understand the biological meaning behind large lists of genes^43^. DAVID's was used to analyze modules at all levels of the hierarchy^44^. The pathways and functions with a corrected *p* < 0.05 were deemed statistically significant.

### Construction of protein–protein interaction (PPI) network

Out of 11,053 genes identified which were significantly overexpressed in BC patients from BioXpress^16^ (FC > 1, adjusted *p* < 0.05), 5557 genes (Table S1) were used for an interactome network using the STRING app in Cytoscape 3.6.016^18,19^. The network was extracted only for the physical interaction network, which represents the protein–protein interaction network of BC-associated genes. A protein–protein interaction network using 2960 nodes and 20,372 edges was constructed as a primary network. Venn diagram server was used to show and identify the relationships among lists of degree, betweenness, and closeness centrality of the node file.

### Survival analysis

KM plotter database^45^ (https://kmplot.com/analysis/) was queried for assessing the prognosis of these functionally enriched module genes in network approach. KM survival plots along with 95% confidence interval (95% CI), hazard ratio (HR), number-at-risk, and log-rank *p-*values of enriched module-genes were presented by splitting network approach into lower and higher expression groups, respectively.

### Analysis of LD and haplotype

Linkage disequilibrium (LD) plays a key role in a wide range of mapping disease associations to demographic history estimation or trail-associated genes^46,47^. Haplotype blocks reveal information on the combination of alleles or a set of single nucleotide polymorphisms (SNPs) found on the same chromosome and aid investigators in localizing disease-causing genes and loci. The Haploview tool^48^ was used to study LD, and haplotype block for Han Chinese (CHB) genotype data retrieved from the International Hapmap Project^49^. The genotype data was visualized and examined for linkages and generation of LD and haplotype blocks.

### Biomarker guided drug repositioning

In order to identify potential anti BC agents two approaches were used; i.e. Gene Set Cancer Analysis (GSCA) and ii. Molecular docking. To visualize and analyze the correlation between the identified biomarkers and drugs, GSCA (http://bioinfo.life.hust.edu.cn/GSCA/#/) an online tool was used. On the basis of Spearman Correlation and FDR value (between drugs and targets) drugs were selected for further study from GSCA analysis. This approach may be helpful in improving the efficacy or safety related to drugs. Further that the LD and haplotype studies revealed that endometriosis is associated with our target key genes^39^. Therefore, FDA-approved drugs used in BC and endometriosis were selected for further studies. Molecular docking studies being a significant *in-silico* technique for validating the drug-target binding interaction^50,51^ was used to validate the selected drugs for multitarget purposing. The 3D structure of target proteins (PBD ID- 4Y29, 3G73, 3M7P) was downloaded from Protein Data Bank (PDB) (https://www.rcsb.org/)^52^ and used for docking studies. The structure of identified/ selected drugs from GSCA and FDA-approved drugs used in BC and endometriosis were download from the PubChem databases (https://pubchem.ncbi.nlm.nih.gov/)^53^. The AutoDock-vina^26^ software was used to examine the structural binding performance between receptor and drugs by computing affinity scores (kcal/mol). Discovery Studio Visualizer was used to visualize 3D protein–ligand interaction.

## Conclusions

In this study, using network approach the key genes as breast cancer regulators were identified. The GO-terms (BPs, MFs and CCs) and key genes regulatory network analyses highlighted as pathogenetic process of BC progression. Our study also suggested two biomarkers-guided tucatinib and THZ-2-102-1 drugs which might be effective in inhibiting BC-causing proteins (FN1, FOXM1, and PPARG). The literature review and molecular docking analysis were used to validate our findings. The findings of our in-silico study may potentially lead to insights into the molecular mechanisms that drive BC progression and potential therapeutic agents.

### Supplementary Information

Supplementary Information.

## Data Availability

The breast cancer gene data utilized in this study are openly available in the BioXpress database at https://hive.biochemistry.gwu.edu/bioxpress. The DAVID database, STRING, Venn Diagram tool, KM plotter database, International Hapmap Project database, Haploview tool used in the present study are available at https://david.ncifcrf.gov/summary.jsp, https://string-db.org/cgi/input?sessionId=biQZA8ZybYYn,https://bioinformatics.psb.ugent.be/cgibin/liste/Venn/calculate_venn.htpl,https://kmplot.com/analysis/index.php?p=service,https://ftp.ncbi.nlm.nih.gov/hapmap/genotypes/200807_phaseIII/hapmap_format/forward/,https://www.broadinstitute.org/haploview/haploview respectively. The protein structure of FN1, PPARG, FOXM1 proteins used in the present study, obtained from the PDB (Protein Data Bank) are available at and https://www.rcsb.org/structure/4Y29, https://www.rcsb.org/structure/3G73, https:// www.rcsb.org/structure/3M7P). GSCA and pubchem used in the present study to obtained drugs from the http://bioinfo.life.hust.edu.cn/GSCA/#/drug and https://pubchem.ncbi.nlm.nih.gov/#query=tucatinib respectively. All data generated in the present study is present in the article and supplementary information.

## Abbreviations

BC: Breast cancer
HR: Hazard ratio
DR: Drug repurposing
SNP: Single nucleotide polymorphism
