# Supplementary material for: Predicting the molecular mechanism-driven progression of breast cancer through comprehensive network pharmacology and molecular docking approach
Source: Sci Rep. 2023 Aug 22;13:13729. doi: 10.1038/s41598-023-40684-7 (PMC10444824; doi:10.1038/s41598-023-40684-7)
Supplement: Supplementary file 1 — Supplementary Information. [file 41598_2023_40684_MOESM1_ESM.docx]

**Predicting the molecular mechanism-driven progression of breast cancer through comprehensive network pharmacology and molecular docking approach**

Bharti Vyas^1^, Sunil kumar^2^, Ratul Bhowmik^3^, Mymoona Akhter^3*^

^1^School of Interdisciplinary Science and Technology, Jamia Hamdard, New Delhi, India

^2^ICAR-Indian Institute of Farming System Research, Modipuram Meerut 250110

^3^Department of Pharmaceutical Chemistry, School of Pharmaceutical Education and Research, Jamia Hamdard, New Delhi, 110062, India

^*^Correspondence:

Mymoona Akhter

Professor

Department of Pharmaceutical Chemistry, School of Pharmaceutical Education and Research, Jamia Hamdard, New Delhi– 110062

**Table S1. List of gene (breas cancer) retrieve from bioxpress**

| Primary Feature | Feature Name | Subjects Ratio | log2FC | P-Value | Adjusted P-Value | Significance | Expression Trend | PMID List |
| --- | --- | --- | --- | --- | --- | --- | --- | --- |
| Q8NFA2 | NOXO1 | 63/114(55.26) | 0.62 | 2.21E-04 | 6.38E-04 | Yes | Up | - |
| Q7L8J4 | SH3BP5L | 58/114(50.88) | 0.33 | 1.38E-06 | 6.83E-06 | Yes | Up | - |
| Q07654 | TFF3 | 56/114(49.12) | 1.11 | 6.92E-07 | 3.71E-06 | Yes | Up | - |
| P18850 | ATF6 | 53/114(46.49) | 0.25 | 9.03E-05 | 2.87E-04 | Yes | Up | - |
| P13612 | ITGA4 | 60/114(52.63) | 0.26 | 5.36E-03 | 1.11E-02 | Yes | Up | - |
| Q9NSP4 | CENPM | 65/114(57.02) | 1.48 | 5.81E-14 | 3.08E-12 | Yes | Up | - |
| Q9GZT3 | C14ORF156 | 65/114(57.02) | 0.25 | 1.61E-04 | 4.83E-04 | Yes | Up | - |
| P20839 | IMPDH1 | 60/114(52.63) | 0.33 | 3.63E-04 | 9.97E-04 | Yes | Up | - |
| Q9UBC2 | EPS15L1 | 61/114(53.51) | 0.43 | 2.45E-11 | 4.98E-10 | Yes | Up | - |
| Q9H6W3 | C14ORF169 | 63/114(55.26) | 0.36 | 5.85E-09 | 5.56E-08 | Yes | Up | - |
| Q13444 | ADAM15 | 65/114(57.02) | 0.44 | 2.14E-06 | 1.01E-05 | Yes | Up | - |
| Q14654 | KCNJ11 | 58/114(50.88) | 0.79 | 7.28E-07 | 3.87E-06 | Yes | Up | - |
| Q9ULQ0 | FAM40B | 65/114(57.02) | 0.73 | 3.19E-06 | 1.44E-05 | Yes | Up | - |
| P29597 | TYK2 | 61/114(53.51) | 0.23 | 3.46E-06 | 1.54E-05 | Yes | Up | - |
| Q92611 | EDEM1 | 50/114(43.86) | 0.13 | 2.40E-02 | 4.23E-02 | Yes | Up | - |
| P48664 | SLC1A6 | 50/114(43.86) | 0.95 | 1.50E-03 | 3.55E-03 | Yes | Up | - |
| Q8NAA6 | C15ORF53 | 41/114(35.96) | 0.73 | 9.84E-03 | 1.91E-02 | Yes | Up | - |
| O60486 | PLXNC1 | 55/114(48.25) | 0.41 | 2.57E-04 | 7.33E-04 | Yes | Up | - |
| P35226 | BMI1 | 50/114(43.86) | 0.32 | 1.41E-04 | 4.30E-04 | Yes | Up | - |
| Q8TDR4 | TCP10L | 59/114(51.75) | 0.37 | 1.87E-04 | 5.51E-04 | Yes | Up | - |
| Q96M29 | TEKT5 | 63/114(55.26) | 0.78 | 1.54E-05 | 5.92E-05 | Yes | Up | - |
| P35250 | RFC2 | 65/114(57.02) | 0.49 | 3.16E-09 | 3.25E-08 | Yes | Up | - |
| Q9BRP0 | OVOL2 | 55/114(48.25) | 0.57 | 2.71E-03 | 6.02E-03 | Yes | Up | - |
| Q14344 | GNA13 | 51/114(44.74) | 0.15 | 1.50E-02 | 2.79E-02 | Yes | Up | - |
| Q8IY31 | IFT20 | 54/114(47.37) | 0.2 | 9.04E-04 | 2.26E-03 | Yes | Up | - |
| Q92826 | HOXB13 | 57/114(50.0) | 1.52 | 2.25E-05 | 8.27E-05 | Yes | Up | - |
| Q96E29 | MTERFD1 | 60/114(52.63) | 0.29 | 3.61E-04 | 9.92E-04 | Yes | Up | - |
| Q3Y452 | TDRG1 | 35/114(30.7) | 1.45 | 2.43E-05 | 8.86E-05 | Yes | Up | - |
| Q8N205 | C19ORF46 | 60/114(52.63) | 0.69 | 2.28E-04 | 6.57E-04 | Yes | Up | - |
| P32942 | ICAM3 | 70/114(61.4) | 0.27 | 3.46E-03 | 7.49E-03 | Yes | Up | - |
| Q6ZMG9 | LASS6 | 57/114(50.0) | 0.6 | 1.58E-06 | 7.70E-06 | Yes | Up | - |
| Q15274 | QPRT | 63/114(55.26) | 0.49 | 7.72E-04 | 1.96E-03 | Yes | Up | - |
| Q13487 | SNAPC2 | 66/114(57.89) | 0.53 | 3.57E-12 | 9.55E-11 | Yes | Up | - |
| Q5JPI3 | C3ORF38 | 58/114(50.88) | 0.12 | 1.22E-02 | 2.32E-02 | Yes | Up | - |
| Q86SS6 | SYT9 | 55/114(48.25) | 1.28 | 3.55E-08 | 2.70E-07 | Yes | Up | - |
| P22492 | HIST1H1T | 40/114(35.09) | 2.04 | 9.67E-10 | 1.16E-08 | Yes | Up | - |
| Q6UWV7 | FAM159A | 66/114(57.89) | 0.62 | 1.37E-05 | 5.31E-05 | Yes | Up | - |
| P09668 | CTSH | 63/114(55.26) | 0.32 | 5.14E-05 | 1.73E-04 | Yes | Up | - |
| Q2WGJ8 | C8ORFK29 | 58/114(50.88) | 0.9 | 4.76E-07 | 2.66E-06 | Yes | Up | - |
| O94992 | HEXIM1 | 58/114(50.88) | 0.17 | 5.30E-03 | 1.10E-02 | Yes | Up | - |
| Q8NHU6 | TDRD7 | 58/114(50.88) | 0.12 | 2.65E-02 | 4.62E-02 | Yes | Up | - |
| Q969S6 | TMEM203 | 60/114(52.63) | 0.13 | 3.23E-03 | 7.03E-03 | Yes | Up | - |
| Q5T6V5 | C9ORF64 | 56/114(49.12) | 0.21 | 5.87E-04 | 1.53E-03 | Yes | Up | - |
| Q9BPW8 | NIPSNAP1 | 59/114(51.75) | 0.5 | 5.62E-07 | 3.09E-06 | Yes | Up | - |
| Q8IXS2 | CCDC65 | 65/114(57.02) | 0.46 | 6.07E-03 | 1.24E-02 | Yes | Up | - |
| Q9ULZ2 | STAP1 | 66/114(57.89) | 0.54 | 1.98E-03 | 4.56E-03 | Yes | Up | - |
| P68400 | CSNK2A1 | 59/114(51.75) | 0.2 | 1.57E-03 | 3.70E-03 | Yes | Up | - |
| Q8N6T0 | C11ORF80 | 64/114(56.14) | 0.4 | 7.45E-04 | 1.90E-03 | Yes | Up | - |
| Q9Y4I5 | MTL5 | 55/114(48.25) | 1.01 | 2.90E-09 | 3.01E-08 | Yes | Up | - |
| P61019 | RAB2A | 55/114(48.25) | 0.2 | 4.09E-03 | 8.70E-03 | Yes | Up | - |
| Q8NCL4 | GALNT6 | 63/114(55.26) | 0.94 | 1.00E-07 | 6.72E-07 | Yes | Up | - |
| Q7Z6I5 | SPATA12 | 62/114(54.39) | 0.41 | 2.94E-03 | 6.47E-03 | Yes | Up | - |
| Q8NDV3 | SMC1B | 59/114(51.75) | 0.91 | 6.76E-06 | 2.82E-05 | Yes | Up | - |
| Q8WVR3 | C7ORF43 | 64/114(56.14) | 0.42 | 4.26E-08 | 3.17E-07 | Yes | Up | - |
| Q9HBH0 | RHOF | 63/114(55.26) | 0.92 | 4.97E-09 | 4.82E-08 | Yes | Up | - |
| Q9BPU9 | B9D2 | 72/114(63.16) | 0.41 | 4.53E-08 | 3.33E-07 | Yes | Up | - |
| P01037 | CST1 | 59/114(51.75) | 2.06 | 2.74E-08 | 2.15E-07 | Yes | Up | - |
| Q53TM1 | SF3B14 | 59/114(51.75) | 0.18 | 4.35E-03 | 9.22E-03 | Yes | Up | - |
| Q14CS0 | UBXN2B | 56/114(49.12) | 0.21 | 5.44E-03 | 1.13E-02 | Yes | Up | - |
| Q16186 | ADRM1 | 61/114(53.51) | 0.44 | 1.13E-07 | 7.45E-07 | Yes | Up | - |
| P15880 | RPS2 | 60/114(52.63) | 0.19 | 1.59E-02 | 2.94E-02 | Yes | Up | - |
| Q9UMX2 | OAZ3 | 63/114(55.26) | 0.68 | 1.97E-07 | 1.22E-06 | Yes | Up | - |
| Q9Y496 | KIF3A | 56/114(49.12) | 0.22 | 1.36E-02 | 2.54E-02 | Yes | Up | - |
| Q8N9M1 | C19ORF47 | 62/114(54.39) | 0.19 | 3.74E-03 | 8.01E-03 | Yes | Up | - |
| Q96I34 | PPP1R16A | 67/114(58.77) | 0.37 | 6.83E-05 | 2.23E-04 | Yes | Up | - |
| O14593 | RFXANK | 71/114(62.28) | 0.47 | 2.62E-12 | 7.31E-11 | Yes | Up | - |
| Q96C01 | FAM136A | 56/114(49.12) | 0.22 | 1.41E-03 | 3.36E-03 | Yes | Up | - |
| Q10469 | MGAT2 | 61/114(53.51) | 0.18 | 6.37E-04 | 1.65E-03 | Yes | Up | - |
| Q9H967 | WDR76 | 60/114(52.63) | 0.63 | 3.72E-08 | 2.81E-07 | Yes | Up | - |
| Q96RY7 | IFT140 | 59/114(51.75) | 0.38 | 2.35E-05 | 8.61E-05 | Yes | Up | - |
| Q86Y97 | SUV420H2 | 68/114(59.65) | 0.45 | 4.49E-08 | 3.31E-07 | Yes | Up | - |
| Q76KD6 | SPATC1 | 58/114(50.88) | 0.71 | 9.93E-06 | 3.99E-05 | Yes | Up | - |
| Q96CS3 | FAF2 | 60/114(52.63) | 0.2 | 8.50E-04 | 2.14E-03 | Yes | Up | - |
| - | LOC283867 | 59/114(51.75) | 2.44 | 1.86E-17 | 4.34E-15 | Yes | Up | - |
| Q9UIU6 | SIX4 | 55/114(48.25) | 0.7 | 6.09E-07 | 3.31E-06 | Yes | Up | - |
| Q63HQ0 | AP1AR | 50/114(43.86) | 0.27 | 6.23E-04 | 1.62E-03 | Yes | Up | - |
| Q04637 | EIF4G1 | 56/114(49.12) | 0.23 | 8.64E-04 | 2.17E-03 | Yes | Up | - |
| Q9BQ61 | C19ORF43 | 62/114(54.39) | 0.15 | 1.07E-02 | 2.06E-02 | Yes | Up | - |
| P43355 | MAGEA1 | 36/114(31.58) | 0.99 | 2.79E-02 | 4.84E-02 | Yes | Up | - |
| Q9UPU3 | SORCS3 | 52/114(45.61) | 1.08 | 3.76E-05 | 1.31E-04 | Yes | Up | - |
| Q5K651 | SAMD9 | 57/114(50.0) | 0.42 | 2.62E-04 | 7.45E-04 | Yes | Up | - |
| O95936 | EOMES | 55/114(48.25) | 0.4 | 1.61E-02 | 2.96E-02 | Yes | Up | - |
| A5A3E0 | POTEF | 59/114(51.75) | 0.35 | 8.92E-03 | 1.75E-02 | Yes | Up | - |
| Q6IA69 | NADSYN1 | 60/114(52.63) | 0.26 | 9.87E-05 | 3.11E-04 | Yes | Up | - |
| Q9H1P6 | C20ORF85 | 26/114(22.81) | 1.31 | 6.47E-03 | 1.32E-02 | Yes | Up | - |
| Q969I3 | GLYATL1 | 59/114(51.75) | 0.68 | 4.20E-03 | 8.92E-03 | Yes | Up | - |
| O95394 | PGM3 | 53/114(46.49) | 0.18 | 2.37E-02 | 4.19E-02 | Yes | Up | - |
| P10619 | CTSA | 59/114(51.75) | 0.33 | 1.21E-04 | 3.73E-04 | Yes | Up | - |
| Q9BRJ7 | NUDT16L1 | 66/114(57.89) | 0.66 | 1.35E-12 | 4.14E-11 | Yes | Up | - |
| Q4KMQ1 | TPRN | 64/114(56.14) | 1.12 | 1.02E-16 | 1.73E-14 | Yes | Up | - |
| Q9NY12 | GAR1 | 55/114(48.25) | 0.14 | 1.45E-02 | 2.71E-02 | Yes | Up | - |
| P11802 | CDK4 | 60/114(52.63) | 0.31 | 7.81E-09 | 7.15E-08 | Yes | Up | - |
| P41252 | IARS | 55/114(48.25) | 0.21 | 1.69E-03 | 3.96E-03 | Yes | Up | - |
| Q8N9T8 | KRI1 | 63/114(55.26) | 0.34 | 2.70E-06 | 1.24E-05 | Yes | Up | - |
| A6NMX2 | EIF4E1B | 32/114(28.07) | 1.78 | 2.45E-05 | 8.93E-05 | Yes | Up | - |
| Q8IWU4 | SLC30A8 | 56/114(49.12) | 1.35 | 1.38E-06 | 6.84E-06 | Yes | Up | - |
| P51570 | GALK1 | 61/114(53.51) | 0.31 | 1.94E-04 | 5.70E-04 | Yes | Up | - |
| P59020 | DSCR9 | 55/114(48.25) | 0.37 | 1.58E-02 | 2.92E-02 | Yes | Up | - |
| Q6PJF5 | RHBDF2 | 61/114(53.51) | 0.47 | 1.07E-07 | 7.14E-07 | Yes | Up | - |
| Q499Z4 | ZNF672 | 66/114(57.89) | 0.44 | 1.66E-09 | 1.84E-08 | Yes | Up | - |
| P30519 | HMOX2 | 61/114(53.51) | 0.4 | 4.17E-09 | 4.13E-08 | Yes | Up | - |
| P13611 | VCAN | 58/114(50.88) | 1.08 | 6.00E-11 | 1.06E-09 | Yes | Up | - |
| Q9H6Z4 | RANBP3 | 69/114(60.53) | 0.13 | 1.07E-03 | 2.64E-03 | Yes | Up | - |
| Q96AE4 | FUBP1 | 50/114(43.86) | 0.19 | 6.99E-04 | 1.79E-03 | Yes | Up | - |
| Q8TAI1 | C18ORF56 | 60/114(52.63) | 1.11 | 1.06E-11 | 2.44E-10 | Yes | Up | - |
| Q8N884 | C6ORF150 | 60/114(52.63) | 0.41 | 7.43E-06 | 3.07E-05 | Yes | Up | - |
| Q5T280 | C9ORF114 | 70/114(61.4) | 0.2 | 2.04E-04 | 5.94E-04 | Yes | Up | - |
| Q9NZZ3 | CHMP5 | 63/114(55.26) | 0.16 | 2.21E-03 | 5.03E-03 | Yes | Up | - |
| Q969N2 | PIGT | 67/114(58.77) | 0.37 | 5.01E-06 | 2.15E-05 | Yes | Up | - |
| P43080 | GUCA1A | 53/114(46.49) | 0.56 | 2.58E-02 | 4.52E-02 | Yes | Up | - |
| Q9BUR4 | WRAP53 | 65/114(57.02) | 0.19 | 1.57E-04 | 4.72E-04 | Yes | Up | - |
| Q9HCU4 | CELSR2 | 57/114(50.0) | 0.55 | 4.15E-05 | 1.43E-04 | Yes | Up | - |
| A4D1W6 | C7ORF11 | 62/114(54.39) | 0.16 | 1.31E-03 | 3.16E-03 | Yes | Up | - |
| Q5T5X7 | BEND3 | 54/114(47.37) | 0.24 | 2.40E-02 | 4.23E-02 | Yes | Up | - |
| Q16778 | HIST2H2BE | 58/114(50.88) | 0.9 | 4.80E-10 | 6.34E-09 | Yes | Up | - |
| O95201 | ZNF205 | 66/114(57.89) | 0.34 | 2.94E-06 | 1.34E-05 | Yes | Up | - |
| Q86Y33 | CDC20B | 53/114(46.49) | 2.74 | 6.09E-17 | 1.14E-14 | Yes | Up | - |
| Q9BSV6 | TSEN34 | 68/114(59.65) | 0.4 | 4.31E-08 | 3.20E-07 | Yes | Up | - |
| Q8WWR8 | NEU4 | 61/114(53.51) | 1.01 | 1.01E-05 | 4.06E-05 | Yes | Up | - |
| Q14728 | MFSD10 | 63/114(55.26) | 0.38 | 4.30E-07 | 2.43E-06 | Yes | Up | - |
| O60356 | NUPR1 | 63/114(55.26) | 0.3 | 3.40E-03 | 7.35E-03 | Yes | Up | - |
| P04792 | HSPB1 | 62/114(54.39) | 0.75 | 1.24E-08 | 1.07E-07 | Yes | Up | - |
| Q8TD08 | MAPK15 | 58/114(50.88) | 1.16 | 1.46E-07 | 9.37E-07 | Yes | Up | - |
| P10176 | COX8A | 61/114(53.51) | 0.29 | 5.10E-04 | 1.35E-03 | Yes | Up | - |
| Q5FBB7 | SGOL1 | 57/114(50.0) | 1.53 | 5.12E-12 | 1.29E-10 | Yes | Up | - |
| O00222 | GRM8 | 63/114(55.26) | 1.56 | 1.66E-10 | 2.52E-09 | Yes | Up | - |
| Q9H3R2 | MUC13 | 52/114(45.61) | 1.29 | 5.43E-07 | 3.00E-06 | Yes | Up | - |
| O14656 | TOR1A | 64/114(56.14) | 0.19 | 1.29E-05 | 5.03E-05 | Yes | Up | - |
| Q7Z7H5 | TMED4 | 61/114(53.51) | 0.13 | 1.34E-02 | 2.52E-02 | Yes | Up | - |
| Q15973 | ZNF124 | 55/114(48.25) | 0.39 | 2.48E-05 | 9.04E-05 | Yes | Up | - |
| Q53HF9 | LASS4 | 58/114(50.88) | 0.55 | 5.09E-06 | 2.18E-05 | Yes | Up | - |
| Q9H7T9 | C1ORF135 | 60/114(52.63) | 1 | 5.46E-10 | 7.08E-09 | Yes | Up | - |
| O95182 | NDUFA7 | 64/114(56.14) | 0.44 | 2.67E-07 | 1.60E-06 | Yes | Up | - |
| Q13162 | PRDX4 | 57/114(50.0) | 0.2 | 2.66E-02 | 4.63E-02 | Yes | Up | - |
| O00631 | SLN | 60/114(52.63) | 0.66 | 4.60E-03 | 9.69E-03 | Yes | Up | - |
| - | NAPSB | 63/114(55.26) | 0.47 | 4.66E-04 | 1.25E-03 | Yes | Up | - |
| Q00887 | PSG9 | 39/114(34.21) | 0.7 | 1.80E-02 | 3.28E-02 | Yes | Up | - |
| Q96PZ2 | FAM111A | 51/114(44.74) | 0.18 | 1.35E-02 | 2.53E-02 | Yes | Up | - |
| Q13541 | EIF4EBP1 | 63/114(55.26) | 0.4 | 4.00E-04 | 1.09E-03 | Yes | Up | - |
| O75787 | ATP6AP2 | 55/114(48.25) | 0.17 | 5.89E-03 | 1.21E-02 | Yes | Up | - |
| Q15077 | P2RY6 | 62/114(54.39) | 0.67 | 1.93E-06 | 9.21E-06 | Yes | Up | - |
| Q9NPF2 | CHST11 | 61/114(53.51) | 0.74 | 9.97E-10 | 1.19E-08 | Yes | Up | - |
| Q9NQ84 | GPRC5C | 60/114(52.63) | 0.37 | 4.37E-03 | 9.26E-03 | Yes | Up | - |
| Q9H171 | ZBP1 | 60/114(52.63) | 0.65 | 5.24E-05 | 1.76E-04 | Yes | Up | - |
| B2RXF5 | ZBTB42 | 57/114(50.0) | 0.52 | 4.34E-07 | 2.45E-06 | Yes | Up | - |
| Q6UY01 | LRRC31 | 55/114(48.25) | 1.35 | 2.79E-06 | 1.28E-05 | Yes | Up | - |
| Q9Y5J1 | UTP18 | 54/114(47.37) | 0.32 | 2.21E-05 | 8.13E-05 | Yes | Up | - |
| Q9P1Z3 | HCN3 | 61/114(53.51) | 0.21 | 2.19E-02 | 3.90E-02 | Yes | Up | - |
| Q9P0S2 | COX16 | 65/114(57.02) | 0.19 | 1.14E-03 | 2.77E-03 | Yes | Up | - |
| Q96PK6 | RBM14 | 67/114(58.77) | 0.14 | 3.67E-04 | 1.01E-03 | Yes | Up | - |
| Q9H160 | ING2 | 58/114(50.88) | 0.27 | 3.44E-05 | 1.21E-04 | Yes | Up | - |
| Q9UHV5 | RAPGEFL1 | 66/114(57.89) | 0.59 | 1.85E-06 | 8.87E-06 | Yes | Up | - |
| P23193 | TCEA1 | 63/114(55.26) | 0.22 | 7.74E-04 | 1.97E-03 | Yes | Up | - |
| P11172 | UMPS | 60/114(52.63) | 0.24 | 6.05E-06 | 2.55E-05 | Yes | Up | - |
| P63272 | SUPT4H1 | 54/114(47.37) | 0.26 | 2.65E-04 | 7.53E-04 | Yes | Up | - |
| O75717 | WDHD1 | 53/114(46.49) | 0.65 | 2.58E-08 | 2.05E-07 | Yes | Up | - |
| Q86UN6 | AKAP14 | 35/114(30.7) | 1.29 | 2.40E-04 | 6.88E-04 | Yes | Up | - |
| - | LOC100144603 | 57/114(50.0) | 0.51 | 3.25E-05 | 1.15E-04 | Yes | Up | - |
| P49427 | CDC34 | 59/114(51.75) | 0.23 | 1.81E-03 | 4.21E-03 | Yes | Up | - |
| P39210 | MPV17 | 59/114(51.75) | 0.17 | 2.45E-03 | 5.50E-03 | Yes | Up | - |
| P41208 | CETN2 | 58/114(50.88) | 0.29 | 1.79E-05 | 6.75E-05 | Yes | Up | - |
| O00268 | TAF4 | 57/114(50.0) | 0.24 | 1.29E-04 | 3.96E-04 | Yes | Up | - |
| Q53HL2 | CDCA8 | 58/114(50.88) | 1.42 | 1.11E-14 | 7.95E-13 | Yes | Up | - |
| Q96K37 | SLC35E1 | 53/114(46.49) | 0.21 | 5.24E-04 | 1.39E-03 | Yes | Up | - |
| P16104 | H2AFX | 69/114(60.53) | 0.88 | 8.85E-13 | 2.90E-11 | Yes | Up | - |
| Q3ZCQ3 | FAM174B | 54/114(47.37) | 0.47 | 3.57E-04 | 9.83E-04 | Yes | Up | - |
| Q96NX5 | CAMK1G | 55/114(48.25) | 0.62 | 2.11E-04 | 6.12E-04 | Yes | Up | - |
| Q15370 | TCEB2 | 62/114(54.39) | 0.54 | 1.35E-10 | 2.11E-09 | Yes | Up | - |
| O75362 | ZNF217 | 53/114(46.49) | 0.43 | 3.26E-06 | 1.47E-05 | Yes | Up | - |
| P36542 | ATP5C1 | 59/114(51.75) | 0.15 | 2.26E-02 | 4.01E-02 | Yes | Up | - |
| Q9NXS2 | QPCTL | 69/114(60.53) | 0.31 | 5.54E-05 | 1.85E-04 | Yes | Up | - |
| Q8IYL9 | GPR65 | 61/114(53.51) | 0.37 | 2.55E-04 | 7.26E-04 | Yes | Up | - |
| P07996 | THBS1 | 60/114(52.63) | 0.25 | 6.02E-03 | 1.23E-02 | Yes | Up | - |
| - | CDKN2BAS | 55/114(48.25) | 0.92 | 1.28E-07 | 8.34E-07 | Yes | Up | - |
| P05109 | S100A8 | 59/114(51.75) | 0.52 | 1.60E-02 | 2.94E-02 | Yes | Up | - |
| Q92599 | Sep-08 | 59/114(51.75) | 0.37 | 5.50E-08 | 3.96E-07 | Yes | Up | - |
| Q12950 | FOXD4 | 62/114(54.39) | 0.51 | 1.90E-04 | 5.58E-04 | Yes | Up | - |
| Q9BXR0 | QTRT1 | 65/114(57.02) | 0.19 | 1.12E-02 | 2.15E-02 | Yes | Up | - |
| Q5VV41 | ARHGEF16 | 59/114(51.75) | 0.62 | 9.00E-08 | 6.11E-07 | Yes | Up | - |
| - | LOC728758 | 65/114(57.02) | 0.26 | 2.21E-04 | 6.38E-04 | Yes | Up | - |
| P14902 | IDO1 | 57/114(50.0) | 0.5 | 8.62E-03 | 1.70E-02 | Yes | Up | - |
| Q4V9L6 | TMEM119 | 58/114(50.88) | 0.39 | 1.51E-03 | 3.57E-03 | Yes | Up | - |
| Q8NEP3 | LRRC50 | 62/114(54.39) | 0.73 | 1.21E-04 | 3.73E-04 | Yes | Up | - |
| Q0VDD8 | DNAH14 | 57/114(50.0) | 0.86 | 1.16E-08 | 1.00E-07 | Yes | Up | - |
| O00232 | PSMD12 | 51/114(44.74) | 0.35 | 5.88E-05 | 1.96E-04 | Yes | Up | - |
| Q14114 | LRP8 | 59/114(51.75) | 0.65 | 7.47E-06 | 3.09E-05 | Yes | Up | - |
| P49411 | TUFM | 68/114(59.65) | 0.26 | 5.75E-05 | 1.91E-04 | Yes | Up | - |
| Q96PC3 | AP1S3 | 58/114(50.88) | 0.57 | 2.03E-05 | 7.55E-05 | Yes | Up | - |
| Q9H1H1 | GTSF1L | 43/114(37.72) | 0.71 | 2.78E-02 | 4.83E-02 | Yes | Up | - |
| P0C7I6 | CCDC159 | 62/114(54.39) | 0.35 | 2.94E-05 | 1.05E-04 | Yes | Up | - |
| Q96MD2 | C12ORF66 | 54/114(47.37) | 0.19 | 1.14E-02 | 2.18E-02 | Yes | Up | - |
| P0C7X2 | ZNF688 | 64/114(56.14) | 0.28 | 1.90E-04 | 5.58E-04 | Yes | Up | - |
| Q9BVT8 | TMUB1 | 69/114(60.53) | 0.37 | 9.61E-07 | 4.96E-06 | Yes | Up | - |
| Q9NQM4 | CXORF41 | 24/114(21.05) | 1.19 | 2.92E-03 | 6.42E-03 | Yes | Up | - |
| Q9Y6J8 | STYXL1 | 65/114(57.02) | 0.47 | 8.15E-11 | 1.37E-09 | Yes | Up | - |
| O00487 | PSMD14 | 60/114(52.63) | 0.36 | 4.36E-05 | 1.49E-04 | Yes | Up | - |
| P12314 | FCGR1A | 62/114(54.39) | 1.09 | 1.43E-10 | 2.22E-09 | Yes | Up | - |
| Q9UI30 | TRMT112 | 63/114(55.26) | 0.36 | 2.92E-08 | 2.28E-07 | Yes | Up | - |
| Q9NPR2 | SEMA4B | 63/114(55.26) | 0.5 | 1.54E-05 | 5.91E-05 | Yes | Up | - |
| Q9BSY9 | PPPDE1 | 57/114(50.0) | 0.26 | 7.48E-04 | 1.91E-03 | Yes | Up | - |
| A6NFT4 | CCDC42B | 63/114(55.26) | 0.47 | 6.21E-03 | 1.27E-02 | Yes | Up | - |
| Q7Z417 | NUFIP2 | 50/114(43.86) | 0.22 | 1.61E-03 | 3.78E-03 | Yes | Up | - |
| Q8IWX8 | CHERP | 62/114(54.39) | 0.24 | 9.27E-06 | 3.75E-05 | Yes | Up | - |
| Q8TCB0 | IFI44 | 59/114(51.75) | 0.33 | 4.70E-03 | 9.87E-03 | Yes | Up | - |
| O75635 | SERPINB7 | 45/114(39.47) | 1.21 | 1.75E-06 | 8.42E-06 | Yes | Up | - |
| O60828 | PQBP1 | 56/114(49.12) | 0.16 | 8.20E-03 | 1.63E-02 | Yes | Up | - |
| Q9H4K7 | GTPBP5 | 69/114(60.53) | 0.36 | 1.17E-06 | 5.91E-06 | Yes | Up | - |
| P84077 | ARF1 | 64/114(56.14) | 0.55 | 5.30E-11 | 9.49E-10 | Yes | Up | - |
| Q96D31 | ORAI1 | 68/114(59.65) | 0.25 | 3.34E-04 | 9.25E-04 | Yes | Up | - |
| Q96DC9 | OTUB2 | 53/114(46.49) | 0.67 | 1.95E-09 | 2.12E-08 | Yes | Up | - |
| C5NM88 | KIAA1244 | 51/114(44.74) | 0.57 | 4.28E-04 | 1.15E-03 | Yes | Up | - |
| Q8TE69 | CXORF40A | 62/114(54.39) | 0.39 | 3.47E-08 | 2.64E-07 | Yes | Up | - |
| P31939 | ATIC | 61/114(53.51) | 0.48 | 7.14E-09 | 6.59E-08 | Yes | Up | - |
| Q6NVU6 | UFSP1 | 63/114(55.26) | 0.27 | 9.13E-04 | 2.28E-03 | Yes | Up | - |
| Q8NI32 | LYPD6B | 50/114(43.86) | 0.45 | 2.39E-02 | 4.21E-02 | Yes | Up | - |
| P18440 | NAT1 | 58/114(50.88) | 1.95 | 9.47E-19 | 3.45E-16 | Yes | Up | - |
| - | LOC401010 | 61/114(53.51) | 0.3 | 2.73E-04 | 7.72E-04 | Yes | Up | - |
| O95159 | ZFPL1 | 60/114(52.63) | 0.2 | 2.53E-04 | 7.23E-04 | Yes | Up | - |
| A8MQ03 | C9ORF169 | 70/114(61.4) | 1.28 | 7.20E-10 | 8.96E-09 | Yes | Up | - |
| Q9UKS6 | PACSIN3 | 64/114(56.14) | 0.49 | 3.19E-05 | 1.13E-04 | Yes | Up | - |
| Q99619 | SPSB2 | 65/114(57.02) | 0.37 | 4.46E-07 | 2.51E-06 | Yes | Up | - |
| Q502X0 | MORN2 | 64/114(56.14) | 0.39 | 2.91E-07 | 1.73E-06 | Yes | Up | - |
| - | C15ORF50 | 48/114(42.11) | 1.12 | 1.66E-05 | 6.31E-05 | Yes | Up | - |
| Q5T4B2 | CERCAM | 60/114(52.63) | 0.53 | 2.64E-07 | 1.58E-06 | Yes | Up | - |
| Q9P2E7 | PCDH10 | 51/114(44.74) | 0.79 | 3.40E-04 | 9.40E-04 | Yes | Up | - |
| Q99797 | MIPEP | 57/114(50.0) | 0.15 | 2.76E-02 | 4.80E-02 | Yes | Up | - |
| P50402 | EMD | 66/114(57.89) | 0.17 | 1.33E-03 | 3.19E-03 | Yes | Up | - |
| Q9NXL6 | SIDT1 | 58/114(50.88) | 0.47 | 3.48E-03 | 7.52E-03 | Yes | Up | - |
| Q6UXB1 | IGFL3 | 43/114(37.72) | 1.88 | 2.95E-07 | 1.74E-06 | Yes | Up | - |
| Q5FWF5 | ESCO1 | 55/114(48.25) | 0.21 | 8.52E-04 | 2.14E-03 | Yes | Up | - |
| - | LOC151534 | 52/114(45.61) | 0.26 | 1.17E-02 | 2.23E-02 | Yes | Up | - |
| Q9Y4Y9 | LSM5 | 65/114(57.02) | 0.15 | 7.94E-03 | 1.58E-02 | Yes | Up | - |
| Q969H9 | DIRC1 | 51/114(44.74) | 0.81 | 1.55E-04 | 4.66E-04 | Yes | Up | - |
| Q7Z407 | CSMD3 | 42/114(36.84) | 0.68 | 2.85E-02 | 4.92E-02 | Yes | Up | - |
| Q8N3F9 | GPR137C | 59/114(51.75) | 0.41 | 5.58E-04 | 1.47E-03 | Yes | Up | - |
| Q8TAK5 | GABPB2 | 55/114(48.25) | 0.23 | 6.20E-03 | 1.27E-02 | Yes | Up | - |
| Q9Y2S7 | POLDIP2 | 58/114(50.88) | 0.22 | 2.09E-04 | 6.09E-04 | Yes | Up | - |
| Q92615 | LARP4B | 53/114(46.49) | 0.18 | 4.31E-03 | 9.12E-03 | Yes | Up | - |
| Q8IZL9 | CDK20 | 53/114(46.49) | 0.19 | 1.47E-02 | 2.73E-02 | Yes | Up | - |
| Q10567 | AP1B1 | 60/114(52.63) | 0.2 | 5.49E-04 | 1.45E-03 | Yes | Up | - |
| - | AACSL | 42/114(36.84) | 0.88 | 8.02E-03 | 1.59E-02 | Yes | Up | - |
| Q9C0H9 | SRCIN1 | 59/114(51.75) | 0.45 | 7.40E-04 | 1.89E-03 | Yes | Up | - |
| Q9UBB6 | NCDN | 60/114(52.63) | 0.46 | 1.24E-06 | 6.20E-06 | Yes | Up | - |
| Q9P206 | KIAA1522 | 57/114(50.0) | 0.44 | 5.67E-05 | 1.89E-04 | Yes | Up | - |
| O75044 | SRGAP2 | 58/114(50.88) | 0.28 | 1.42E-04 | 4.31E-04 | Yes | Up | - |
| Q86X76 | NIT1 | 58/114(50.88) | 0.15 | 7.35E-03 | 1.48E-02 | Yes | Up | - |
| Q9BZL6 | PRKD2 | 68/114(59.65) | 0.18 | 1.17E-04 | 3.64E-04 | Yes | Up | - |
| Q8NHY5 | HUS1B | 63/114(55.26) | 0.64 | 7.31E-04 | 1.87E-03 | Yes | Up | - |
| Q9BX26 | SYCP2 | 60/114(52.63) | 0.45 | 6.84E-03 | 1.38E-02 | Yes | Up | - |
| P08637 | FCGR3A | 62/114(54.39) | 0.72 | 2.04E-08 | 1.68E-07 | Yes | Up | - |
| Q5W041 | ARMC3 | 66/114(57.89) | 1.53 | 9.39E-10 | 1.13E-08 | Yes | Up | - |
| O95473 | SYNGR4 | 64/114(56.14) | 0.86 | 6.94E-04 | 1.78E-03 | Yes | Up | - |
| Q969Z0 | TBRG4 | 60/114(52.63) | 0.24 | 5.27E-04 | 1.39E-03 | Yes | Up | - |
| Q99497 | PARK7 | 63/114(55.26) | 0.14 | 1.25E-02 | 2.37E-02 | Yes | Up | - |
| Q969E4 | TCEAL3 | 61/114(53.51) | 0.5 | 2.97E-06 | 1.35E-05 | Yes | Up | - |
| Q5U649 | C12ORF60 | 58/114(50.88) | 0.27 | 7.19E-03 | 1.45E-02 | Yes | Up | - |
| Q8TDM0 | BCAS4 | 62/114(54.39) | 0.27 | 2.33E-03 | 5.27E-03 | Yes | Up | - |
| Q9NZ42 | PSENEN | 63/114(55.26) | 0.6 | 1.31E-11 | 2.88E-10 | Yes | Up | - |
| Q14656 | TMEM187 | 64/114(56.14) | 0.19 | 1.43E-03 | 3.41E-03 | Yes | Up | - |
| Q3MHD2 | LSM12 | 60/114(52.63) | 0.27 | 2.42E-06 | 1.13E-05 | Yes | Up | - |
| Q3MIX3 | ADCK5 | 73/114(64.04) | 0.51 | 2.53E-06 | 1.17E-05 | Yes | Up | - |
| O75352 | MPDU1 | 57/114(50.0) | 0.21 | 2.27E-03 | 5.15E-03 | Yes | Up | - |
| P20042 | EIF2S2 | 62/114(54.39) | 0.16 | 7.95E-03 | 1.58E-02 | Yes | Up | - |
| - | C6ORF218 | 46/114(40.35) | 1.31 | 9.67E-06 | 3.90E-05 | Yes | Up | - |
| P20809 | IL11 | 61/114(53.51) | 0.78 | 7.83E-06 | 3.22E-05 | Yes | Up | - |
| Q68BL8 | OLFML2B | 57/114(50.0) | 0.8 | 3.35E-09 | 3.42E-08 | Yes | Up | - |
| Q92759 | GTF2H4 | 64/114(56.14) | 0.12 | 2.27E-02 | 4.02E-02 | Yes | Up | - |
| Q9UM11 | FZR1 | 73/114(64.04) | 0.27 | 6.22E-07 | 3.37E-06 | Yes | Up | - |
| P83111 | LACTB | 62/114(54.39) | 0.22 | 3.83E-03 | 8.20E-03 | Yes | Up | - |
| Q96CP6 | GRAMD1A | 62/114(54.39) | 0.56 | 4.93E-12 | 1.26E-10 | Yes | Up | - |
| Q9H0N0 | RAB6C | 55/114(48.25) | 0.32 | 6.41E-04 | 1.66E-03 | Yes | Up | - |
| P63261 | ACTG1 | 62/114(54.39) | 0.4 | 2.07E-09 | 2.23E-08 | Yes | Up | - |
| Q9H4I0 | RAD21L1 | 40/114(35.09) | 1.9 | 8.95E-07 | 4.66E-06 | Yes | Up | - |
| Q07065 | CKAP4 | 62/114(54.39) | 0.56 | 3.09E-10 | 4.30E-09 | Yes | Up | - |
| Q96NY8 | PVRL4 | 55/114(48.25) | 0.64 | 9.08E-04 | 2.27E-03 | Yes | Up | - |
| - | C15ORF21 | 56/114(49.12) | 0.22 | 4.99E-03 | 1.04E-02 | Yes | Up | - |
| Q9BZX2 | UCK2 | 54/114(47.37) | 0.41 | 4.06E-05 | 1.40E-04 | Yes | Up | - |
| Q15653 | NFKBIB | 67/114(58.77) | 0.37 | 4.61E-06 | 1.99E-05 | Yes | Up | - |
| Q8IW75 | SERPINA12 | 52/114(45.61) | 0.59 | 2.24E-02 | 3.97E-02 | Yes | Up | - |
| Q5BJF6 | ODF2 | 58/114(50.88) | 0.49 | 9.81E-10 | 1.17E-08 | Yes | Up | - |
| Q9UBL9 | P2RX2 | 56/114(49.12) | 0.65 | 1.47E-02 | 2.73E-02 | Yes | Up | - |
| Q96P70 | IPO9 | 59/114(51.75) | 0.28 | 1.17E-05 | 4.61E-05 | Yes | Up | - |
| Q96CN9 | GCC1 | 53/114(46.49) | 0.12 | 6.47E-03 | 1.32E-02 | Yes | Up | - |
| Q96SA4 | SERINC2 | 62/114(54.39) | 0.71 | 2.07E-06 | 9.80E-06 | Yes | Up | - |
| Q9Y3C6 | PPIL1 | 58/114(50.88) | 0.32 | 5.35E-05 | 1.79E-04 | Yes | Up | - |
| O75154 | RAB11FIP3 | 65/114(57.02) | 0.37 | 4.16E-06 | 1.82E-05 | Yes | Up | - |
| O15204 | ADAMDEC1 | 66/114(57.89) | 1.28 | 6.46E-08 | 4.57E-07 | Yes | Up | - |
| Q9UK45 | LSM7 | 64/114(56.14) | 0.51 | 2.02E-10 | 2.98E-09 | Yes | Up | - |
| O00217 | NDUFS8 | 61/114(53.51) | 0.42 | 5.87E-06 | 2.48E-05 | Yes | Up | - |
| Q15120 | PDK3 | 45/114(39.47) | 0.37 | 1.96E-04 | 5.73E-04 | Yes | Up | - |
| Q8TDW5 | SYTL5 | 55/114(48.25) | 1.02 | 1.69E-05 | 6.43E-05 | Yes | Up | - |
| - | NCRNA00176 | 58/114(50.88) | 1.08 | 2.79E-15 | 2.63E-13 | Yes | Up | - |
| Q13275 | SEMA3F | 63/114(55.26) | 0.63 | 1.54E-11 | 3.30E-10 | Yes | Up | - |
| Q53GD3 | SLC44A4 | 57/114(50.0) | 1.48 | 3.65E-11 | 6.95E-10 | Yes | Up | - |
| Q86YN1 | DOLPP1 | 62/114(54.39) | 0.39 | 1.01E-08 | 8.94E-08 | Yes | Up | - |
| Q8TBK6 | ZCCHC10 | 59/114(51.75) | 0.14 | 1.59E-02 | 2.93E-02 | Yes | Up | - |
| Q8IV53 | DENND1C | 57/114(50.0) | 0.5 | 3.66E-06 | 1.62E-05 | Yes | Up | - |
| P31274 | HOXC9 | 64/114(56.14) | 0.23 | 1.99E-02 | 3.58E-02 | Yes | Up | - |
| Q01094 | E2F1 | 63/114(55.26) | 1.18 | 3.47E-13 | 1.33E-11 | Yes | Up | - |
| Q9NYI0 | PSD3 | 55/114(48.25) | 0.58 | 8.13E-05 | 2.61E-04 | Yes | Up | - |
| Q9H2P0 | ADNP | 57/114(50.0) | 0.22 | 5.02E-04 | 1.33E-03 | Yes | Up | - |
| P12074 | COX6A1 | 63/114(55.26) | 0.48 | 1.45E-09 | 1.65E-08 | Yes | Up | - |
| Q00688 | FKBP3 | 59/114(51.75) | 0.29 | 1.65E-04 | 4.93E-04 | Yes | Up | - |
| Q7Z2Z2 | EFTUD1 | 54/114(47.37) | 0.15 | 1.86E-03 | 4.32E-03 | Yes | Up | - |
| Q9UJA5 | TRMT6 | 62/114(54.39) | 0.15 | 2.18E-02 | 3.89E-02 | Yes | Up | - |
| P59510 | ADAMTS20 | 34/114(29.82) | 1.4 | 2.51E-04 | 7.17E-04 | Yes | Up | - |
| Q9Y547 | HSPB11 | 62/114(54.39) | 0.31 | 5.05E-06 | 2.17E-05 | Yes | Up | - |
| P62310 | LSM3 | 65/114(57.02) | 0.18 | 2.43E-03 | 5.46E-03 | Yes | Up | - |
| P54886 | ALDH18A1 | 57/114(50.0) | 0.55 | 1.31E-09 | 1.51E-08 | Yes | Up | - |
| Q96LC9 | BMF | 59/114(51.75) | 0.42 | 7.05E-06 | 2.92E-05 | Yes | Up | - |
| Q8N5K1 | CISD2 | 60/114(52.63) | 0.35 | 2.99E-07 | 1.76E-06 | Yes | Up | - |
| Q9Y4P3 | TBL2 | 59/114(51.75) | 0.49 | 5.13E-10 | 6.73E-09 | Yes | Up | - |
| Q9UHL4 | DPP7 | 67/114(58.77) | 0.32 | 1.76E-04 | 5.21E-04 | Yes | Up | - |
| O95786 | DDX58 | 58/114(50.88) | 0.38 | 4.43E-05 | 1.51E-04 | Yes | Up | - |
| O43592 | XPOT | 55/114(48.25) | 0.21 | 7.78E-03 | 1.55E-02 | Yes | Up | - |
| Q8NFH4 | NUP37 | 67/114(58.77) | 0.25 | 4.01E-06 | 1.76E-05 | Yes | Up | - |
| Q9NPD8 | UBE2T | 60/114(52.63) | 1.61 | 1.39E-16 | 2.25E-14 | Yes | Up | - |
| Q6ZW49 | PAXIP1 | 63/114(55.26) | 0.35 | 1.07E-05 | 4.26E-05 | Yes | Up | - |
| Q9H8P0 | SRD5A3 | 55/114(48.25) | 0.55 | 1.02E-07 | 6.81E-07 | Yes | Up | - |
| Q5JSS6 | MEIG1 | 50/114(43.86) | 0.57 | 2.41E-03 | 5.43E-03 | Yes | Up | - |
| Q96EY5 | FAM125A | 67/114(58.77) | 0.29 | 1.23E-06 | 6.16E-06 | Yes | Up | - |
| Q96EG3 | ZNF837 | 70/114(61.4) | 0.31 | 2.26E-03 | 5.12E-03 | Yes | Up | - |
| P13497 | BMP1 | 58/114(50.88) | 0.3 | 3.47E-03 | 7.51E-03 | Yes | Up | - |
| Q9UM01 | SLC7A7 | 58/114(50.88) | 0.33 | 1.10E-03 | 2.70E-03 | Yes | Up | - |
| Q8N816 | TMEM99 | 57/114(50.0) | 0.28 | 4.25E-04 | 1.15E-03 | Yes | Up | - |
| Q9H299 | SH3BGRL3 | 64/114(56.14) | 0.35 | 1.98E-04 | 5.80E-04 | Yes | Up | - |
| P26640 | VARS | 63/114(55.26) | 0.23 | 1.35E-03 | 3.25E-03 | Yes | Up | - |
| Q8TDN6 | BRIX1 | 63/114(55.26) | 0.21 | 2.87E-03 | 6.33E-03 | Yes | Up | - |
| A6NJB7 | PRR19 | 59/114(51.75) | 1.07 | 3.96E-12 | 1.04E-10 | Yes | Up | - |
| Q04941 | PLP2 | 60/114(52.63) | 0.25 | 2.42E-03 | 5.45E-03 | Yes | Up | - |
| O60927 | PPP1R11 | 59/114(51.75) | 0.26 | 2.44E-06 | 1.14E-05 | Yes | Up | - |
| Q9UMS4 | PRPF19 | 62/114(54.39) | 0.31 | 3.99E-07 | 2.28E-06 | Yes | Up | - |
| Q92783 | STAM | 58/114(50.88) | 0.13 | 9.32E-03 | 1.82E-02 | Yes | Up | - |
| P14854 | COX6B1 | 62/114(54.39) | 0.27 | 1.67E-04 | 4.98E-04 | Yes | Up | - |
| P35913 | PDE6B | 61/114(53.51) | 0.35 | 1.65E-02 | 3.03E-02 | Yes | Up | - |
| Q5SZJ8 | BEND6 | 58/114(50.88) | 0.42 | 8.37E-04 | 2.11E-03 | Yes | Up | - |
| P78381 | SLC35A2 | 61/114(53.51) | 0.72 | 4.83E-12 | 1.24E-10 | Yes | Up | - |
| Q13508 | ART3 | 59/114(51.75) | 0.6 | 1.05E-02 | 2.02E-02 | Yes | Up | - |
| Q9UFD9 | RIMBP3 | 60/114(52.63) | 0.81 | 1.18E-12 | 3.71E-11 | Yes | Up | - |
| P10244 | MYBL2 | 60/114(52.63) | 1.82 | 2.56E-16 | 3.80E-14 | Yes | Up | - |
| Q5VV52 | ZNF691 | 61/114(53.51) | 0.13 | 2.30E-02 | 4.07E-02 | Yes | Up | - |
| Q9Y4F1 | FARP1 | 62/114(54.39) | 0.23 | 6.62E-03 | 1.34E-02 | Yes | Up | - |
| Q9BS34 | ZNF670 | 54/114(47.37) | 0.15 | 2.58E-02 | 4.51E-02 | Yes | Up | - |
| P35556 | FBN2 | 57/114(50.0) | 1.23 | 4.09E-09 | 4.06E-08 | Yes | Up | - |
| Q8NCU7 | C2CD4A | 66/114(57.89) | 0.92 | 1.31E-05 | 5.11E-05 | Yes | Up | - |
| P61244 | MAX | 62/114(54.39) | 0.13 | 1.94E-03 | 4.49E-03 | Yes | Up | - |
| Q9H0J9 | PARP12 | 66/114(57.89) | 0.41 | 2.50E-07 | 1.51E-06 | Yes | Up | - |
| O14669 | PRRG2 | 59/114(51.75) | 0.54 | 1.56E-04 | 4.69E-04 | Yes | Up | - |
| Q96PQ0 | SORCS2 | 62/114(54.39) | 0.51 | 2.91E-04 | 8.15E-04 | Yes | Up | - |
| P22415 | USF1 | 66/114(57.89) | 0.41 | 4.67E-11 | 8.49E-10 | Yes | Up | - |
| Q8NEZ5 | FBXO22 | 51/114(44.74) | 0.22 | 4.79E-04 | 1.28E-03 | Yes | Up | - |
| Q14242 | SELPLG | 64/114(56.14) | 0.36 | 7.68E-04 | 1.95E-03 | Yes | Up | - |
| Q9NV66 | TYW1 | 58/114(50.88) | 0.17 | 7.39E-05 | 2.40E-04 | Yes | Up | - |
| Q5VWZ2 | LYPLAL1 | 53/114(46.49) | 0.23 | 6.96E-04 | 1.79E-03 | Yes | Up | - |
| Q9NSD7 | RXFP3 | 42/114(36.84) | 1 | 8.43E-04 | 2.12E-03 | Yes | Up | - |
| Q5VSG8 | MANEAL | 57/114(50.0) | 0.67 | 6.60E-06 | 2.75E-05 | Yes | Up | - |
| P55145 | MANF | 63/114(55.26) | 0.56 | 1.19E-11 | 2.65E-10 | Yes | Up | - |
| Q8N609 | TRAM1L1 | 53/114(46.49) | 0.25 | 6.96E-03 | 1.40E-02 | Yes | Up | - |
| Q9UPT8 | ZC3H4 | 63/114(55.26) | 0.15 | 3.79E-05 | 1.32E-04 | Yes | Up | - |
| Q8TAP6 | CEP76 | 55/114(48.25) | 0.17 | 2.02E-03 | 4.64E-03 | Yes | Up | - |
| - | TOP1P2 | 65/114(57.02) | 0.35 | 7.64E-03 | 1.53E-02 | Yes | Up | - |
| Q9NVM1 | FAM176B | 69/114(60.53) | 0.66 | 2.95E-08 | 2.29E-07 | Yes | Up | - |
| Q96SB4 | SRPK1 | 54/114(47.37) | 0.34 | 3.29E-04 | 9.11E-04 | Yes | Up | - |
| Q7L8C5 | SYT13 | 58/114(50.88) | 1.72 | 2.40E-10 | 3.45E-09 | Yes | Up | - |
| P50851 | LRBA | 55/114(48.25) | 0.25 | 1.13E-02 | 2.16E-02 | Yes | Up | - |
| P43155 | CRAT | 57/114(50.0) | 0.43 | 3.27E-03 | 7.11E-03 | Yes | Up | - |
| Q8N114 | SHISA5 | 64/114(56.14) | 0.32 | 4.21E-06 | 1.84E-05 | Yes | Up | - |
| P00390 | GSR | 56/114(49.12) | 0.44 | 7.87E-06 | 3.24E-05 | Yes | Up | - |
| - | NME1-NME2 | 58/114(50.88) | 0.64 | 1.35E-07 | 8.75E-07 | Yes | Up | - |
| P35613 | BSG | 64/114(56.14) | 0.29 | 2.55E-04 | 7.26E-04 | Yes | Up | - |
| P11021 | HSPA5 | 59/114(51.75) | 0.41 | 2.67E-08 | 2.11E-07 | Yes | Up | - |
| Q8IWA0 | WDR75 | 59/114(51.75) | 0.17 | 6.01E-04 | 1.57E-03 | Yes | Up | - |
| - | PLAC2 | 68/114(59.65) | 1.68 | 4.17E-18 | 1.19E-15 | Yes | Up | - |
| Q96AA8 | JAKMIP2 | 59/114(51.75) | 0.66 | 3.64E-05 | 1.27E-04 | Yes | Up | - |
| Q9H1K6 | MESDC1 | 59/114(51.75) | 0.3 | 3.84E-05 | 1.34E-04 | Yes | Up | - |
| O00512 | BCL9 | 53/114(46.49) | 0.72 | 2.72E-12 | 7.52E-11 | Yes | Up | - |
| Q03111 | MLLT1 | 64/114(56.14) | 0.13 | 2.71E-03 | 6.02E-03 | Yes | Up | - |
| Q8IUW3 | SPATA2L | 70/114(61.4) | 0.35 | 2.73E-04 | 7.71E-04 | Yes | Up | - |
| Q9NW75 | GPATCH2 | 57/114(50.0) | 0.58 | 5.34E-09 | 5.12E-08 | Yes | Up | - |
| P19525 | EIF2AK2 | 57/114(50.0) | 0.34 | 2.21E-04 | 6.38E-04 | Yes | Up | - |
| Q9BTD3 | TMEM121 | 66/114(57.89) | 0.73 | 3.62E-08 | 2.74E-07 | Yes | Up | - |
| Q9H0U6 | MRPL18 | 58/114(50.88) | 0.19 | 7.16E-04 | 1.83E-03 | Yes | Up | - |
| O76096 | CST7 | 62/114(54.39) | 0.38 | 5.08E-03 | 1.06E-02 | Yes | Up | - |
| O95453 | PARN | 63/114(55.26) | 0.42 | 1.61E-10 | 2.46E-09 | Yes | Up | - |
| Q6ICH7 | ASPHD2 | 66/114(57.89) | 0.54 | 2.49E-07 | 1.51E-06 | Yes | Up | - |
| Q9BZM5 | ULBP2 | 60/114(52.63) | 0.66 | 2.05E-05 | 7.62E-05 | Yes | Up | - |
| P08574 | CYC1 | 65/114(57.02) | 0.33 | 2.97E-04 | 8.31E-04 | Yes | Up | - |
| Q8TCQ1 | Mar-01 | 59/114(51.75) | 0.33 | 1.39E-03 | 3.33E-03 | Yes | Up | - |
| Q5VTQ0 | TTC39B | 57/114(50.0) | 0.31 | 5.01E-03 | 1.05E-02 | Yes | Up | - |
| Q8NEG4 | FAM83F | 55/114(48.25) | 0.36 | 3.11E-03 | 6.80E-03 | Yes | Up | - |
| P82930 | MRPS34 | 65/114(57.02) | 0.73 | 8.94E-14 | 4.38E-12 | Yes | Up | - |
| Q6IA86 | ELP2 | 62/114(54.39) | 0.28 | 6.01E-03 | 1.23E-02 | Yes | Up | - |
| O95528 | SLC2A10 | 62/114(54.39) | 0.66 | 4.82E-08 | 3.52E-07 | Yes | Up | - |
| P49589 | CARS | 56/114(49.12) | 0.18 | 6.76E-03 | 1.37E-02 | Yes | Up | - |
| Q15084 | PDIA6 | 55/114(48.25) | 0.25 | 4.54E-04 | 1.22E-03 | Yes | Up | - |
| Q14146 | URB2 | 55/114(48.25) | 0.26 | 1.46E-03 | 3.47E-03 | Yes | Up | - |
| P57737 | CORO7 | 65/114(57.02) | 0.48 | 7.15E-12 | 1.73E-10 | Yes | Up | - |
| Q5VZI3 | C9ORF91 | 50/114(43.86) | 0.28 | 5.02E-04 | 1.33E-03 | Yes | Up | - |
| Q4ZHG4 | FNDC1 | 59/114(51.75) | 1.06 | 7.87E-08 | 5.45E-07 | Yes | Up | - |
| Q96F82 | ORC1L | 56/114(49.12) | 1.09 | 8.33E-10 | 1.02E-08 | Yes | Up | - |
| - | LOC149134 | 60/114(52.63) | 0.29 | 2.39E-02 | 4.22E-02 | Yes | Up | - |
| P52798 | EFNA4 | 63/114(55.26) | 0.78 | 1.81E-10 | 2.72E-09 | Yes | Up | - |
| Q9Y5L4 | TIMM13 | 67/114(58.77) | 0.36 | 3.03E-05 | 1.08E-04 | Yes | Up | - |
| Q92820 | GGH | 55/114(48.25) | 0.35 | 1.08E-02 | 2.07E-02 | Yes | Up | - |
| Q9Y365 | STARD10 | 62/114(54.39) | 1.15 | 6.33E-15 | 5.10E-13 | Yes | Up | - |
| Q96B01 | RAD51AP1 | 60/114(52.63) | 1.05 | 2.71E-10 | 3.83E-09 | Yes | Up | - |
| Q16623 | STX1A | 59/114(51.75) | 0.61 | 1.41E-07 | 9.10E-07 | Yes | Up | - |
| P46087 | NOP2 | 59/114(51.75) | 0.4 | 2.30E-07 | 1.40E-06 | Yes | Up | - |
| O75185 | ATP2C2 | 62/114(54.39) | 0.79 | 2.02E-05 | 7.54E-05 | Yes | Up | - |
| Q8WWB3 | DYDC1 | 38/114(33.33) | 0.84 | 1.37E-02 | 2.57E-02 | Yes | Up | - |
| O15156 | ZBTB7B | 51/114(44.74) | 0.35 | 1.78E-06 | 8.59E-06 | Yes | Up | - |
| P61978 | HNRNPK | 57/114(50.0) | 0.13 | 2.61E-04 | 7.43E-04 | Yes | Up | - |
| Q8IW41 | MAPKAPK5 | 56/114(49.12) | 0.16 | 6.97E-04 | 1.79E-03 | Yes | Up | - |
| Q9NXN4 | GDAP2 | 48/114(42.11) | 0.16 | 9.53E-03 | 1.86E-02 | Yes | Up | - |
| Q9Y2Y1 | POLR3K | 60/114(52.63) | 0.85 | 6.23E-16 | 7.57E-14 | Yes | Up | - |
| P45452 | MMP13 | 63/114(55.26) | 1.78 | 2.84E-08 | 2.22E-07 | Yes | Up | - |
| P57789 | KCNK10 | 58/114(50.88) | 0.62 | 2.22E-03 | 5.05E-03 | Yes | Up | - |
| Q6DD87 | ZNF787 | 66/114(57.89) | 0.33 | 3.93E-05 | 1.36E-04 | Yes | Up | - |
| Q8TE99 | ACPL2 | 50/114(43.86) | 0.22 | 3.06E-03 | 6.69E-03 | Yes | Up | - |
| Q9BV81 | TMEM93 | 66/114(57.89) | 0.16 | 1.99E-02 | 3.58E-02 | Yes | Up | - |
| Q9NRD1 | FBXO6 | 57/114(50.0) | 0.51 | 2.27E-08 | 1.84E-07 | Yes | Up | - |
| Q15696 | ZRSR2 | 65/114(57.02) | 0.13 | 1.01E-02 | 1.95E-02 | Yes | Up | - |
| P55000 | SLURP1 | 44/114(38.6) | 1.01 | 1.12E-04 | 3.50E-04 | Yes | Up | - |
| P41146 | OPRL1 | 57/114(50.0) | 0.27 | 2.12E-03 | 4.84E-03 | Yes | Up | - |
| Q8WZ19 | KCTD13 | 62/114(54.39) | 0.52 | 3.00E-09 | 3.10E-08 | Yes | Up | - |
| O75575 | CRCP | 58/114(50.88) | 0.22 | 4.17E-05 | 1.44E-04 | Yes | Up | - |
| A9UHW6 | MIF4GD | 56/114(49.12) | 0.16 | 1.38E-02 | 2.58E-02 | Yes | Up | - |
| Q99426 | TBCB | 61/114(53.51) | 0.46 | 2.29E-08 | 1.85E-07 | Yes | Up | - |
| Q14353 | GAMT | 63/114(55.26) | 0.53 | 1.00E-05 | 4.03E-05 | Yes | Up | - |
| Q92979 | EMG1 | 56/114(49.12) | 0.23 | 2.33E-05 | 8.55E-05 | Yes | Up | - |
| Q8TBY9 | WDR66 | 59/114(51.75) | 0.48 | 7.68E-04 | 1.95E-03 | Yes | Up | - |
| Q8N9H9 | C1ORF127 | 58/114(50.88) | 0.55 | 9.21E-04 | 2.29E-03 | Yes | Up | - |
| Q9BZS1 | FOXP3 | 62/114(54.39) | 1.37 | 2.54E-15 | 2.48E-13 | Yes | Up | - |
| O43172 | PRPF4 | 56/114(49.12) | 0.27 | 2.76E-06 | 1.26E-05 | Yes | Up | - |
| Q9Y6X1 | SERP1 | 60/114(52.63) | 0.25 | 3.44E-04 | 9.49E-04 | Yes | Up | - |
| - | NCRNA00152 | 63/114(55.26) | 0.39 | 4.68E-04 | 1.25E-03 | Yes | Up | - |
| Q99417 | MYCBP | 63/114(55.26) | 0.34 | 1.36E-05 | 5.29E-05 | Yes | Up | - |
| Q96LR7 | C2ORF50 | 66/114(57.89) | 0.79 | 3.51E-06 | 1.57E-05 | Yes | Up | - |
| Q5BJF2 | TMEM97 | 58/114(50.88) | 0.67 | 2.15E-07 | 1.32E-06 | Yes | Up | - |
| Q9BR77 | CCDC77 | 60/114(52.63) | 0.16 | 1.87E-02 | 3.39E-02 | Yes | Up | - |
| Q8N1P7 | AIM1L | 63/114(55.26) | 2.02 | 1.85E-17 | 4.34E-15 | Yes | Up | - |
| P0C5K7 | CT62 | 55/114(48.25) | 0.61 | 7.45E-03 | 1.49E-02 | Yes | Up | - |
| Q9BYK8 | PRIC285 | 58/114(50.88) | 0.5 | 6.07E-07 | 3.30E-06 | Yes | Up | - |
| O15240 | VGF | 54/114(47.37) | 1.07 | 2.51E-05 | 9.11E-05 | Yes | Up | - |
| B7Z6K7 | ZNF814 | 60/114(52.63) | 0.31 | 6.20E-04 | 1.61E-03 | Yes | Up | - |
| P14784 | IL2RB | 51/114(44.74) | 0.29 | 1.67E-02 | 3.06E-02 | Yes | Up | - |
| Q9BVN2 | RUSC1 | 62/114(54.39) | 0.76 | 4.15E-13 | 1.55E-11 | Yes | Up | - |
| P80217 | IFI35 | 69/114(60.53) | 0.52 | 2.62E-07 | 1.57E-06 | Yes | Up | - |
| Q96NY7 | CLIC6 | 62/114(54.39) | 0.62 | 2.74E-03 | 6.07E-03 | Yes | Up | - |
| P53041 | PPP5C | 65/114(57.02) | 0.26 | 6.30E-06 | 2.64E-05 | Yes | Up | - |
| Q9UKD1 | GMEB2 | 68/114(59.65) | 0.18 | 8.13E-04 | 2.06E-03 | Yes | Up | - |
| Q6L9T8 | FAM72D | 61/114(53.51) | 1.3 | 3.08E-12 | 8.45E-11 | Yes | Up | - |
| - | LOC643677 | 62/114(54.39) | 0.36 | 5.93E-03 | 1.22E-02 | Yes | Up | - |
| Q0P6H9 | TMEM62 | 57/114(50.0) | 0.32 | 1.01E-04 | 3.18E-04 | Yes | Up | - |
| Q9Y5M8 | SRPRB | 61/114(53.51) | 0.35 | 8.84E-06 | 3.60E-05 | Yes | Up | - |
| Q96GR4 | ZDHHC12 | 65/114(57.02) | 0.6 | 2.49E-11 | 5.06E-10 | Yes | Up | - |
| Q15828 | CST6 | 63/114(55.26) | 1.12 | 8.42E-08 | 5.78E-07 | Yes | Up | - |
| P36507 | MAP2K2 | 73/114(64.04) | 0.19 | 4.45E-03 | 9.41E-03 | Yes | Up | - |
| Q9NX18 | SDHAF2 | 61/114(53.51) | 0.16 | 5.45E-04 | 1.44E-03 | Yes | Up | - |
| P00505 | GOT2 | 57/114(50.0) | 0.33 | 2.18E-04 | 6.32E-04 | Yes | Up | - |
| Q9H9B1 | EHMT1 | 63/114(55.26) | 0.15 | 2.62E-03 | 5.84E-03 | Yes | Up | - |
| Q04837 | SSBP1 | 68/114(59.65) | 0.22 | 5.90E-04 | 1.54E-03 | Yes | Up | - |
| Q15072 | ZNF146 | 52/114(45.61) | 0.16 | 1.95E-02 | 3.51E-02 | Yes | Up | - |
| Q9Y5Z9 | UBIAD1 | 50/114(43.86) | 0.11 | 1.14E-02 | 2.17E-02 | Yes | Up | - |
| O60232 | SSSCA1 | 66/114(57.89) | 0.27 | 4.68E-05 | 1.59E-04 | Yes | Up | - |
| Q16891 | IMMT | 58/114(50.88) | 0.13 | 1.77E-02 | 3.23E-02 | Yes | Up | - |
| Q92569 | PIK3R3 | 60/114(52.63) | 0.33 | 4.14E-04 | 1.12E-03 | Yes | Up | - |
| Q02833 | RASSF7 | 59/114(51.75) | 0.86 | 7.64E-11 | 1.29E-09 | Yes | Up | - |
| P14652 | HOXB2 | 54/114(47.37) | 0.72 | 6.76E-07 | 3.63E-06 | Yes | Up | - |
| Q7Z4N8 | P4HA3 | 61/114(53.51) | 1.9 | 8.42E-22 | 8.56E-19 | Yes | Up | - |
| P42224 | STAT1 | 57/114(50.0) | 0.49 | 1.40E-05 | 5.43E-05 | Yes | Up | - |
| Q01726 | MC1R | 58/114(50.88) | 0.8 | 8.46E-12 | 2.00E-10 | Yes | Up | - |
| Q7Z4H9 | C7ORF70 | 55/114(48.25) | 0.23 | 9.58E-05 | 3.03E-04 | Yes | Up | - |
| - | RPPH1 | 59/114(51.75) | 0.44 | 2.28E-03 | 5.17E-03 | Yes | Up | - |
| P51153 | RAB13 | 64/114(56.14) | 0.37 | 1.35E-07 | 8.74E-07 | Yes | Up | - |
| Q9NUG6 | PDRG1 | 63/114(55.26) | 0.5 | 4.42E-08 | 3.27E-07 | Yes | Up | - |
| O15492 | RGS16 | 62/114(54.39) | 0.64 | 2.01E-07 | 1.24E-06 | Yes | Up | - |
| - | PRSS30P | 53/114(46.49) | 0.34 | 2.67E-02 | 4.64E-02 | Yes | Up | - |
| Q8IU99 | CALHM1 | 58/114(50.88) | 0.92 | 9.04E-06 | 3.67E-05 | Yes | Up | - |
| Q5R3F8 | ELFN2 | 58/114(50.88) | 0.93 | 1.78E-06 | 8.56E-06 | Yes | Up | - |
| Q9NQS5 | GPR84 | 63/114(55.26) | 1.01 | 2.16E-09 | 2.32E-08 | Yes | Up | - |
| P21127 | CDK11B | 59/114(51.75) | 0.14 | 1.81E-02 | 3.29E-02 | Yes | Up | - |
| Q6F5E8 | RLTPR | 62/114(54.39) | 0.65 | 1.29E-04 | 3.95E-04 | Yes | Up | - |
| Q8WX94 | NLRP7 | 57/114(50.0) | 0.53 | 4.00E-03 | 8.52E-03 | Yes | Up | - |
| Q86YD1 | PTOV1 | 66/114(57.89) | 0.32 | 1.74E-06 | 8.39E-06 | Yes | Up | - |
| Q8IXB1 | DNAJC10 | 53/114(46.49) | 0.18 | 1.05E-02 | 2.03E-02 | Yes | Up | - |
| Q2NKX8 | ERCC6L | 60/114(52.63) | 1.34 | 6.25E-12 | 1.55E-10 | Yes | Up | - |
| - | HAR1B | 57/114(50.0) | 0.93 | 2.26E-05 | 8.30E-05 | Yes | Up | - |
| Q9Y5K2 | KLK4 | 66/114(57.89) | 2.06 | 8.89E-21 | 5.48E-18 | Yes | Up | - |
| P35227 | PCGF2 | 61/114(53.51) | 0.49 | 1.34E-09 | 1.54E-08 | Yes | Up | - |
| Q8IVP5 | FUNDC1 | 56/114(49.12) | 0.42 | 4.62E-08 | 3.40E-07 | Yes | Up | - |
| Q969K7 | TMEM54 | 60/114(52.63) | 0.62 | 3.70E-10 | 5.05E-09 | Yes | Up | - |
| Q9BRQ5 | ORAI3 | 60/114(52.63) | 0.19 | 9.73E-03 | 1.89E-02 | Yes | Up | - |
| Q9UKJ8 | ADAM21 | 58/114(50.88) | 0.35 | 9.47E-03 | 1.85E-02 | Yes | Up | - |
| P78411 | IRX5 | 60/114(52.63) | 0.6 | 8.71E-06 | 3.55E-05 | Yes | Up | - |
| Q9UPR3 | SMG5 | 57/114(50.0) | 0.24 | 1.19E-03 | 2.88E-03 | Yes | Up | - |
| Q9Y666 | SLC12A7 | 60/114(52.63) | 0.31 | 3.42E-05 | 1.20E-04 | Yes | Up | - |
| Q8N8U2 | CDYL2 | 60/114(52.63) | 0.33 | 1.22E-02 | 2.31E-02 | Yes | Up | - |
| Q9ULV0 | MYO5B | 56/114(49.12) | 0.45 | 2.33E-03 | 5.28E-03 | Yes | Up | - |
| Q96A35 | MRPL24 | 64/114(56.14) | 0.52 | 6.67E-10 | 8.40E-09 | Yes | Up | - |
| P08123 | COL1A2 | 62/114(54.39) | 1.11 | 4.77E-11 | 8.64E-10 | Yes | Up | - |
| E7ERK7 | WDR67 | 61/114(53.51) | 0.54 | 3.93E-07 | 2.25E-06 | Yes | Up | - |
| O75419 | CDC45 | 63/114(55.26) | 1.45 | 1.05E-10 | 1.71E-09 | Yes | Up | - |
| Q9H5H4 | ZNF768 | 66/114(57.89) | 0.34 | 4.73E-07 | 2.65E-06 | Yes | Up | - |
| Q9UDW1 | UQCR10 | 59/114(51.75) | 0.23 | 3.13E-03 | 6.84E-03 | Yes | Up | - |
| Q9P2B2 | PTGFRN | 61/114(53.51) | 0.36 | 3.09E-05 | 1.10E-04 | Yes | Up | - |
| Q86XJ1 | GAS2L3 | 62/114(54.39) | 0.57 | 8.68E-05 | 2.77E-04 | Yes | Up | - |
| P33991 | MCM4 | 58/114(50.88) | 0.85 | 7.97E-10 | 9.79E-09 | Yes | Up | - |
| B4DS86 | NEURL | 63/114(55.26) | 1.68 | 7.91E-16 | 9.15E-14 | Yes | Up | - |
| O43315 | AQP9 | 57/114(50.0) | 0.64 | 1.58E-03 | 3.73E-03 | Yes | Up | - |
| Q676U5 | ATG16L1 | 59/114(51.75) | 0.32 | 1.79E-06 | 8.62E-06 | Yes | Up | - |
| P63172 | DYNLT1 | 61/114(53.51) | 0.39 | 8.18E-07 | 4.30E-06 | Yes | Up | - |
| Q5SRH9 | TTC39A | 59/114(51.75) | 0.9 | 1.84E-07 | 1.15E-06 | Yes | Up | - |
| O14764 | GABRD | 65/114(57.02) | 2.04 | 2.96E-21 | 2.49E-18 | Yes | Up | - |
| Q8NA77 | TEX19 | 52/114(45.61) | 2.57 | 2.76E-15 | 2.63E-13 | Yes | Up | - |
| P36888 | FLT3 | 54/114(47.37) | 1.01 | 5.83E-08 | 4.16E-07 | Yes | Up | - |
| Q14764 | MVP | 66/114(57.89) | 0.4 | 1.27E-05 | 4.99E-05 | Yes | Up | - |
| P20132 | SDS | 62/114(54.39) | 1.58 | 9.88E-16 | 1.09E-13 | Yes | Up | - |
| Q9UP52 | TFR2 | 63/114(55.26) | 1.63 | 1.12E-14 | 7.99E-13 | Yes | Up | - |
| P52907 | CAPZA1 | 55/114(48.25) | 0.37 | 3.83E-07 | 2.20E-06 | Yes | Up | - |
| Q6P582 | FAM128A | 68/114(59.65) | 0.7 | 5.42E-10 | 7.05E-09 | Yes | Up | - |
| P48431 | SOX2 | 53/114(46.49) | 1.33 | 8.12E-08 | 5.60E-07 | Yes | Up | - |
| P13051 | UNG | 56/114(49.12) | 0.2 | 2.58E-03 | 5.76E-03 | Yes | Up | - |
| O95258 | SLC25A14 | 60/114(52.63) | 0.31 | 4.88E-08 | 3.56E-07 | Yes | Up | - |
| Q9Y336 | SIGLEC9 | 67/114(58.77) | 0.52 | 7.02E-06 | 2.91E-05 | Yes | Up | - |
| Q9NZ71 | RTEL1 | 62/114(54.39) | 0.18 | 1.21E-02 | 2.29E-02 | Yes | Up | - |
| O95180 | CACNA1H | 57/114(50.0) | 0.83 | 4.04E-06 | 1.77E-05 | Yes | Up | - |
| Q8WXF8 | DEDD2 | 58/114(50.88) | 0.27 | 3.57E-06 | 1.59E-05 | Yes | Up | - |
| Q15058 | KIF14 | 56/114(49.12) | 1.53 | 3.68E-11 | 7.00E-10 | Yes | Up | - |
| O75995 | SASH3 | 58/114(50.88) | 0.26 | 1.22E-02 | 2.31E-02 | Yes | Up | - |
| D3DTV9 | C17ORF93 | 30/114(26.32) | 1.05 | 2.05E-02 | 3.68E-02 | Yes | Up | - |
| Q9Y3P4 | RHBDD3 | 67/114(58.77) | 0.5 | 5.51E-08 | 3.96E-07 | Yes | Up | - |
| Q9P209 | CEP72 | 61/114(53.51) | 0.32 | 7.35E-04 | 1.88E-03 | Yes | Up | - |
| P31275 | HOXC12 | 43/114(37.72) | 1.13 | 4.60E-04 | 1.23E-03 | Yes | Up | - |
| Q9P0T4 | ZNF581 | 70/114(61.4) | 0.24 | 1.62E-03 | 3.82E-03 | Yes | Up | - |
| Q71SY5 | MED25 | 67/114(58.77) | 0.42 | 6.45E-08 | 4.56E-07 | Yes | Up | - |
| Q9NQQ7 | SLC35C2 | 66/114(57.89) | 0.22 | 4.05E-05 | 1.40E-04 | Yes | Up | - |
| Q13426 | XRCC4 | 62/114(54.39) | 0.2 | 7.20E-04 | 1.84E-03 | Yes | Up | - |
| P52744 | ZNF138 | 58/114(50.88) | 0.29 | 4.11E-04 | 1.11E-03 | Yes | Up | - |
| Q93033 | CD101 | 58/114(50.88) | 0.29 | 3.50E-03 | 7.55E-03 | Yes | Up | - |
| - | LOC100128977 | 55/114(48.25) | 0.64 | 2.01E-02 | 3.60E-02 | Yes | Up | - |
| Q9Y257 | KCNK6 | 64/114(56.14) | 0.79 | 2.20E-10 | 3.20E-09 | Yes | Up | - |
| Q16626 | MEA1 | 61/114(53.51) | 0.33 | 1.55E-07 | 9.88E-07 | Yes | Up | - |
| Q9NRJ4 | TULP4 | 55/114(48.25) | 0.23 | 7.59E-04 | 1.93E-03 | Yes | Up | - |
| P20800 | EDN2 | 63/114(55.26) | 1.03 | 1.19E-06 | 5.99E-06 | Yes | Up | - |
| - | LOC100130148 | 56/114(49.12) | 0.77 | 1.31E-03 | 3.15E-03 | Yes | Up | - |
| Q5MNZ9 | WIPI1 | 56/114(49.12) | 0.17 | 1.90E-02 | 3.43E-02 | Yes | Up | - |
| O60481 | ZIC3 | 39/114(34.21) | 1.08 | 1.36E-03 | 3.25E-03 | Yes | Up | - |
| Q9NV06 | DCAF13 | 60/114(52.63) | 0.54 | 1.98E-07 | 1.22E-06 | Yes | Up | - |
| Q96DV4 | MRPL38 | 55/114(48.25) | 0.21 | 3.57E-03 | 7.69E-03 | Yes | Up | - |
| Q9Y2G8 | DNAJC16 | 60/114(52.63) | 0.12 | 2.52E-02 | 4.42E-02 | Yes | Up | - |
| P17480 | UBTF | 62/114(54.39) | 0.22 | 1.33E-06 | 6.61E-06 | Yes | Up | - |
| O14957 | UQCR11 | 56/114(49.12) | 0.17 | 1.79E-02 | 3.26E-02 | Yes | Up | - |
| Q96JC9 | EAF1 | 63/114(55.26) | 0.37 | 1.26E-06 | 6.31E-06 | Yes | Up | - |
| Q96FE5 | LINGO1 | 57/114(50.0) | 0.88 | 8.18E-08 | 5.63E-07 | Yes | Up | - |
| Q13308 | PTK7 | 55/114(48.25) | 0.6 | 1.86E-05 | 7.00E-05 | Yes | Up | - |
| O75487 | GPC4 | 54/114(47.37) | 0.62 | 6.80E-07 | 3.65E-06 | Yes | Up | - |
| O00566 | MPHOSPH10 | 64/114(56.14) | 0.15 | 1.61E-02 | 2.96E-02 | Yes | Up | - |
| Q96JA4 | MS4A14 | 55/114(48.25) | 0.49 | 1.02E-03 | 2.52E-03 | Yes | Up | - |
| Q14376 | GALE | 62/114(54.39) | 0.94 | 1.29E-14 | 9.03E-13 | Yes | Up | - |
| Q969W3 | FAM104A | 51/114(44.74) | 0.22 | 2.46E-05 | 8.97E-05 | Yes | Up | - |
| O94772 | LY6H | 64/114(56.14) | 1.43 | 3.34E-11 | 6.47E-10 | Yes | Up | - |
| Q5U5X8 | C12ORF34 | 58/114(50.88) | 0.67 | 8.28E-08 | 5.69E-07 | Yes | Up | - |
| Q8N7J2 | FAM123A | 28/114(24.56) | 1.58 | 2.79E-03 | 6.17E-03 | Yes | Up | - |
| Q8IXX5 | TMEM183A | 59/114(51.75) | 0.37 | 5.17E-07 | 2.87E-06 | Yes | Up | - |
| Q9C002 | C15ORF48 | 56/114(49.12) | 1.4 | 8.16E-13 | 2.73E-11 | Yes | Up | - |
| Q9H295 | TM7SF4 | 56/114(49.12) | 0.84 | 5.18E-05 | 1.74E-04 | Yes | Up | - |
| Q7Z6K4 | NRARP | 58/114(50.88) | 0.33 | 5.02E-04 | 1.33E-03 | Yes | Up | - |
| Q9GZP8 | C19ORF33 | 61/114(53.51) | 1.35 | 1.03E-11 | 2.38E-10 | Yes | Up | - |
| Q96AZ6 | ISG20 | 67/114(58.77) | 0.49 | 7.84E-06 | 3.22E-05 | Yes | Up | - |
| O75607 | NPM3 | 59/114(51.75) | 0.24 | 1.13E-02 | 2.16E-02 | Yes | Up | - |
| P33765 | ADORA3 | 57/114(50.0) | 0.45 | 1.43E-04 | 4.34E-04 | Yes | Up | - |
| Q9H944 | MED20 | 60/114(52.63) | 0.17 | 2.54E-03 | 5.69E-03 | Yes | Up | - |
| O43143 | DHX15 | 58/114(50.88) | 0.14 | 2.25E-02 | 3.99E-02 | Yes | Up | - |
| Q96M43 | NBPF4 | 61/114(53.51) | 1.48 | 1.55E-09 | 1.75E-08 | Yes | Up | - |
| Q9NRW7 | VPS45 | 50/114(43.86) | 0.21 | 2.69E-04 | 7.62E-04 | Yes | Up | - |
| Q13155 | AIMP2 | 65/114(57.02) | 0.34 | 8.05E-06 | 3.30E-05 | Yes | Up | - |
| Q8N165 | PDIK1L | 56/114(49.12) | 0.35 | 4.55E-05 | 1.55E-04 | Yes | Up | - |
| Q14573 | ITPR3 | 56/114(49.12) | 0.37 | 2.73E-04 | 7.73E-04 | Yes | Up | - |
| O60894 | RAMP1 | 70/114(61.4) | 1.34 | 2.14E-13 | 8.84E-12 | Yes | Up | - |
| P10588 | NR2F6 | 65/114(57.02) | 0.61 | 1.90E-12 | 5.59E-11 | Yes | Up | - |
| Q6NXS1 | PPP1R2P3 | 58/114(50.88) | 0.24 | 6.62E-04 | 1.71E-03 | Yes | Up | - |
| Q9HCC6 | HES4 | 68/114(59.65) | 0.65 | 6.91E-07 | 3.70E-06 | Yes | Up | - |
| Q9P1W3 | TMEM63C | 57/114(50.0) | 0.99 | 4.29E-06 | 1.87E-05 | Yes | Up | - |
| Q9BW92 | TARS2 | 59/114(51.75) | 0.55 | 2.19E-10 | 3.18E-09 | Yes | Up | - |
| Q9NRI6 | PYY2 | 67/114(58.77) | 0.53 | 1.96E-03 | 4.52E-03 | Yes | Up | - |
| Q16625 | OCLN | 59/114(51.75) | 0.56 | 1.83E-04 | 5.39E-04 | Yes | Up | - |
| Q9BTD8 | RBM42 | 67/114(58.77) | 0.27 | 3.06E-05 | 1.09E-04 | Yes | Up | - |
| Q07325 | CXCL9 | 61/114(53.51) | 1.41 | 8.95E-12 | 2.10E-10 | Yes | Up | - |
| P20701 | ITGAL | 59/114(51.75) | 0.4 | 6.78E-04 | 1.75E-03 | Yes | Up | - |
| P01588 | EPO | 59/114(51.75) | 1.63 | 2.20E-12 | 6.28E-11 | Yes | Up | - |
| Q9NZV6 | SEPX1 | 63/114(55.26) | 0.5 | 5.93E-08 | 4.23E-07 | Yes | Up | - |
| O75794 | CDC123 | 62/114(54.39) | 0.18 | 4.37E-03 | 9.25E-03 | Yes | Up | - |
| Q9ULW5 | RAB26 | 60/114(52.63) | 1.69 | 2.66E-17 | 5.78E-15 | Yes | Up | - |
| Q9UNS1 | TIMELESS | 56/114(49.12) | 0.79 | 2.17E-11 | 4.47E-10 | Yes | Up | - |
| P22792 | CPN2 | 54/114(47.37) | 0.59 | 8.70E-03 | 1.71E-02 | Yes | Up | - |
| Q29980 | MICB | 66/114(57.89) | 0.7 | 2.86E-09 | 2.97E-08 | Yes | Up | - |
| I3L3T0 | TIMM16 | 66/114(57.89) | 0.51 | 4.78E-09 | 4.67E-08 | Yes | Up | - |
| P60174 | TPI1 | 65/114(57.02) | 0.39 | 4.19E-06 | 1.83E-05 | Yes | Up | - |
| P52824 | DGKQ | 68/114(59.65) | 0.34 | 3.81E-06 | 1.68E-05 | Yes | Up | - |
| Q96HA7 | NFKBIL2 | 65/114(57.02) | 1.06 | 8.46E-13 | 2.80E-11 | Yes | Up | - |
| O43248 | HOXC11 | 67/114(58.77) | 1.31 | 1.95E-07 | 1.21E-06 | Yes | Up | - |
| Q9NSI8 | SAMSN1 | 59/114(51.75) | 0.44 | 1.21E-04 | 3.74E-04 | Yes | Up | - |
| P55199 | ELL | 73/114(64.04) | 0.13 | 4.92E-03 | 1.03E-02 | Yes | Up | - |
| B5MC22 | MTP18 | 58/114(50.88) | 0.57 | 5.89E-10 | 7.54E-09 | Yes | Up | - |
| Q8NG06 | TRIM58 | 55/114(48.25) | 0.9 | 7.50E-07 | 3.98E-06 | Yes | Up | - |
| P52961 | ART1 | 37/114(32.46) | 0.83 | 1.71E-02 | 3.12E-02 | Yes | Up | - |
| Q6NUQ4 | TMEM214 | 57/114(50.0) | 0.14 | 1.42E-02 | 2.65E-02 | Yes | Up | - |
| A6NFX1 | MFSD2B | 60/114(52.63) | 0.69 | 1.58E-07 | 1.00E-06 | Yes | Up | - |
| Q96IJ6 | GMPPA | 64/114(56.14) | 0.45 | 1.64E-10 | 2.50E-09 | Yes | Up | - |
| Q14CX5 | TMEM180 | 59/114(51.75) | 0.29 | 9.54E-04 | 2.37E-03 | Yes | Up | - |
| Q96A04 | C1ORF182 | 56/114(49.12) | 0.94 | 1.42E-07 | 9.13E-07 | Yes | Up | - |
| Q9Y2H9 | MAST1 | 59/114(51.75) | 0.6 | 8.83E-04 | 2.21E-03 | Yes | Up | - |
| P56715 | RP1 | 59/114(51.75) | 0.65 | 1.66E-03 | 3.90E-03 | Yes | Up | - |
| Q9BSB4 | C12ORF44 | 68/114(59.65) | 0.39 | 2.53E-10 | 3.61E-09 | Yes | Up | - |
| O15091 | KIAA0391 | 53/114(46.49) | 0.18 | 2.80E-03 | 6.20E-03 | Yes | Up | - |
| P56537 | EIF6 | 59/114(51.75) | 0.37 | 2.88E-08 | 2.25E-07 | Yes | Up | - |
| Q92833 | JARID2 | 60/114(52.63) | 0.32 | 2.71E-05 | 9.75E-05 | Yes | Up | - |
| O75326 | SEMA7A | 66/114(57.89) | 0.68 | 2.92E-07 | 1.73E-06 | Yes | Up | - |
| P82979 | SARNP | 64/114(56.14) | 0.21 | 3.82E-05 | 1.33E-04 | Yes | Up | - |
| Q8N0Z3 | CCDC52 | 59/114(51.75) | 0.34 | 3.05E-04 | 8.54E-04 | Yes | Up | - |
| Q8TAA9 | VANGL1 | 55/114(48.25) | 0.66 | 1.21E-11 | 2.69E-10 | Yes | Up | - |
| P62993 | GRB2 | 56/114(49.12) | 0.35 | 5.01E-07 | 2.79E-06 | Yes | Up | - |
| Q53RY4 | KRTCAP3 | 60/114(52.63) | 0.7 | 1.28E-04 | 3.93E-04 | Yes | Up | - |
| P06746 | POLB | 67/114(58.77) | 0.61 | 7.82E-12 | 1.87E-10 | Yes | Up | - |
| Q9Y2D1 | ATF5 | 68/114(59.65) | 0.3 | 4.16E-05 | 1.43E-04 | Yes | Up | - |
| Q7Z2K6 | ERMP1 | 62/114(54.39) | 0.54 | 2.49E-06 | 1.16E-05 | Yes | Up | - |
| Q71RH2 | FAM57B | 63/114(55.26) | 1.44 | 9.28E-10 | 1.12E-08 | Yes | Up | - |
| Q96K58 | ZNF668 | 61/114(53.51) | 0.64 | 5.51E-13 | 1.96E-11 | Yes | Up | - |
| A6NIV6 | LRRIQ4 | 53/114(46.49) | 0.65 | 1.48E-03 | 3.51E-03 | Yes | Up | - |
| Q6ZNH5 | ZNF497 | 60/114(52.63) | 0.44 | 8.28E-05 | 2.65E-04 | Yes | Up | - |
| P78426 | NKX6-1 | 57/114(50.0) | 1.42 | 1.78E-12 | 5.27E-11 | Yes | Up | - |
| P04233 | CD74 | 60/114(52.63) | 0.26 | 1.62E-02 | 2.98E-02 | Yes | Up | - |
| Q99679 | GPR21 | 58/114(50.88) | 0.39 | 1.14E-02 | 2.19E-02 | Yes | Up | - |
| P05114 | HMGN1 | 58/114(50.88) | 0.48 | 5.40E-07 | 2.98E-06 | Yes | Up | - |
| - | LOC127841 | 52/114(45.61) | 0.87 | 3.76E-04 | 1.03E-03 | Yes | Up | - |
| O00148 | DDX39 | 59/114(51.75) | 0.82 | 7.63E-14 | 3.82E-12 | Yes | Up | - |
| Q5TDP6 | LGSN | 48/114(42.11) | 0.93 | 1.07E-03 | 2.61E-03 | Yes | Up | - |
| A1L190 | C22ORF41 | 62/114(54.39) | 0.89 | 1.16E-06 | 5.86E-06 | Yes | Up | - |
| Q53G59 | KLHL12 | 59/114(51.75) | 0.29 | 1.51E-05 | 5.81E-05 | Yes | Up | - |
| P34932 | HSPA4 | 62/114(54.39) | 0.33 | 1.36E-05 | 5.29E-05 | Yes | Up | - |
| Q08629 | SPOCK1 | 62/114(54.39) | 0.54 | 2.85E-04 | 8.02E-04 | Yes | Up | - |
| O15370 | SOX12 | 62/114(54.39) | 0.89 | 1.84E-12 | 5.42E-11 | Yes | Up | - |
| Q9HCC8 | GDPD2 | 50/114(43.86) | 0.91 | 4.50E-05 | 1.54E-04 | Yes | Up | - |
| Q05481 | ZNF91 | 58/114(50.88) | 0.31 | 1.17E-03 | 2.84E-03 | Yes | Up | - |
| Q8NAE3 | C1ORF180 | 48/114(42.11) | 0.88 | 3.95E-03 | 8.44E-03 | Yes | Up | - |
| Q96G25 | MED8 | 64/114(56.14) | 0.19 | 1.36E-03 | 3.27E-03 | Yes | Up | - |
| Q8IX07 | ZFPM1 | 69/114(60.53) | 0.47 | 1.06E-06 | 5.42E-06 | Yes | Up | - |
| O75956 | CDK2AP2 | 59/114(51.75) | 0.42 | 1.03E-06 | 5.26E-06 | Yes | Up | - |
| P28340 | POLD1 | 69/114(60.53) | 0.42 | 2.39E-06 | 1.11E-05 | Yes | Up | - |
| P56159 | GFRA1 | 67/114(58.77) | 0.47 | 2.44E-02 | 4.29E-02 | Yes | Up | - |
| Q86Y07 | VRK2 | 64/114(56.14) | 0.18 | 8.49E-03 | 1.68E-02 | Yes | Up | - |
| O95049 | TJP3 | 61/114(53.51) | 1.05 | 9.30E-07 | 4.82E-06 | Yes | Up | - |
| B4DVQ4 | C10ORF46 | 59/114(51.75) | 0.14 | 9.26E-03 | 1.81E-02 | Yes | Up | - |
| Q5T700 | LDLRAD1 | 54/114(47.37) | 1.16 | 1.02E-06 | 5.20E-06 | Yes | Up | - |
| Q969Q4 | ARL11 | 64/114(56.14) | 0.55 | 1.17E-06 | 5.88E-06 | Yes | Up | - |
| Q9NZH4 | PTTG3P | 54/114(47.37) | 1.02 | 1.05E-04 | 3.29E-04 | Yes | Up | - |
| P78330 | PSPH | 59/114(51.75) | 0.34 | 3.94E-04 | 1.07E-03 | Yes | Up | - |
| O43665 | RGS10 | 57/114(50.0) | 0.47 | 3.94E-07 | 2.25E-06 | Yes | Up | - |
| Q13951 | CBFB | 56/114(49.12) | 0.15 | 1.75E-02 | 3.19E-02 | Yes | Up | - |
| P51955 | NEK2 | 59/114(51.75) | 1.92 | 8.56E-15 | 6.46E-13 | Yes | Up | - |
| P80098 | CCL7 | 55/114(48.25) | 1.53 | 3.32E-06 | 1.49E-05 | Yes | Up | - |
| O00488 | ZNF593 | 67/114(58.77) | 0.43 | 7.05E-07 | 3.77E-06 | Yes | Up | - |
| Q6B0B8 | TIGD3 | 61/114(53.51) | 0.92 | 1.62E-09 | 1.82E-08 | Yes | Up | - |
| Q9Y6K5 | OAS3 | 56/114(49.12) | 0.79 | 1.38E-09 | 1.58E-08 | Yes | Up | - |
| P13501 | CCL5 | 56/114(49.12) | 0.29 | 2.63E-02 | 4.59E-02 | Yes | Up | - |
| Q96LK0 | C3ORF34 | 53/114(46.49) | 0.25 | 6.60E-04 | 1.71E-03 | Yes | Up | - |
| - | LOC148709 | 60/114(52.63) | 1.22 | 1.41E-10 | 2.19E-09 | Yes | Up | - |
| - | CXADRP3 | 56/114(49.12) | 1.27 | 2.55E-07 | 1.53E-06 | Yes | Up | - |
| Q2MV58 | TCTN1 | 62/114(54.39) | 0.23 | 2.38E-03 | 5.37E-03 | Yes | Up | - |
| Q9P2K2 | TXNDC16 | 50/114(43.86) | 0.18 | 2.48E-02 | 4.36E-02 | Yes | Up | - |
| Q9H8W5 | TRIM45 | 54/114(47.37) | 0.38 | 4.71E-04 | 1.26E-03 | Yes | Up | - |
| Q9BTN0 | LRFN3 | 62/114(54.39) | 0.31 | 2.45E-06 | 1.14E-05 | Yes | Up | - |
| Q9NR45 | NANS | 67/114(58.77) | 0.6 | 6.05E-11 | 1.06E-09 | Yes | Up | - |
| Q8TD06 | AGR3 | 60/114(52.63) | 1.42 | 8.74E-08 | 5.96E-07 | Yes | Up | - |
| Q9NZC3 | GDE1 | 60/114(52.63) | 0.18 | 3.86E-03 | 8.25E-03 | Yes | Up | - |
| P09228 | CST2 | 56/114(49.12) | 2.15 | 2.65E-12 | 7.36E-11 | Yes | Up | - |
| Q969M1 | TOMM40L | 62/114(54.39) | 0.22 | 2.67E-03 | 5.94E-03 | Yes | Up | - |
| Q15398 | DLGAP5 | 58/114(50.88) | 1.47 | 1.30E-10 | 2.05E-09 | Yes | Up | - |
| O96019 | ACTL6A | 56/114(49.12) | 0.5 | 3.80E-09 | 3.80E-08 | Yes | Up | - |
| Q8NC44 | FAM134A | 61/114(53.51) | 0.14 | 9.62E-04 | 2.39E-03 | Yes | Up | - |
| Q13349 | ITGAD | 59/114(51.75) | 0.69 | 1.01E-04 | 3.18E-04 | Yes | Up | - |
| Q02809 | PLOD1 | 59/114(51.75) | 0.26 | 4.53E-04 | 1.21E-03 | Yes | Up | - |
| O75427 | LRCH4 | 68/114(59.65) | 0.22 | 1.49E-04 | 4.50E-04 | Yes | Up | - |
| Q9Y6L7 | TLL2 | 61/114(53.51) | 1.09 | 4.27E-08 | 3.18E-07 | Yes | Up | - |
| Q7L7L0 | HIST3H2A | 63/114(55.26) | 0.92 | 3.62E-07 | 2.09E-06 | Yes | Up | - |
| Q13287 | NMI | 62/114(54.39) | 0.2 | 4.47E-03 | 9.45E-03 | Yes | Up | - |
| P50552 | VASP | 76/114(66.67) | 0.31 | 1.14E-06 | 5.77E-06 | Yes | Up | - |
| Q99575 | POP1 | 60/114(52.63) | 0.41 | 3.35E-05 | 1.18E-04 | Yes | Up | - |
| Q5SXM1 | ZNF678 | 55/114(48.25) | 0.28 | 8.18E-03 | 1.62E-02 | Yes | Up | - |
| Q15825 | CHRNA6 | 63/114(55.26) | 1.81 | 6.91E-11 | 1.19E-09 | Yes | Up | - |
| P52306 | RAP1GDS1 | 55/114(48.25) | 0.21 | 1.00E-02 | 1.94E-02 | Yes | Up | - |
| Q9Y2X7 | GIT1 | 60/114(52.63) | 0.21 | 8.00E-04 | 2.03E-03 | Yes | Up | - |
| - | PVT1 | 62/114(54.39) | 0.53 | 2.57E-04 | 7.32E-04 | Yes | Up | - |
| Q9Y2H0 | DLGAP4 | 56/114(49.12) | 0.12 | 2.25E-02 | 3.99E-02 | Yes | Up | - |
| Q8NFW5 | DMBX1 | 59/114(51.75) | 0.68 | 3.74E-03 | 8.03E-03 | Yes | Up | - |
| Q9UHR5 | SAP30BP | 55/114(48.25) | 0.11 | 2.57E-02 | 4.50E-02 | Yes | Up | - |
| Q92797 | SYMPK | 65/114(57.02) | 0.19 | 2.10E-04 | 6.10E-04 | Yes | Up | - |
| O15335 | CHAD | 53/114(46.49) | 1.08 | 7.13E-07 | 3.81E-06 | Yes | Up | - |
| Q56NI9 | ESCO2 | 57/114(50.0) | 1.49 | 4.92E-13 | 1.79E-11 | Yes | Up | - |
| P10412 | HIST1H1E | 58/114(50.88) | 0.83 | 3.56E-06 | 1.59E-05 | Yes | Up | - |
| Q6MZW2 | FSTL4 | 53/114(46.49) | 0.86 | 9.85E-06 | 3.97E-05 | Yes | Up | - |
| Q96C86 | DCPS | 69/114(60.53) | 0.26 | 3.46E-05 | 1.22E-04 | Yes | Up | - |
| Q14919 | DRAP1 | 57/114(50.0) | 0.49 | 4.38E-08 | 3.24E-07 | Yes | Up | - |
| P00558 | PGK1 | 61/114(53.51) | 0.31 | 1.12E-03 | 2.74E-03 | Yes | Up | - |
| Q5JTY5 | CBWD3 | 60/114(52.63) | 0.47 | 9.85E-05 | 3.10E-04 | Yes | Up | - |
| B0AZU2 | C22ORF9 | 56/114(49.12) | 0.18 | 8.52E-04 | 2.14E-03 | Yes | Up | - |
| P24941 | CDK2 | 58/114(50.88) | 0.4 | 4.60E-08 | 3.38E-07 | Yes | Up | - |
| P20702 | ITGAX | 64/114(56.14) | 0.74 | 1.74E-09 | 1.92E-08 | Yes | Up | - |
| Q03692 | COL10A1 | 62/114(54.39) | 2.79 | 3.82E-22 | 4.92E-19 | Yes | Up | - |
| P51805 | PLXNA3 | 68/114(59.65) | 0.41 | 6.05E-06 | 2.55E-05 | Yes | Up | - |
| Q8N1W2 | ZNF710 | 58/114(50.88) | 0.3 | 3.60E-04 | 9.89E-04 | Yes | Up | - |
| Q9NXB0 | MKS1 | 58/114(50.88) | 0.19 | 2.66E-03 | 5.92E-03 | Yes | Up | - |
| B7ZMH9 | YSK4 | 50/114(43.86) | 0.99 | 5.99E-05 | 1.99E-04 | Yes | Up | - |
| Q9Y3R0 | GRIP1 | 50/114(43.86) | 0.53 | 3.34E-04 | 9.23E-04 | Yes | Up | - |
| Q96FW1 | OTUB1 | 61/114(53.51) | 0.34 | 6.01E-09 | 5.68E-08 | Yes | Up | - |
| Q96PJ5 | FCRL4 | 55/114(48.25) | 1.28 | 1.51E-04 | 4.56E-04 | Yes | Up | - |
| O43396 | TXNL1 | 57/114(50.0) | 0.15 | 5.34E-03 | 1.11E-02 | Yes | Up | - |
| O43676 | NDUFB3 | 56/114(49.12) | 0.2 | 3.51E-03 | 7.58E-03 | Yes | Up | - |
| Q9UII5 | ZNF107 | 56/114(49.12) | 0.35 | 1.68E-03 | 3.94E-03 | Yes | Up | - |
| - | C1ORF170 | 67/114(58.77) | 0.6 | 1.31E-04 | 4.00E-04 | Yes | Up | - |
| Q9P0Z9 | PIPOX | 57/114(50.0) | 0.22 | 2.47E-02 | 4.33E-02 | Yes | Up | - |
| Q6ZMM2 | ADAMTSL5 | 63/114(55.26) | 0.52 | 4.55E-04 | 1.22E-03 | Yes | Up | - |
| Q9NWQ9 | C14ORF119 | 56/114(49.12) | 0.18 | 3.58E-04 | 9.84E-04 | Yes | Up | - |
| - | LOC541471 | 68/114(59.65) | 0.49 | 1.44E-05 | 5.57E-05 | Yes | Up | - |
| Q8N9W8 | FAM71D | 58/114(50.88) | 0.52 | 1.55E-08 | 1.31E-07 | Yes | Up | - |
| Q01196 | RUNX1 | 61/114(53.51) | 0.28 | 2.87E-03 | 6.33E-03 | Yes | Up | - |
| P29372 | MPG | 72/114(63.16) | 0.45 | 1.18E-08 | 1.03E-07 | Yes | Up | - |
| Q5TCQ9 | MAGI3 | 48/114(42.11) | 0.29 | 5.56E-03 | 1.15E-02 | Yes | Up | - |
| Q6PI26 | SHQ1 | 60/114(52.63) | 0.23 | 1.38E-04 | 4.21E-04 | Yes | Up | - |
| P83916 | CBX1 | 52/114(45.61) | 0.3 | 4.64E-05 | 1.58E-04 | Yes | Up | - |
| - | C14ORF184 | 57/114(50.0) | 0.44 | 1.49E-02 | 2.77E-02 | Yes | Up | - |
| Q9BYX2 | TBC1D2 | 57/114(50.0) | 0.37 | 1.57E-05 | 6.02E-05 | Yes | Up | - |
| Q4VC31 | CCDC58 | 59/114(51.75) | 0.32 | 1.15E-05 | 4.56E-05 | Yes | Up | - |
| Q8TD31 | CCHCR1 | 65/114(57.02) | 0.21 | 8.04E-03 | 1.60E-02 | Yes | Up | - |
| P32189 | GK | 59/114(51.75) | 0.67 | 5.40E-09 | 5.17E-08 | Yes | Up | - |
| Q5VZ03 | NXNL2 | 57/114(50.0) | 0.52 | 9.58E-03 | 1.86E-02 | Yes | Up | - |
| Q9UHQ9 | CYB5R1 | 55/114(48.25) | 0.33 | 1.64E-05 | 6.25E-05 | Yes | Up | - |
| O95886 | DLGAP3 | 59/114(51.75) | 0.56 | 7.81E-04 | 1.98E-03 | Yes | Up | - |
| Q969H8 | C19ORF10 | 64/114(56.14) | 0.55 | 2.08E-10 | 3.05E-09 | Yes | Up | - |
| Q6IPU0 | CENPP | 61/114(53.51) | 0.62 | 3.64E-07 | 2.10E-06 | Yes | Up | - |
| Q96NL6 | SCLT1 | 58/114(50.88) | 0.12 | 2.41E-02 | 4.25E-02 | Yes | Up | - |
| Q96EN9 | C19ORF60 | 68/114(59.65) | 0.23 | 8.73E-03 | 1.72E-02 | Yes | Up | - |
| Q99460 | PSMD1 | 53/114(46.49) | 0.16 | 1.85E-02 | 3.35E-02 | Yes | Up | - |
| Q9NYY1 | IL20 | 59/114(51.75) | 1.71 | 2.93E-11 | 5.79E-10 | Yes | Up | - |
| P49590 | HARS2 | 57/114(50.0) | 0.17 | 2.41E-04 | 6.90E-04 | Yes | Up | - |
| P42684 | ABL2 | 59/114(51.75) | 0.26 | 1.33E-05 | 5.18E-05 | Yes | Up | - |
| Q8NBM8 | PCYOX1L | 62/114(54.39) | 0.33 | 6.24E-07 | 3.38E-06 | Yes | Up | - |
| Q9C099 | LRRCC1 | 60/114(52.63) | 0.37 | 6.36E-05 | 2.09E-04 | Yes | Up | - |
| O95407 | TNFRSF6B | 68/114(59.65) | 0.29 | 1.06E-02 | 2.04E-02 | Yes | Up | - |
| P60896 | SHFM1 | 67/114(58.77) | 0.33 | 2.43E-06 | 1.13E-05 | Yes | Up | - |
| - | FMO9P | 40/114(35.09) | 2.3 | 3.72E-10 | 5.07E-09 | Yes | Up | - |
| Q8N1E6 | FBXL14 | 58/114(50.88) | 0.17 | 7.63E-04 | 1.94E-03 | Yes | Up | - |
| Q5VZK9 | LRRC16A | 61/114(53.51) | 0.23 | 6.59E-03 | 1.34E-02 | Yes | Up | - |
| Q15906 | VPS72 | 60/114(52.63) | 0.38 | 3.19E-07 | 1.87E-06 | Yes | Up | - |
| Q5JU67 | C9ORF117 | 55/114(48.25) | 0.48 | 1.49E-03 | 3.54E-03 | Yes | Up | - |
| O95447 | LCA5L | 68/114(59.65) | 0.49 | 9.65E-06 | 3.89E-05 | Yes | Up | - |
| P19652 | ORM2 | 55/114(48.25) | 1.6 | 8.51E-07 | 4.46E-06 | Yes | Up | - |
| Q9BQY6 | WFDC6 | 54/114(47.37) | 1.4 | 1.02E-06 | 5.21E-06 | Yes | Up | - |
| - | LOC100134368 | 61/114(53.51) | 0.43 | 1.19E-03 | 2.90E-03 | Yes | Up | - |
| Q8NHY0 | B4GALNT2 | 51/114(44.74) | 1.54 | 9.58E-10 | 1.15E-08 | Yes | Up | - |
| P31146 | CORO1A | 62/114(54.39) | 0.42 | 1.96E-04 | 5.74E-04 | Yes | Up | - |
| Q658Y4 | FAM91A1 | 61/114(53.51) | 0.27 | 3.17E-03 | 6.91E-03 | Yes | Up | - |
| O60568 | PLOD3 | 57/114(50.0) | 0.24 | 2.82E-03 | 6.22E-03 | Yes | Up | - |
| O95922 | TTLL1 | 58/114(50.88) | 0.21 | 1.41E-03 | 3.36E-03 | Yes | Up | - |
| Q15390 | MTFR1 | 55/114(48.25) | 0.38 | 8.46E-05 | 2.71E-04 | Yes | Up | - |
| P28749 | RBL1 | 60/114(52.63) | 0.35 | 1.58E-03 | 3.72E-03 | Yes | Up | - |
| Q8WXD9 | CASKIN1 | 58/114(50.88) | 0.69 | 4.56E-04 | 1.22E-03 | Yes | Up | - |
| Q8N2K0 | ABHD12 | 61/114(53.51) | 0.57 | 2.18E-10 | 3.18E-09 | Yes | Up | - |
| - | LOC348926 | 58/114(50.88) | 0.24 | 4.61E-03 | 9.71E-03 | Yes | Up | - |
| Q07864 | POLE | 59/114(51.75) | 0.34 | 4.00E-07 | 2.28E-06 | Yes | Up | - |
| Q9UPN7 | SAPS1 | 64/114(56.14) | 0.38 | 1.04E-07 | 6.98E-07 | Yes | Up | - |
| Q99643 | SDHC | 59/114(51.75) | 0.3 | 1.03E-04 | 3.24E-04 | Yes | Up | - |
| - | LOC645332 | 56/114(49.12) | 0.24 | 3.34E-03 | 7.25E-03 | Yes | Up | - |
| - | LOC100128675 | 62/114(54.39) | 0.88 | 2.26E-05 | 8.29E-05 | Yes | Up | - |
| Q9BVG3 | TRIM62 | 65/114(57.02) | 0.42 | 1.40E-06 | 6.94E-06 | Yes | Up | - |
| Q6UWP7 | LCLAT1 | 62/114(54.39) | 0.31 | 3.66E-04 | 1.00E-03 | Yes | Up | - |
| O43760 | SYNGR2 | 65/114(57.02) | 0.57 | 5.08E-10 | 6.66E-09 | Yes | Up | - |
| Q9H0W8 | C19ORF61 | 61/114(53.51) | 0.31 | 8.56E-08 | 5.86E-07 | Yes | Up | - |
| Q7Z465 | BNIPL | 58/114(50.88) | 0.58 | 6.52E-03 | 1.33E-02 | Yes | Up | - |
| Q9Y5Y0 | FLVCR1 | 56/114(49.12) | 0.89 | 1.08E-10 | 1.74E-09 | Yes | Up | - |
| P02144 | MB | 60/114(52.63) | 0.88 | 4.73E-07 | 2.65E-06 | Yes | Up | - |
| Q6ZV77 | C9ORF139 | 57/114(50.0) | 0.38 | 1.34E-02 | 2.51E-02 | Yes | Up | - |
| O95831 | AIFM1 | 54/114(47.37) | 0.29 | 6.76E-05 | 2.21E-04 | Yes | Up | - |
| Q9Y679 | AUP1 | 60/114(52.63) | 0.28 | 3.46E-06 | 1.55E-05 | Yes | Up | - |
| Q9H853 | TUBA4B | 38/114(33.33) | 1.6 | 3.01E-06 | 1.37E-05 | Yes | Up | - |
| O00468 | AGRN | 64/114(56.14) | 0.36 | 8.09E-04 | 2.05E-03 | Yes | Up | - |
| Q9UBC3 | DNMT3B | 52/114(45.61) | 0.68 | 4.76E-08 | 3.49E-07 | Yes | Up | - |
| Q8TDQ0 | HAVCR2 | 59/114(51.75) | 0.33 | 2.31E-03 | 5.22E-03 | Yes | Up | - |
| Q8NEV9 | IL27 | 56/114(49.12) | 0.76 | 2.18E-04 | 6.33E-04 | Yes | Up | - |
| P23246 | SFPQ | 61/114(53.51) | 0.32 | 5.32E-08 | 3.84E-07 | Yes | Up | - |
| P54136 | RARS | 61/114(53.51) | 0.21 | 5.43E-05 | 1.82E-04 | Yes | Up | - |
| Q9Y5K5 | UCHL5 | 51/114(44.74) | 0.27 | 8.22E-04 | 2.08E-03 | Yes | Up | - |
| Q8NET6 | CHST13 | 59/114(51.75) | 0.37 | 3.28E-03 | 7.12E-03 | Yes | Up | - |
| Q9H7Z7 | PTGES2 | 64/114(56.14) | 0.24 | 3.15E-04 | 8.78E-04 | Yes | Up | - |
| Q8NHM5 | KDM2B | 56/114(49.12) | 0.2 | 2.39E-04 | 6.85E-04 | Yes | Up | - |
| Q9H3Y8 | PPDPF | 68/114(59.65) | 0.49 | 1.16E-07 | 7.62E-07 | Yes | Up | - |
| O43819 | SCO2 | 62/114(54.39) | 0.42 | 7.43E-08 | 5.18E-07 | Yes | Up | - |
| Q9UHV9 | PFDN2 | 64/114(56.14) | 0.54 | 7.57E-09 | 6.97E-08 | Yes | Up | - |
| P12757 | SKIL | 61/114(53.51) | 0.4 | 2.10E-05 | 7.80E-05 | Yes | Up | - |
| Q96E14 | C16ORF75 | 61/114(53.51) | 1.25 | 5.68E-13 | 2.01E-11 | Yes | Up | - |
| P31785 | IL2RG | 55/114(48.25) | 0.33 | 1.38E-02 | 2.58E-02 | Yes | Up | - |
| O95372 | LYPLA2 | 66/114(57.89) | 0.5 | 2.91E-11 | 5.75E-10 | Yes | Up | - |
| P53396 | ACLY | 54/114(47.37) | 0.24 | 3.48E-03 | 7.51E-03 | Yes | Up | - |
| Q6L9W6 | B4GALNT3 | 62/114(54.39) | 0.72 | 2.55E-05 | 9.26E-05 | Yes | Up | - |
| P33993 | MCM7 | 62/114(54.39) | 0.18 | 2.52E-02 | 4.42E-02 | Yes | Up | - |
| P49458 | SRP9 | 57/114(50.0) | 0.5 | 5.46E-08 | 3.93E-07 | Yes | Up | - |
| Q9HCU8 | POLD4 | 65/114(57.02) | 0.5 | 4.90E-08 | 3.57E-07 | Yes | Up | - |
| Q9P219 | CCDC88C | 63/114(55.26) | 0.43 | 7.57E-06 | 3.12E-05 | Yes | Up | - |
| Q969Y2 | GTPBP3 | 61/114(53.51) | 0.46 | 3.59E-09 | 3.63E-08 | Yes | Up | - |
| Q8IYS5 | OSCAR | 68/114(59.65) | 0.93 | 2.09E-13 | 8.76E-12 | Yes | Up | - |
| O14862 | AIM2 | 67/114(58.77) | 0.85 | 5.97E-07 | 3.26E-06 | Yes | Up | - |
| Q13007 | IL24 | 61/114(53.51) | 0.97 | 9.43E-07 | 4.88E-06 | Yes | Up | - |
| Q9NSV4 | DIAPH3 | 60/114(52.63) | 0.98 | 1.65E-07 | 1.04E-06 | Yes | Up | - |
| O00189 | AP4M1 | 60/114(52.63) | 0.35 | 3.92E-09 | 3.91E-08 | Yes | Up | - |
| Q14994 | NR1I3 | 58/114(50.88) | 0.31 | 1.55E-03 | 3.67E-03 | Yes | Up | - |
| Q9BQT9 | CLSTN3 | 58/114(50.88) | 0.41 | 8.27E-06 | 3.39E-05 | Yes | Up | - |
| O60610 | DIAPH1 | 59/114(51.75) | 0.15 | 3.67E-03 | 7.89E-03 | Yes | Up | - |
| Q86W33 | TPRA1 | 61/114(53.51) | 0.15 | 9.25E-04 | 2.30E-03 | Yes | Up | - |
| Q6PJG9 | LRFN4 | 53/114(46.49) | 0.28 | 5.32E-03 | 1.10E-02 | Yes | Up | - |
| O75843 | AP1G2 | 60/114(52.63) | 0.47 | 8.48E-06 | 3.46E-05 | Yes | Up | - |
| P13645 | KRT10 | 67/114(58.77) | 0.28 | 2.01E-03 | 4.62E-03 | Yes | Up | - |
| Q9Y2Q5 | ROBLD3 | 63/114(55.26) | 0.75 | 1.44E-13 | 6.47E-12 | Yes | Up | - |
| Q9H093 | NUAK2 | 57/114(50.0) | 1.2 | 5.20E-15 | 4.36E-13 | Yes | Up | - |
| Q9Y6Q5 | AP1M2 | 60/114(52.63) | 0.93 | 1.28E-06 | 6.40E-06 | Yes | Up | - |
| Q9NVM9 | C12ORF11 | 54/114(47.37) | 0.25 | 2.01E-04 | 5.87E-04 | Yes | Up | - |
| P02708 | CHRNA1 | 61/114(53.51) | 1.17 | 1.73E-07 | 1.09E-06 | Yes | Up | - |
| P52823 | STC1 | 60/114(52.63) | 0.39 | 6.55E-03 | 1.33E-02 | Yes | Up | - |
| Q9UBS4 | DNAJB11 | 64/114(56.14) | 0.44 | 1.57E-09 | 1.77E-08 | Yes | Up | - |
| Q9Y6I4 | USP3 | 57/114(50.0) | 0.16 | 2.14E-03 | 4.89E-03 | Yes | Up | - |
| P78367 | NKX3-2 | 59/114(51.75) | 1.62 | 3.70E-14 | 2.16E-12 | Yes | Up | - |
| Q9P2E8 | Mar-04 | 58/114(50.88) | 1.27 | 2.76E-05 | 9.90E-05 | Yes | Up | - |
| Q7L3S4 | ZNF771 | 70/114(61.4) | 0.31 | 7.01E-05 | 2.29E-04 | Yes | Up | - |
| Q9BTP6 | ZBED2 | 57/114(50.0) | 0.93 | 2.23E-05 | 8.22E-05 | Yes | Up | - |
| Q5JTW2 | CEP78 | 49/114(42.98) | 0.18 | 2.08E-03 | 4.76E-03 | Yes | Up | - |
| Q3SYB3 | FOXD4L6 | 55/114(48.25) | 0.76 | 1.16E-04 | 3.59E-04 | Yes | Up | - |
| P32322 | PYCR1 | 59/114(51.75) | 1.36 | 2.10E-16 | 3.23E-14 | Yes | Up | - |
| Q6P1J9 | CDC73 | 50/114(43.86) | 0.15 | 1.94E-02 | 3.50E-02 | Yes | Up | - |
| P80723 | BASP1 | 62/114(54.39) | 0.64 | 2.60E-07 | 1.56E-06 | Yes | Up | - |
| Q9UKW4 | VAV3 | 59/114(51.75) | 0.97 | 9.95E-09 | 8.85E-08 | Yes | Up | - |
| Q9BZD2 | SLC29A3 | 61/114(53.51) | 0.64 | 1.01E-10 | 1.65E-09 | Yes | Up | - |
| - | LOC388796 | 59/114(51.75) | 0.23 | 4.76E-03 | 9.98E-03 | Yes | Up | - |
| - | LOC145837 | 61/114(53.51) | 0.69 | 1.07E-03 | 2.63E-03 | Yes | Up | - |
| P17482 | HOXB9 | 57/114(50.0) | 1.06 | 1.94E-10 | 2.88E-09 | Yes | Up | - |
| Q8WUM0 | NUP133 | 59/114(51.75) | 0.14 | 1.57E-02 | 2.90E-02 | Yes | Up | - |
| Q5TKA1 | LIN9 | 60/114(52.63) | 0.43 | 5.23E-05 | 1.76E-04 | Yes | Up | - |
| Q8NGS3 | OR1J1 | 28/114(24.56) | 0.83 | 1.55E-02 | 2.86E-02 | Yes | Up | - |
| Q9BT22 | ALG1 | 63/114(55.26) | 0.49 | 6.45E-10 | 8.17E-09 | Yes | Up | - |
| Q8NF86 | PRSS33 | 44/114(38.6) | 1.69 | 8.12E-08 | 5.60E-07 | Yes | Up | - |
| O14975 | SLC27A2 | 55/114(48.25) | 0.48 | 1.28E-02 | 2.41E-02 | Yes | Up | - |
| P01116 | KRAS | 57/114(50.0) | 0.31 | 1.58E-04 | 4.74E-04 | Yes | Up | - |
| Q96KD3 | FAM71F1 | 51/114(44.74) | 0.83 | 7.52E-04 | 1.92E-03 | Yes | Up | - |
| Q96NY9 | MUS81 | 66/114(57.89) | 0.15 | 2.14E-03 | 4.89E-03 | Yes | Up | - |
| P61006 | RAB8A | 57/114(50.0) | 0.54 | 4.03E-15 | 3.59E-13 | Yes | Up | - |
| P62330 | ARF6 | 65/114(57.02) | 0.25 | 8.75E-06 | 3.56E-05 | Yes | Up | - |
| Q99456 | KRT12 | 48/114(42.11) | 0.83 | 3.65E-04 | 1.00E-03 | Yes | Up | - |
| Q71H61 | ILDR2 | 60/114(52.63) | 1.14 | 3.00E-08 | 2.33E-07 | Yes | Up | - |
| Q6UVY6 | MOXD1 | 63/114(55.26) | 0.74 | 9.14E-09 | 8.21E-08 | Yes | Up | - |
| P53801 | PTTG1IP | 63/114(55.26) | 0.24 | 7.48E-05 | 2.42E-04 | Yes | Up | - |
| Q9BZJ0 | CRNKL1 | 56/114(49.12) | 0.26 | 2.22E-04 | 6.43E-04 | Yes | Up | - |
| Q8NHG7 | SVIP | 53/114(46.49) | 0.25 | 1.06E-03 | 2.61E-03 | Yes | Up | - |
| Q8NHE4 | ATP6V0E2 | 53/114(46.49) | 0.28 | 3.11E-03 | 6.79E-03 | Yes | Up | - |
| P07949 | RET | 67/114(58.77) | 1.23 | 2.40E-09 | 2.54E-08 | Yes | Up | - |
| A6NKF9 | GPR89C | 58/114(50.88) | 0.39 | 1.23E-03 | 2.98E-03 | Yes | Up | - |
| Q6ZRS2 | SRCAP | 57/114(50.0) | 0.2 | 3.21E-04 | 8.91E-04 | Yes | Up | - |
| Q99062 | CSF3R | 56/114(49.12) | 0.35 | 8.39E-03 | 1.66E-02 | Yes | Up | - |
| Q86TI2 | DPP9 | 62/114(54.39) | 0.44 | 3.71E-10 | 5.06E-09 | Yes | Up | - |
| O43900 | PRICKLE3 | 60/114(52.63) | 0.26 | 2.79E-05 | 1.00E-04 | Yes | Up | - |
| Q06432 | CACNG1 | 63/114(55.26) | 1.34 | 9.45E-09 | 8.46E-08 | Yes | Up | - |
| P04198 | MYCN | 55/114(48.25) | 0.33 | 2.83E-02 | 4.89E-02 | Yes | Up | - |
| P46020 | PHKA1 | 56/114(49.12) | 0.25 | 7.87E-03 | 1.57E-02 | Yes | Up | - |
| Q8IXW0 | C11ORF35 | 61/114(53.51) | 0.6 | 4.70E-05 | 1.60E-04 | Yes | Up | - |
| P53999 | SUB1 | 64/114(56.14) | 0.18 | 2.71E-02 | 4.71E-02 | Yes | Up | - |
| Q9NWC5 | TMEM45A | 55/114(48.25) | 0.4 | 1.70E-03 | 3.97E-03 | Yes | Up | - |
| O43292 | GPAA1 | 65/114(57.02) | 0.26 | 7.65E-04 | 1.95E-03 | Yes | Up | - |
| O60869 | EDF1 | 65/114(57.02) | 0.27 | 6.80E-05 | 2.22E-04 | Yes | Up | - |
| Q6UXN2 | TREML4 | 31/114(27.19) | 0.79 | 1.49E-02 | 2.77E-02 | Yes | Up | - |
| P23258 | TUBG1 | 61/114(53.51) | 0.5 | 1.04E-07 | 6.94E-07 | Yes | Up | - |
| O75764 | TCEA3 | 62/114(54.39) | 0.51 | 8.24E-07 | 4.33E-06 | Yes | Up | - |
| Q9BXJ9 | NAA15 | 56/114(49.12) | 0.19 | 8.68E-03 | 1.71E-02 | Yes | Up | - |
| Q16548 | BCL2A1 | 59/114(51.75) | 0.53 | 1.14E-04 | 3.55E-04 | Yes | Up | - |
| Q6UE05 | WBSCR28 | 56/114(49.12) | 2.25 | 2.65E-13 | 1.07E-11 | Yes | Up | - |
| P18887 | XRCC1 | 62/114(54.39) | 0.24 | 2.78E-05 | 9.97E-05 | Yes | Up | - |
| P63313 | TMSB10 | 65/114(57.02) | 0.36 | 2.45E-04 | 7.01E-04 | Yes | Up | - |
| - | RPSAP52 | 67/114(58.77) | 1.07 | 6.04E-09 | 5.70E-08 | Yes | Up | - |
| Q86YS3 | RAB11FIP4 | 58/114(50.88) | 0.67 | 4.58E-06 | 1.98E-05 | Yes | Up | - |
| Q8NBM4 | UBAC2 | 52/114(45.61) | 0.12 | 1.31E-02 | 2.47E-02 | Yes | Up | - |
| Q9H0T7 | RAB17 | 57/114(50.0) | 0.64 | 1.70E-04 | 5.07E-04 | Yes | Up | - |
| Q8TDF5 | NETO1 | 58/114(50.88) | 0.71 | 2.44E-03 | 5.49E-03 | Yes | Up | - |
| - | LOC339674 | 62/114(54.39) | 0.92 | 1.87E-05 | 7.03E-05 | Yes | Up | - |
| Q13111 | CHAF1A | 62/114(54.39) | 0.48 | 4.77E-08 | 3.49E-07 | Yes | Up | - |
| Q7Z602 | GPR141 | 60/114(52.63) | 1.02 | 2.07E-09 | 2.23E-08 | Yes | Up | - |
| Q9NQZ7 | ENTPD7 | 56/114(49.12) | 0.85 | 1.57E-11 | 3.35E-10 | Yes | Up | - |
| P78383 | SLC35B1 | 65/114(57.02) | 0.45 | 1.25E-07 | 8.14E-07 | Yes | Up | - |
| B4DHA3 | TMEM48 | 53/114(46.49) | 0.29 | 1.22E-03 | 2.95E-03 | Yes | Up | - |
| F5H5Y1 | GPR172B | 54/114(47.37) | 0.67 | 3.51E-05 | 1.23E-04 | Yes | Up | - |
| Q9NVS9 | PNPO | 54/114(47.37) | 0.23 | 3.31E-03 | 7.20E-03 | Yes | Up | - |
| Q92619 | HMHA1 | 68/114(59.65) | 0.22 | 1.53E-02 | 2.83E-02 | Yes | Up | - |
| - | NCRNA00160 | 61/114(53.51) | 1.31 | 2.76E-09 | 2.87E-08 | Yes | Up | - |
| Q9Y251 | HPSE | 57/114(50.0) | 0.49 | 2.42E-04 | 6.92E-04 | Yes | Up | - |
| Q13421 | MSLN | 57/114(50.0) | 1.06 | 4.69E-05 | 1.59E-04 | Yes | Up | - |
| Q9H497 | TOR3A | 58/114(50.88) | 0.65 | 1.23E-16 | 2.04E-14 | Yes | Up | - |
| P00441 | SOD1 | 62/114(54.39) | 0.17 | 1.70E-02 | 3.12E-02 | Yes | Up | - |
| Q6ZW33 | MICALCL | 56/114(49.12) | 0.81 | 1.88E-07 | 1.17E-06 | Yes | Up | - |
| Q96AC6 | KIFC2 | 60/114(52.63) | 0.81 | 3.56E-10 | 4.87E-09 | Yes | Up | - |
| Q9HA64 | FN3KRP | 54/114(47.37) | 0.14 | 5.25E-03 | 1.09E-02 | Yes | Up | - |
| Q99611 | SEPHS2 | 60/114(52.63) | 0.62 | 6.90E-10 | 8.65E-09 | Yes | Up | - |
| Q8NF37 | LPCAT1 | 63/114(55.26) | 0.43 | 3.34E-05 | 1.18E-04 | Yes | Up | - |
| Q9H6X4 | TMEM134 | 67/114(58.77) | 0.38 | 4.42E-06 | 1.92E-05 | Yes | Up | - |
| P50219 | MNX1 | 66/114(57.89) | 0.92 | 8.15E-05 | 2.62E-04 | Yes | Up | - |
| Q8N292 | GAPT | 57/114(50.0) | 0.66 | 2.26E-06 | 1.06E-05 | Yes | Up | - |
| Q8WVP5 | TNFAIP8L1 | 68/114(59.65) | 0.45 | 5.03E-07 | 2.80E-06 | Yes | Up | - |
| - | LOC220429 | 58/114(50.88) | 0.28 | 1.82E-02 | 3.31E-02 | Yes | Up | - |
| Q9NPG3 | UBN1 | 56/114(49.12) | 0.15 | 5.11E-03 | 1.06E-02 | Yes | Up | - |
| Q92485 | SMPDL3B | 57/114(50.0) | 0.49 | 3.66E-03 | 7.87E-03 | Yes | Up | - |
| P35236 | PTPN7 | 65/114(57.02) | 0.53 | 3.87E-05 | 1.34E-04 | Yes | Up | - |
| Q9NWS9 | ZNF446 | 63/114(55.26) | 0.39 | 4.00E-07 | 2.28E-06 | Yes | Up | - |
| O15541 | RNF113A | 63/114(55.26) | 0.19 | 5.50E-05 | 1.84E-04 | Yes | Up | - |
| Q13105 | ZBTB17 | 68/114(59.65) | 0.17 | 7.00E-03 | 1.41E-02 | Yes | Up | - |
| Q6UWP8 | SBSN | 50/114(43.86) | 1.35 | 3.90E-06 | 1.72E-05 | Yes | Up | - |
| P61011 | SRP54 | 58/114(50.88) | 0.36 | 8.67E-07 | 4.53E-06 | Yes | Up | - |
| A0AV96 | RBM47 | 56/114(49.12) | 0.49 | 7.62E-06 | 3.14E-05 | Yes | Up | - |
| Q8WV07 | ORAOV1 | 62/114(54.39) | 0.21 | 4.99E-03 | 1.04E-02 | Yes | Up | - |
| Q7Z7B1 | PIGW | 55/114(48.25) | 0.27 | 9.26E-04 | 2.30E-03 | Yes | Up | - |
| P52435 | POLR2J | 63/114(55.26) | 0.27 | 4.31E-05 | 1.48E-04 | Yes | Up | - |
| Q5JPH6 | EARS2 | 56/114(49.12) | 0.31 | 1.40E-04 | 4.25E-04 | Yes | Up | - |
| Q6ZWJ1 | STXBP4 | 50/114(43.86) | 0.27 | 2.59E-03 | 5.78E-03 | Yes | Up | - |
| P33947 | KDELR2 | 56/114(49.12) | 0.34 | 5.83E-06 | 2.47E-05 | Yes | Up | - |
| Q969E8 | TSR2 | 65/114(57.02) | 0.13 | 1.08E-02 | 2.08E-02 | Yes | Up | - |
| O15479 | MAGEB2 | 37/114(32.46) | 1.31 | 4.73E-05 | 1.61E-04 | Yes | Up | - |
| Q96FF9 | CDCA5 | 60/114(52.63) | 1.4 | 1.52E-13 | 6.80E-12 | Yes | Up | - |
| Q7Z5Y6 | BMP8A | 57/114(50.0) | 1.42 | 2.50E-15 | 2.46E-13 | Yes | Up | - |
| P13725 | OSM | 62/114(54.39) | 0.62 | 7.86E-05 | 2.54E-04 | Yes | Up | - |
| Q6ZQX7 | C17ORF97 | 60/114(52.63) | 0.25 | 6.21E-03 | 1.27E-02 | Yes | Up | - |
| Q5HY98 | ZNF766 | 58/114(50.88) | 0.16 | 4.02E-03 | 8.58E-03 | Yes | Up | - |
| Q5T8I9 | C1ORF59 | 57/114(50.0) | 0.27 | 1.11E-02 | 2.13E-02 | Yes | Up | - |
| Q86WK7 | AMIGO3 | 65/114(57.02) | 0.51 | 3.96E-08 | 2.97E-07 | Yes | Up | - |
| Q9H832 | UBE2Z | 53/114(46.49) | 0.31 | 1.57E-05 | 6.02E-05 | Yes | Up | - |
| Q9H875 | PRKRIP1 | 66/114(57.89) | 0.18 | 9.72E-04 | 2.41E-03 | Yes | Up | - |
| P48730 | CSNK1D | 53/114(46.49) | 0.11 | 1.59E-02 | 2.94E-02 | Yes | Up | - |
| Q9Y2A4 | ZNF443 | 50/114(43.86) | 0.34 | 1.24E-04 | 3.82E-04 | Yes | Up | - |
| C9JDP6 | CLDN25 | 40/114(35.09) | 1.81 | 2.86E-07 | 1.70E-06 | Yes | Up | - |
| Q6PI48 | DARS2 | 56/114(49.12) | 0.51 | 4.29E-07 | 2.43E-06 | Yes | Up | - |
| Q2NL98 | VMAC | 57/114(50.0) | 0.14 | 9.00E-03 | 1.77E-02 | Yes | Up | - |
| Q9C0D9 | EPT1 | 54/114(47.37) | 0.47 | 3.67E-05 | 1.28E-04 | Yes | Up | - |
| Q13144 | EIF2B5 | 63/114(55.26) | 0.22 | 3.58E-06 | 1.59E-05 | Yes | Up | - |
| Q96C19 | EFHD2 | 61/114(53.51) | 0.39 | 2.89E-06 | 1.32E-05 | Yes | Up | - |
| Q12931 | TRAP1 | 61/114(53.51) | 0.27 | 1.29E-04 | 3.96E-04 | Yes | Up | - |
| P23526 | AHCY | 62/114(54.39) | 0.33 | 5.63E-05 | 1.88E-04 | Yes | Up | - |
| Q96RG2 | PASK | 62/114(54.39) | 0.59 | 2.35E-09 | 2.49E-08 | Yes | Up | - |
| Q02817 | MUC2 | 51/114(44.74) | 3.59 | 2.79E-27 | 1.79E-23 | Yes | Up | - |
| P41743 | PRKCI | 60/114(52.63) | 0.19 | 1.02E-02 | 1.97E-02 | Yes | Up | - |
| O60814 | HIST1H2BK | 58/114(50.88) | 1.02 | 2.02E-12 | 5.89E-11 | Yes | Up | - |
| Q9UPM9 | B9D1 | 66/114(57.89) | 0.25 | 2.67E-03 | 5.94E-03 | Yes | Up | - |
| Q14847 | LASP1 | 62/114(54.39) | 0.25 | 2.88E-03 | 6.35E-03 | Yes | Up | - |
| Q9NY25 | CLEC5A | 58/114(50.88) | 1.63 | 4.22E-15 | 3.72E-13 | Yes | Up | - |
| B4DRX9 | C17ORF63 | 55/114(48.25) | 0.25 | 5.37E-05 | 1.80E-04 | Yes | Up | - |
| Q6IPM2 | IQCE | 57/114(50.0) | 0.34 | 2.15E-05 | 7.96E-05 | Yes | Up | - |
| Q10981 | FUT2 | 54/114(47.37) | 0.54 | 2.45E-03 | 5.51E-03 | Yes | Up | - |
| Q9BPX5 | ARPC5L | 66/114(57.89) | 0.39 | 2.14E-07 | 1.31E-06 | Yes | Up | - |
| Q96H78 | SLC25A44 | 59/114(51.75) | 0.37 | 3.30E-08 | 2.54E-07 | Yes | Up | - |
| Q9Y619 | SLC25A15 | 47/114(41.23) | 0.27 | 4.99E-03 | 1.04E-02 | Yes | Up | - |
| P0C1Z6 | TFPT | 71/114(62.28) | 0.51 | 3.11E-07 | 1.82E-06 | Yes | Up | - |
| Q66K64 | DCAF15 | 66/114(57.89) | 0.26 | 3.20E-05 | 1.14E-04 | Yes | Up | - |
| O96017 | CHEK2 | 67/114(58.77) | 0.49 | 6.12E-08 | 4.35E-07 | Yes | Up | - |
| Q9H6A9 | PCNXL3 | 58/114(50.88) | 0.3 | 9.34E-06 | 3.78E-05 | Yes | Up | - |
| O15496 | PLA2G10 | 54/114(47.37) | 1.75 | 4.82E-14 | 2.67E-12 | Yes | Up | - |
| O95857 | TSPAN13 | 62/114(54.39) | 0.8 | 6.79E-10 | 8.53E-09 | Yes | Up | - |
| Q16553 | LY6E | 70/114(61.4) | 0.36 | 1.69E-03 | 3.96E-03 | Yes | Up | - |
| Q9BZK7 | TBL1XR1 | 55/114(48.25) | 0.25 | 3.85E-04 | 1.05E-03 | Yes | Up | - |
| Q92544 | TM9SF4 | 56/114(49.12) | 0.22 | 3.04E-05 | 1.08E-04 | Yes | Up | - |
| P58505 | C21ORF58 | 62/114(54.39) | 0.68 | 1.33E-08 | 1.14E-07 | Yes | Up | - |
| Q8WWG9 | KCNE4 | 58/114(50.88) | 1.11 | 1.66E-10 | 2.52E-09 | Yes | Up | - |
| Q2Y0W8 | SLC4A8 | 65/114(57.02) | 0.81 | 1.02E-05 | 4.08E-05 | Yes | Up | - |
| Q7RTY8 | TMPRSS7 | 39/114(34.21) | 1.03 | 1.72E-04 | 5.10E-04 | Yes | Up | - |
| Q6PHR2 | ULK3 | 57/114(50.0) | 0.26 | 2.78E-05 | 9.97E-05 | Yes | Up | - |
| O60292 | SIPA1L3 | 55/114(48.25) | 0.61 | 5.26E-08 | 3.81E-07 | Yes | Up | - |
| P35442 | THBS2 | 62/114(54.39) | 0.82 | 8.04E-09 | 7.33E-08 | Yes | Up | - |
| - | C1ORF97 | 60/114(52.63) | 0.42 | 2.24E-05 | 8.24E-05 | Yes | Up | - |
| I3L153 | NAT15 | 66/114(57.89) | 0.4 | 9.69E-13 | 3.13E-11 | Yes | Up | - |
| O75150 | RNF40 | 63/114(55.26) | 0.41 | 3.97E-10 | 5.37E-09 | Yes | Up | - |
| Q8NFJ5 | GPRC5A | 60/114(52.63) | 1.44 | 2.79E-15 | 2.63E-13 | Yes | Up | - |
| Q9HD67 | MYO10 | 64/114(56.14) | 0.22 | 1.95E-02 | 3.52E-02 | Yes | Up | - |
| Q6DKI2 | LGALS9C | 60/114(52.63) | 0.64 | 1.61E-05 | 6.15E-05 | Yes | Up | - |
| Q6ZNW5 | C15ORF58 | 59/114(51.75) | 0.31 | 6.15E-05 | 2.04E-04 | Yes | Up | - |
| O14976 | GAK | 63/114(55.26) | 0.3 | 1.40E-06 | 6.90E-06 | Yes | Up | - |
| Q9BRX9 | WDR83 | 72/114(63.16) | 0.39 | 2.05E-06 | 9.74E-06 | Yes | Up | - |
| P14091 | CTSE | 50/114(43.86) | 0.94 | 9.85E-04 | 2.43E-03 | Yes | Up | - |
| A1A5D9 | CCDC64B | 65/114(57.02) | 0.46 | 1.40E-02 | 2.61E-02 | Yes | Up | - |
| Q9H0E3 | SAP130 | 61/114(53.51) | 0.17 | 7.90E-05 | 2.55E-04 | Yes | Up | - |
| Q9Y6C2 | EMILIN1 | 64/114(56.14) | 0.3 | 1.52E-02 | 2.82E-02 | Yes | Up | - |
| O00746 | NME4 | 57/114(50.0) | 0.46 | 1.84E-07 | 1.15E-06 | Yes | Up | - |
| Q8N4B1 | FAM109A | 68/114(59.65) | 0.29 | 2.08E-05 | 7.70E-05 | Yes | Up | - |
| P36941 | LTBR | 61/114(53.51) | 0.19 | 8.18E-04 | 2.07E-03 | Yes | Up | - |
| Q9ULJ7 | ANKRD50 | 55/114(48.25) | 0.43 | 1.68E-05 | 6.39E-05 | Yes | Up | - |
| O15392 | BIRC5 | 61/114(53.51) | 1.64 | 3.19E-13 | 1.24E-11 | Yes | Up | - |
| Q9NZ81 | PRR13 | 60/114(52.63) | 0.42 | 1.27E-09 | 1.48E-08 | Yes | Up | - |
| Q9BZD6 | PRRG4 | 54/114(47.37) | 0.5 | 2.22E-04 | 6.42E-04 | Yes | Up | - |
| Q9HCC0 | MCCC2 | 53/114(46.49) | 0.5 | 1.24E-06 | 6.22E-06 | Yes | Up | - |
| P12107 | COL11A1 | 59/114(51.75) | 2.69 | 1.66E-20 | 9.14E-18 | Yes | Up | - |
| A8MZ36 | EVPLL | 56/114(49.12) | 0.44 | 2.82E-02 | 4.88E-02 | Yes | Up | - |
| Q96S16 | JMJD8 | 64/114(56.14) | 0.3 | 9.83E-07 | 5.05E-06 | Yes | Up | - |
| Q9Y5U5 | TNFRSF18 | 59/114(51.75) | 1.59 | 1.68E-22 | 2.50E-19 | Yes | Up | - |
| Q9H1R3 | MYLK2 | 63/114(55.26) | 1.07 | 3.34E-06 | 1.50E-05 | Yes | Up | - |
| Q6VB85 | FOXD4L2 | 56/114(49.12) | 0.42 | 7.77E-03 | 1.55E-02 | Yes | Up | - |
| Q8WYR4 | RSPH1 | 65/114(57.02) | 1.01 | 9.90E-08 | 6.66E-07 | Yes | Up | - |
| - | PA2G4P4 | 60/114(52.63) | 0.15 | 9.64E-03 | 1.88E-02 | Yes | Up | - |
| P60602 | ROMO1 | 64/114(56.14) | 0.49 | 9.37E-08 | 6.33E-07 | Yes | Up | - |
| Q9Y5Y5 | PEX16 | 60/114(52.63) | 0.3 | 2.71E-05 | 9.77E-05 | Yes | Up | - |
| O95229 | ZWINT | 60/114(52.63) | 1.3 | 4.45E-14 | 2.51E-12 | Yes | Up | - |
| P50570 | DNM2 | 64/114(56.14) | 0.26 | 1.73E-05 | 6.55E-05 | Yes | Up | - |
| P50454 | SERPINH1 | 57/114(50.0) | 0.42 | 5.39E-05 | 1.81E-04 | Yes | Up | - |
| Q9UFC0 | LRWD1 | 65/114(57.02) | 0.56 | 1.99E-10 | 2.95E-09 | Yes | Up | - |
| - | AOX2P | 38/114(33.33) | 0.77 | 6.78E-03 | 1.37E-02 | Yes | Up | - |
| Q8NAJ2 | C9ORF106 | 58/114(50.88) | 0.69 | 3.86E-04 | 1.05E-03 | Yes | Up | - |
| Q9H0P0 | NT5C3 | 60/114(52.63) | 0.23 | 5.77E-04 | 1.51E-03 | Yes | Up | - |
| O43763 | TLX2 | 37/114(32.46) | 0.67 | 2.01E-02 | 3.61E-02 | Yes | Up | - |
| Q8N6L1 | KRTCAP2 | 62/114(54.39) | 0.42 | 1.45E-06 | 7.14E-06 | Yes | Up | - |
| Q8TD22 | SFXN5 | 64/114(56.14) | 0.62 | 2.59E-07 | 1.56E-06 | Yes | Up | - |
| Q96L34 | MARK4 | 65/114(57.02) | 0.24 | 2.87E-05 | 1.03E-04 | Yes | Up | - |
| - | LOC80154 | 63/114(55.26) | 0.33 | 1.70E-03 | 3.98E-03 | Yes | Up | - |
| Q96MW1 | CCDC43 | 60/114(52.63) | 0.3 | 2.01E-05 | 7.48E-05 | Yes | Up | - |
| Q9UKP4 | ADAMTS7 | 58/114(50.88) | 0.6 | 1.08E-05 | 4.31E-05 | Yes | Up | - |
| Q9HD42 | CHMP1A | 60/114(52.63) | 0.19 | 1.24E-03 | 2.99E-03 | Yes | Up | - |
| Q9H3M0 | KCNF1 | 59/114(51.75) | 1.54 | 2.20E-13 | 9.04E-12 | Yes | Up | - |
| Q9NQT5 | EXOSC3 | 65/114(57.02) | 0.4 | 1.28E-09 | 1.48E-08 | Yes | Up | - |
| P24001 | IL32 | 70/114(61.4) | 0.37 | 2.49E-03 | 5.58E-03 | Yes | Up | - |
| O60548 | FOXD2 | 55/114(48.25) | 0.94 | 4.98E-11 | 8.99E-10 | Yes | Up | - |
| Q9UKT4 | FBXO5 | 56/114(49.12) | 0.32 | 9.70E-04 | 2.40E-03 | Yes | Up | - |
| O95947 | TBX6 | 63/114(55.26) | 0.27 | 6.86E-03 | 1.39E-02 | Yes | Up | - |
| Q8TBX8 | PIP4K2C | 52/114(45.61) | 0.51 | 2.21E-09 | 2.36E-08 | Yes | Up | - |
| Q9UJY5 | GGA1 | 67/114(58.77) | 0.17 | 2.73E-03 | 6.05E-03 | Yes | Up | - |
| O14523 | C2CD2L | 55/114(48.25) | 0.4 | 3.86E-07 | 2.22E-06 | Yes | Up | - |
| Q7Z5J4 | RAI1 | 58/114(50.88) | 0.22 | 3.62E-03 | 7.79E-03 | Yes | Up | - |
| O60934 | NBN | 50/114(43.86) | 0.24 | 1.23E-03 | 2.97E-03 | Yes | Up | - |
| P51690 | ARSE | 60/114(52.63) | 0.58 | 3.65E-04 | 1.00E-03 | Yes | Up | - |
| Q8IWT0 | ZBTB8OS | 64/114(56.14) | 0.19 | 2.30E-03 | 5.20E-03 | Yes | Up | - |
| Q96IM9 | DYDC2 | 61/114(53.51) | 0.81 | 2.07E-05 | 7.70E-05 | Yes | Up | - |
| O15391 | YY2 | 52/114(45.61) | 0.29 | 1.39E-03 | 3.32E-03 | Yes | Up | - |
| - | C4ORF42 | 60/114(52.63) | 0.16 | 6.24E-03 | 1.27E-02 | Yes | Up | - |
| Q96L12 | CALR3 | 35/114(30.7) | 1.02 | 5.70E-04 | 1.49E-03 | Yes | Up | - |
| P06127 | CD5 | 60/114(52.63) | 0.54 | 2.26E-04 | 6.51E-04 | Yes | Up | - |
| Q86SG6 | NEK8 | 58/114(50.88) | 0.36 | 3.35E-04 | 9.26E-04 | Yes | Up | - |
| Q96MY1 | C20ORF112 | 57/114(50.0) | 0.25 | 7.47E-04 | 1.91E-03 | Yes | Up | - |
| Q96CP2 | FLYWCH2 | 70/114(61.4) | 0.6 | 1.42E-12 | 4.32E-11 | Yes | Up | - |
| Q8N371 | JMJD5 | 58/114(50.88) | 0.16 | 6.73E-03 | 1.36E-02 | Yes | Up | - |
| Q9BXT2 | CACNG6 | 55/114(48.25) | 1.32 | 4.45E-07 | 2.51E-06 | Yes | Up | - |
| Q15517 | CDSN | 66/114(57.89) | 0.93 | 9.44E-07 | 4.88E-06 | Yes | Up | - |
| Q5T036 | FAM120AOS | 51/114(44.74) | 0.12 | 2.53E-02 | 4.44E-02 | Yes | Up | - |
| Q99873 | PRMT1 | 67/114(58.77) | 0.24 | 6.62E-05 | 2.17E-04 | Yes | Up | - |
| Q8TD57 | DNAH3 | 52/114(45.61) | 0.43 | 2.35E-02 | 4.14E-02 | Yes | Up | - |
| Q9UPX6 | KIAA1024 | 58/114(50.88) | 0.42 | 1.95E-03 | 4.50E-03 | Yes | Up | - |
| Q6P4A7 | SFXN4 | 58/114(50.88) | 0.19 | 8.91E-04 | 2.23E-03 | Yes | Up | - |
| Q13515 | BFSP2 | 61/114(53.51) | 0.76 | 3.52E-04 | 9.69E-04 | Yes | Up | - |
| O94761 | RECQL4 | 61/114(53.51) | 1.34 | 1.99E-14 | 1.31E-12 | Yes | Up | - |
| B3EWG3 | FAM25A | 35/114(30.7) | 1.44 | 2.08E-04 | 6.05E-04 | Yes | Up | - |
| Q86VE0 | MYPOP | 62/114(54.39) | 0.27 | 8.10E-06 | 3.32E-05 | Yes | Up | - |
| Q9UKN5 | PRDM4 | 50/114(43.86) | 0.1 | 2.23E-02 | 3.97E-02 | Yes | Up | - |
| Q9UKP5 | ADAMTS6 | 64/114(56.14) | 0.72 | 4.16E-07 | 2.36E-06 | Yes | Up | - |
| P35244 | RPA3 | 63/114(55.26) | 0.43 | 2.23E-08 | 1.81E-07 | Yes | Up | - |
| Q9BU64 | CENPO | 56/114(49.12) | 0.6 | 5.27E-07 | 2.92E-06 | Yes | Up | - |
| Q9NPB0 | C6ORF64 | 54/114(47.37) | 0.23 | 1.06E-03 | 2.61E-03 | Yes | Up | - |
| Q96CC6 | RHBDF1 | 61/114(53.51) | 0.34 | 7.41E-07 | 3.94E-06 | Yes | Up | - |
| A9QM74 | KPNA7 | 54/114(47.37) | 1.29 | 3.00E-07 | 1.76E-06 | Yes | Up | - |
| P30203 | CD6 | 59/114(51.75) | 0.38 | 3.50E-03 | 7.55E-03 | Yes | Up | - |
| Q14534 | SQLE | 56/114(49.12) | 1.04 | 7.20E-10 | 8.96E-09 | Yes | Up | - |
| Q9Y230 | RUVBL2 | 67/114(58.77) | 0.43 | 1.38E-07 | 8.92E-07 | Yes | Up | - |
| Q674R7 | ATG9B | 55/114(48.25) | 0.61 | 3.67E-05 | 1.28E-04 | Yes | Up | - |
| P49915 | GMPS | 58/114(50.88) | 0.4 | 2.58E-06 | 1.19E-05 | Yes | Up | - |
| P84074 | HPCA | 72/114(63.16) | 0.48 | 1.84E-03 | 4.27E-03 | Yes | Up | - |
| Q86XN8 | MEX3D | 67/114(58.77) | 0.41 | 1.95E-08 | 1.61E-07 | Yes | Up | - |
| Q9NRX4 | PHPT1 | 68/114(59.65) | 0.34 | 4.80E-05 | 1.63E-04 | Yes | Up | - |
| Q96EX2 | RNFT2 | 59/114(51.75) | 0.99 | 7.20E-12 | 1.74E-10 | Yes | Up | - |
| Q67FW5 | B3GNTL1 | 62/114(54.39) | 0.33 | 4.87E-05 | 1.65E-04 | Yes | Up | - |
| Q86XP0 | PLA2G4D | 50/114(43.86) | 1.11 | 2.74E-06 | 1.26E-05 | Yes | Up | - |
| O94832 | MYO1D | 58/114(50.88) | 0.35 | 6.59E-06 | 2.75E-05 | Yes | Up | - |
| Q9BX40 | LSM14B | 64/114(56.14) | 0.4 | 1.64E-07 | 1.04E-06 | Yes | Up | - |
| P35716 | SOX11 | 61/114(53.51) | 0.88 | 2.91E-05 | 1.04E-04 | Yes | Up | - |
| Q9UK76 | HN1 | 63/114(55.26) | 1.03 | 4.93E-13 | 1.79E-11 | Yes | Up | - |
| Q96H55 | MYO19 | 57/114(50.0) | 0.51 | 1.94E-07 | 1.20E-06 | Yes | Up | - |
| P30043 | BLVRB | 64/114(56.14) | 0.28 | 5.62E-03 | 1.16E-02 | Yes | Up | - |
| Q9NVI7 | ATAD3A | 59/114(51.75) | 0.42 | 2.79E-05 | 1.00E-04 | Yes | Up | - |
| Q9UBU7 | DBF4 | 55/114(48.25) | 0.55 | 3.24E-07 | 1.89E-06 | Yes | Up | - |
| Q9UL12 | SARDH | 66/114(57.89) | 0.37 | 9.36E-03 | 1.83E-02 | Yes | Up | - |
| Q9BUL9 | RPP25 | 62/114(54.39) | 0.3 | 5.20E-03 | 1.08E-02 | Yes | Up | - |
| - | GTF2H2B | 62/114(54.39) | 0.57 | 5.84E-04 | 1.53E-03 | Yes | Up | - |
| Q8N6T3 | ARFGAP1 | 67/114(58.77) | 0.46 | 6.57E-10 | 8.31E-09 | Yes | Up | - |
| Q8WXH2 | JPH3 | 56/114(49.12) | 0.62 | 2.45E-04 | 7.00E-04 | Yes | Up | - |
| Q99627 | COPS8 | 58/114(50.88) | 0.14 | 1.33E-02 | 2.50E-02 | Yes | Up | - |
| Q6PCD5 | RFWD3 | 57/114(50.0) | 0.22 | 4.96E-03 | 1.04E-02 | Yes | Up | - |
| P51681 | CCR5 | 58/114(50.88) | 0.45 | 1.60E-04 | 4.80E-04 | Yes | Up | - |
| P18858 | LIG1 | 64/114(56.14) | 0.59 | 6.24E-08 | 4.43E-07 | Yes | Up | - |
| Q92911 | SLC5A5 | 60/114(52.63) | 0.62 | 2.95E-03 | 6.48E-03 | Yes | Up | - |
| Q9UPP1 | PHF8 | 61/114(53.51) | 0.15 | 1.33E-02 | 2.51E-02 | Yes | Up | - |
| Q13098 | GPS1 | 61/114(53.51) | 0.25 | 3.27E-04 | 9.07E-04 | Yes | Up | - |
| Q9Y5B0 | CTDP1 | 72/114(63.16) | 0.17 | 8.65E-04 | 2.17E-03 | Yes | Up | - |
| - | FBXO22OS | 59/114(51.75) | 0.48 | 3.13E-08 | 2.42E-07 | Yes | Up | - |
| Q8NFF5 | FLAD1 | 61/114(53.51) | 0.58 | 1.64E-10 | 2.49E-09 | Yes | Up | - |
| O15354 | GPR37 | 59/114(51.75) | 0.5 | 1.91E-04 | 5.62E-04 | Yes | Up | - |
| Q8N149 | LILRA2 | 58/114(50.88) | 0.35 | 4.90E-03 | 1.02E-02 | Yes | Up | - |
| Q8N7C3 | TRIML2 | 37/114(32.46) | 1.3 | 2.63E-05 | 9.50E-05 | Yes | Up | - |
| Q8N715 | C1ORF65 | 54/114(47.37) | 1.05 | 4.97E-05 | 1.68E-04 | Yes | Up | - |
| Q6ZT21 | TMPPE | 50/114(43.86) | 0.18 | 2.32E-02 | 4.10E-02 | Yes | Up | - |
| Q9Y2Y4 | ZBTB32 | 59/114(51.75) | 0.4 | 4.54E-03 | 9.57E-03 | Yes | Up | - |
| Q8IUR0 | TRAPPC5 | 68/114(59.65) | 0.41 | 5.04E-07 | 2.81E-06 | Yes | Up | - |
| P23528 | CFL1 | 63/114(55.26) | 0.5 | 2.11E-09 | 2.27E-08 | Yes | Up | - |
| Q16611 | BAK1 | 69/114(60.53) | 0.49 | 3.00E-08 | 2.33E-07 | Yes | Up | - |
| Q8N0Z8 | PUSL1 | 60/114(52.63) | 0.64 | 6.87E-11 | 1.18E-09 | Yes | Up | - |
| Q9H6T0 | ESRP2 | 55/114(48.25) | 0.45 | 2.18E-05 | 8.05E-05 | Yes | Up | - |
| Q9P244 | LRFN1 | 57/114(50.0) | 0.58 | 1.84E-06 | 8.83E-06 | Yes | Up | - |
| Q9NVR5 | C14ORF104 | 52/114(45.61) | 0.13 | 2.18E-02 | 3.89E-02 | Yes | Up | - |
| Q96K31 | C8ORF76 | 64/114(56.14) | 0.37 | 1.31E-05 | 5.11E-05 | Yes | Up | - |
| Q9Y3E2 | BOLA1 | 59/114(51.75) | 0.37 | 1.98E-06 | 9.44E-06 | Yes | Up | - |
| Q12965 | MYO1E | 59/114(51.75) | 0.14 | 2.55E-02 | 4.47E-02 | Yes | Up | - |
| - | PMS2L2 | 56/114(49.12) | 0.22 | 5.10E-03 | 1.06E-02 | Yes | Up | - |
| Q14145 | KEAP1 | 61/114(53.51) | 0.32 | 5.94E-07 | 3.25E-06 | Yes | Up | - |
| B7Z1M9 | C2CD4D | 56/114(49.12) | 0.54 | 1.94E-04 | 5.70E-04 | Yes | Up | - |
| O43598 | C6ORF108 | 62/114(54.39) | 0.28 | 2.50E-03 | 5.59E-03 | Yes | Up | - |
| Q9H4K1 | RIBC2 | 65/114(57.02) | 1.11 | 4.55E-11 | 8.34E-10 | Yes | Up | - |
| Q93079 | HIST1H2BH | 61/114(53.51) | 1.6 | 1.22E-13 | 5.67E-12 | Yes | Up | - |
| Q59FA2 | SFRS1 | 55/114(48.25) | 0.23 | 5.17E-05 | 1.74E-04 | Yes | Up | - |
| Q9Y6G3 | MRPL42 | 61/114(53.51) | 0.4 | 1.64E-07 | 1.04E-06 | Yes | Up | - |
| P47224 | RABIF | 61/114(53.51) | 0.69 | 6.83E-12 | 1.67E-10 | Yes | Up | - |
| Q96DE9 | CXORF40B | 61/114(53.51) | 0.42 | 2.66E-10 | 3.76E-09 | Yes | Up | - |
| Q96M63 | CCDC114 | 51/114(44.74) | 0.52 | 3.14E-04 | 8.75E-04 | Yes | Up | - |
| Q96G27 | WBP1 | 64/114(56.14) | 0.25 | 3.01E-06 | 1.37E-05 | Yes | Up | - |
| Q6N075 | MFSD5 | 60/114(52.63) | 0.4 | 4.39E-11 | 8.09E-10 | Yes | Up | - |
| P05496 | ATP5G1 | 56/114(49.12) | 0.28 | 2.07E-03 | 4.75E-03 | Yes | Up | - |
| P06454 | PTMA | 65/114(57.02) | 0.25 | 1.25E-04 | 3.84E-04 | Yes | Up | - |
| P04217 | A1BG | 66/114(57.89) | 0.62 | 5.31E-08 | 3.84E-07 | Yes | Up | - |
| Q6P2M8 | PNCK | 69/114(60.53) | 1.19 | 1.49E-08 | 1.26E-07 | Yes | Up | - |
| Q8IYJ3 | SYTL1 | 56/114(49.12) | 0.32 | 2.79E-02 | 4.84E-02 | Yes | Up | - |
| - | RNF216L | 55/114(48.25) | 0.11 | 7.80E-03 | 1.56E-02 | Yes | Up | - |
| O15405 | TOX3 | 60/114(52.63) | 0.88 | 9.00E-05 | 2.86E-04 | Yes | Up | - |
| P23921 | RRM1 | 54/114(47.37) | 0.22 | 3.10E-03 | 6.77E-03 | Yes | Up | - |
| Q8WW14 | C10ORF82 | 52/114(45.61) | 0.64 | 1.10E-02 | 2.11E-02 | Yes | Up | - |
| Q12981 | BNIP1 | 65/114(57.02) | 0.19 | 2.75E-04 | 7.75E-04 | Yes | Up | - |
| P22674 | CCNO | 59/114(51.75) | 1.16 | 1.94E-08 | 1.61E-07 | Yes | Up | - |
| Q9UFG5 | C19ORF25 | 65/114(57.02) | 0.36 | 1.44E-06 | 7.12E-06 | Yes | Up | - |
| Q9P287 | BCCIP | 64/114(56.14) | 0.31 | 7.40E-07 | 3.93E-06 | Yes | Up | - |
| Q9H707 | ZNF552 | 59/114(51.75) | 0.77 | 2.28E-07 | 1.39E-06 | Yes | Up | - |
| Q96ER9 | CCDC51 | 59/114(51.75) | 0.31 | 3.47E-05 | 1.22E-04 | Yes | Up | - |
| Q9H6R4 | NOL6 | 58/114(50.88) | 0.17 | 2.01E-03 | 4.62E-03 | Yes | Up | - |
| Q96EI5 | TCEAL4 | 62/114(54.39) | 0.29 | 4.15E-04 | 1.12E-03 | Yes | Up | - |
| P55265 | ADAR | 60/114(52.63) | 0.5 | 9.54E-10 | 1.15E-08 | Yes | Up | - |
| P35270 | SPR | 65/114(57.02) | 0.5 | 1.35E-08 | 1.16E-07 | Yes | Up | - |
| Q99961 | SH3GL1 | 66/114(57.89) | 0.32 | 7.22E-07 | 3.85E-06 | Yes | Up | - |
| Q8N9Z0 | ZNF610 | 61/114(53.51) | 0.36 | 2.42E-04 | 6.92E-04 | Yes | Up | - |
| Q9BSD3 | C12ORF32 | 55/114(48.25) | 0.56 | 3.67E-10 | 5.02E-09 | Yes | Up | - |
| Q9NRR5 | UBQLN4 | 56/114(49.12) | 0.47 | 2.21E-08 | 1.80E-07 | Yes | Up | - |
| Q7L945 | ZNF627 | 56/114(49.12) | 0.28 | 1.70E-04 | 5.07E-04 | Yes | Up | - |
| Q99988 | GDF15 | 61/114(53.51) | 1.16 | 1.15E-11 | 2.57E-10 | Yes | Up | - |
| Q9BTX3 | TMEM208 | 65/114(57.02) | 0.41 | 1.78E-08 | 1.49E-07 | Yes | Up | - |
| Q6NSI8 | KIAA1841 | 56/114(49.12) | 0.22 | 4.98E-03 | 1.04E-02 | Yes | Up | - |
| Q6PIF2 | SYCE2 | 56/114(49.12) | 0.83 | 6.67E-07 | 3.59E-06 | Yes | Up | - |
| Q9ULB5 | CDH7 | 53/114(46.49) | 1.69 | 2.72E-09 | 2.84E-08 | Yes | Up | - |
| Q9H479 | FN3K | 58/114(50.88) | 0.29 | 3.64E-04 | 9.99E-04 | Yes | Up | - |
| P09874 | PARP1 | 60/114(52.63) | 0.63 | 1.75E-10 | 2.64E-09 | Yes | Up | - |
| O94777 | DPM2 | 63/114(55.26) | 0.51 | 7.69E-11 | 1.30E-09 | Yes | Up | - |
| O43242 | PSMD3 | 65/114(57.02) | 0.35 | 2.55E-04 | 7.28E-04 | Yes | Up | - |
| Q9Y5K8 | ATP6V1D | 60/114(52.63) | 0.15 | 5.31E-03 | 1.10E-02 | Yes | Up | - |
| - | PMS2L5 | 64/114(56.14) | 0.2 | 2.02E-03 | 4.64E-03 | Yes | Up | - |
| Q6NXT1 | ANKRD54 | 58/114(50.88) | 0.27 | 2.19E-07 | 1.34E-06 | Yes | Up | - |
| E0CX11 | PL-5283 | 55/114(48.25) | 0.25 | 2.89E-04 | 8.11E-04 | Yes | Up | - |
| B7Z9G5 | C1ORF58 | 56/114(49.12) | 0.31 | 1.28E-03 | 3.08E-03 | Yes | Up | - |
| Q8N129 | CNPY4 | 60/114(52.63) | 0.21 | 5.57E-04 | 1.47E-03 | Yes | Up | - |
| O95965 | ITGBL1 | 56/114(49.12) | 0.57 | 8.90E-07 | 4.64E-06 | Yes | Up | - |
| P49447 | CYB561 | 56/114(49.12) | 0.72 | 8.01E-10 | 9.82E-09 | Yes | Up | - |
| Q8N271 | PROM2 | 57/114(50.0) | 0.51 | 7.37E-03 | 1.48E-02 | Yes | Up | - |
| Q8N1N4 | KRT78 | 32/114(28.07) | 1.15 | 1.16E-02 | 2.21E-02 | Yes | Up | - |
| Q6UX72 | B3GNT9 | 63/114(55.26) | 0.22 | 8.62E-03 | 1.70E-02 | Yes | Up | - |
| Q9BRQ3 | NUDT22 | 69/114(60.53) | 0.35 | 2.80E-05 | 1.00E-04 | Yes | Up | - |
| O43715 | TRIAP1 | 47/114(41.23) | 0.15 | 2.66E-03 | 5.92E-03 | Yes | Up | - |
| Q6NSI3 | FAM53A | 59/114(51.75) | 0.37 | 8.44E-05 | 2.70E-04 | Yes | Up | - |
| Q9GZN4 | PRSS22 | 64/114(56.14) | 0.84 | 6.64E-06 | 2.77E-05 | Yes | Up | - |
| Q6IQ16 | SPOPL | 56/114(49.12) | 0.27 | 3.61E-03 | 7.77E-03 | Yes | Up | - |
| Q8TBC3 | SHKBP1 | 64/114(56.14) | 0.44 | 3.67E-09 | 3.70E-08 | Yes | Up | - |
| Q15910 | EZH2 | 57/114(50.0) | 1.16 | 1.41E-12 | 4.28E-11 | Yes | Up | - |
| P55001 | MFAP2 | 64/114(56.14) | 1.51 | 5.28E-21 | 3.97E-18 | Yes | Up | - |
| P09683 | SCT | 57/114(50.0) | 2.19 | 8.74E-13 | 2.87E-11 | Yes | Up | - |
| Q12815 | TROAP | 60/114(52.63) | 1.79 | 3.61E-16 | 4.94E-14 | Yes | Up | - |
| Q8NDD1 | C1ORF131 | 60/114(52.63) | 0.43 | 4.68E-09 | 4.58E-08 | Yes | Up | - |
| P45974 | USP5 | 60/114(52.63) | 0.38 | 9.92E-08 | 6.66E-07 | Yes | Up | - |
| P15104 | GLUL | 55/114(48.25) | 0.23 | 2.80E-02 | 4.85E-02 | Yes | Up | - |
| Q9NYZ3 | GTSE1 | 59/114(51.75) | 1.4 | 1.37E-12 | 4.20E-11 | Yes | Up | - |
| Q9NZB2 | FAM120A | 52/114(45.61) | 0.21 | 2.44E-04 | 6.97E-04 | Yes | Up | - |
| Q9GZX3 | CHST6 | 64/114(56.14) | 1.1 | 6.60E-10 | 8.33E-09 | Yes | Up | - |
| Q01664 | TFAP4 | 60/114(52.63) | 0.25 | 2.14E-03 | 4.88E-03 | Yes | Up | - |
| - | LOC100128191 | 57/114(50.0) | 0.63 | 1.98E-07 | 1.23E-06 | Yes | Up | - |
| Q6P2E9 | EDC4 | 59/114(51.75) | 0.13 | 5.70E-03 | 1.17E-02 | Yes | Up | - |
| Q86W42 | THOC6 | 66/114(57.89) | 0.6 | 6.27E-13 | 2.19E-11 | Yes | Up | - |
| P43246 | MSH2 | 55/114(48.25) | 0.23 | 3.73E-03 | 8.00E-03 | Yes | Up | - |
| Q6DWJ6 | GPR139 | 37/114(32.46) | 1.39 | 1.67E-04 | 4.99E-04 | Yes | Up | - |
| Q9H808 | TLE6 | 60/114(52.63) | 0.96 | 3.21E-08 | 2.48E-07 | Yes | Up | - |
| O00400 | SLC33A1 | 54/114(47.37) | 0.17 | 8.47E-03 | 1.67E-02 | Yes | Up | - |
| - | DGCR10 | 44/114(38.6) | 0.57 | 1.10E-02 | 2.12E-02 | Yes | Up | - |
| Q9NX52 | RHBDL2 | 66/114(57.89) | 0.6 | 6.41E-07 | 3.46E-06 | Yes | Up | - |
| P04155 | TFF1 | 64/114(56.14) | 1.57 | 3.74E-08 | 2.82E-07 | Yes | Up | - |
| Q6IA17 | SIGIRR | 65/114(57.02) | 0.57 | 1.05E-11 | 2.42E-10 | Yes | Up | - |
| Q96DD7 | SHISA4 | 65/114(57.02) | 0.27 | 3.56E-03 | 7.68E-03 | Yes | Up | - |
| P53384 | NUBP1 | 68/114(59.65) | 0.31 | 2.61E-09 | 2.73E-08 | Yes | Up | - |
| Q9P0V8 | SLAMF8 | 63/114(55.26) | 0.8 | 1.09E-08 | 9.57E-08 | Yes | Up | - |
| Q8IYS4 | C16ORF71 | 60/114(52.63) | 0.99 | 2.58E-10 | 3.68E-09 | Yes | Up | - |
| A0AVK6 | E2F8 | 59/114(51.75) | 1.3 | 1.40E-08 | 1.19E-07 | Yes | Up | - |
| Q96E35 | ZMYND19 | 61/114(53.51) | 0.53 | 5.40E-10 | 7.04E-09 | Yes | Up | - |
| Q9H444 | CHMP4B | 68/114(59.65) | 0.32 | 2.04E-07 | 1.26E-06 | Yes | Up | - |
| Q9H3Q3 | GAL3ST2 | 55/114(48.25) | 2.44 | 3.20E-17 | 6.80E-15 | Yes | Up | - |
| Q8IZP7 | HS6ST3 | 55/114(48.25) | 1.23 | 1.11E-07 | 7.35E-07 | Yes | Up | - |
| Q9P0P0 | RNF181 | 65/114(57.02) | 0.33 | 5.15E-07 | 2.86E-06 | Yes | Up | - |
| O43502 | RAD51C | 58/114(50.88) | 0.25 | 3.33E-04 | 9.21E-04 | Yes | Up | - |
| Q96N28 | SLMO1 | 55/114(48.25) | 0.44 | 9.95E-06 | 4.00E-05 | Yes | Up | - |
| Q13435 | SF3B2 | 62/114(54.39) | 0.18 | 5.83E-04 | 1.52E-03 | Yes | Up | - |
| P29373 | CRABP2 | 57/114(50.0) | 1.15 | 1.51E-11 | 3.24E-10 | Yes | Up | - |
| Q9Y277 | VDAC3 | 60/114(52.63) | 0.29 | 4.31E-05 | 1.48E-04 | Yes | Up | - |
| Q8WXS8 | ADAMTS14 | 62/114(54.39) | 1.91 | 2.23E-18 | 7.42E-16 | Yes | Up | - |
| Q9H633 | RPP21 | 63/114(55.26) | 0.33 | 7.05E-06 | 2.92E-05 | Yes | Up | - |
| - | MGC23270 | 66/114(57.89) | 0.28 | 1.69E-03 | 3.96E-03 | Yes | Up | - |
| Q8N4L1 | TMEM151A | 55/114(48.25) | 0.75 | 3.01E-03 | 6.59E-03 | Yes | Up | - |
| P07360 | C8G | 57/114(50.0) | 0.55 | 6.83E-04 | 1.76E-03 | Yes | Up | - |
| Q6UWM7 | LCTL | 59/114(51.75) | 0.5 | 8.64E-03 | 1.70E-02 | Yes | Up | - |
| P12036 | NEFH | 59/114(51.75) | 0.43 | 1.52E-03 | 3.60E-03 | Yes | Up | - |
| Q9H4H8 | FAM83D | 58/114(50.88) | 1.55 | 4.75E-13 | 1.74E-11 | Yes | Up | - |
| Q6NVH7 | C19ORF39 | 60/114(52.63) | 0.43 | 5.96E-08 | 4.25E-07 | Yes | Up | - |
| Q96NZ8 | WFIKKN1 | 52/114(45.61) | 0.4 | 2.46E-03 | 5.52E-03 | Yes | Up | - |
| Q9HB15 | KCNK12 | 60/114(52.63) | 0.57 | 1.48E-02 | 2.75E-02 | Yes | Up | - |
| Q8N4S9 | MARVELD2 | 58/114(50.88) | 0.59 | 7.90E-05 | 2.55E-04 | Yes | Up | - |
| O15305 | PMM2 | 60/114(52.63) | 0.54 | 4.92E-10 | 6.47E-09 | Yes | Up | - |
| Q96LP2 | FAM81B | 62/114(54.39) | 0.71 | 2.57E-03 | 5.73E-03 | Yes | Up | - |
| Q9BUV8 | C20ORF24 | 61/114(53.51) | 0.47 | 2.88E-07 | 1.71E-06 | Yes | Up | - |
| - | LOC284749 | 62/114(54.39) | 0.75 | 1.96E-04 | 5.75E-04 | Yes | Up | - |
| Q9P2X0 | DPM3 | 68/114(59.65) | 0.35 | 7.31E-05 | 2.38E-04 | Yes | Up | - |
| Q9UBF8 | PI4KB | 46/114(40.35) | 0.15 | 1.60E-03 | 3.77E-03 | Yes | Up | - |
| O60478 | GPR137B | 52/114(45.61) | 0.38 | 2.49E-04 | 7.11E-04 | Yes | Up | - |
| P61018 | RAB4B | 62/114(54.39) | 0.32 | 1.52E-05 | 5.84E-05 | Yes | Up | - |
| Q13477 | MADCAM1 | 54/114(47.37) | 0.48 | 1.33E-02 | 2.49E-02 | Yes | Up | - |
| P21217 | FUT3 | 63/114(55.26) | 0.49 | 2.00E-02 | 3.59E-02 | Yes | Up | - |
| P49368 | CCT3 | 60/114(52.63) | 0.56 | 3.63E-09 | 3.66E-08 | Yes | Up | - |
| Q9H9D4 | ZNF408 | 62/114(54.39) | 0.25 | 1.28E-05 | 5.01E-05 | Yes | Up | - |
| P41440 | SLC19A1 | 55/114(48.25) | 0.36 | 2.72E-04 | 7.70E-04 | Yes | Up | - |
| Q9Y3Q3 | TMED3 | 59/114(51.75) | 0.79 | 1.08E-10 | 1.74E-09 | Yes | Up | - |
| Q9NYS7 | WSB2 | 56/114(49.12) | 0.17 | 2.16E-02 | 3.85E-02 | Yes | Up | - |
| P01374 | LTA | 63/114(55.26) | 0.76 | 1.04E-05 | 4.15E-05 | Yes | Up | - |
| Q63HN8 | RNF213 | 56/114(49.12) | 0.32 | 3.07E-04 | 8.58E-04 | Yes | Up | - |
| Q14978 | NOLC1 | 54/114(47.37) | 0.29 | 6.61E-05 | 2.17E-04 | Yes | Up | - |
| Q9BS16 | CENPK | 57/114(50.0) | 0.98 | 2.43E-08 | 1.95E-07 | Yes | Up | - |
| O96008 | TOMM40 | 67/114(58.77) | 0.51 | 2.95E-08 | 2.29E-07 | Yes | Up | - |
| Q8TCA0 | LRRC20 | 72/114(63.16) | 0.3 | 2.11E-03 | 4.83E-03 | Yes | Up | - |
| Q92674 | CENPI | 58/114(50.88) | 1.23 | 2.48E-10 | 3.55E-09 | Yes | Up | - |
| Q8WXG1 | RSAD2 | 63/114(55.26) | 0.78 | 7.99E-07 | 4.22E-06 | Yes | Up | - |
| O60294 | LCMT2 | 61/114(53.51) | 0.18 | 7.16E-04 | 1.83E-03 | Yes | Up | - |
| Q96P11 | NSUN5 | 66/114(57.89) | 0.39 | 2.68E-08 | 2.11E-07 | Yes | Up | - |
| Q9NP59 | SLC40A1 | 59/114(51.75) | 0.35 | 2.02E-02 | 3.62E-02 | Yes | Up | - |
| Q9BRA2 | TXNDC17 | 63/114(55.26) | 0.45 | 7.63E-08 | 5.30E-07 | Yes | Up | - |
| P51532 | SMARCA4 | 57/114(50.0) | 0.54 | 4.80E-10 | 6.34E-09 | Yes | Up | - |
| O96013 | PAK4 | 61/114(53.51) | 0.66 | 4.97E-09 | 4.82E-08 | Yes | Up | - |
| P21731 | TBXA2R | 68/114(59.65) | 0.35 | 6.76E-04 | 1.74E-03 | Yes | Up | - |
| O94964 | C20ORF117 | 52/114(45.61) | 0.27 | 4.93E-04 | 1.31E-03 | Yes | Up | - |
| Q9Y6K9 | IKBKG | 62/114(54.39) | 0.16 | 7.82E-03 | 1.56E-02 | Yes | Up | - |
| Q14257 | RCN2 | 56/114(49.12) | 0.15 | 2.64E-02 | 4.60E-02 | Yes | Up | - |
| Q6PKC3 | TXNDC11 | 59/114(51.75) | 0.22 | 8.99E-06 | 3.65E-05 | Yes | Up | - |
| P55212 | CASP6 | 60/114(52.63) | 0.5 | 7.77E-11 | 1.31E-09 | Yes | Up | - |
| Q8TEM1 | NUP210 | 57/114(50.0) | 1.04 | 7.21E-11 | 1.23E-09 | Yes | Up | - |
| P15289 | ARSA | 62/114(54.39) | 0.18 | 2.78E-02 | 4.82E-02 | Yes | Up | - |
| Q9BTV5 | FSD1 | 62/114(54.39) | 0.56 | 9.55E-03 | 1.86E-02 | Yes | Up | - |
| Q16790 | CA9 | 55/114(48.25) | 1.02 | 8.21E-04 | 2.07E-03 | Yes | Up | - |
| P40227 | CCT6A | 61/114(53.51) | 0.25 | 1.81E-03 | 4.20E-03 | Yes | Up | - |
| P00918 | CA2 | 67/114(58.77) | 0.44 | 9.36E-03 | 1.83E-02 | Yes | Up | - |
| Q6ZWB5 | C3ORF57 | 56/114(49.12) | 0.64 | 1.65E-03 | 3.87E-03 | Yes | Up | - |
| Q96N20 | ZNF75A | 60/114(52.63) | 0.38 | 4.83E-06 | 2.08E-05 | Yes | Up | - |
| Q96RU7 | TRIB3 | 64/114(56.14) | 0.97 | 1.73E-13 | 7.58E-12 | Yes | Up | - |
| Q9H1B5 | XYLT2 | 62/114(54.39) | 0.31 | 2.07E-05 | 7.69E-05 | Yes | Up | - |
| Q7Z2W9 | MRPL21 | 61/114(53.51) | 0.33 | 4.80E-06 | 2.07E-05 | Yes | Up | - |
| O14950 | MYL12B | 65/114(57.02) | 0.19 | 3.77E-04 | 1.03E-03 | Yes | Up | - |
| Q96NU1 | SAMD11 | 59/114(51.75) | 1.66 | 8.75E-16 | 9.94E-14 | Yes | Up | - |
| P53621 | COPA | 56/114(49.12) | 0.47 | 8.80E-08 | 6.00E-07 | Yes | Up | - |
| O60635 | TSPAN1 | 61/114(53.51) | 1.33 | 1.75E-11 | 3.69E-10 | Yes | Up | - |
| Q86XZ4 | SPATS2 | 56/114(49.12) | 0.44 | 1.07E-07 | 7.11E-07 | Yes | Up | - |
| Q9NWW0 | HCFC1R1 | 73/114(64.04) | 0.41 | 2.10E-06 | 9.93E-06 | Yes | Up | - |
| Q9ULM6 | CNOT6 | 50/114(43.86) | 0.23 | 4.52E-03 | 9.53E-03 | Yes | Up | - |
| Q8WW22 | DNAJA4 | 53/114(46.49) | 0.43 | 3.24E-05 | 1.15E-04 | Yes | Up | - |
| Q6ZSJ8 | C1ORF122 | 61/114(53.51) | 0.31 | 2.78E-04 | 7.84E-04 | Yes | Up | - |
| Q2M2I3 | FAM83E | 51/114(44.74) | 0.61 | 7.10E-03 | 1.43E-02 | Yes | Up | - |
| Q3ZCQ8 | TIMM50 | 60/114(52.63) | 0.16 | 1.44E-02 | 2.69E-02 | Yes | Up | - |
| Q15014 | MORF4L2 | 61/114(53.51) | 0.34 | 1.40E-05 | 5.42E-05 | Yes | Up | - |
| Q8NC69 | KCTD6 | 54/114(47.37) | 0.38 | 1.49E-04 | 4.50E-04 | Yes | Up | - |
| E9PHY8 | HEATR7A | 64/114(56.14) | 0.26 | 9.21E-04 | 2.29E-03 | Yes | Up | - |
| P40926 | MDH2 | 63/114(55.26) | 0.16 | 1.62E-02 | 2.97E-02 | Yes | Up | - |
| Q96C90 | PPP1R14B | 65/114(57.02) | 0.66 | 2.27E-09 | 2.41E-08 | Yes | Up | - |
| P15735 | PHKG2 | 65/114(57.02) | 0.54 | 3.08E-12 | 8.45E-11 | Yes | Up | - |
| P17812 | CTPS | 62/114(54.39) | 0.47 | 4.00E-05 | 1.39E-04 | Yes | Up | - |
| Q16827 | PTPRO | 60/114(52.63) | 0.45 | 2.34E-04 | 6.72E-04 | Yes | Up | - |
| Q6PJG6 | C7ORF27 | 63/114(55.26) | 0.32 | 1.90E-06 | 9.07E-06 | Yes | Up | - |
| Q13505 | MTX1 | 61/114(53.51) | 0.49 | 5.25E-10 | 6.86E-09 | Yes | Up | - |
| Q9Y3B1 | SLMO2 | 55/114(48.25) | 0.28 | 7.68E-04 | 1.95E-03 | Yes | Up | - |
| Q07666 | KHDRBS1 | 55/114(48.25) | 0.1 | 5.26E-03 | 1.09E-02 | Yes | Up | - |
| Q13519 | PNOC | 69/114(60.53) | 0.56 | 1.92E-03 | 4.44E-03 | Yes | Up | - |
| Q15005 | SPCS2 | 54/114(47.37) | 0.24 | 2.73E-04 | 7.71E-04 | Yes | Up | - |
| Q9Y6H1 | CHCHD2 | 69/114(60.53) | 0.3 | 2.79E-05 | 1.00E-04 | Yes | Up | - |
| Q8N490 | PNKD | 69/114(60.53) | 0.43 | 1.04E-06 | 5.32E-06 | Yes | Up | - |
| Q6IQ19 | C1ORF96 | 59/114(51.75) | 0.29 | 1.03E-03 | 2.54E-03 | Yes | Up | - |
| O15116 | LSM1 | 67/114(58.77) | 0.35 | 2.23E-05 | 8.21E-05 | Yes | Up | - |
| Q9BUJ2 | HNRNPUL1 | 55/114(48.25) | 0.11 | 7.28E-03 | 1.46E-02 | Yes | Up | - |
| Q99880 | HIST1H2BL | 52/114(45.61) | 1.11 | 1.10E-06 | 5.56E-06 | Yes | Up | - |
| O43184 | ADAM12 | 56/114(49.12) | 0.75 | 6.12E-06 | 2.57E-05 | Yes | Up | - |
| Q9H0R5 | GBP3 | 56/114(49.12) | 0.28 | 1.58E-03 | 3.71E-03 | Yes | Up | - |
| Q07960 | ARHGAP1 | 56/114(49.12) | 0.17 | 1.88E-03 | 4.35E-03 | Yes | Up | - |
| O95873 | C6ORF47 | 68/114(59.65) | 0.2 | 2.24E-04 | 6.47E-04 | Yes | Up | - |
| P63215 | GNG3 | 61/114(53.51) | 0.7 | 4.14E-06 | 1.81E-05 | Yes | Up | - |
| O14896 | IRF6 | 57/114(50.0) | 0.38 | 9.95E-03 | 1.93E-02 | Yes | Up | - |
| P61619 | SEC61A1 | 60/114(52.63) | 0.29 | 4.41E-06 | 1.92E-05 | Yes | Up | - |
| Q66K89 | E4F1 | 63/114(55.26) | 0.44 | 3.79E-08 | 2.85E-07 | Yes | Up | - |
| Q96DY7 | MTBP | 61/114(53.51) | 0.49 | 5.40E-05 | 1.81E-04 | Yes | Up | - |
| A4QMS7 | C5ORF49 | 59/114(51.75) | 0.9 | 6.51E-08 | 4.60E-07 | Yes | Up | - |
| Q00597 | FANCC | 54/114(47.37) | 0.15 | 2.50E-02 | 4.38E-02 | Yes | Up | - |
| P22607 | FGFR3 | 62/114(54.39) | 1.23 | 6.65E-14 | 3.42E-12 | Yes | Up | - |
| Q15843 | NEDD8 | 64/114(56.14) | 0.15 | 4.85E-03 | 1.02E-02 | Yes | Up | - |
| P07738 | BPGM | 56/114(49.12) | 0.15 | 1.12E-02 | 2.15E-02 | Yes | Up | - |
| Q8IYF3 | TEX11 | 64/114(56.14) | 0.75 | 1.94E-03 | 4.48E-03 | Yes | Up | - |
| Q9P258 | RCC2 | 57/114(50.0) | 0.56 | 3.72E-11 | 7.05E-10 | Yes | Up | - |
| Q9UKD2 | MRTO4 | 60/114(52.63) | 0.18 | 8.33E-04 | 2.10E-03 | Yes | Up | - |
| Q9BUN1 | C1ORF56 | 63/114(55.26) | 0.39 | 1.93E-06 | 9.18E-06 | Yes | Up | - |
| Q96KA5 | CLPTM1L | 61/114(53.51) | 0.18 | 2.94E-03 | 6.46E-03 | Yes | Up | - |
| Q9H999 | PANK3 | 54/114(47.37) | 0.26 | 1.81E-02 | 3.29E-02 | Yes | Up | - |
| Q9C0B2 | KIAA1751 | 62/114(54.39) | 0.5 | 9.77E-03 | 1.90E-02 | Yes | Up | - |
| Q6UX07 | DHRS13 | 54/114(47.37) | 0.71 | 5.93E-11 | 1.05E-09 | Yes | Up | - |
| P60891 | PRPS1 | 60/114(52.63) | 0.2 | 5.54E-03 | 1.14E-02 | Yes | Up | - |
| Q5SQN1 | SNAP47 | 61/114(53.51) | 0.37 | 2.25E-08 | 1.83E-07 | Yes | Up | - |
| Q96D70 | C19ORF22 | 62/114(54.39) | 0.43 | 1.05E-09 | 1.25E-08 | Yes | Up | - |
| O14818 | PSMA7 | 60/114(52.63) | 0.27 | 7.01E-04 | 1.80E-03 | Yes | Up | - |
| P34820 | BMP8B | 61/114(53.51) | 0.43 | 2.46E-03 | 5.52E-03 | Yes | Up | - |
| Q6ZMC9 | SIGLEC15 | 57/114(50.0) | 0.59 | 2.36E-03 | 5.33E-03 | Yes | Up | - |
| Q9H0H5 | RACGAP1 | 59/114(51.75) | 0.95 | 3.23E-11 | 6.29E-10 | Yes | Up | - |
| Q15020 | SART3 | 57/114(50.0) | 0.13 | 2.41E-03 | 5.43E-03 | Yes | Up | - |
| Q13158 | FADD | 60/114(52.63) | 0.51 | 1.23E-09 | 1.44E-08 | Yes | Up | - |
| Q9BQE3 | TUBA1C | 66/114(57.89) | 0.89 | 1.56E-13 | 6.94E-12 | Yes | Up | - |
| Q9H0A3 | TMEM191A | 63/114(55.26) | 0.6 | 1.37E-05 | 5.31E-05 | Yes | Up | - |
| P16278 | GLB1 | 60/114(52.63) | 0.39 | 3.85E-07 | 2.21E-06 | Yes | Up | - |
| Q92540 | SMG7 | 56/114(49.12) | 0.37 | 6.04E-07 | 3.29E-06 | Yes | Up | - |
| Q6RUI8 | C19ORF48 | 66/114(57.89) | 0.56 | 5.75E-08 | 4.12E-07 | Yes | Up | - |
| Q6NXG1 | ESRP1 | 54/114(47.37) | 0.88 | 4.35E-05 | 1.49E-04 | Yes | Up | - |
| Q8NB91 | FANCB | 55/114(48.25) | 0.48 | 2.32E-04 | 6.67E-04 | Yes | Up | - |
| P34972 | CNR2 | 52/114(45.61) | 0.54 | 1.61E-02 | 2.96E-02 | Yes | Up | - |
| O95976 | IGSF6 | 57/114(50.0) | 0.41 | 2.73E-04 | 7.73E-04 | Yes | Up | - |
| Q5EBL2 | ZNF628 | 72/114(63.16) | 0.23 | 1.45E-03 | 3.45E-03 | Yes | Up | - |
| Q9H869 | YY1AP1 | 61/114(53.51) | 0.24 | 1.07E-05 | 4.27E-05 | Yes | Up | - |
| Q9UJX3 | ANAPC7 | 59/114(51.75) | 0.26 | 1.36E-06 | 6.73E-06 | Yes | Up | - |
| O43684 | BUB3 | 60/114(52.63) | 0.53 | 5.84E-10 | 7.51E-09 | Yes | Up | - |
| Q9UII2 | ATPIF1 | 65/114(57.02) | 0.4 | 1.17E-06 | 5.88E-06 | Yes | Up | - |
| Q06609 | RAD51 | 60/114(52.63) | 1.29 | 5.56E-13 | 1.97E-11 | Yes | Up | - |
| Q9NRP2 | C16ORF61 | 67/114(58.77) | 0.25 | 1.70E-03 | 3.97E-03 | Yes | Up | - |
| Q92900 | UPF1 | 59/114(51.75) | 0.16 | 8.63E-04 | 2.17E-03 | Yes | Up | - |
| Q9NQT4 | EXOSC5 | 63/114(55.26) | 0.33 | 1.83E-05 | 6.88E-05 | Yes | Up | - |
| Q15744 | CEBPE | 57/114(50.0) | 0.68 | 2.05E-04 | 5.99E-04 | Yes | Up | - |
| Q8TC12 | RDH11 | 57/114(50.0) | 0.26 | 7.06E-05 | 2.30E-04 | Yes | Up | - |
| P52434 | POLR2H | 63/114(55.26) | 0.53 | 5.09E-13 | 1.84E-11 | Yes | Up | - |
| P17509 | HOXB6 | 55/114(48.25) | 0.61 | 2.13E-04 | 6.18E-04 | Yes | Up | - |
| O00459 | PIK3R2 | 61/114(53.51) | 0.71 | 3.48E-13 | 1.33E-11 | Yes | Up | - |
| Q6IEE7 | TMEM132E | 60/114(52.63) | 0.5 | 1.65E-03 | 3.88E-03 | Yes | Up | - |
| Q96M11 | HYLS1 | 57/114(50.0) | 0.55 | 7.22E-10 | 8.98E-09 | Yes | Up | - |
| Q8N9L9 | ACOT4 | 63/114(55.26) | 0.49 | 1.08E-04 | 3.38E-04 | Yes | Up | - |
| Q8IYI8 | ZNF440 | 51/114(44.74) | 0.27 | 6.81E-04 | 1.75E-03 | Yes | Up | - |
| Q9BYD2 | MRPL9 | 66/114(57.89) | 0.33 | 5.82E-07 | 3.19E-06 | Yes | Up | - |
| P62491 | RAB11A | 53/114(46.49) | 0.19 | 2.33E-03 | 5.27E-03 | Yes | Up | - |
| Q5D0E6 | DALRD3 | 60/114(52.63) | 0.3 | 7.15E-05 | 2.33E-04 | Yes | Up | - |
| O43768 | ENSA | 64/114(56.14) | 0.39 | 9.89E-09 | 8.80E-08 | Yes | Up | - |
| Q06265 | EXOSC9 | 66/114(57.89) | 0.14 | 1.78E-03 | 4.14E-03 | Yes | Up | - |
| Q8WY91 | THAP4 | 59/114(51.75) | 0.21 | 9.28E-05 | 2.94E-04 | Yes | Up | - |
| P0C7P0 | CISD3 | 61/114(53.51) | 0.37 | 2.56E-06 | 1.18E-05 | Yes | Up | - |
| P52815 | MRPL12 | 61/114(53.51) | 0.48 | 1.39E-06 | 6.86E-06 | Yes | Up | - |
| Q8NC74 | C20ORF151 | 61/114(53.51) | 0.82 | 2.73E-05 | 9.81E-05 | Yes | Up | - |
| Q9Y6A9 | SPCS1 | 66/114(57.89) | 0.24 | 6.29E-05 | 2.07E-04 | Yes | Up | - |
| Q9BV90 | SNRNP25 | 61/114(53.51) | 0.7 | 3.80E-12 | 1.00E-10 | Yes | Up | - |
| Q7L7V1 | DHX32 | 65/114(57.02) | 0.17 | 2.51E-03 | 5.62E-03 | Yes | Up | - |
| Q9BXP2 | SLC12A9 | 61/114(53.51) | 0.2 | 1.92E-03 | 4.44E-03 | Yes | Up | - |
| O95388 | WISP1 | 62/114(54.39) | 2.07 | 7.85E-21 | 5.31E-18 | Yes | Up | - |
| Q53GA4 | PHLDA2 | 63/114(55.26) | 0.59 | 4.61E-05 | 1.57E-04 | Yes | Up | - |
| O15239 | NDUFA1 | 56/114(49.12) | 0.19 | 4.21E-03 | 8.93E-03 | Yes | Up | - |
| O95238 | SPDEF | 60/114(52.63) | 0.87 | 4.60E-05 | 1.57E-04 | Yes | Up | - |
| Q8IVV8 | NKAIN4 | 56/114(49.12) | 0.76 | 1.81E-04 | 5.35E-04 | Yes | Up | - |
| O95158 | NXPH4 | 65/114(57.02) | 0.93 | 1.39E-07 | 8.97E-07 | Yes | Up | - |
| Q8WUJ3 | KIAA1199 | 57/114(50.0) | 1.95 | 3.96E-17 | 7.96E-15 | Yes | Up | - |
| Q8NHY2 | RFWD2 | 60/114(52.63) | 0.26 | 8.37E-06 | 3.42E-05 | Yes | Up | - |
| O75947 | ATP5H | 60/114(52.63) | 0.23 | 2.80E-03 | 6.20E-03 | Yes | Up | - |
| Q92994 | BRF1 | 64/114(56.14) | 0.22 | 7.66E-04 | 1.95E-03 | Yes | Up | - |
| Q9Y3Q4 | HCN4 | 49/114(42.98) | 0.49 | 2.04E-02 | 3.65E-02 | Yes | Up | - |
| Q4KMZ8 | NKAIN1 | 60/114(52.63) | 2.24 | 4.62E-20 | 2.23E-17 | Yes | Up | - |
| Q99418 | CYTH2 | 70/114(61.4) | 0.27 | 2.65E-05 | 9.57E-05 | Yes | Up | - |
| O14734 | ACOT8 | 65/114(57.02) | 0.25 | 2.65E-04 | 7.53E-04 | Yes | Up | - |
| Q7Z4S9 | SH2D6 | 47/114(41.23) | 0.76 | 4.67E-03 | 9.82E-03 | Yes | Up | - |
| Q9UK59 | DBR1 | 53/114(46.49) | 0.15 | 3.05E-04 | 8.52E-04 | Yes | Up | - |
| Q96AQ2 | TMEM125 | 58/114(50.88) | 0.67 | 9.57E-05 | 3.02E-04 | Yes | Up | - |
| P49895 | DIO1 | 53/114(46.49) | 1.6 | 9.81E-12 | 2.28E-10 | Yes | Up | - |
| Q9Y620 | RAD54B | 56/114(49.12) | 0.86 | 3.35E-09 | 3.42E-08 | Yes | Up | - |
| Q9HBJ0 | PLAC1 | 58/114(50.88) | 2.1 | 3.63E-13 | 1.38E-11 | Yes | Up | - |
| Q5RGS2 | FAM75A2 | 34/114(29.82) | 1.61 | 1.48E-06 | 7.25E-06 | Yes | Up | - |
| Q8NBJ4 | GOLM1 | 63/114(55.26) | 0.58 | 1.01E-05 | 4.04E-05 | Yes | Up | - |
| Q53GL0 | PLEKHO1 | 68/114(59.65) | 0.41 | 1.76E-06 | 8.49E-06 | Yes | Up | - |
| Q15287 | RNPS1 | 60/114(52.63) | 0.35 | 5.06E-08 | 3.68E-07 | Yes | Up | - |
| O43683 | BUB1 | 56/114(49.12) | 1.59 | 6.37E-13 | 2.22E-11 | Yes | Up | - |
| Q8NFU3 | TSTD1 | 62/114(54.39) | 0.67 | 1.00E-10 | 1.64E-09 | Yes | Up | - |
| Q9BTV7 | CABLES2 | 61/114(53.51) | 0.62 | 8.01E-10 | 9.82E-09 | Yes | Up | - |
| Q99758 | ABCA3 | 57/114(50.0) | 0.31 | 6.99E-03 | 1.41E-02 | Yes | Up | - |
| O60284 | ST18 | 66/114(57.89) | 0.63 | 5.55E-04 | 1.46E-03 | Yes | Up | - |
| P09326 | CD48 | 55/114(48.25) | 0.29 | 1.42E-02 | 2.66E-02 | Yes | Up | - |
| Q9UK39 | CCRN4L | 60/114(52.63) | 0.28 | 6.68E-03 | 1.35E-02 | Yes | Up | - |
| Q9UNW8 | GPR132 | 65/114(57.02) | 0.38 | 3.26E-04 | 9.06E-04 | Yes | Up | - |
| Q9H201 | EPN3 | 57/114(50.0) | 1.17 | 5.12E-09 | 4.95E-08 | Yes | Up | - |
| P07858 | CTSB | 60/114(52.63) | 0.38 | 5.55E-06 | 2.36E-05 | Yes | Up | - |
| Q8N565 | MREG | 62/114(54.39) | 0.42 | 5.57E-04 | 1.47E-03 | Yes | Up | - |
| P55089 | UCN | 67/114(58.77) | 0.36 | 1.45E-02 | 2.70E-02 | Yes | Up | - |
| Q9UL51 | HCN2 | 62/114(54.39) | 1.26 | 2.50E-08 | 2.00E-07 | Yes | Up | - |
| Q9BYX4 | IFIH1 | 58/114(50.88) | 0.33 | 5.22E-04 | 1.38E-03 | Yes | Up | - |
| - | SNHG11 | 58/114(50.88) | 0.27 | 1.53E-04 | 4.61E-04 | Yes | Up | - |
| Q96RQ1 | ERGIC2 | 57/114(50.0) | 0.15 | 2.04E-02 | 3.66E-02 | Yes | Up | - |
| P0C7U0 | ELFN1 | 63/114(55.26) | 0.23 | 1.91E-02 | 3.45E-02 | Yes | Up | - |
| Q2M329 | CCDC96 | 66/114(57.89) | 0.59 | 1.70E-09 | 1.89E-08 | Yes | Up | - |
| P0CG42 | FAM157B | 47/114(41.23) | 0.52 | 1.71E-02 | 3.13E-02 | Yes | Up | - |
| P21583 | KITLG | 55/114(48.25) | 0.35 | 5.10E-03 | 1.06E-02 | Yes | Up | - |
| Q9P0J6 | MRPL36 | 63/114(55.26) | 0.23 | 2.26E-03 | 5.13E-03 | Yes | Up | - |
| O60701 | UGDH | 50/114(43.86) | 0.45 | 7.91E-05 | 2.55E-04 | Yes | Up | - |
| Q5TCH4 | CYP4A22 | 49/114(42.98) | 0.71 | 8.85E-03 | 1.74E-02 | Yes | Up | - |
| O60500 | NPHS1 | 50/114(43.86) | 1.37 | 2.05E-06 | 9.74E-06 | Yes | Up | - |
| O60825 | PFKFB2 | 62/114(54.39) | 0.34 | 1.61E-04 | 4.83E-04 | Yes | Up | - |
| P21815 | IBSP | 58/114(50.88) | 1.79 | 8.08E-07 | 4.26E-06 | Yes | Up | - |
| Q15059 | BRD3 | 53/114(46.49) | 0.14 | 1.41E-02 | 2.64E-02 | Yes | Up | - |
| Q9NPD3 | EXOSC4 | 66/114(57.89) | 0.6 | 2.14E-09 | 2.30E-08 | Yes | Up | - |
| P22794 | EVI2A | 60/114(52.63) | 0.46 | 4.10E-05 | 1.42E-04 | Yes | Up | - |
| Q9NPI8 | FANCF | 56/114(49.12) | 0.36 | 1.58E-04 | 4.74E-04 | Yes | Up | - |
| Q96C28 | ZNF707 | 66/114(57.89) | 0.45 | 6.03E-07 | 3.28E-06 | Yes | Up | - |
| Q8TCD1 | C18ORF32 | 55/114(48.25) | 0.26 | 6.12E-05 | 2.03E-04 | Yes | Up | - |
| Q6ZUT3 | FRMD7 | 60/114(52.63) | 0.45 | 1.24E-02 | 2.34E-02 | Yes | Up | - |
| Q14833 | GRM4 | 57/114(50.0) | 2.02 | 1.11E-13 | 5.33E-12 | Yes | Up | - |
| P81274 | GPSM2 | 60/114(52.63) | 0.6 | 2.89E-07 | 1.72E-06 | Yes | Up | - |
| O76054 | SEC14L2 | 57/114(50.0) | 0.51 | 2.12E-03 | 4.84E-03 | Yes | Up | - |
| Q14330 | GPR18 | 62/114(54.39) | 0.44 | 2.64E-03 | 5.87E-03 | Yes | Up | - |
| Q9UI12 | ATP6V1H | 63/114(55.26) | 0.18 | 3.05E-03 | 6.68E-03 | Yes | Up | - |
| P01137 | TGFB1 | 67/114(58.77) | 0.47 | 2.11E-06 | 9.98E-06 | Yes | Up | - |
| Q99714 | HSD17B10 | 64/114(56.14) | 0.36 | 6.55E-07 | 3.53E-06 | Yes | Up | - |
| Q6PI25 | CNIH2 | 61/114(53.51) | 1.67 | 4.32E-16 | 5.80E-14 | Yes | Up | - |
| Q8TEQ8 | PIGO | 57/114(50.0) | 0.31 | 3.98E-05 | 1.38E-04 | Yes | Up | - |
| Q49AA0 | ZNF642 | 59/114(51.75) | 0.26 | 1.27E-04 | 3.91E-04 | Yes | Up | - |
| Q6ISB3 | GRHL2 | 58/114(50.88) | 0.56 | 3.68E-03 | 7.91E-03 | Yes | Up | - |
| Q8NHP1 | AKR7L | 57/114(50.0) | 0.63 | 1.55E-05 | 5.96E-05 | Yes | Up | - |
| Q8N0Y2 | ZNF444 | 60/114(52.63) | 0.39 | 1.82E-07 | 1.14E-06 | Yes | Up | - |
| Q8IZF3 | GPR115 | 63/114(55.26) | 1.77 | 1.72E-09 | 1.90E-08 | Yes | Up | - |
| Q03923 | ZNF85 | 53/114(46.49) | 0.24 | 1.44E-03 | 3.42E-03 | Yes | Up | - |
| Q5GAN6 | RNASE10 | 59/114(51.75) | 0.79 | 9.14E-04 | 2.28E-03 | Yes | Up | - |
| Q8WZ55 | BSND | 44/114(38.6) | 0.72 | 6.81E-03 | 1.38E-02 | Yes | Up | - |
| Q9HBZ2 | ARNT2 | 60/114(52.63) | 1 | 5.34E-09 | 5.12E-08 | Yes | Up | - |
| Q6ZPD8 | DGAT2L6 | 35/114(30.7) | 1.27 | 1.78E-04 | 5.26E-04 | Yes | Up | - |
| P42575 | CASP2 | 59/114(51.75) | 0.22 | 2.45E-03 | 5.50E-03 | Yes | Up | - |
| Q02790 | FKBP4 | 60/114(52.63) | 0.81 | 6.90E-11 | 1.18E-09 | Yes | Up | - |
| P11049 | CD37 | 60/114(52.63) | 0.37 | 8.26E-04 | 2.08E-03 | Yes | Up | - |
| P55211 | CASP9 | 61/114(53.51) | 0.13 | 4.73E-03 | 9.92E-03 | Yes | Up | - |
| Q9Y6I3 | EPN1 | 69/114(60.53) | 0.26 | 4.07E-04 | 1.10E-03 | Yes | Up | - |
| - | LOC284551 | 52/114(45.61) | 0.73 | 6.01E-03 | 1.23E-02 | Yes | Up | - |
| B3KS81 | SRRM5 | 60/114(52.63) | 0.36 | 1.76E-05 | 6.66E-05 | Yes | Up | - |
| Q96G97 | BSCL2 | 66/114(57.89) | 0.48 | 1.08E-10 | 1.74E-09 | Yes | Up | - |
| Q96A54 | ADIPOR1 | 57/114(50.0) | 0.5 | 5.16E-09 | 4.98E-08 | Yes | Up | - |
| Q9BWG4 | SSBP4 | 68/114(59.65) | 0.27 | 2.23E-04 | 6.44E-04 | Yes | Up | - |
| Q9BWF2 | TRAIP | 64/114(56.14) | 0.66 | 6.44E-09 | 6.04E-08 | Yes | Up | - |
| Q9Y6M5 | SLC30A1 | 60/114(52.63) | 0.32 | 2.46E-04 | 7.02E-04 | Yes | Up | - |
| - | LOC645431 | 55/114(48.25) | 0.42 | 2.23E-03 | 5.07E-03 | Yes | Up | - |
| Q2MJR0 | SPRED3 | 65/114(57.02) | 0.55 | 1.02E-02 | 1.98E-02 | Yes | Up | - |
| O75529 | TAF5L | 55/114(48.25) | 0.22 | 1.42E-04 | 4.31E-04 | Yes | Up | - |
| Q99705 | MCHR1 | 59/114(51.75) | 0.79 | 3.90E-06 | 1.72E-05 | Yes | Up | - |
| B4DWJ7 | C8ORF55 | 64/114(56.14) | 0.31 | 3.65E-04 | 1.00E-03 | Yes | Up | - |
| Q6ZSZ5 | ARHGEF18 | 66/114(57.89) | 0.29 | 1.44E-07 | 9.27E-07 | Yes | Up | - |
| Q9H5V8 | CDCP1 | 57/114(50.0) | 0.52 | 9.25E-04 | 2.30E-03 | Yes | Up | - |
| Q9NUI1 | DECR2 | 63/114(55.26) | 0.27 | 2.82E-04 | 7.94E-04 | Yes | Up | - |
| Q8IYG6 | LRRC56 | 57/114(50.0) | 1.01 | 1.08E-10 | 1.74E-09 | Yes | Up | - |
| Q9ULZ3 | PYCARD | 66/114(57.89) | 1.02 | 7.71E-15 | 5.98E-13 | Yes | Up | - |
| Q9NXK6 | PAQR5 | 62/114(54.39) | 0.48 | 1.33E-03 | 3.19E-03 | Yes | Up | - |
| P51671 | CCL11 | 63/114(55.26) | 1.68 | 9.60E-10 | 1.15E-08 | Yes | Up | - |
| Q92985 | IRF7 | 62/114(54.39) | 1.13 | 8.13E-21 | 5.31E-18 | Yes | Up | - |
| O00203 | AP3B1 | 58/114(50.88) | 0.14 | 1.57E-03 | 3.70E-03 | Yes | Up | - |
| Q92630 | DYRK2 | 57/114(50.0) | 0.22 | 1.35E-03 | 3.23E-03 | Yes | Up | - |
| Q7Z7M9 | GALNT5 | 57/114(50.0) | 0.87 | 2.37E-06 | 1.11E-05 | Yes | Up | - |
| P67870 | CSNK2B | 62/114(54.39) | 0.19 | 1.77E-03 | 4.11E-03 | Yes | Up | - |
| Q96F07 | CYFIP2 | 63/114(55.26) | 0.42 | 2.76E-03 | 6.12E-03 | Yes | Up | - |
| Q8TAG5 | VSTM2A | 62/114(54.39) | 2.31 | 1.96E-12 | 5.74E-11 | Yes | Up | - |
| O60906 | SMPD2 | 65/114(57.02) | 0.45 | 1.75E-07 | 1.10E-06 | Yes | Up | - |
| O15205 | UBD | 59/114(51.75) | 0.5 | 9.10E-03 | 1.78E-02 | Yes | Up | - |
| - | SUMO1P3 | 56/114(49.12) | 0.18 | 1.16E-03 | 2.82E-03 | Yes | Up | - |
| O43570 | CA12 | 58/114(50.88) | 1.21 | 8.29E-11 | 1.39E-09 | Yes | Up | - |
| Q9NR22 | PRMT8 | 53/114(46.49) | 0.57 | 2.47E-02 | 4.34E-02 | Yes | Up | - |
| Q9BSJ6 | FAM64A | 56/114(49.12) | 1.39 | 2.19E-11 | 4.50E-10 | Yes | Up | - |
| Q9NVF7 | FBXO28 | 53/114(46.49) | 0.18 | 6.24E-03 | 1.27E-02 | Yes | Up | - |
| Q96M89 | CCDC138 | 57/114(50.0) | 0.34 | 1.53E-03 | 3.62E-03 | Yes | Up | - |
| P59998 | ARPC4 | 66/114(57.89) | 0.26 | 4.05E-07 | 2.31E-06 | Yes | Up | - |
| O75792 | RNASEH2A | 63/114(55.26) | 0.99 | 6.62E-16 | 7.89E-14 | Yes | Up | - |
| Q86VU5 | COMTD1 | 63/114(55.26) | 0.57 | 2.89E-06 | 1.32E-05 | Yes | Up | - |
| Q9NRP0 | OSTC | 65/114(57.02) | 0.35 | 1.24E-06 | 6.20E-06 | Yes | Up | - |
| P51617 | IRAK1 | 58/114(50.88) | 0.32 | 8.47E-05 | 2.71E-04 | Yes | Up | - |
| Q6NX49 | ZNF544 | 58/114(50.88) | 0.24 | 2.04E-03 | 4.68E-03 | Yes | Up | - |
| Q92613 | PHF16 | 52/114(45.61) | 0.49 | 1.50E-06 | 7.34E-06 | Yes | Up | - |
| Q9UMX9 | SLC45A2 | 54/114(47.37) | 0.58 | 8.26E-03 | 1.64E-02 | Yes | Up | - |
| Q2M3G4 | SHROOM1 | 59/114(51.75) | 0.76 | 1.51E-06 | 7.37E-06 | Yes | Up | - |
| Q9NR83 | SLC2A4RG | 60/114(52.63) | 0.21 | 1.10E-02 | 2.11E-02 | Yes | Up | - |
| O60244 | MED14 | 59/114(51.75) | 0.2 | 8.42E-03 | 1.66E-02 | Yes | Up | - |
| Q9NVT9 | ARMC1 | 59/114(51.75) | 0.15 | 2.57E-02 | 4.50E-02 | Yes | Up | - |
| Q9H568 | ACTL8 | 41/114(35.96) | 1.73 | 1.51E-06 | 7.37E-06 | Yes | Up | - |
| P57740 | NUP107 | 53/114(46.49) | 0.17 | 1.17E-03 | 2.84E-03 | Yes | Up | - |
| Q99942 | RNF5 | 65/114(57.02) | 0.15 | 3.44E-03 | 7.45E-03 | Yes | Up | - |
| Q16659 | MAPK6 | 60/114(52.63) | 0.17 | 5.96E-03 | 1.22E-02 | Yes | Up | - |
| Q96QD5 | DEPDC7 | 60/114(52.63) | 0.7 | 1.00E-07 | 6.72E-07 | Yes | Up | - |
| O15347 | HMGB3 | 59/114(51.75) | 1.02 | 2.01E-10 | 2.97E-09 | Yes | Up | - |
| Q8IZ81 | ELMOD2 | 51/114(44.74) | 0.25 | 8.02E-04 | 2.03E-03 | Yes | Up | - |
| P84085 | ARF5 | 68/114(59.65) | 0.33 | 2.98E-07 | 1.76E-06 | Yes | Up | - |
| Q6P6B7 | ANKRD16 | 59/114(51.75) | 0.21 | 9.99E-04 | 2.47E-03 | Yes | Up | - |
| P04626 | ERBB2 | 58/114(50.88) | 0.45 | 1.52E-03 | 3.60E-03 | Yes | Up | - |
| O75363 | BCAS1 | 55/114(48.25) | 1.38 | 2.56E-11 | 5.20E-10 | Yes | Up | - |
| Q9H3J6 | C12ORF65 | 59/114(51.75) | 0.12 | 1.76E-02 | 3.21E-02 | Yes | Up | - |
| Q96S38 | RPS6KC1 | 57/114(50.0) | 0.23 | 2.08E-04 | 6.04E-04 | Yes | Up | - |
| O43761 | SYNGR3 | 65/114(57.02) | 1.38 | 1.74E-13 | 7.59E-12 | Yes | Up | - |
| P30793 | GCH1 | 57/114(50.0) | 0.41 | 2.52E-05 | 9.15E-05 | Yes | Up | - |
| Q9UKZ1 | C2ORF29 | 60/114(52.63) | 0.26 | 2.25E-05 | 8.28E-05 | Yes | Up | - |
| A0AV02 | SLC12A8 | 55/114(48.25) | 0.98 | 1.10E-10 | 1.77E-09 | Yes | Up | - |
| Q13509 | TUBB3 | 62/114(54.39) | 1.36 | 2.12E-13 | 8.82E-12 | Yes | Up | - |
| Q8NEW0 | SLC30A7 | 55/114(48.25) | 0.32 | 4.68E-06 | 2.02E-05 | Yes | Up | - |
| Q8IX12 | CCAR1 | 54/114(47.37) | 0.32 | 1.10E-06 | 5.57E-06 | Yes | Up | - |
| A5D8V7 | CCDC151 | 59/114(51.75) | 1.11 | 4.34E-11 | 8.03E-10 | Yes | Up | - |
| O94851 | MICAL2 | 61/114(53.51) | 0.92 | 2.05E-12 | 5.96E-11 | Yes | Up | - |
| O60779 | SLC19A2 | 58/114(50.88) | 0.48 | 7.89E-06 | 3.24E-05 | Yes | Up | - |
| - | LOC388955 | 68/114(59.65) | 0.27 | 7.17E-05 | 2.33E-04 | Yes | Up | - |
| Q7Z309 | FAM122B | 54/114(47.37) | 0.21 | 1.16E-03 | 2.83E-03 | Yes | Up | - |
| A9Z1Z3 | FER1L4 | 61/114(53.51) | 0.43 | 1.08E-02 | 2.08E-02 | Yes | Up | - |
| P56851 | EDDM3B | 46/114(40.35) | 1.06 | 1.53E-04 | 4.61E-04 | Yes | Up | - |
| Q9Y284 | C19ORF56 | 60/114(52.63) | 0.21 | 1.01E-04 | 3.18E-04 | Yes | Up | - |
| Q10570 | CPSF1 | 64/114(56.14) | 0.29 | 4.68E-04 | 1.25E-03 | Yes | Up | - |
| O15389 | SIGLEC5 | 57/114(50.0) | 0.47 | 1.37E-04 | 4.17E-04 | Yes | Up | - |
| P54284 | CACNB3 | 62/114(54.39) | 0.74 | 2.34E-11 | 4.79E-10 | Yes | Up | - |
| Q9Y592 | CCDC41 | 53/114(46.49) | 0.2 | 4.79E-03 | 1.00E-02 | Yes | Up | - |
| P07492 | GRP | 66/114(57.89) | 0.78 | 1.07E-03 | 2.62E-03 | Yes | Up | - |
| Q9UBW8 | COPS7A | 59/114(51.75) | 0.18 | 2.43E-04 | 6.96E-04 | Yes | Up | - |
| O15049 | N4BP3 | 63/114(55.26) | 0.96 | 4.76E-13 | 1.74E-11 | Yes | Up | - |
| Q8WW27 | APOBEC4 | 37/114(32.46) | 1.77 | 1.59E-07 | 1.01E-06 | Yes | Up | - |
| A6NFY7 | SDHAF1 | 59/114(51.75) | 0.14 | 8.70E-03 | 1.71E-02 | Yes | Up | - |
| Q5TB30 | DEPDC1 | 58/114(50.88) | 1.43 | 1.14E-11 | 2.56E-10 | Yes | Up | - |
| - | DVWA | 31/114(27.19) | 1.13 | 1.45E-03 | 3.44E-03 | Yes | Up | - |
| Q86UK0 | ABCA12 | 55/114(48.25) | 1.03 | 3.17E-05 | 1.13E-04 | Yes | Up | - |
| O15446 | CD3EAP | 61/114(53.51) | 0.21 | 9.54E-04 | 2.37E-03 | Yes | Up | - |
| Q7Z3K3 | POGZ | 50/114(43.86) | 0.15 | 9.01E-03 | 1.77E-02 | Yes | Up | - |
| Q9BZL1 | UBL5 | 60/114(52.63) | 0.43 | 3.50E-08 | 2.66E-07 | Yes | Up | - |
| Q8IW36 | ZNF695 | 61/114(53.51) | 1.38 | 1.39E-11 | 3.03E-10 | Yes | Up | - |
| P28566 | HTR1E | 33/114(28.95) | 1.34 | 6.78E-04 | 1.75E-03 | Yes | Up | - |
| Q01105 | SET | 60/114(52.63) | 0.25 | 5.19E-05 | 1.75E-04 | Yes | Up | - |
| P08779 | KRT16 | 60/114(52.63) | 0.59 | 1.37E-02 | 2.56E-02 | Yes | Up | - |
| P07737 | PFN1 | 65/114(57.02) | 0.36 | 1.76E-06 | 8.49E-06 | Yes | Up | - |
| Q7Z7M8 | B3GNT8 | 61/114(53.51) | 0.19 | 2.79E-02 | 4.83E-02 | Yes | Up | - |
| Q9Y5Y2 | NUBP2 | 71/114(62.28) | 0.47 | 5.45E-09 | 5.21E-08 | Yes | Up | - |
| Q6UWV6 | ENPP7 | 42/114(36.84) | 0.94 | 5.85E-04 | 1.53E-03 | Yes | Up | - |
| P54725 | RAD23A | 57/114(50.0) | 0.15 | 2.47E-02 | 4.34E-02 | Yes | Up | - |
| Q00796 | SORD | 56/114(49.12) | 0.82 | 6.19E-08 | 4.40E-07 | Yes | Up | - |
| O14519 | CDK2AP1 | 54/114(47.37) | 0.17 | 2.15E-02 | 3.84E-02 | Yes | Up | - |
| Q96DM3 | C18ORF8 | 60/114(52.63) | 0.13 | 1.51E-03 | 3.58E-03 | Yes | Up | - |
| P36956 | SREBF1 | 62/114(54.39) | 0.48 | 8.61E-06 | 3.51E-05 | Yes | Up | - |
| - | ZSCAN12P1 | 55/114(48.25) | 0.54 | 1.52E-05 | 5.85E-05 | Yes | Up | - |
| Q6UN15 | FIP1L1 | 59/114(51.75) | 0.22 | 3.81E-05 | 1.33E-04 | Yes | Up | - |
| Q96SL1 | DIRC2 | 57/114(50.0) | 0.19 | 1.18E-03 | 2.87E-03 | Yes | Up | - |
| Q9UGM1 | CHRNA9 | 45/114(39.47) | 2.16 | 2.77E-14 | 1.72E-12 | Yes | Up | - |
| Q6NSJ2 | PHLDB3 | 70/114(61.4) | 0.62 | 3.67E-08 | 2.77E-07 | Yes | Up | - |
| P49137 | MAPKAPK2 | 66/114(57.89) | 0.61 | 3.10E-10 | 4.31E-09 | Yes | Up | - |
| P62316 | SNRPD2 | 71/114(62.28) | 0.22 | 1.79E-04 | 5.30E-04 | Yes | Up | - |
| P15086 | CPB1 | 64/114(56.14) | 0.91 | 2.12E-03 | 4.84E-03 | Yes | Up | - |
| - | LOC100132354 | 48/114(42.11) | 1.11 | 1.68E-04 | 5.01E-04 | Yes | Up | - |
| Q9Y3A0 | COQ4 | 62/114(54.39) | 0.17 | 2.05E-03 | 4.71E-03 | Yes | Up | - |
| Q969R2 | OSBP2 | 60/114(52.63) | 0.52 | 1.40E-05 | 5.43E-05 | Yes | Up | - |
| Q15004 | KIAA0101 | 58/114(50.88) | 1.65 | 1.49E-14 | 1.03E-12 | Yes | Up | - |
| O75528 | TADA3 | 71/114(62.28) | 0.19 | 3.48E-05 | 1.22E-04 | Yes | Up | - |
| Q86VK4 | ZNF410 | 63/114(55.26) | 0.13 | 6.08E-04 | 1.58E-03 | Yes | Up | - |
| Q9Y6G1 | TMEM14A | 63/114(55.26) | 0.45 | 2.61E-08 | 2.06E-07 | Yes | Up | - |
| P13796 | LCP1 | 50/114(43.86) | 0.3 | 6.65E-03 | 1.35E-02 | Yes | Up | - |
| Q6PL24 | TMED8 | 57/114(50.0) | 0.14 | 2.09E-02 | 3.74E-02 | Yes | Up | - |
| Q6P087 | RPUSD3 | 57/114(50.0) | 0.16 | 9.30E-03 | 1.82E-02 | Yes | Up | - |
| P48595 | SERPINB10 | 28/114(24.56) | 1.13 | 3.70E-03 | 7.95E-03 | Yes | Up | - |
| P43364 | MAGEA11 | 32/114(28.07) | 0.85 | 2.78E-02 | 4.82E-02 | Yes | Up | - |
| P09669 | COX6C | 61/114(53.51) | 1.09 | 8.02E-13 | 2.69E-11 | Yes | Up | - |
| Q9BYJ1 | ALOXE3 | 65/114(57.02) | 0.48 | 1.19E-02 | 2.25E-02 | Yes | Up | - |
| Q6TCH4 | PAQR6 | 57/114(50.0) | 0.47 | 2.28E-03 | 5.17E-03 | Yes | Up | - |
| Q53HC9 | TSSC1 | 66/114(57.89) | 0.44 | 6.61E-09 | 6.17E-08 | Yes | Up | - |
| Q96A59 | MARVELD3 | 60/114(52.63) | 0.48 | 4.84E-03 | 1.01E-02 | Yes | Up | - |
| - | GUSBP3 | 64/114(56.14) | 0.46 | 6.54E-04 | 1.69E-03 | Yes | Up | - |
| Q9H4T2 | ZSCAN16 | 54/114(47.37) | 0.3 | 4.86E-04 | 1.29E-03 | Yes | Up | - |
| Q96GE5 | ZNF799 | 55/114(48.25) | 0.2 | 3.68E-03 | 7.90E-03 | Yes | Up | - |
| Q8IZ83 | ALDH16A1 | 68/114(59.65) | 0.22 | 1.75E-03 | 4.09E-03 | Yes | Up | - |
| Q8WW35 | TCTEX1D2 | 68/114(59.65) | 0.67 | 1.24E-11 | 2.74E-10 | Yes | Up | - |
| Q9H8Y8 | GORASP2 | 55/114(48.25) | 0.21 | 2.50E-04 | 7.13E-04 | Yes | Up | - |
| P56545 | CTBP2 | 54/114(47.37) | 0.34 | 2.85E-06 | 1.30E-05 | Yes | Up | - |
| O15355 | PPM1G | 62/114(54.39) | 0.37 | 3.11E-08 | 2.41E-07 | Yes | Up | - |
| Q13084 | MRPL28 | 68/114(59.65) | 0.42 | 2.31E-08 | 1.86E-07 | Yes | Up | - |
| Q9BV38 | WDR18 | 65/114(57.02) | 0.41 | 4.98E-08 | 3.63E-07 | Yes | Up | - |
| Q9NRP7 | STK36 | 60/114(52.63) | 0.2 | 6.22E-03 | 1.27E-02 | Yes | Up | - |
| Q6UXD5 | SEZ6L2 | 60/114(52.63) | 0.66 | 1.76E-04 | 5.23E-04 | Yes | Up | - |
| O75674 | TOM1L1 | 53/114(46.49) | 0.49 | 2.39E-05 | 8.72E-05 | Yes | Up | - |
| Q9NQS7 | INCENP | 51/114(44.74) | 0.42 | 6.19E-06 | 2.60E-05 | Yes | Up | - |
| A6NFE2 | C12ORF70 | 54/114(47.37) | 1.1 | 2.13E-06 | 1.00E-05 | Yes | Up | - |
| Q13950 | RUNX2 | 64/114(56.14) | 0.66 | 1.03E-06 | 5.28E-06 | Yes | Up | - |
| Q9H8V3 | ECT2 | 62/114(54.39) | 0.98 | 1.41E-11 | 3.06E-10 | Yes | Up | - |
| Q8TAC9 | SCAMP5 | 62/114(54.39) | 0.32 | 1.49E-03 | 3.54E-03 | Yes | Up | - |
| Q9P2E9 | RRBP1 | 61/114(53.51) | 0.32 | 3.38E-05 | 1.19E-04 | Yes | Up | - |
| Q5JPE7 | NOMO2 | 57/114(50.0) | 0.22 | 1.68E-02 | 3.08E-02 | Yes | Up | - |
| Q96FN5 | KIF12 | 58/114(50.88) | 0.53 | 1.73E-02 | 3.16E-02 | Yes | Up | - |
| Q8NG50 | RDM1 | 57/114(50.0) | 1.44 | 7.10E-09 | 6.56E-08 | Yes | Up | - |
| Q8IUB2 | WFDC3 | 63/114(55.26) | 0.49 | 5.56E-04 | 1.46E-03 | Yes | Up | - |
| Q9NX70 | MED29 | 64/114(56.14) | 0.23 | 3.37E-05 | 1.19E-04 | Yes | Up | - |
| Q9C0C9 | UBE2O | 56/114(49.12) | 0.29 | 1.50E-05 | 5.77E-05 | Yes | Up | - |
| Q9NY26 | SLC39A1 | 63/114(55.26) | 0.45 | 3.01E-10 | 4.20E-09 | Yes | Up | - |
| P09238 | MMP10 | 63/114(55.26) | 1.64 | 4.13E-10 | 5.56E-09 | Yes | Up | - |
| Q9H900 | ZWILCH | 63/114(55.26) | 0.59 | 2.17E-08 | 1.77E-07 | Yes | Up | - |
| Q8WUX1 | SLC38A5 | 65/114(57.02) | 0.9 | 4.05E-08 | 3.03E-07 | Yes | Up | - |
| Q9GZN7 | ROGDI | 63/114(55.26) | 0.77 | 6.95E-12 | 1.69E-10 | Yes | Up | - |
| Q9UBF2 | COPG2 | 61/114(53.51) | 0.14 | 2.37E-02 | 4.18E-02 | Yes | Up | - |
| P39900 | MMP12 | 54/114(47.37) | 0.69 | 2.40E-03 | 5.41E-03 | Yes | Up | - |
| Q96JZ2 | HSH2D | 58/114(50.88) | 1.29 | 5.20E-15 | 4.36E-13 | Yes | Up | - |
| Q9UI40 | SLC24A2 | 63/114(55.26) | 2.41 | 1.21E-13 | 5.66E-12 | Yes | Up | - |
| Q9Y2W6 | TDRKH | 63/114(55.26) | 0.62 | 1.06E-08 | 9.36E-08 | Yes | Up | - |
| P13631 | RARG | 57/114(50.0) | 0.16 | 1.11E-02 | 2.14E-02 | Yes | Up | - |
| Q6NZ67 | FAM128B | 67/114(58.77) | 0.52 | 1.77E-07 | 1.11E-06 | Yes | Up | - |
| Q15286 | RAB35 | 57/114(50.0) | 0.17 | 8.11E-05 | 2.61E-04 | Yes | Up | - |
| Q96N22 | ZNF681 | 55/114(48.25) | 0.56 | 3.51E-06 | 1.57E-05 | Yes | Up | - |
| Q9BXI9 | C1QTNF6 | 62/114(54.39) | 1.41 | 1.22E-20 | 7.15E-18 | Yes | Up | - |
| Q2T9L4 | C15ORF59 | 59/114(51.75) | 0.47 | 4.49E-03 | 9.49E-03 | Yes | Up | - |
| Q9BZE0 | GLIS2 | 59/114(51.75) | 0.52 | 4.40E-06 | 1.91E-05 | Yes | Up | - |
| Q9BQI6 | ANKRD32 | 57/114(50.0) | 0.22 | 4.90E-03 | 1.02E-02 | Yes | Up | - |
| A2RU67 | KIAA1467 | 49/114(42.98) | 0.61 | 3.44E-05 | 1.21E-04 | Yes | Up | - |
| A4D1S5 | RAB19 | 60/114(52.63) | 0.98 | 6.25E-07 | 3.39E-06 | Yes | Up | - |
| A4ZI32 | C17ORF70 | 59/114(51.75) | 0.33 | 1.38E-07 | 8.92E-07 | Yes | Up | - |
| P49916 | LIG3 | 53/114(46.49) | 0.52 | 1.68E-08 | 1.41E-07 | Yes | Up | - |
| Q8IZL8 | PELP1 | 60/114(52.63) | 0.14 | 6.78E-03 | 1.37E-02 | Yes | Up | - |
| Q9HAW4 | CLSPN | 56/114(49.12) | 1.45 | 6.59E-12 | 1.62E-10 | Yes | Up | - |
| P02461 | COL3A1 | 61/114(53.51) | 1.02 | 2.49E-09 | 2.62E-08 | Yes | Up | - |
| Q9ULA0 | DNPEP | 68/114(59.65) | 0.2 | 3.48E-04 | 9.59E-04 | Yes | Up | - |
| Q7Z6J0 | SH3RF1 | 59/114(51.75) | 0.19 | 2.08E-02 | 3.72E-02 | Yes | Up | - |
| Q8IY81 | FTSJ3 | 59/114(51.75) | 0.24 | 1.80E-04 | 5.33E-04 | Yes | Up | - |
| Q12907 | LMAN2 | 64/114(56.14) | 0.41 | 1.88E-07 | 1.17E-06 | Yes | Up | - |
| Q07617 | SPAG1 | 56/114(49.12) | 0.73 | 2.06E-08 | 1.69E-07 | Yes | Up | - |
| P62304 | SNRPE | 62/114(54.39) | 0.46 | 4.79E-07 | 2.68E-06 | Yes | Up | - |
| O95948 | ONECUT2 | 52/114(45.61) | 0.82 | 1.22E-04 | 3.76E-04 | Yes | Up | - |
| P17980 | PSMC3 | 57/114(50.0) | 0.19 | 4.00E-03 | 8.52E-03 | Yes | Up | - |
| Q8N531 | FBXL6 | 63/114(55.26) | 0.76 | 4.65E-11 | 8.47E-10 | Yes | Up | - |
| P46977 | STT3A | 58/114(50.88) | 0.2 | 8.93E-04 | 2.23E-03 | Yes | Up | - |
| Q9NVE4 | CCDC87 | 60/114(52.63) | 0.76 | 1.46E-07 | 9.37E-07 | Yes | Up | - |
| P55287 | CDH11 | 62/114(54.39) | 1.04 | 2.77E-14 | 1.72E-12 | Yes | Up | - |
| Q96N03 | VSTM2L | 59/114(51.75) | 1.21 | 6.11E-09 | 5.76E-08 | Yes | Up | - |
| Q12851 | MAP4K2 | 61/114(53.51) | 0.29 | 6.85E-05 | 2.24E-04 | Yes | Up | - |
| Q969P0 | IGSF8 | 59/114(51.75) | 0.62 | 2.46E-13 | 9.96E-12 | Yes | Up | - |
| Q7Z629 | C22ORF13 | 58/114(50.88) | 0.14 | 2.33E-02 | 4.12E-02 | Yes | Up | - |
| P33032 | MC5R | 38/114(33.33) | 0.72 | 2.89E-02 | 4.98E-02 | Yes | Up | - |
| Q6ZSG2 | FAM196A | 59/114(51.75) | 1.69 | 8.95E-17 | 1.56E-14 | Yes | Up | - |
| Q96BJ8 | ELMO3 | 61/114(53.51) | 0.57 | 2.32E-04 | 6.68E-04 | Yes | Up | - |
| Q15048 | LRRC14 | 62/114(54.39) | 0.16 | 1.47E-02 | 2.73E-02 | Yes | Up | - |
| Q8WWI5 | SLC44A1 | 59/114(51.75) | 0.34 | 2.64E-06 | 1.22E-05 | Yes | Up | - |
| Q9UHC6 | CNTNAP2 | 55/114(48.25) | 1.64 | 2.38E-10 | 3.43E-09 | Yes | Up | - |
| P49842 | STK19 | 58/114(50.88) | 0.17 | 3.20E-03 | 6.97E-03 | Yes | Up | - |
| Q9BY79 | MFRP | 63/114(55.26) | 0.49 | 1.40E-04 | 4.27E-04 | Yes | Up | - |
| Q6RW13 | AGTRAP | 65/114(57.02) | 0.44 | 2.67E-10 | 3.78E-09 | Yes | Up | - |
| O75832 | PSMD10 | 56/114(49.12) | 0.2 | 1.39E-03 | 3.32E-03 | Yes | Up | - |
| Q9BSU1 | C16ORF70 | 62/114(54.39) | 0.22 | 2.63E-05 | 9.52E-05 | Yes | Up | - |
| Q9Y2G0 | EFR3B | 60/114(52.63) | 0.66 | 5.25E-07 | 2.91E-06 | Yes | Up | - |
| P20671 | HIST1H2AD | 51/114(44.74) | 1.95 | 7.27E-11 | 1.24E-09 | Yes | Up | - |
| Q9P1V8 | C14ORF174 | 60/114(52.63) | 0.67 | 9.05E-06 | 3.67E-05 | Yes | Up | - |
| P53677 | AP3M2 | 62/114(54.39) | 0.24 | 4.57E-04 | 1.22E-03 | Yes | Up | - |
| Q96AY3 | FKBP10 | 56/114(49.12) | 0.56 | 3.18E-07 | 1.86E-06 | Yes | Up | - |
| Q8IV56 | PRR15 | 59/114(51.75) | 1.34 | 1.25E-12 | 3.88E-11 | Yes | Up | - |
| P62877 | RBX1 | 62/114(54.39) | 0.27 | 1.45E-04 | 4.39E-04 | Yes | Up | - |
| Q14157 | UBAP2L | 55/114(48.25) | 0.43 | 1.03E-08 | 9.09E-08 | Yes | Up | - |
| O43583 | DENR | 55/114(48.25) | 0.2 | 3.86E-04 | 1.05E-03 | Yes | Up | - |
| - | NCRNA00094 | 62/114(54.39) | 0.15 | 2.37E-03 | 5.35E-03 | Yes | Up | - |
| P01033 | TIMP1 | 67/114(58.77) | 0.54 | 5.14E-06 | 2.20E-05 | Yes | Up | - |
| Q8N9M5 | TMEM102 | 59/114(51.75) | 0.24 | 1.67E-03 | 3.92E-03 | Yes | Up | - |
| A6NML5 | TMEM212 | 42/114(36.84) | 0.87 | 2.33E-03 | 5.28E-03 | Yes | Up | - |
| Q5T5N4 | C6ORF118 | 32/114(28.07) | 1.05 | 3.22E-03 | 7.02E-03 | Yes | Up | - |
| Q9HBL7 | C9ORF46 | 66/114(57.89) | 0.33 | 2.90E-06 | 1.32E-05 | Yes | Up | - |
| Q58WW2 | DCAF6 | 57/114(50.0) | 0.15 | 2.83E-02 | 4.90E-02 | Yes | Up | - |
| Q9NV88 | INTS9 | 69/114(60.53) | 0.16 | 1.09E-03 | 2.67E-03 | Yes | Up | - |
| P29353 | SHC1 | 57/114(50.0) | 0.21 | 3.60E-05 | 1.26E-04 | Yes | Up | - |
| O14777 | NDC80 | 59/114(51.75) | 1.59 | 4.36E-14 | 2.47E-12 | Yes | Up | - |
| Q8NA92 | THAP8 | 62/114(54.39) | 0.29 | 4.87E-05 | 1.65E-04 | Yes | Up | - |
| P17096 | HMGA1 | 60/114(52.63) | 0.61 | 4.84E-07 | 2.70E-06 | Yes | Up | - |
| P15291 | B4GALT1 | 70/114(61.4) | 0.34 | 6.66E-05 | 2.18E-04 | Yes | Up | - |
| P04440 | HLA-DPB1 | 60/114(52.63) | 0.25 | 1.48E-02 | 2.75E-02 | Yes | Up | - |
| P04075 | ALDOA | 65/114(57.02) | 0.57 | 8.87E-08 | 6.04E-07 | Yes | Up | - |
| J3KTI2 | C17ORF37 | 63/114(55.26) | 0.5 | 3.19E-05 | 1.13E-04 | Yes | Up | - |
| Q9BV99 | LRRC61 | 61/114(53.51) | 0.39 | 2.78E-05 | 9.99E-05 | Yes | Up | - |
| Q9UPI3 | FLVCR2 | 60/114(52.63) | 0.21 | 2.39E-02 | 4.21E-02 | Yes | Up | - |
| Q6T4P5 | LPPR3 | 58/114(50.88) | 0.85 | 5.04E-04 | 1.34E-03 | Yes | Up | - |
| Q96JA3 | PLEKHA8 | 58/114(50.88) | 0.36 | 5.83E-05 | 1.94E-04 | Yes | Up | - |
| P15848 | ARSB | 54/114(47.37) | 0.26 | 6.70E-03 | 1.36E-02 | Yes | Up | - |
| Q96DA6 | DNAJC19 | 62/114(54.39) | 0.25 | 7.61E-05 | 2.46E-04 | Yes | Up | - |
| Q99687 | MEIS3 | 65/114(57.02) | 0.65 | 1.18E-07 | 7.71E-07 | Yes | Up | - |
| Q6P996 | PDXDC1 | 59/114(51.75) | 0.55 | 1.26E-09 | 1.46E-08 | Yes | Up | - |
| Q15738 | NSDHL | 63/114(55.26) | 0.19 | 2.45E-03 | 5.50E-03 | Yes | Up | - |
| Q9NY47 | CACNA2D2 | 58/114(50.88) | 0.47 | 4.92E-03 | 1.03E-02 | Yes | Up | - |
| Q3V5L5 | MGAT5B | 61/114(53.51) | 0.68 | 6.49E-05 | 2.13E-04 | Yes | Up | - |
| Q9H788 | SH2D4A | 57/114(50.0) | 0.48 | 4.34E-05 | 1.49E-04 | Yes | Up | - |
| Q05084 | ICA1 | 60/114(52.63) | 0.39 | 4.54E-06 | 1.97E-05 | Yes | Up | - |
| P16949 | STMN1 | 60/114(52.63) | 0.75 | 2.46E-09 | 2.59E-08 | Yes | Up | - |
| Q9BYH1 | SEZ6L | 62/114(54.39) | 0.74 | 5.52E-04 | 1.45E-03 | Yes | Up | - |
| - | MGC12982 | 65/114(57.02) | 0.24 | 1.94E-02 | 3.49E-02 | Yes | Up | - |
| O75973 | C1QL1 | 62/114(54.39) | 0.42 | 9.18E-03 | 1.80E-02 | Yes | Up | - |
| Q9BV73 | CEP250 | 59/114(51.75) | 0.44 | 5.31E-08 | 3.84E-07 | Yes | Up | - |
| Q13242 | SFRS9 | 68/114(59.65) | 0.37 | 1.54E-09 | 1.74E-08 | Yes | Up | - |
| Q14147 | DHX34 | 59/114(51.75) | 0.37 | 8.37E-07 | 4.39E-06 | Yes | Up | - |
| Q8WWH5 | TRUB1 | 54/114(47.37) | 0.18 | 1.45E-02 | 2.70E-02 | Yes | Up | - |
| O43820 | HYAL3 | 60/114(52.63) | 0.53 | 3.06E-07 | 1.80E-06 | Yes | Up | - |
| P80365 | HSD11B2 | 61/114(53.51) | 0.54 | 2.29E-05 | 8.40E-05 | Yes | Up | - |
| Q5EE01 | CENPW | 63/114(55.26) | 0.5 | 2.32E-05 | 8.51E-05 | Yes | Up | - |
| H7BXH9 | FAM119A | 63/114(55.26) | 0.2 | 7.27E-04 | 1.86E-03 | Yes | Up | - |
| O14745 | SLC9A3R1 | 61/114(53.51) | 1.1 | 2.44E-13 | 9.93E-12 | Yes | Up | - |
| Q8TCW7 | ZPLD1 | 54/114(47.37) | 0.93 | 3.34E-06 | 1.50E-05 | Yes | Up | - |
| Q00613 | HSF1 | 58/114(50.88) | 0.19 | 1.57E-02 | 2.90E-02 | Yes | Up | - |
| A8K979 | ERI2 | 57/114(50.0) | 0.25 | 2.77E-03 | 6.14E-03 | Yes | Up | - |
| P56270 | MAZ | 65/114(57.02) | 0.73 | 1.91E-13 | 8.15E-12 | Yes | Up | - |
| Q53GL7 | PARP10 | 60/114(52.63) | 0.46 | 2.68E-06 | 1.23E-05 | Yes | Up | - |
| Q9NVN8 | GNL3L | 56/114(49.12) | 0.22 | 1.55E-03 | 3.65E-03 | Yes | Up | - |
| Q107X0 | KLKP1 | 60/114(52.63) | 1.83 | 3.68E-11 | 7.00E-10 | Yes | Up | - |
| P36405 | ARL3 | 54/114(47.37) | 0.35 | 6.26E-07 | 3.39E-06 | Yes | Up | - |
| Q8NBN7 | RDH13 | 57/114(50.0) | 0.41 | 9.55E-09 | 8.54E-08 | Yes | Up | - |
| P26374 | CHML | 55/114(48.25) | 0.37 | 5.74E-04 | 1.50E-03 | Yes | Up | - |
| P17024 | ZNF20 | 57/114(50.0) | 0.25 | 5.25E-04 | 1.39E-03 | Yes | Up | - |
| Q96MA6 | C9ORF98 | 64/114(56.14) | 0.72 | 5.17E-08 | 3.75E-07 | Yes | Up | - |
| Q8NBX0 | SCCPDH | 59/114(51.75) | 0.45 | 6.30E-05 | 2.08E-04 | Yes | Up | - |
| O14657 | TOR1B | 65/114(57.02) | 0.23 | 1.59E-05 | 6.07E-05 | Yes | Up | - |
| Q09028 | RBBP4 | 53/114(46.49) | 0.17 | 3.17E-03 | 6.91E-03 | Yes | Up | - |
| O76027 | ANXA9 | 58/114(50.88) | 1.37 | 1.60E-14 | 1.09E-12 | Yes | Up | - |
| Q86YJ5 | Mar-09 | 63/114(55.26) | 0.46 | 1.05E-11 | 2.42E-10 | Yes | Up | - |
| Q9H7X7 | RABL5 | 64/114(56.14) | 0.34 | 6.60E-07 | 3.55E-06 | Yes | Up | - |
| Q9BXP5 | SRRT | 57/114(50.0) | 0.35 | 1.21E-09 | 1.41E-08 | Yes | Up | - |
| Q6P1X5 | TAF2 | 60/114(52.63) | 0.24 | 9.37E-03 | 1.83E-02 | Yes | Up | - |
| Q7L2K0 | C16ORF59 | 64/114(56.14) | 1.5 | 5.42E-17 | 1.03E-14 | Yes | Up | - |
| Q9HC57 | WFDC1 | 56/114(49.12) | 0.6 | 1.65E-04 | 4.94E-04 | Yes | Up | - |
| Q96KB5 | PBK | 60/114(52.63) | 1.73 | 7.78E-15 | 5.99E-13 | Yes | Up | - |
| Q9Y296 | TRAPPC4 | 56/114(49.12) | 0.14 | 1.11E-02 | 2.13E-02 | Yes | Up | - |
| Q13257 | MAD2L1 | 55/114(48.25) | 1 | 1.90E-10 | 2.83E-09 | Yes | Up | - |
| Q9BUP0 | EFHD1 | 49/114(42.98) | 0.53 | 3.28E-05 | 1.16E-04 | Yes | Up | - |
| Q99729 | HNRNPAB | 63/114(55.26) | 0.5 | 1.31E-08 | 1.12E-07 | Yes | Up | - |
| Q8N8R3 | SLC25A29 | 65/114(57.02) | 0.3 | 5.25E-04 | 1.39E-03 | Yes | Up | - |
| - | UBE2MP1 | 64/114(56.14) | 0.18 | 1.06E-02 | 2.04E-02 | Yes | Up | - |
| Q13882 | PTK6 | 62/114(54.39) | 1.02 | 1.34E-07 | 8.70E-07 | Yes | Up | - |
| Q5SZQ8 | CELF3 | 46/114(40.35) | 1.11 | 5.71E-04 | 1.50E-03 | Yes | Up | - |
| P55789 | GFER | 66/114(57.89) | 0.58 | 6.50E-13 | 2.26E-11 | Yes | Up | - |
| P04062 | GBA | 59/114(51.75) | 0.59 | 8.88E-12 | 2.09E-10 | Yes | Up | - |
| Q96K21 | ZFYVE19 | 70/114(61.4) | 0.29 | 9.21E-06 | 3.73E-05 | Yes | Up | - |
| Q96JG9 | ZNF469 | 61/114(53.51) | 0.8 | 5.41E-10 | 7.04E-09 | Yes | Up | - |
| Q8NA03 | FSIP1 | 58/114(50.88) | 1.56 | 7.86E-14 | 3.90E-12 | Yes | Up | - |
| Q8IWA5 | SLC44A2 | 59/114(51.75) | 0.16 | 8.07E-03 | 1.60E-02 | Yes | Up | - |
| Q6ZTW0 | C19ORF20 | 68/114(59.65) | 0.3 | 1.85E-02 | 3.36E-02 | Yes | Up | - |
| Q9Y3R5 | DOPEY2 | 58/114(50.88) | 0.63 | 2.72E-07 | 1.63E-06 | Yes | Up | - |
| P42696 | RBM34 | 57/114(50.0) | 0.29 | 6.66E-06 | 2.78E-05 | Yes | Up | - |
| Q07912 | TNK2 | 60/114(52.63) | 0.23 | 5.81E-04 | 1.52E-03 | Yes | Up | - |
| Q3SXZ7 | TTLL9 | 56/114(49.12) | 0.46 | 6.36E-04 | 1.65E-03 | Yes | Up | - |
| P33981 | TTK | 59/114(51.75) | 1.25 | 6.00E-10 | 7.67E-09 | Yes | Up | - |
| Q14247 | CTTN | 57/114(50.0) | 0.36 | 2.00E-06 | 9.49E-06 | Yes | Up | - |
| O15431 | SLC31A1 | 54/114(47.37) | 0.17 | 1.96E-02 | 3.53E-02 | Yes | Up | - |
| P03372 | ESR1 | 61/114(53.51) | 0.95 | 1.82E-06 | 8.74E-06 | Yes | Up | - |
| Q53EQ6 | TIGD5 | 66/114(57.89) | 0.54 | 2.31E-07 | 1.41E-06 | Yes | Up | - |
| P61964 | WDR5 | 58/114(50.88) | 0.19 | 4.52E-04 | 1.21E-03 | Yes | Up | - |
| P54132 | BLM | 60/114(52.63) | 0.98 | 6.51E-10 | 8.24E-09 | Yes | Up | - |
| Q00975 | CACNA1B | 59/114(51.75) | 1.07 | 4.65E-07 | 2.61E-06 | Yes | Up | - |
| Q8IYK8 | REM2 | 56/114(49.12) | 0.8 | 1.68E-07 | 1.06E-06 | Yes | Up | - |
| Q96ES6 | MFSD3 | 68/114(59.65) | 0.54 | 4.01E-06 | 1.76E-05 | Yes | Up | - |
| P60900 | PSMA6 | 60/114(52.63) | 0.32 | 4.49E-05 | 1.53E-04 | Yes | Up | - |
| P05981 | HPN | 60/114(52.63) | 1.16 | 1.33E-09 | 1.53E-08 | Yes | Up | - |
| Q86YD5 | LDLRAD3 | 52/114(45.61) | 0.27 | 7.09E-03 | 1.43E-02 | Yes | Up | - |
| O14933 | UBE2L6 | 59/114(51.75) | 0.54 | 8.60E-08 | 5.87E-07 | Yes | Up | - |
| Q9P2E3 | ZNFX1 | 58/114(50.88) | 0.16 | 1.20E-02 | 2.29E-02 | Yes | Up | - |
| K7EJP2 | C19ORF26 | 62/114(54.39) | 0.55 | 1.73E-03 | 4.05E-03 | Yes | Up | - |
| - | LOC338651 | 59/114(51.75) | 0.65 | 4.77E-04 | 1.27E-03 | Yes | Up | - |
| O14828 | SCAMP3 | 63/114(55.26) | 0.55 | 6.52E-11 | 1.13E-09 | Yes | Up | - |
| O14829 | PPEF1 | 57/114(50.0) | 1.71 | 2.50E-12 | 7.01E-11 | Yes | Up | - |
| P51677 | CCR3 | 67/114(58.77) | 0.47 | 1.19E-02 | 2.25E-02 | Yes | Up | - |
| Q8NDX1 | PSD4 | 59/114(51.75) | 0.38 | 2.12E-03 | 4.84E-03 | Yes | Up | - |
| O75437 | ZNF254 | 57/114(50.0) | 0.2 | 1.50E-02 | 2.78E-02 | Yes | Up | - |
| Q8N6I4 | C14ORF109 | 54/114(47.37) | 0.16 | 3.36E-03 | 7.29E-03 | Yes | Up | - |
| O95716 | RAB3D | 60/114(52.63) | 0.57 | 2.33E-07 | 1.41E-06 | Yes | Up | - |
| Q96L94 | SNX22 | 69/114(60.53) | 0.55 | 1.36E-08 | 1.16E-07 | Yes | Up | - |
| Q14164 | IKBKE | 57/114(50.0) | 0.68 | 2.64E-10 | 3.74E-09 | Yes | Up | - |
| Q9H773 | DCTPP1 | 60/114(52.63) | 0.67 | 1.07E-11 | 2.46E-10 | Yes | Up | - |
| Q9GZP1 | NRSN2 | 67/114(58.77) | 0.47 | 1.31E-07 | 8.51E-07 | Yes | Up | - |
| - | LOC388152 | 56/114(49.12) | 0.41 | 1.33E-04 | 4.08E-04 | Yes | Up | - |
| P16403 | HIST1H1C | 64/114(56.14) | 1.11 | 3.14E-11 | 6.13E-10 | Yes | Up | - |
| Q99720 | SIGMAR1 | 70/114(61.4) | 0.17 | 9.09E-03 | 1.78E-02 | Yes | Up | - |
| Q9H790 | DEM1 | 62/114(54.39) | 0.2 | 8.27E-05 | 2.65E-04 | Yes | Up | - |
| P15153 | RAC2 | 65/114(57.02) | 0.48 | 6.35E-06 | 2.66E-05 | Yes | Up | - |
| - | LOC440356 | 54/114(47.37) | 0.86 | 7.65E-04 | 1.95E-03 | Yes | Up | - |
| Q9UHB4 | NDOR1 | 63/114(55.26) | 0.55 | 6.58E-12 | 1.62E-10 | Yes | Up | - |
| Q969F1 | GTF3C6 | 63/114(55.26) | 0.26 | 4.90E-04 | 1.30E-03 | Yes | Up | - |
| Q96Q05 | TRAPPC9 | 64/114(56.14) | 0.28 | 7.73E-04 | 1.96E-03 | Yes | Up | - |
| Q8NCW5 | APOA1BP | 63/114(55.26) | 0.72 | 2.11E-13 | 8.80E-12 | Yes | Up | - |
| Q9NRF9 | POLE3 | 54/114(47.37) | 0.19 | 1.29E-03 | 3.10E-03 | Yes | Up | - |
| P0DI80 | LOC100130933 | 59/114(51.75) | 0.52 | 1.71E-02 | 3.13E-02 | Yes | Up | - |
| Q8NFZ5 | TNIP2 | 63/114(55.26) | 0.17 | 6.35E-03 | 1.30E-02 | Yes | Up | - |
| O15047 | SETD1A | 58/114(50.88) | 0.11 | 1.77E-02 | 3.23E-02 | Yes | Up | - |
| P49798 | RGS4 | 58/114(50.88) | 0.96 | 2.05E-08 | 1.69E-07 | Yes | Up | - |
| Q3ZCM7 | TUBB8 | 65/114(57.02) | 0.92 | 4.77E-10 | 6.31E-09 | Yes | Up | - |
| Q8N680 | ZBTB2 | 59/114(51.75) | 0.15 | 3.02E-03 | 6.62E-03 | Yes | Up | - |
| P13498 | CYBA | 66/114(57.89) | 0.39 | 1.37E-04 | 4.18E-04 | Yes | Up | - |
| Q9NTK5 | OLA1 | 54/114(47.37) | 0.15 | 2.43E-02 | 4.27E-02 | Yes | Up | - |
| Q96D53 | ADCK4 | 61/114(53.51) | 0.19 | 1.22E-03 | 2.95E-03 | Yes | Up | - |
| Q96SQ5 | ZNF587 | 53/114(46.49) | 0.38 | 7.03E-04 | 1.80E-03 | Yes | Up | - |
| P25815 | S100P | 62/114(54.39) | 1.97 | 1.95E-13 | 8.25E-12 | Yes | Up | - |
| Q9BQ31 | KCNS3 | 61/114(53.51) | 0.56 | 3.63E-06 | 1.62E-05 | Yes | Up | - |
| Q8WUP2 | FBLIM1 | 61/114(53.51) | 0.28 | 2.12E-03 | 4.84E-03 | Yes | Up | - |
| Q96SI9 | STRBP | 58/114(50.88) | 0.66 | 7.88E-09 | 7.20E-08 | Yes | Up | - |
| Q8NEC7 | GSTCD | 56/114(49.12) | 0.32 | 1.46E-03 | 3.47E-03 | Yes | Up | - |
| Q96IC2 | LOC81691 | 61/114(53.51) | 0.19 | 2.38E-02 | 4.20E-02 | Yes | Up | - |
| Q9BQB4 | SOST | 48/114(42.11) | 0.92 | 2.91E-03 | 6.40E-03 | Yes | Up | - |
| Q13488 | TCIRG1 | 63/114(55.26) | 0.49 | 3.92E-07 | 2.24E-06 | Yes | Up | - |
| P55060 | CSE1L | 55/114(48.25) | 0.33 | 1.49E-04 | 4.50E-04 | Yes | Up | - |
| Q8TBP5 | FAM174A | 58/114(50.88) | 0.21 | 2.33E-03 | 5.26E-03 | Yes | Up | - |
| P31948 | STIP1 | 55/114(48.25) | 0.45 | 5.12E-07 | 2.84E-06 | Yes | Up | - |
| Q7Z3B0 | C5ORF43 | 55/114(48.25) | 0.28 | 4.03E-06 | 1.77E-05 | Yes | Up | - |
| Q9NP84 | TNFRSF12A | 72/114(63.16) | 0.7 | 1.07E-06 | 5.42E-06 | Yes | Up | - |
| Q13112 | CHAF1B | 65/114(57.02) | 0.76 | 2.18E-09 | 2.33E-08 | Yes | Up | - |
| O75783 | RHBDL1 | 56/114(49.12) | 1.49 | 1.94E-18 | 6.69E-16 | Yes | Up | - |
| Q9ULX6 | AKAP8L | 58/114(50.88) | 0.27 | 2.29E-06 | 1.07E-05 | Yes | Up | - |
| O75191 | XYLB | 47/114(41.23) | 0.27 | 6.97E-03 | 1.41E-02 | Yes | Up | - |
| Q96L08 | SUSD3 | 62/114(54.39) | 1.24 | 4.03E-12 | 1.05E-10 | Yes | Up | - |
| P55291 | CDH15 | 62/114(54.39) | 0.69 | 1.09E-03 | 2.67E-03 | Yes | Up | - |
| Q5SWH9 | TMEM69 | 53/114(46.49) | 0.11 | 2.20E-02 | 3.91E-02 | Yes | Up | - |
| Q71F23 | MLF1IP | 62/114(54.39) | 1.1 | 6.19E-12 | 1.54E-10 | Yes | Up | - |
| P41229 | KDM5C | 62/114(54.39) | 0.17 | 2.29E-04 | 6.59E-04 | Yes | Up | - |
| Q5VZY2 | PPAPDC1A | 60/114(52.63) | 2.38 | 2.52E-16 | 3.78E-14 | Yes | Up | - |
| P43235 | CTSK | 59/114(51.75) | 0.31 | 1.74E-02 | 3.19E-02 | Yes | Up | - |
| O75031 | HSF2BP | 57/114(50.0) | 0.75 | 2.17E-08 | 1.78E-07 | Yes | Up | - |
| Q9BXS6 | NUSAP1 | 60/114(52.63) | 1.46 | 7.94E-13 | 2.68E-11 | Yes | Up | - |
| P19404 | NDUFV2 | 61/114(53.51) | 0.29 | 2.76E-05 | 9.92E-05 | Yes | Up | - |
| P19419 | ELK1 | 58/114(50.88) | 0.22 | 2.48E-04 | 7.09E-04 | Yes | Up | - |
| Q9BY07 | SLC4A5 | 63/114(55.26) | 0.55 | 2.43E-08 | 1.95E-07 | Yes | Up | - |
| Q02962 | PAX2 | 60/114(52.63) | 1.44 | 3.84E-08 | 2.89E-07 | Yes | Up | - |
| Q9BXL8 | CDCA4 | 64/114(56.14) | 0.59 | 9.48E-10 | 1.14E-08 | Yes | Up | - |
| P26371 | KRTAP5-9 | 62/114(54.39) | 0.3 | 2.67E-02 | 4.65E-02 | Yes | Up | - |
| A6NNX1 | C1ORF230 | 51/114(44.74) | 1.85 | 2.57E-09 | 2.70E-08 | Yes | Up | - |
| O75752 | B3GALNT1 | 48/114(42.11) | 0.36 | 1.14E-04 | 3.55E-04 | Yes | Up | - |
| Q9Y6A5 | TACC3 | 65/114(57.02) | 1.16 | 4.16E-14 | 2.38E-12 | Yes | Up | - |
| Q96LC7 | SIGLEC10 | 66/114(57.89) | 0.42 | 2.34E-04 | 6.71E-04 | Yes | Up | - |
| Q9BY43 | CHMP4A | 58/114(50.88) | 0.15 | 1.49E-02 | 2.77E-02 | Yes | Up | - |
| Q96GX1 | TCTN2 | 58/114(50.88) | 0.25 | 7.40E-04 | 1.89E-03 | Yes | Up | - |
| Q8NE65 | ZNF738 | 57/114(50.0) | 0.2 | 1.78E-02 | 3.25E-02 | Yes | Up | - |
| Q9BSF8 | BTBD10 | 62/114(54.39) | 0.18 | 1.00E-04 | 3.15E-04 | Yes | Up | - |
| Q8WXX5 | DNAJC9 | 60/114(52.63) | 0.41 | 3.36E-07 | 1.95E-06 | Yes | Up | - |
| P63279 | UBE2I | 62/114(54.39) | 0.29 | 3.96E-07 | 2.26E-06 | Yes | Up | - |
| P09017 | HOXC4 | 57/114(50.0) | 0.27 | 1.40E-02 | 2.61E-02 | Yes | Up | - |
| Q9Y6Y8 | SEC23IP | 52/114(45.61) | 0.23 | 1.10E-03 | 2.69E-03 | Yes | Up | - |
| Q12899 | TRIM26 | 55/114(48.25) | 0.21 | 7.96E-05 | 2.56E-04 | Yes | Up | - |
| Q8IW93 | ARHGEF19 | 64/114(56.14) | 0.5 | 1.12E-05 | 4.44E-05 | Yes | Up | - |
| Q9HBE5 | IL21R | 64/114(56.14) | 1.5 | 1.60E-16 | 2.56E-14 | Yes | Up | - |
| O15399 | GRIN2D | 55/114(48.25) | 1.23 | 6.44E-16 | 7.72E-14 | Yes | Up | - |
| O00180 | KCNK1 | 61/114(53.51) | 0.74 | 2.39E-05 | 8.72E-05 | Yes | Up | - |
| B1AL88 | FAM155A | 57/114(50.0) | 0.42 | 7.47E-03 | 1.50E-02 | Yes | Up | - |
| Q6P1X6 | MGC70857 | 62/114(54.39) | 0.25 | 1.41E-03 | 3.36E-03 | Yes | Up | - |
| O75164 | KDM4A | 52/114(45.61) | 0.15 | 2.66E-03 | 5.92E-03 | Yes | Up | - |
| Q9HCN3 | TMEM8A | 64/114(56.14) | 0.61 | 8.82E-09 | 7.95E-08 | Yes | Up | - |
| Q14C87 | TMEM132D | 33/114(28.95) | 0.76 | 1.30E-02 | 2.45E-02 | Yes | Up | - |
| Q9UHK6 | AMACR | 55/114(48.25) | 0.29 | 3.43E-04 | 9.46E-04 | Yes | Up | - |
| Q5VX71 | SUSD4 | 66/114(57.89) | 0.51 | 5.14E-03 | 1.07E-02 | Yes | Up | - |
| Q9Y548 | YIPF1 | 57/114(50.0) | 0.36 | 1.23E-06 | 6.16E-06 | Yes | Up | - |
| A6NK97 | SLC22A20 | 68/114(59.65) | 0.39 | 5.80E-03 | 1.20E-02 | Yes | Up | - |
| B1AJZ9 | FHAD1 | 60/114(52.63) | 0.81 | 3.29E-09 | 3.37E-08 | Yes | Up | - |
| Q9BT30 | ALKBH7 | 69/114(60.53) | 0.25 | 2.24E-03 | 5.08E-03 | Yes | Up | - |
| Q6UXD7 | MFSD7 | 60/114(52.63) | 0.5 | 7.52E-05 | 2.43E-04 | Yes | Up | - |
| P20248 | CCNA2 | 58/114(50.88) | 1.38 | 2.29E-13 | 9.34E-12 | Yes | Up | - |
| Q8N2K1 | UBE2J2 | 60/114(52.63) | 0.2 | 5.12E-04 | 1.35E-03 | Yes | Up | - |
| Q96MF2 | STAC3 | 65/114(57.02) | 0.66 | 1.07E-07 | 7.13E-07 | Yes | Up | - |
| O95782 | AP2A1 | 58/114(50.88) | 0.22 | 1.11E-03 | 2.72E-03 | Yes | Up | - |
| P07101 | TH | 53/114(46.49) | 1.07 | 1.84E-04 | 5.44E-04 | Yes | Up | - |
| A0PJZ3 | GXYLT2 | 62/114(54.39) | 0.41 | 7.77E-04 | 1.97E-03 | Yes | Up | - |
| P46091 | GPR1 | 56/114(49.12) | 0.46 | 1.65E-02 | 3.03E-02 | Yes | Up | - |
| Q9Y463 | DYRK1B | 61/114(53.51) | 0.24 | 8.40E-04 | 2.11E-03 | Yes | Up | - |
| O75385 | ULK1 | 72/114(63.16) | 0.23 | 4.33E-05 | 1.49E-04 | Yes | Up | - |
| P24855 | DNASE1 | 62/114(54.39) | 0.41 | 9.53E-05 | 3.01E-04 | Yes | Up | - |
| P04070 | PROC | 62/114(54.39) | 1.79 | 7.03E-17 | 1.29E-14 | Yes | Up | - |
| Q9BUT9 | FAM195A | 67/114(58.77) | 0.8 | 2.39E-12 | 6.75E-11 | Yes | Up | - |
| Q9UKP3 | ITGB1BP2 | 64/114(56.14) | 0.49 | 2.65E-03 | 5.90E-03 | Yes | Up | - |
| Q0VDF9 | HSPA14 | 60/114(52.63) | 0.24 | 5.91E-04 | 1.54E-03 | Yes | Up | - |
| O95167 | NDUFA3 | 66/114(57.89) | 0.3 | 3.33E-04 | 9.21E-04 | Yes | Up | - |
| Q6WRX3 | ZYG11A | 57/114(50.0) | 1.11 | 1.48E-10 | 2.29E-09 | Yes | Up | - |
| P52565 | ARHGDIA | 58/114(50.88) | 0.3 | 6.40E-05 | 2.11E-04 | Yes | Up | - |
| Q9Y2K7 | KDM2A | 60/114(52.63) | 0.11 | 2.16E-02 | 3.84E-02 | Yes | Up | - |
| - | NCRNA00052 | 42/114(36.84) | 0.94 | 2.00E-02 | 3.59E-02 | Yes | Up | - |
| Q9H4I3 | TRABD | 65/114(57.02) | 0.4 | 4.76E-07 | 2.66E-06 | Yes | Up | - |
| Q15633 | TARBP2 | 62/114(54.39) | 0.35 | 1.43E-08 | 1.22E-07 | Yes | Up | - |
| Q8N5M4 | TTC9C | 57/114(50.0) | 0.21 | 1.12E-04 | 3.48E-04 | Yes | Up | - |
| Q96DB2 | HDAC11 | 68/114(59.65) | 0.43 | 8.63E-07 | 4.52E-06 | Yes | Up | - |
| Q9UBN1 | CACNG4 | 60/114(52.63) | 1.29 | 1.94E-08 | 1.61E-07 | Yes | Up | - |
| - | FLJ90757 | 57/114(50.0) | 0.24 | 2.08E-03 | 4.76E-03 | Yes | Up | - |
| P55273 | CDKN2D | 57/114(50.0) | 0.66 | 4.70E-09 | 4.60E-08 | Yes | Up | - |
| P0C2W1 | FBXO45 | 59/114(51.75) | 0.39 | 4.04E-06 | 1.77E-05 | Yes | Up | - |
| O75955 | FLOT1 | 61/114(53.51) | 0.21 | 5.07E-04 | 1.34E-03 | Yes | Up | - |
| - | LOC606724 | 62/114(54.39) | 0.37 | 4.56E-03 | 9.60E-03 | Yes | Up | - |
| Q99706 | KIR2DL4 | 55/114(48.25) | 0.81 | 9.82E-04 | 2.43E-03 | Yes | Up | - |
| Q5T1B0 | C1ORF125 | 52/114(45.61) | 0.86 | 7.08E-05 | 2.31E-04 | Yes | Up | - |
| P18827 | SDC1 | 64/114(56.14) | 1.15 | 1.17E-09 | 1.37E-08 | Yes | Up | - |
| A6NK44 | GLOD5 | 55/114(48.25) | 0.56 | 3.68E-03 | 7.91E-03 | Yes | Up | - |
| - | FLJ35776 | 69/114(60.53) | 0.23 | 1.58E-03 | 3.73E-03 | Yes | Up | - |
| P31941 | APOBEC3A | 65/114(57.02) | 0.91 | 2.10E-07 | 1.29E-06 | Yes | Up | - |
| O00445 | SYT5 | 50/114(43.86) | 0.63 | 1.91E-02 | 3.45E-02 | Yes | Up | - |
| O14569 | CYB561D2 | 66/114(57.89) | 0.55 | 3.96E-09 | 3.95E-08 | Yes | Up | - |
| O60344 | ECE2 | 62/114(54.39) | 0.88 | 1.08E-10 | 1.74E-09 | Yes | Up | - |
| Q8NHM4 | TRY6 | 29/114(25.44) | 3.1 | 1.34E-10 | 2.09E-09 | Yes | Up | - |
| P55327 | TPD52 | 57/114(50.0) | 0.93 | 1.48E-08 | 1.25E-07 | Yes | Up | - |
| Q5TAT6 | COL13A1 | 55/114(48.25) | 0.48 | 6.18E-04 | 1.61E-03 | Yes | Up | - |
| Q14696 | MESDC2 | 53/114(46.49) | 0.23 | 4.98E-06 | 2.14E-05 | Yes | Up | - |
| Q9HB09 | BCL2L12 | 71/114(62.28) | 0.57 | 1.35E-10 | 2.11E-09 | Yes | Up | - |
| Q9H8X2 | IPPK | 58/114(50.88) | 0.42 | 7.94E-08 | 5.49E-07 | Yes | Up | - |
| P29322 | EPHA8 | 55/114(48.25) | 2.14 | 1.01E-14 | 7.34E-13 | Yes | Up | - |
| P49711 | CTCF | 57/114(50.0) | 0.08 | 1.91E-02 | 3.46E-02 | Yes | Up | - |
| O95707 | POP4 | 59/114(51.75) | 0.16 | 5.20E-03 | 1.08E-02 | Yes | Up | - |
| Q3YEC7 | C9ORF86 | 66/114(57.89) | 0.25 | 5.35E-04 | 1.41E-03 | Yes | Up | - |
| Q9Y3A4 | RRP7A | 63/114(55.26) | 0.16 | 1.03E-02 | 1.99E-02 | Yes | Up | - |
| Q969S3 | ZNF622 | 62/114(54.39) | 0.2 | 1.19E-04 | 3.68E-04 | Yes | Up | - |
| P53609 | PGGT1B | 55/114(48.25) | 0.17 | 4.32E-03 | 9.14E-03 | Yes | Up | - |
| P05107 | ITGB2 | 67/114(58.77) | 0.41 | 1.36E-04 | 4.15E-04 | Yes | Up | - |
| P56747 | CLDN6 | 47/114(41.23) | 0.64 | 1.44E-02 | 2.68E-02 | Yes | Up | - |
| Q9NSC5 | HOMER3 | 63/114(55.26) | 0.59 | 7.50E-10 | 9.27E-09 | Yes | Up | - |
| Q13445 | TMED1 | 68/114(59.65) | 0.27 | 2.32E-04 | 6.67E-04 | Yes | Up | - |
| Q99607 | ELF4 | 58/114(50.88) | 0.33 | 2.91E-05 | 1.04E-04 | Yes | Up | - |
| P07476 | IVL | 47/114(41.23) | 1.15 | 2.39E-04 | 6.85E-04 | Yes | Up | - |
| Q8TEY5 | CREB3L4 | 60/114(52.63) | 0.91 | 8.44E-11 | 1.41E-09 | Yes | Up | - |
| K0A6P4 | C20ORF54 | 62/114(54.39) | 0.88 | 1.88E-11 | 3.96E-10 | Yes | Up | - |
| Q9HD20 | ATP13A1 | 61/114(53.51) | 0.48 | 2.89E-11 | 5.74E-10 | Yes | Up | - |
| Q9P2W1 | PSMC3IP | 64/114(56.14) | 0.5 | 8.84E-07 | 4.61E-06 | Yes | Up | - |
| Q9H6R6 | ZDHHC6 | 52/114(45.61) | 0.18 | 8.27E-05 | 2.65E-04 | Yes | Up | - |
| Q96GD4 | AURKB | 57/114(50.0) | 1.54 | 6.72E-13 | 2.32E-11 | Yes | Up | - |
| Q8N8Q9 | NIPA2 | 63/114(55.26) | 0.15 | 1.09E-02 | 2.09E-02 | Yes | Up | - |
| Q96DB9 | FXYD5 | 57/114(50.0) | 0.3 | 2.00E-03 | 4.60E-03 | Yes | Up | - |
| A2BFH1 | PPIAL4G | 65/114(57.02) | 0.39 | 2.85E-06 | 1.30E-05 | Yes | Up | - |
| O60831 | PRAF2 | 63/114(55.26) | 0.5 | 5.40E-08 | 3.90E-07 | Yes | Up | - |
| Q8NBT0 | POC1A | 62/114(54.39) | 1.11 | 1.41E-14 | 9.80E-13 | Yes | Up | - |
| P52951 | GBX2 | 46/114(40.35) | 1.51 | 3.43E-06 | 1.54E-05 | Yes | Up | - |
| Q9NZC9 | SMARCAL1 | 54/114(47.37) | 0.14 | 5.02E-05 | 1.70E-04 | Yes | Up | - |
| O15394 | NCAM2 | 58/114(50.88) | 0.69 | 3.99E-05 | 1.38E-04 | Yes | Up | - |
| - | C10ORF93 | 61/114(53.51) | 0.46 | 2.63E-02 | 4.59E-02 | Yes | Up | - |
| Q7Z7F7 | MRPL55 | 65/114(57.02) | 0.51 | 1.10E-09 | 1.29E-08 | Yes | Up | - |
| Q16650 | TBR1 | 31/114(27.19) | 0.99 | 7.92E-03 | 1.58E-02 | Yes | Up | - |
| O00214 | LGALS8 | 58/114(50.88) | 0.21 | 1.50E-02 | 2.79E-02 | Yes | Up | - |
| Q9H9A5 | CNOT10 | 60/114(52.63) | 0.15 | 3.20E-03 | 6.98E-03 | Yes | Up | - |
| Q68D85 | DKFZP686O24166 | 52/114(45.61) | 0.39 | 1.02E-02 | 1.98E-02 | Yes | Up | - |
| O75396 | SEC22B | 53/114(46.49) | 0.19 | 1.08E-02 | 2.07E-02 | Yes | Up | - |
| Q9NRM6 | IL17RB | 51/114(44.74) | 0.4 | 4.71E-03 | 9.88E-03 | Yes | Up | - |
| - | PP14571 | 53/114(46.49) | 1.86 | 3.04E-18 | 9.47E-16 | Yes | Up | - |
| Q6ZN44 | UNC5A | 57/114(50.0) | 1.01 | 1.92E-07 | 1.19E-06 | Yes | Up | - |
| Q9UGB7 | MIOX | 37/114(32.46) | 0.86 | 5.56E-03 | 1.15E-02 | Yes | Up | - |
| Q9UBE0 | SAE1 | 64/114(56.14) | 0.34 | 3.60E-07 | 2.08E-06 | Yes | Up | - |
| Q96KP4 | CNDP2 | 57/114(50.0) | 0.28 | 2.22E-05 | 8.16E-05 | Yes | Up | - |
| Q8N5U6 | RNF10 | 56/114(49.12) | 0.11 | 1.38E-02 | 2.59E-02 | Yes | Up | - |
| P61225 | RAP2B | 57/114(50.0) | 0.2 | 3.72E-03 | 7.98E-03 | Yes | Up | - |
| Q6ZRV2 | FAM83H | 64/114(56.14) | 0.89 | 3.41E-08 | 2.61E-07 | Yes | Up | - |
| P08243 | ASNS | 57/114(50.0) | 0.29 | 6.54E-03 | 1.33E-02 | Yes | Up | - |
| Q9HC07 | TMEM165 | 57/114(50.0) | 0.33 | 3.25E-05 | 1.15E-04 | Yes | Up | - |
| Q9H3Y0 | R3HDML | 26/114(22.81) | 1.66 | 1.08E-05 | 4.30E-05 | Yes | Up | - |
| P07237 | P4HB | 60/114(52.63) | 0.42 | 1.61E-06 | 7.80E-06 | Yes | Up | - |
| A0PJK1 | SLC5A10 | 62/114(54.39) | 0.72 | 9.77E-05 | 3.08E-04 | Yes | Up | - |
| P33681 | CD80 | 60/114(52.63) | 1.21 | 3.47E-12 | 9.32E-11 | Yes | Up | - |
| Q9UKG9 | CROT | 61/114(53.51) | 0.25 | 2.67E-02 | 4.64E-02 | Yes | Up | - |
| Q8IXQ6 | PARP9 | 58/114(50.88) | 0.54 | 6.76E-08 | 4.75E-07 | Yes | Up | - |
| P56192 | MARS | 61/114(53.51) | 0.36 | 5.14E-08 | 3.73E-07 | Yes | Up | - |
| Q9NYV6 | RRN3 | 59/114(51.75) | 0.17 | 6.19E-03 | 1.27E-02 | Yes | Up | - |
| P49848 | TAF6 | 62/114(54.39) | 0.28 | 8.69E-08 | 5.93E-07 | Yes | Up | - |
| O15169 | AXIN1 | 61/114(53.51) | 0.42 | 2.28E-08 | 1.84E-07 | Yes | Up | - |
| Q16696 | CYP2A13 | 36/114(31.58) | 1.28 | 1.96E-03 | 4.52E-03 | Yes | Up | - |
| Q9H8H2 | DDX31 | 59/114(51.75) | 0.2 | 1.37E-03 | 3.28E-03 | Yes | Up | - |
| Q9H7T3 | C10ORF95 | 52/114(45.61) | 0.77 | 5.07E-08 | 3.68E-07 | Yes | Up | - |
| P31213 | SRD5A2 | 59/114(51.75) | 1.16 | 1.08E-08 | 9.47E-08 | Yes | Up | - |
| Q8NI77 | KIF18A | 58/114(50.88) | 1.18 | 1.07E-10 | 1.73E-09 | Yes | Up | - |
| Q8IYX4 | DND1 | 63/114(55.26) | 0.3 | 1.74E-05 | 6.60E-05 | Yes | Up | - |
| P18510 | IL1RN | 63/114(55.26) | 0.52 | 1.64E-04 | 4.91E-04 | Yes | Up | - |
| Q8WUY9 | DEPDC1B | 59/114(51.75) | 1.37 | 4.57E-11 | 8.35E-10 | Yes | Up | - |
| Q9P0R6 | C14ORF129 | 56/114(49.12) | 0.25 | 6.29E-04 | 1.63E-03 | Yes | Up | - |
| Q9NVU0 | POLR3E | 59/114(51.75) | 0.17 | 3.20E-03 | 6.97E-03 | Yes | Up | - |
| P47736 | RAP1GAP | 51/114(44.74) | 0.4 | 2.11E-02 | 3.76E-02 | Yes | Up | - |
| Q92623 | TTC9 | 56/114(49.12) | 0.37 | 4.92E-03 | 1.03E-02 | Yes | Up | - |
| P10242 | MYB | 55/114(48.25) | 0.75 | 9.39E-05 | 2.98E-04 | Yes | Up | - |
| P62318 | SNRPD3 | 54/114(47.37) | 0.28 | 1.58E-05 | 6.05E-05 | Yes | Up | - |
| P22466 | GAL | 68/114(59.65) | 0.87 | 3.05E-05 | 1.09E-04 | Yes | Up | - |
| P10072 | HKR1 | 58/114(50.88) | 0.19 | 5.02E-03 | 1.05E-02 | Yes | Up | - |
| Q8N3Y1 | FBXW8 | 53/114(46.49) | 0.12 | 2.32E-02 | 4.11E-02 | Yes | Up | - |
| P0C0L4 | C4A | 60/114(52.63) | 0.83 | 1.03E-05 | 4.11E-05 | Yes | Up | - |
| Q9NRF8 | CTPS2 | 53/114(46.49) | 0.39 | 1.55E-05 | 5.95E-05 | Yes | Up | - |
| O00170 | AIP | 60/114(52.63) | 0.18 | 1.05E-02 | 2.03E-02 | Yes | Up | - |
| Q56VL3 | OCIAD2 | 61/114(53.51) | 0.46 | 1.12E-06 | 5.66E-06 | Yes | Up | - |
| Q99829 | CPNE1 | 59/114(51.75) | 0.25 | 2.17E-04 | 6.29E-04 | Yes | Up | - |
| Q9NX55 | C15ORF63 | 65/114(57.02) | 0.4 | 3.62E-06 | 1.61E-05 | Yes | Up | - |
| Q9BV94 | EDEM2 | 63/114(55.26) | 0.28 | 2.64E-06 | 1.22E-05 | Yes | Up | - |
| Q8NCG7 | DAGLB | 62/114(54.39) | 0.26 | 5.73E-07 | 3.14E-06 | Yes | Up | - |
| Q9H0F6 | SHARPIN | 64/114(56.14) | 0.43 | 3.41E-07 | 1.98E-06 | Yes | Up | - |
| - | LOC399815 | 59/114(51.75) | 0.91 | 5.59E-08 | 4.01E-07 | Yes | Up | - |
| Q96S19 | C16ORF13 | 67/114(58.77) | 0.56 | 2.19E-10 | 3.18E-09 | Yes | Up | - |
| Q96HV5 | TMEM41A | 61/114(53.51) | 0.31 | 1.23E-06 | 6.15E-06 | Yes | Up | - |
| Q9BXW9 | FANCD2 | 63/114(55.26) | 0.72 | 2.49E-07 | 1.50E-06 | Yes | Up | - |
| P05121 | SERPINE1 | 68/114(59.65) | 0.87 | 8.91E-10 | 1.08E-08 | Yes | Up | - |
| Q8N1G0 | ZNF687 | 57/114(50.0) | 0.55 | 9.62E-11 | 1.58E-09 | Yes | Up | - |
| P49841 | GSK3B | 52/114(45.61) | 0.14 | 1.86E-02 | 3.36E-02 | Yes | Up | - |
| Q9BY50 | SEC11C | 60/114(52.63) | 0.36 | 2.39E-05 | 8.75E-05 | Yes | Up | - |
| Q9UI95 | MAD2L2 | 65/114(57.02) | 0.39 | 3.00E-07 | 1.77E-06 | Yes | Up | - |
| Q9UKU0 | ACSL6 | 62/114(54.39) | 0.69 | 4.75E-05 | 1.61E-04 | Yes | Up | - |
| P63000 | RAC1 | 61/114(53.51) | 0.09 | 2.31E-02 | 4.08E-02 | Yes | Up | - |
| Q9HCH3 | CPNE5 | 71/114(62.28) | 0.34 | 3.97E-03 | 8.47E-03 | Yes | Up | - |
| Q8NCW0 | KREMEN2 | 63/114(55.26) | 1.8 | 4.99E-16 | 6.34E-14 | Yes | Up | - |
| Q96A70 | ADC | 65/114(57.02) | 0.45 | 2.51E-08 | 2.00E-07 | Yes | Up | - |
| Q9NW61 | PLEKHJ1 | 64/114(56.14) | 0.19 | 9.67E-03 | 1.88E-02 | Yes | Up | - |
| Q96MH2 | HEXIM2 | 68/114(59.65) | 0.47 | 2.93E-06 | 1.34E-05 | Yes | Up | - |
| O14578 | CIT | 64/114(56.14) | 1.07 | 3.04E-12 | 8.35E-11 | Yes | Up | - |
| Q9Y2R0 | CCDC56 | 60/114(52.63) | 0.24 | 8.68E-05 | 2.77E-04 | Yes | Up | - |
| Q01844 | EWSR1 | 61/114(53.51) | 0.09 | 1.58E-02 | 2.91E-02 | Yes | Up | - |
| Q6XYB7 | LBX2 | 70/114(61.4) | 0.55 | 1.57E-04 | 4.72E-04 | Yes | Up | - |
| O43257 | ZNHIT1 | 66/114(57.89) | 0.14 | 2.62E-02 | 4.57E-02 | Yes | Up | - |
| Q8WXE1 | ATRIP | 58/114(50.88) | 0.34 | 6.26E-07 | 3.39E-06 | Yes | Up | - |
| Q86T82 | USP37 | 60/114(52.63) | 0.24 | 2.60E-03 | 5.79E-03 | Yes | Up | - |
| Q9UJA3 | MCM8 | 52/114(45.61) | 0.21 | 2.37E-02 | 4.19E-02 | Yes | Up | - |
| P29692 | EEF1D | 69/114(60.53) | 0.24 | 1.96E-03 | 4.52E-03 | Yes | Up | - |
| O15143 | ARPC1B | 66/114(57.89) | 0.57 | 1.73E-08 | 1.45E-07 | Yes | Up | - |
| Q9BTT6 | LRRC1 | 53/114(46.49) | 0.48 | 3.22E-05 | 1.14E-04 | Yes | Up | - |
| Q9UBV7 | B4GALT7 | 68/114(59.65) | 0.24 | 1.18E-04 | 3.66E-04 | Yes | Up | - |
| Q9Y4P1 | ATG4B | 58/114(50.88) | 0.11 | 1.91E-02 | 3.46E-02 | Yes | Up | - |
| Q9H6H4 | REEP4 | 66/114(57.89) | 0.8 | 5.69E-16 | 7.05E-14 | Yes | Up | - |
| Q96QU8 | XPO6 | 58/114(50.88) | 0.25 | 2.53E-05 | 9.19E-05 | Yes | Up | - |
| Q9NX46 | ADPRHL2 | 68/114(59.65) | 0.3 | 8.19E-07 | 4.31E-06 | Yes | Up | - |
| P32238 | CCKAR | 44/114(38.6) | 1.13 | 1.15E-04 | 3.57E-04 | Yes | Up | - |
| Q96J66 | ABCC11 | 61/114(53.51) | 0.72 | 2.97E-03 | 6.53E-03 | Yes | Up | - |
| Q8WZ79 | DNASE2B | 55/114(48.25) | 0.76 | 5.98E-04 | 1.56E-03 | Yes | Up | - |
| O96020 | CCNE2 | 60/114(52.63) | 1.26 | 4.00E-11 | 7.51E-10 | Yes | Up | - |
| Q96PN7 | TRERF1 | 54/114(47.37) | 0.38 | 1.30E-03 | 3.13E-03 | Yes | Up | - |
| Q8NCT3 | KIAA0895 | 57/114(50.0) | 0.48 | 2.32E-05 | 8.50E-05 | Yes | Up | - |
| Q4G0X9 | CCDC40 | 63/114(55.26) | 0.51 | 4.66E-05 | 1.59E-04 | Yes | Up | - |
| Q9Y394 | DHRS7 | 60/114(52.63) | 0.22 | 6.40E-04 | 1.66E-03 | Yes | Up | - |
| Q9Y328 | HMP19 | 59/114(51.75) | 0.59 | 2.36E-02 | 4.16E-02 | Yes | Up | - |
| Q9H5J4 | ELOVL6 | 51/114(44.74) | 0.29 | 2.31E-02 | 4.09E-02 | Yes | Up | - |
| Q68D42 | TMEM215 | 55/114(48.25) | 2.11 | 1.29E-14 | 9.05E-13 | Yes | Up | - |
| - | HLA-DRB6 | 65/114(57.02) | 0.27 | 1.15E-02 | 2.20E-02 | Yes | Up | - |
| Q9NZG7 | NINJ2 | 59/114(51.75) | 0.39 | 2.94E-03 | 6.46E-03 | Yes | Up | - |
| Q53RE8 | ANKRD39 | 59/114(51.75) | 0.34 | 3.17E-06 | 1.43E-05 | Yes | Up | - |
| P56279 | TCL1A | 58/114(50.88) | 1.13 | 1.02E-05 | 4.09E-05 | Yes | Up | - |
| Q96S90 | LYSMD1 | 58/114(50.88) | 0.28 | 4.26E-04 | 1.15E-03 | Yes | Up | - |
| Q86T20 | C6ORF1 | 59/114(51.75) | 0.55 | 1.96E-10 | 2.90E-09 | Yes | Up | - |
| Q9H4A4 | RNPEP | 65/114(57.02) | 0.53 | 1.48E-09 | 1.68E-08 | Yes | Up | - |
| P51798 | CLCN7 | 63/114(55.26) | 0.4 | 2.62E-08 | 2.07E-07 | Yes | Up | - |
| C9JVW0 | PRR24 | 67/114(58.77) | 0.3 | 1.09E-04 | 3.41E-04 | Yes | Up | - |
| P61604 | HSPE1 | 62/114(54.39) | 0.49 | 3.71E-08 | 2.80E-07 | Yes | Up | - |
| Q86XI2 | NCAPG2 | 58/114(50.88) | 0.46 | 9.52E-06 | 3.84E-05 | Yes | Up | - |
| P13667 | PDIA4 | 61/114(53.51) | 0.61 | 1.47E-11 | 3.18E-10 | Yes | Up | - |
| Q8N5A5 | ZGPAT | 69/114(60.53) | 0.29 | 2.76E-04 | 7.78E-04 | Yes | Up | - |
| Q92504 | SLC39A7 | 62/114(54.39) | 0.47 | 1.10E-07 | 7.29E-07 | Yes | Up | - |
| Q8WV22 | NSMCE1 | 66/114(57.89) | 0.29 | 1.01E-06 | 5.19E-06 | Yes | Up | - |
| Q8IUE6 | HIST2H2AB | 45/114(39.47) | 0.85 | 4.88E-03 | 1.02E-02 | Yes | Up | - |
| O75414 | NME6 | 60/114(52.63) | 0.2 | 4.41E-04 | 1.19E-03 | Yes | Up | - |
| O75123 | ZNF623 | 56/114(49.12) | 0.28 | 3.38E-03 | 7.32E-03 | Yes | Up | - |
| P02763 | ORM1 | 52/114(45.61) | 1.34 | 5.99E-06 | 2.53E-05 | Yes | Up | - |
| O95471 | CLDN7 | 56/114(49.12) | 0.71 | 4.46E-06 | 1.94E-05 | Yes | Up | - |
| Q9UBP6 | METTL1 | 62/114(54.39) | 0.43 | 1.17E-08 | 1.02E-07 | Yes | Up | - |
| Q8N443 | RIBC1 | 62/114(54.39) | 0.57 | 1.19E-06 | 5.97E-06 | Yes | Up | - |
| P50336 | PPOX | 68/114(59.65) | 0.42 | 7.61E-10 | 9.40E-09 | Yes | Up | - |
| Q8NA69 | C19ORF45 | 63/114(55.26) | 1.29 | 1.53E-10 | 2.35E-09 | Yes | Up | - |
| Q66GS9 | CEP135 | 59/114(51.75) | 0.15 | 1.84E-02 | 3.34E-02 | Yes | Up | - |
| P40937 | RFC5 | 55/114(48.25) | 0.33 | 9.20E-06 | 3.73E-05 | Yes | Up | - |
| Q9UHY1 | NRBP1 | 61/114(53.51) | 0.25 | 7.40E-07 | 3.93E-06 | Yes | Up | - |
| Q9BVC4 | MLST8 | 64/114(56.14) | 0.43 | 2.41E-09 | 2.54E-08 | Yes | Up | - |
| O75762 | TRPA1 | 66/114(57.89) | 2.41 | 1.29E-23 | 2.50E-20 | Yes | Up | - |
| Q96KG9 | SCYL1 | 59/114(51.75) | 0.2 | 5.98E-04 | 1.56E-03 | Yes | Up | - |
| Q9H477 | RBKS | 66/114(57.89) | 0.35 | 1.61E-04 | 4.81E-04 | Yes | Up | - |
| Q9UI14 | RABAC1 | 69/114(60.53) | 0.24 | 3.38E-03 | 7.32E-03 | Yes | Up | - |
| Q9H665 | TMEM149 | 61/114(53.51) | 0.49 | 3.86E-08 | 2.90E-07 | Yes | Up | - |
| - | RPLP0P2 | 57/114(50.0) | 1.26 | 3.14E-14 | 1.90E-12 | Yes | Up | - |
| Q3SY56 | SP6 | 60/114(52.63) | 0.35 | 1.15E-02 | 2.19E-02 | Yes | Up | - |
| P08185 | SERPINA6 | 62/114(54.39) | 1.28 | 3.46E-05 | 1.22E-04 | Yes | Up | - |
| Q9H9R9 | DBNDD1 | 64/114(56.14) | 0.88 | 3.76E-09 | 3.77E-08 | Yes | Up | - |
| O75690 | KRTAP5-8 | 58/114(50.88) | 0.48 | 1.17E-02 | 2.23E-02 | Yes | Up | - |
| O60512 | B4GALT3 | 63/114(55.26) | 0.71 | 3.34E-14 | 2.00E-12 | Yes | Up | - |
| Q9UGQ2 | C9ORF7 | 68/114(59.65) | 0.82 | 8.10E-13 | 2.72E-11 | Yes | Up | - |
| P31150 | GDI1 | 64/114(56.14) | 0.31 | 5.20E-07 | 2.88E-06 | Yes | Up | - |
| P31350 | RRM2 | 57/114(50.0) | 1.57 | 1.48E-12 | 4.47E-11 | Yes | Up | - |
| Q5U623 | ATF7IP2 | 66/114(57.89) | 0.27 | 7.33E-03 | 1.47E-02 | Yes | Up | - |
| P17568 | NDUFB7 | 63/114(55.26) | 0.26 | 1.93E-03 | 4.45E-03 | Yes | Up | - |
| P05161 | ISG15 | 60/114(52.63) | 1.65 | 3.97E-19 | 1.57E-16 | Yes | Up | - |
| P21854 | CD72 | 61/114(53.51) | 0.49 | 1.29E-05 | 5.04E-05 | Yes | Up | - |
| P24311 | COX7B | 56/114(49.12) | 0.18 | 2.23E-02 | 3.96E-02 | Yes | Up | - |
| - | RAET1K | 57/114(50.0) | 1.02 | 3.25E-06 | 1.46E-05 | Yes | Up | - |
| P34903 | GABRA3 | 37/114(32.46) | 0.98 | 6.31E-03 | 1.29E-02 | Yes | Up | - |
| Q8NC42 | RNF149 | 67/114(58.77) | 0.29 | 6.69E-07 | 3.60E-06 | Yes | Up | - |
| Q6ZMN8 | CCNI2 | 63/114(55.26) | 0.95 | 1.16E-08 | 1.01E-07 | Yes | Up | - |
| P51858 | HDGF | 63/114(55.26) | 0.57 | 5.34E-09 | 5.13E-08 | Yes | Up | - |
| P08579 | SNRPB2 | 63/114(55.26) | 0.17 | 4.96E-03 | 1.04E-02 | Yes | Up | - |
| Q8IXA5 | SPACA3 | 20/114(17.54) | 0.93 | 2.14E-02 | 3.81E-02 | Yes | Up | - |
| Q9BWF3 | RBM4 | 58/114(50.88) | 0.23 | 3.84E-05 | 1.34E-04 | Yes | Up | - |
| Q9BSM1 | PCGF1 | 59/114(51.75) | 0.15 | 4.04E-03 | 8.61E-03 | Yes | Up | - |
| Q96QK8 | C4ORF34 | 55/114(48.25) | 0.29 | 2.62E-03 | 5.84E-03 | Yes | Up | - |
| - | C14ORF34 | 49/114(42.98) | 1.19 | 2.45E-05 | 8.94E-05 | Yes | Up | - |
| O00115 | DNASE2 | 56/114(49.12) | 0.55 | 1.26E-12 | 3.91E-11 | Yes | Up | - |
| Q6EIG7 | CLEC6A | 38/114(33.33) | 0.89 | 5.20E-03 | 1.08E-02 | Yes | Up | - |
| O14625 | CXCL11 | 58/114(50.88) | 1.71 | 1.18E-14 | 8.35E-13 | Yes | Up | - |
| P62333 | PSMC6 | 54/114(47.37) | 0.12 | 1.97E-02 | 3.55E-02 | Yes | Up | - |
| Q8TE85 | GRHL3 | 57/114(50.0) | 0.65 | 5.07E-04 | 1.34E-03 | Yes | Up | - |
| Q9NYG5 | ANAPC11 | 64/114(56.14) | 0.5 | 3.55E-08 | 2.70E-07 | Yes | Up | - |
| Q9NP64 | ZCCHC17 | 59/114(51.75) | 0.13 | 6.00E-03 | 1.23E-02 | Yes | Up | - |
| O76013 | KRT36 | 51/114(44.74) | 0.67 | 2.59E-03 | 5.78E-03 | Yes | Up | - |
| Q9BST9 | RTKN | 56/114(49.12) | 0.57 | 9.30E-09 | 8.34E-08 | Yes | Up | - |
| O95834 | EML2 | 64/114(56.14) | 0.41 | 1.47E-06 | 7.21E-06 | Yes | Up | - |
| Q96FV9 | THOC1 | 58/114(50.88) | 0.15 | 7.16E-03 | 1.44E-02 | Yes | Up | - |
| P01111 | NRAS | 49/114(42.98) | 0.21 | 9.80E-04 | 2.42E-03 | Yes | Up | - |
| Q96H72 | SLC39A13 | 62/114(54.39) | 0.18 | 2.72E-03 | 6.03E-03 | Yes | Up | - |
| Q6UWJ1 | TMCO3 | 47/114(41.23) | 0.25 | 1.20E-03 | 2.91E-03 | Yes | Up | - |
| P42771 | CDKN2A | 63/114(55.26) | 0.85 | 2.10E-08 | 1.72E-07 | Yes | Up | - |
| P13521 | SCG2 | 60/114(52.63) | 0.98 | 1.01E-07 | 6.77E-07 | Yes | Up | - |
| - | LOC84740 | 57/114(50.0) | 0.77 | 7.17E-05 | 2.33E-04 | Yes | Up | - |
| Q96LS8 | C2ORF48 | 55/114(48.25) | 1.01 | 2.68E-05 | 9.66E-05 | Yes | Up | - |
| Q96PQ5 | PPP1R2P1 | 66/114(57.89) | 0.28 | 1.28E-02 | 2.41E-02 | Yes | Up | - |
| Q5MY95 | ENTPD8 | 54/114(47.37) | 1.24 | 6.02E-09 | 5.69E-08 | Yes | Up | - |
| Q5EBL8 | PDZD11 | 59/114(51.75) | 0.4 | 2.37E-07 | 1.44E-06 | Yes | Up | - |
| O00762 | UBE2C | 62/114(54.39) | 1.85 | 1.69E-15 | 1.71E-13 | Yes | Up | - |
| Q9UNZ5 | C19ORF53 | 67/114(58.77) | 0.41 | 4.34E-09 | 4.28E-08 | Yes | Up | - |
| Q96I45 | TMEM141 | 64/114(56.14) | 0.65 | 7.44E-12 | 1.79E-10 | Yes | Up | - |
| Q96IZ6 | METTL2A | 51/114(44.74) | 0.24 | 2.78E-04 | 7.84E-04 | Yes | Up | - |
| P0CG08 | GPR89B | 49/114(42.98) | 0.42 | 1.35E-03 | 3.23E-03 | Yes | Up | - |
| Q06187 | BTK | 60/114(52.63) | 0.25 | 1.46E-02 | 2.72E-02 | Yes | Up | - |
| Q6ZMZ0 | RNF19B | 63/114(55.26) | 0.27 | 1.80E-06 | 8.64E-06 | Yes | Up | - |
| P17947 | SPI1 | 65/114(57.02) | 0.39 | 4.83E-04 | 1.29E-03 | Yes | Up | - |
| Q92598 | HSPH1 | 57/114(50.0) | 0.41 | 1.00E-04 | 3.15E-04 | Yes | Up | - |
| Q15853 | USF2 | 68/114(59.65) | 0.18 | 7.20E-03 | 1.45E-02 | Yes | Up | - |
| Q9UBY0 | SLC9A2 | 65/114(57.02) | 0.66 | 3.97E-03 | 8.47E-03 | Yes | Up | - |
| Q9UBR5 | CKLF | 60/114(52.63) | 0.31 | 3.56E-06 | 1.59E-05 | Yes | Up | - |
| Q9NYJ1 | CHCHD8 | 65/114(57.02) | 0.34 | 7.38E-07 | 3.92E-06 | Yes | Up | - |
| P63167 | DYNLL1 | 64/114(56.14) | 0.28 | 5.05E-06 | 2.17E-05 | Yes | Up | - |
| Q6UWQ7 | IGFL2 | 56/114(49.12) | 1.54 | 1.24E-07 | 8.09E-07 | Yes | Up | - |
| Q6L8H4 | KRTAP5-1 | 63/114(55.26) | 0.62 | 2.70E-04 | 7.65E-04 | Yes | Up | - |
| Q9NRX1 | PNO1 | 55/114(48.25) | 0.19 | 2.81E-03 | 6.21E-03 | Yes | Up | - |
| Q9UI43 | FTSJ2 | 54/114(47.37) | 0.23 | 7.28E-06 | 3.02E-05 | Yes | Up | - |
| Q92888 | ARHGEF1 | 68/114(59.65) | 0.14 | 2.82E-02 | 4.89E-02 | Yes | Up | - |
| Q5T1B1 | C10ORF91 | 53/114(46.49) | 0.8 | 5.75E-04 | 1.51E-03 | Yes | Up | - |
| P59901 | LILRA4 | 61/114(53.51) | 0.68 | 3.06E-04 | 8.56E-04 | Yes | Up | - |
| Q9NZM4 | GLTSCR1 | 68/114(59.65) | 0.27 | 1.51E-06 | 7.39E-06 | Yes | Up | - |
| Q9H8M1 | COQ10B | 63/114(55.26) | 0.13 | 1.52E-02 | 2.82E-02 | Yes | Up | - |
| Q5BVD1 | C3ORF52 | 62/114(54.39) | 1.01 | 7.26E-11 | 1.24E-09 | Yes | Up | - |
| P36776 | LONP1 | 71/114(62.28) | 0.26 | 1.68E-03 | 3.93E-03 | Yes | Up | - |
| Q15329 | E2F5 | 55/114(48.25) | 0.51 | 7.57E-05 | 2.45E-04 | Yes | Up | - |
| Q8N257 | HIST3H2BB | 59/114(51.75) | 1.35 | 3.02E-09 | 3.11E-08 | Yes | Up | - |
| O95178 | NDUFB2 | 64/114(56.14) | 0.29 | 1.43E-04 | 4.34E-04 | Yes | Up | - |
| Q86VR2 | FAM134C | 60/114(52.63) | 0.13 | 8.25E-03 | 1.63E-02 | Yes | Up | - |
| Q9BRB3 | PIGQ | 68/114(59.65) | 0.55 | 8.95E-10 | 1.08E-08 | Yes | Up | - |
| Q6IWH7 | ANO7 | 62/114(54.39) | 0.52 | 3.53E-05 | 1.24E-04 | Yes | Up | - |
| P01215 | CGA | 50/114(43.86) | 3.89 | 5.09E-25 | 1.64E-21 | Yes | Up | - |
| Q96BM9 | ARL8A | 61/114(53.51) | 0.3 | 5.34E-07 | 2.95E-06 | Yes | Up | - |
| Q15418 | RPS6KA1 | 61/114(53.51) | 0.52 | 4.28E-08 | 3.18E-07 | Yes | Up | - |
| O95057 | DIRAS1 | 63/114(55.26) | 0.39 | 1.97E-02 | 3.55E-02 | Yes | Up | - |
| Q13895 | BYSL | 64/114(56.14) | 0.33 | 1.44E-05 | 5.58E-05 | Yes | Up | - |
| P27449 | ATP6V0C | 61/114(53.51) | 0.51 | 1.64E-09 | 1.83E-08 | Yes | Up | - |
| P08236 | GUSB | 62/114(54.39) | 0.33 | 1.06E-04 | 3.32E-04 | Yes | Up | - |
| Q9H6E4 | CCDC134 | 59/114(51.75) | 0.22 | 7.51E-03 | 1.50E-02 | Yes | Up | - |
| Q15041 | ARL6IP1 | 61/114(53.51) | 0.52 | 2.24E-08 | 1.82E-07 | Yes | Up | - |
| Q3T8J9 | GON4L | 55/114(48.25) | 0.2 | 4.47E-04 | 1.20E-03 | Yes | Up | - |
| O43916 | CHST1 | 59/114(51.75) | 0.83 | 1.66E-06 | 8.04E-06 | Yes | Up | - |
| Q92934 | BAD | 66/114(57.89) | 0.2 | 4.42E-03 | 9.34E-03 | Yes | Up | - |
| Q96H86 | ZNF764 | 64/114(56.14) | 0.26 | 5.28E-05 | 1.77E-04 | Yes | Up | - |
| Q05639 | EEF1A2 | 59/114(51.75) | 1.58 | 3.06E-10 | 4.26E-09 | Yes | Up | - |
| P26583 | HMGB2 | 62/114(54.39) | 0.42 | 1.56E-05 | 5.98E-05 | Yes | Up | - |
| Q6PRD7 | CEMP1 | 60/114(52.63) | 0.26 | 1.06E-03 | 2.60E-03 | Yes | Up | - |
| O14948 | TFEC | 55/114(48.25) | 0.36 | 4.24E-03 | 8.98E-03 | Yes | Up | - |
| Q92997 | DVL3 | 52/114(45.61) | 0.28 | 3.84E-07 | 2.20E-06 | Yes | Up | - |
| Q14296 | FASTK | 70/114(61.4) | 0.25 | 1.08E-04 | 3.37E-04 | Yes | Up | - |
| M4WDD3 | LOC388588 | 60/114(52.63) | 0.48 | 2.20E-04 | 6.38E-04 | Yes | Up | - |
| Q14002 | CEACAM7 | 61/114(53.51) | 0.79 | 3.27E-04 | 9.08E-04 | Yes | Up | - |
| Q8WVB6 | CHTF18 | 65/114(57.02) | 0.77 | 5.58E-10 | 7.21E-09 | Yes | Up | - |
| P09104 | ENO2 | 63/114(55.26) | 0.52 | 3.10E-05 | 1.10E-04 | Yes | Up | - |
| - | LOC93622 | 57/114(50.0) | 0.19 | 5.26E-03 | 1.09E-02 | Yes | Up | - |
| Q15773 | MLF2 | 64/114(56.14) | 0.33 | 3.66E-07 | 2.11E-06 | Yes | Up | - |
| Q9NY61 | AATF | 60/114(52.63) | 0.31 | 6.42E-07 | 3.47E-06 | Yes | Up | - |
| P60763 | RAC3 | 54/114(47.37) | 0.43 | 1.55E-04 | 4.66E-04 | Yes | Up | - |
| O43854 | EDIL3 | 54/114(47.37) | 0.36 | 1.33E-02 | 2.50E-02 | Yes | Up | - |
| Q5T848 | GPR158 | 58/114(50.88) | 0.54 | 7.44E-03 | 1.49E-02 | Yes | Up | - |
| O95067 | CCNB2 | 57/114(50.0) | 1.56 | 4.64E-14 | 2.60E-12 | Yes | Up | - |
| Q9NYA4 | MTMR4 | 53/114(46.49) | 0.22 | 1.59E-03 | 3.75E-03 | Yes | Up | - |
| O75425 | MOSPD3 | 62/114(54.39) | 0.27 | 1.81E-04 | 5.35E-04 | Yes | Up | - |
| Q86XK7 | VSIG1 | 68/114(59.65) | 0.42 | 8.42E-03 | 1.66E-02 | Yes | Up | - |
| Q12834 | CDC20 | 61/114(53.51) | 1.63 | 8.63E-15 | 6.48E-13 | Yes | Up | - |
| B4DS77 | SHISA9 | 54/114(47.37) | 0.87 | 1.18E-04 | 3.65E-04 | Yes | Up | - |
| P43005 | SLC1A1 | 57/114(50.0) | 0.95 | 7.55E-08 | 5.25E-07 | Yes | Up | - |
| Q9NUP1 | CNO | 65/114(57.02) | 0.22 | 9.03E-05 | 2.87E-04 | Yes | Up | - |
| P40199 | CEACAM6 | 59/114(51.75) | 1.24 | 3.06E-06 | 1.39E-05 | Yes | Up | - |
| Q9H4A6 | GOLPH3 | 54/114(47.37) | 0.2 | 1.08E-03 | 2.64E-03 | Yes | Up | - |
| Q8NEB5 | PPAPDC1B | 60/114(52.63) | 0.53 | 1.14E-06 | 5.77E-06 | Yes | Up | - |
| Q9ULE0 | WWC3 | 60/114(52.63) | 0.18 | 3.01E-03 | 6.61E-03 | Yes | Up | - |
| Q8TAT5 | NEIL3 | 59/114(51.75) | 1.48 | 2.59E-10 | 3.69E-09 | Yes | Up | - |
| Q9H427 | KCNK15 | 60/114(52.63) | 1.47 | 3.01E-13 | 1.18E-11 | Yes | Up | - |
| Q96C10 | DHX58 | 68/114(59.65) | 0.33 | 7.41E-05 | 2.40E-04 | Yes | Up | - |
| O15417 | TNRC18 | 63/114(55.26) | 0.32 | 1.45E-05 | 5.61E-05 | Yes | Up | - |
| Q8NEF3 | CCDC112 | 54/114(47.37) | 0.17 | 7.28E-03 | 1.46E-02 | Yes | Up | - |
| O95347 | SMC2 | 50/114(43.86) | 0.24 | 3.52E-03 | 7.60E-03 | Yes | Up | - |
| P61956 | SUMO2 | 52/114(45.61) | 0.19 | 6.63E-04 | 1.71E-03 | Yes | Up | - |
| Q8IUH3 | RBM45 | 56/114(49.12) | 0.17 | 4.97E-04 | 1.32E-03 | Yes | Up | - |
| Q9UGY1 | NOL12 | 66/114(57.89) | 0.16 | 1.26E-02 | 2.38E-02 | Yes | Up | - |
| Q17RM4 | CCDC142 | 55/114(48.25) | 0.22 | 1.68E-03 | 3.93E-03 | Yes | Up | - |
| P98187 | CYP4F8 | 52/114(45.61) | 0.99 | 1.39E-03 | 3.32E-03 | Yes | Up | - |
| Q6ZNF0 | PAPL | 62/114(54.39) | 0.82 | 3.01E-03 | 6.60E-03 | Yes | Up | - |
| P10746 | UROS | 62/114(54.39) | 0.26 | 1.10E-04 | 3.42E-04 | Yes | Up | - |
| Q9ULZ9 | MMP17 | 69/114(60.53) | 0.49 | 4.43E-04 | 1.19E-03 | Yes | Up | - |
| Q7LBE3 | SLC26A9 | 59/114(51.75) | 0.82 | 1.07E-04 | 3.35E-04 | Yes | Up | - |
| Q14657 | LAGE3 | 67/114(58.77) | 0.89 | 9.60E-14 | 4.67E-12 | Yes | Up | - |
| P12034 | FGF5 | 56/114(49.12) | 1.44 | 6.25E-06 | 2.62E-05 | Yes | Up | - |
| Q92600 | RQCD1 | 54/114(47.37) | 0.17 | 1.01E-02 | 1.96E-02 | Yes | Up | - |
| Q8TCX5 | RHPN1 | 67/114(58.77) | 1.07 | 2.86E-11 | 5.68E-10 | Yes | Up | - |
| O75952 | CABYR | 56/114(49.12) | 0.64 | 8.28E-07 | 4.35E-06 | Yes | Up | - |
| O75145 | PPFIA3 | 60/114(52.63) | 0.5 | 8.93E-05 | 2.84E-04 | Yes | Up | - |
| Q13136 | PPFIA1 | 53/114(46.49) | 0.2 | 7.75E-03 | 1.55E-02 | Yes | Up | - |
| Q6QNY1 | BLOC1S2 | 62/114(54.39) | 0.18 | 1.20E-03 | 2.91E-03 | Yes | Up | - |
| Q99733 | NAP1L4 | 65/114(57.02) | 0.17 | 4.06E-04 | 1.10E-03 | Yes | Up | - |
| Q9BSG5 | RTBDN | 51/114(44.74) | 2.22 | 5.30E-11 | 9.49E-10 | Yes | Up | - |
| Q10472 | GALNT1 | 53/114(46.49) | 0.22 | 1.48E-03 | 3.51E-03 | Yes | Up | - |
| Q9BV40 | VAMP8 | 56/114(49.12) | 0.62 | 5.86E-10 | 7.52E-09 | Yes | Up | - |
| Q9BYC8 | MRPL32 | 63/114(55.26) | 0.1 | 1.08E-02 | 2.08E-02 | Yes | Up | - |
| Q9HB58 | SP110 | 64/114(56.14) | 0.29 | 8.71E-04 | 2.18E-03 | Yes | Up | - |
| Q7Z5U6 | WDR53 | 58/114(50.88) | 0.2 | 9.80E-05 | 3.09E-04 | Yes | Up | - |
| Q5TDE9 | C1ORF57 | 62/114(54.39) | 0.24 | 2.16E-04 | 6.27E-04 | Yes | Up | - |
| O60331 | PIP5K1C | 64/114(56.14) | 0.15 | 2.73E-03 | 6.06E-03 | Yes | Up | - |
| Q9H813 | TMEM206 | 61/114(53.51) | 0.69 | 5.41E-10 | 7.04E-09 | Yes | Up | - |
| P10082 | PYY | 44/114(38.6) | 1.31 | 8.97E-07 | 4.67E-06 | Yes | Up | - |
| Q53H96 | PYCRL | 67/114(58.77) | 0.81 | 2.15E-11 | 4.43E-10 | Yes | Up | - |
| O75204 | TMEM127 | 55/114(48.25) | 0.16 | 6.23E-05 | 2.06E-04 | Yes | Up | - |
| Q9Y5X2 | SNX8 | 60/114(52.63) | 0.22 | 2.87E-03 | 6.33E-03 | Yes | Up | - |
| Q03518 | TAP1 | 66/114(57.89) | 0.51 | 1.28E-05 | 5.02E-05 | Yes | Up | - |
| Q9BVI4 | NOC4L | 69/114(60.53) | 0.4 | 3.49E-09 | 3.54E-08 | Yes | Up | - |
| Q07011 | TNFRSF9 | 57/114(50.0) | 1.43 | 1.01E-10 | 1.65E-09 | Yes | Up | - |
| Q9UQ80 | PA2G4 | 58/114(50.88) | 0.1 | 2.68E-02 | 4.67E-02 | Yes | Up | - |
| A0A0B4J2A2 | PPIAL4C | 63/114(55.26) | 0.45 | 2.09E-07 | 1.29E-06 | Yes | Up | - |
| P28074 | PSMB5 | 59/114(51.75) | 0.32 | 9.53E-06 | 3.84E-05 | Yes | Up | - |
| - | DGCR9 | 58/114(50.88) | 0.7 | 1.53E-05 | 5.89E-05 | Yes | Up | - |
| O60711 | LPXN | 59/114(51.75) | 0.32 | 1.96E-04 | 5.75E-04 | Yes | Up | - |
| Q8WVQ1 | CANT1 | 56/114(49.12) | 0.63 | 4.32E-10 | 5.78E-09 | Yes | Up | - |
| Q2TAA5 | ALG11 | 48/114(42.11) | 0.42 | 1.81E-04 | 5.33E-04 | Yes | Up | - |
| Q8N6G5 | CSGALNACT2 | 59/114(51.75) | 0.13 | 1.76E-02 | 3.22E-02 | Yes | Up | - |
| Q9P296 | GPR77 | 51/114(44.74) | 0.8 | 1.57E-07 | 1.00E-06 | Yes | Up | - |
| Q9NR28 | DIABLO | 67/114(58.77) | 0.36 | 7.66E-09 | 7.04E-08 | Yes | Up | - |
| P04216 | THY1 | 59/114(51.75) | 0.64 | 1.55E-06 | 7.54E-06 | Yes | Up | - |
| Q9Y6C9 | MTCH2 | 52/114(45.61) | 0.23 | 3.67E-03 | 7.89E-03 | Yes | Up | - |
| Q96J92 | WNK4 | 68/114(59.65) | 0.75 | 1.43E-03 | 3.41E-03 | Yes | Up | - |
| Q86SG5 | S100A7A | 44/114(38.6) | 2.21 | 1.53E-08 | 1.30E-07 | Yes | Up | - |
| Q13571 | LAPTM5 | 58/114(50.88) | 0.42 | 7.93E-05 | 2.55E-04 | Yes | Up | - |
| Q6PJP8 | DCLRE1A | 59/114(51.75) | 0.25 | 1.65E-04 | 4.92E-04 | Yes | Up | - |
| P48382 | RFX5 | 58/114(50.88) | 0.41 | 1.21E-07 | 7.89E-07 | Yes | Up | - |
| Q5T3F8 | TMEM63B | 60/114(52.63) | 0.64 | 3.75E-11 | 7.10E-10 | Yes | Up | - |
| Q96RE7 | NACC1 | 64/114(56.14) | 0.79 | 3.47E-14 | 2.06E-12 | Yes | Up | - |
| Q86UD0 | C9ORF140 | 55/114(48.25) | 1.12 | 1.93E-10 | 2.87E-09 | Yes | Up | - |
| Q7Z2E3 | APTX | 63/114(55.26) | 0.2 | 3.66E-05 | 1.28E-04 | Yes | Up | - |
| Q9P107 | GMIP | 67/114(58.77) | 0.61 | 4.42E-13 | 1.64E-11 | Yes | Up | - |
| Q2KJY2 | KIF26B | 62/114(54.39) | 2 | 1.02E-23 | 2.46E-20 | Yes | Up | - |
| Q70JA7 | CHSY3 | 58/114(50.88) | 0.34 | 2.01E-03 | 4.64E-03 | Yes | Up | - |
| P16422 | EPCAM | 55/114(48.25) | 0.72 | 1.63E-04 | 4.87E-04 | Yes | Up | - |
| Q96RD6 | PANX2 | 59/114(51.75) | 0.62 | 7.35E-05 | 2.38E-04 | Yes | Up | - |
| - | WBP11P1 | 58/114(50.88) | 0.19 | 2.57E-02 | 4.50E-02 | Yes | Up | - |
| Q7L590 | MCM10 | 61/114(53.51) | 1.34 | 4.54E-11 | 8.34E-10 | Yes | Up | - |
| Q12884 | FAP | 65/114(57.02) | 1.15 | 2.44E-13 | 9.93E-12 | Yes | Up | - |
| O94766 | B3GAT3 | 67/114(58.77) | 0.39 | 3.60E-07 | 2.08E-06 | Yes | Up | - |
| P00492 | HPRT1 | 63/114(55.26) | 0.48 | 2.22E-06 | 1.04E-05 | Yes | Up | - |
| P15941 | MUC1 | 63/114(55.26) | 1.81 | 4.18E-19 | 1.62E-16 | Yes | Up | - |
| Q9Y248 | GINS2 | 59/114(51.75) | 1.1 | 2.36E-11 | 4.82E-10 | Yes | Up | - |
| Q9H3S5 | PIGM | 54/114(47.37) | 0.47 | 5.05E-09 | 4.89E-08 | Yes | Up | - |
| Q9NZ53 | PODXL2 | 61/114(53.51) | 0.52 | 1.41E-04 | 4.30E-04 | Yes | Up | - |
| Q8IYW4 | ENTHD1 | 61/114(53.51) | 1.39 | 4.33E-08 | 3.22E-07 | Yes | Up | - |
| Q7Z408 | CSMD2 | 63/114(55.26) | 1.23 | 3.10E-12 | 8.47E-11 | Yes | Up | - |
| Q8NHP7 | EXD1 | 52/114(45.61) | 0.67 | 4.40E-03 | 9.31E-03 | Yes | Up | - |
| Q8N4T4 | C9ORF100 | 62/114(54.39) | 0.61 | 1.02E-06 | 5.22E-06 | Yes | Up | - |
| Q9Y5L5 | LENEP | 46/114(40.35) | 0.51 | 2.85E-02 | 4.92E-02 | Yes | Up | - |
| O75676 | RPS6KA4 | 59/114(51.75) | 0.18 | 1.80E-02 | 3.28E-02 | Yes | Up | - |
| P39687 | ANP32A | 60/114(52.63) | 0.17 | 3.61E-04 | 9.92E-04 | Yes | Up | - |
| Q9Y448 | C15ORF23 | 56/114(49.12) | 0.46 | 2.52E-05 | 9.15E-05 | Yes | Up | - |
| Q9P2E5 | CHPF2 | 61/114(53.51) | 0.43 | 1.15E-08 | 1.00E-07 | Yes | Up | - |
| Q96GC5 | MRPL48 | 62/114(54.39) | 0.15 | 1.34E-02 | 2.52E-02 | Yes | Up | - |
| Q9BUR5 | APOO | 60/114(52.63) | 0.48 | 5.85E-08 | 4.17E-07 | Yes | Up | - |
| Q7Z7F0 | KIAA0907 | 50/114(43.86) | 0.17 | 1.96E-02 | 3.53E-02 | Yes | Up | - |
| Q8IWB6 | TEX14 | 56/114(49.12) | 0.9 | 2.74E-08 | 2.15E-07 | Yes | Up | - |
| Q9H013 | ADAM19 | 58/114(50.88) | 0.93 | 4.85E-10 | 6.38E-09 | Yes | Up | - |
| Q92692 | PVRL2 | 61/114(53.51) | 0.77 | 7.27E-13 | 2.48E-11 | Yes | Up | - |
| Q2VIQ3 | KIF4B | 63/114(55.26) | 1.11 | 1.90E-08 | 1.58E-07 | Yes | Up | - |
| Q96EP5 | DAZAP1 | 61/114(53.51) | 0.26 | 2.34E-06 | 1.10E-05 | Yes | Up | - |
| B3KQ72 | MGC29506 | 63/114(55.26) | 0.52 | 3.46E-03 | 7.48E-03 | Yes | Up | - |
| Q96BZ4 | PLD4 | 63/114(55.26) | 0.36 | 2.19E-02 | 3.90E-02 | Yes | Up | - |
| Q6P4Q7 | CNNM4 | 60/114(52.63) | 0.41 | 6.03E-05 | 2.00E-04 | Yes | Up | - |
| Q14142 | TRIM14 | 53/114(46.49) | 0.34 | 7.76E-04 | 1.97E-03 | Yes | Up | - |
| Q5ST30 | VARS2 | 65/114(57.02) | 0.34 | 2.34E-06 | 1.10E-05 | Yes | Up | - |
| P23468 | PTPRD | 55/114(48.25) | 0.33 | 2.61E-02 | 4.55E-02 | Yes | Up | - |
| Q9HCK4 | ROBO2 | 52/114(45.61) | 1.22 | 2.69E-08 | 2.12E-07 | Yes | Up | - |
| Q4U2R6 | MRPL51 | 62/114(54.39) | 0.34 | 3.14E-07 | 1.84E-06 | Yes | Up | - |
| Q99598 | TSNAX | 57/114(50.0) | 0.2 | 3.08E-03 | 6.73E-03 | Yes | Up | - |
| Q9NP31 | SH2D2A | 58/114(50.88) | 0.74 | 5.42E-07 | 2.99E-06 | Yes | Up | - |
| Q9NQR1 | SETD8 | 64/114(56.14) | 0.13 | 5.89E-03 | 1.21E-02 | Yes | Up | - |
| Q6MZZ7 | CAPN13 | 61/114(53.51) | 0.82 | 1.26E-03 | 3.04E-03 | Yes | Up | - |
| Q8IV03 | C9ORF150 | 53/114(46.49) | 0.38 | 5.45E-04 | 1.44E-03 | Yes | Up | - |
| Q9BQ67 | GRWD1 | 62/114(54.39) | 0.22 | 1.62E-05 | 6.16E-05 | Yes | Up | - |
| O60909 | B4GALT2 | 56/114(49.12) | 0.16 | 2.87E-02 | 4.96E-02 | Yes | Up | - |
| Q16576 | RBBP7 | 62/114(54.39) | 0.32 | 1.76E-04 | 5.23E-04 | Yes | Up | - |
| Q96B36 | AKT1S1 | 65/114(57.02) | 0.18 | 4.74E-03 | 9.95E-03 | Yes | Up | - |
| Q08116 | RGS1 | 65/114(57.02) | 0.39 | 2.52E-03 | 5.65E-03 | Yes | Up | - |
| Q9NYJ7 | DLL3 | 57/114(50.0) | 0.81 | 2.13E-03 | 4.87E-03 | Yes | Up | - |
| Q9UJJ9 | GNPTG | 72/114(63.16) | 0.2 | 5.08E-03 | 1.06E-02 | Yes | Up | - |
| P53803 | POLR2K | 62/114(54.39) | 0.32 | 4.80E-05 | 1.63E-04 | Yes | Up | - |
| Q3MJ13 | WDR72 | 56/114(49.12) | 1.3 | 9.23E-08 | 6.25E-07 | Yes | Up | - |
| Q13153 | PAK1 | 53/114(46.49) | 0.34 | 2.43E-04 | 6.96E-04 | Yes | Up | - |
| Q8IXR9 | C12ORF56 | 33/114(28.95) | 0.79 | 1.86E-02 | 3.36E-02 | Yes | Up | - |
| Q969P6 | TOP1MT | 55/114(48.25) | 0.26 | 6.02E-03 | 1.23E-02 | Yes | Up | - |
| Q9H9B4 | SFXN1 | 58/114(50.88) | 0.51 | 7.87E-07 | 4.16E-06 | Yes | Up | - |
| Q08345 | DDR1 | 57/114(50.0) | 0.47 | 4.86E-05 | 1.65E-04 | Yes | Up | - |
| Q9BWG6 | SCNM1 | 62/114(54.39) | 0.46 | 2.09E-09 | 2.25E-08 | Yes | Up | - |
| Q96SB8 | SMC6 | 60/114(52.63) | 0.3 | 5.43E-06 | 2.31E-05 | Yes | Up | - |
| Q9P215 | POGK | 50/114(43.86) | 0.53 | 6.15E-10 | 7.84E-09 | Yes | Up | - |
| Q9Y5B8 | NME7 | 59/114(51.75) | 0.23 | 1.28E-04 | 3.94E-04 | Yes | Up | - |
| Q9BUL5 | PHF23 | 62/114(54.39) | 0.17 | 7.95E-04 | 2.01E-03 | Yes | Up | - |
| P48065 | SLC6A12 | 60/114(52.63) | 0.43 | 3.33E-04 | 9.21E-04 | Yes | Up | - |
| Q95HC2 | HLA-C | 67/114(58.77) | 0.28 | 3.56E-03 | 7.68E-03 | Yes | Up | - |
| P04920 | SLC4A2 | 60/114(52.63) | 0.33 | 2.81E-06 | 1.29E-05 | Yes | Up | - |
| Q16600 | ZNF239 | 51/114(44.74) | 0.42 | 9.47E-04 | 2.35E-03 | Yes | Up | - |
| Q8N5U1 | MS4A15 | 58/114(50.88) | 2.08 | 1.73E-09 | 1.91E-08 | Yes | Up | - |
| Q9UM54 | MYO6 | 64/114(56.14) | 0.4 | 1.70E-04 | 5.07E-04 | Yes | Up | - |
| Q53FV1 | ORMDL2 | 63/114(55.26) | 0.49 | 1.20E-08 | 1.04E-07 | Yes | Up | - |
| Q6ZRH7 | CATSPERG | 69/114(60.53) | 0.35 | 8.65E-03 | 1.71E-02 | Yes | Up | - |
| P09661 | SNRPA1 | 65/114(57.02) | 0.41 | 1.18E-06 | 5.93E-06 | Yes | Up | - |
| Q6RI45 | BRWD3 | 56/114(49.12) | 0.26 | 1.25E-02 | 2.37E-02 | Yes | Up | - |
| Q8TED4 | SLC37A2 | 57/114(50.0) | 0.54 | 2.06E-05 | 7.67E-05 | Yes | Up | - |
| Q96J88 | EPSTI1 | 63/114(55.26) | 0.65 | 1.34E-06 | 6.65E-06 | Yes | Up | - |
| Q9Y5S9 | RBM8A | 60/114(52.63) | 0.32 | 6.02E-07 | 3.28E-06 | Yes | Up | - |
| Q9UI09 | NDUFA12 | 55/114(48.25) | 0.14 | 2.41E-02 | 4.25E-02 | Yes | Up | - |
| Q9BPX3 | NCAPG | 59/114(51.75) | 1.35 | 4.83E-12 | 1.24E-10 | Yes | Up | - |
| Q9Y3C8 | UFC1 | 60/114(52.63) | 0.51 | 4.03E-12 | 1.05E-10 | Yes | Up | - |
| Q8IWZ8 | SF4 | 70/114(61.4) | 0.23 | 8.30E-06 | 3.40E-05 | Yes | Up | - |
| Q96GP6 | SCARF2 | 62/114(54.39) | 0.44 | 8.27E-05 | 2.65E-04 | Yes | Up | - |
| Q9H2G9 | BLZF1 | 50/114(43.86) | 0.24 | 1.89E-04 | 5.56E-04 | Yes | Up | - |
| P45877 | PPIC | 65/114(57.02) | 0.24 | 2.80E-03 | 6.19E-03 | Yes | Up | - |
| Q96DN6 | MBD6 | 60/114(52.63) | 0.2 | 1.20E-05 | 4.73E-05 | Yes | Up | - |
| Q96NB2 | SFXN2 | 54/114(47.37) | 0.69 | 8.58E-09 | 7.76E-08 | Yes | Up | - |
| O75072 | FKTN | 57/114(50.0) | 0.16 | 1.62E-02 | 2.98E-02 | Yes | Up | - |
| Q8WVC0 | LEO1 | 55/114(48.25) | 0.48 | 1.93E-09 | 2.10E-08 | Yes | Up | - |
| Q13733 | ATP1A4 | 56/114(49.12) | 0.73 | 1.14E-04 | 3.53E-04 | Yes | Up | - |
| Q6ZXV5 | TMTC3 | 57/114(50.0) | 0.23 | 6.20E-04 | 1.61E-03 | Yes | Up | - |
| Q99615 | DNAJC7 | 59/114(51.75) | 0.15 | 1.99E-03 | 4.58E-03 | Yes | Up | - |
| O14497 | ARID1A | 59/114(51.75) | 0.19 | 2.71E-04 | 7.66E-04 | Yes | Up | - |
| Q9NZL4 | HSPBP1 | 68/114(59.65) | 0.38 | 3.07E-07 | 1.80E-06 | Yes | Up | - |
| Q96F86 | EDC3 | 54/114(47.37) | 0.11 | 5.57E-03 | 1.15E-02 | Yes | Up | - |
| Q9Y5K6 | CD2AP | 51/114(44.74) | 0.22 | 4.27E-03 | 9.05E-03 | Yes | Up | - |
| Q8N5G2 | TMEM57 | 60/114(52.63) | 0.33 | 4.37E-07 | 2.46E-06 | Yes | Up | - |
| Q6AWA7 | PPIL5 | 57/114(50.0) | 0.24 | 2.08E-03 | 4.76E-03 | Yes | Up | - |
| O00515 | LAD1 | 59/114(51.75) | 0.54 | 4.60E-03 | 9.69E-03 | Yes | Up | - |
| Q7L591 | DOK3 | 62/114(54.39) | 0.41 | 4.56E-05 | 1.55E-04 | Yes | Up | - |
| P55771 | PAX9 | 63/114(55.26) | 0.81 | 1.02E-05 | 4.10E-05 | Yes | Up | - |
| Q969L4 | LSM10 | 61/114(53.51) | 0.23 | 1.37E-03 | 3.28E-03 | Yes | Up | - |
| P62308 | SNRPG | 67/114(58.77) | 0.42 | 1.56E-07 | 9.95E-07 | Yes | Up | - |
| Q9BXN2 | CLEC7A | 60/114(52.63) | 0.7 | 3.82E-08 | 2.88E-07 | Yes | Up | - |
| Q14112 | NID2 | 55/114(48.25) | 0.36 | 6.56E-03 | 1.33E-02 | Yes | Up | - |
| Q96JY6 | PDLIM2 | 62/114(54.39) | 0.21 | 1.44E-02 | 2.68E-02 | Yes | Up | - |
| Q9UKF6 | CPSF3 | 60/114(52.63) | 0.22 | 2.31E-04 | 6.65E-04 | Yes | Up | - |
| P49863 | GZMK | 57/114(50.0) | 0.62 | 2.82E-04 | 7.92E-04 | Yes | Up | - |
| Q96CN5 | LRRC45 | 63/114(55.26) | 0.72 | 2.06E-12 | 5.97E-11 | Yes | Up | - |
| Q9NPH2 | ISYNA1 | 64/114(56.14) | 0.66 | 3.84E-10 | 5.22E-09 | Yes | Up | - |
| E3W9A1 | C19ORF51 | 65/114(57.02) | 1.23 | 3.31E-09 | 3.38E-08 | Yes | Up | - |
| Q7Z5L0 | VMO1 | 64/114(56.14) | 0.5 | 6.22E-04 | 1.62E-03 | Yes | Up | - |
| Q32ZL2 | LPPR5 | 59/114(51.75) | 1.34 | 3.43E-06 | 1.54E-05 | Yes | Up | - |
| Q9BY11 | PACSIN1 | 60/114(52.63) | 1.29 | 7.06E-13 | 2.43E-11 | Yes | Up | - |
| Q7Z2H8 | SLC36A1 | 55/114(48.25) | 0.23 | 3.52E-03 | 7.60E-03 | Yes | Up | - |
| Q9H4A5 | GOLPH3L | 53/114(46.49) | 0.32 | 1.73E-05 | 6.56E-05 | Yes | Up | - |
| Q6ULP2 | AFTPH | 61/114(53.51) | 0.26 | 1.69E-04 | 5.02E-04 | Yes | Up | - |
| P04843 | RPN1 | 61/114(53.51) | 0.37 | 2.22E-08 | 1.80E-07 | Yes | Up | - |
| Q7Z7E8 | UBE2Q1 | 58/114(50.88) | 0.3 | 1.85E-07 | 1.15E-06 | Yes | Up | - |
| Q96H96 | COQ2 | 61/114(53.51) | 0.25 | 2.77E-03 | 6.14E-03 | Yes | Up | - |
| Q9NWW5 | CLN6 | 59/114(51.75) | 0.38 | 5.50E-08 | 3.96E-07 | Yes | Up | - |
| - | LOC723972 | 55/114(48.25) | 0.27 | 4.98E-03 | 1.04E-02 | Yes | Up | - |
| Q9BQ50 | TREX2 | 63/114(55.26) | 0.55 | 8.66E-04 | 2.17E-03 | Yes | Up | - |
| Q08708 | CD300C | 62/114(54.39) | 0.4 | 2.57E-04 | 7.32E-04 | Yes | Up | - |
| Q96NS5 | ASB16 | 57/114(50.0) | 0.42 | 2.25E-03 | 5.12E-03 | Yes | Up | - |
| Q9NYD6 | HOXC10 | 66/114(57.89) | 1.01 | 1.07E-06 | 5.44E-06 | Yes | Up | - |
| Q9Y253 | POLH | 55/114(48.25) | 0.18 | 4.40E-04 | 1.18E-03 | Yes | Up | - |
| P10243 | MYBL1 | 58/114(50.88) | 0.75 | 1.58E-06 | 7.69E-06 | Yes | Up | - |
| O95785 | WIZ | 57/114(50.0) | 0.19 | 5.38E-04 | 1.42E-03 | Yes | Up | - |
| P54219 | SLC18A1 | 43/114(37.72) | 0.88 | 2.68E-03 | 5.95E-03 | Yes | Up | - |
| Q9NXH3 | PPP1R14D | 57/114(50.0) | 0.45 | 2.09E-02 | 3.74E-02 | Yes | Up | - |
| O94927 | HAUS5 | 65/114(57.02) | 0.16 | 2.51E-02 | 4.40E-02 | Yes | Up | - |
| Q4AC94 | C2CD3 | 55/114(48.25) | 0.14 | 2.65E-02 | 4.62E-02 | Yes | Up | - |
| P83110 | HTRA3 | 63/114(55.26) | 0.41 | 1.15E-02 | 2.19E-02 | Yes | Up | - |
| - | NCRNA00095 | 52/114(45.61) | 0.35 | 2.03E-03 | 4.67E-03 | Yes | Up | - |
| Q96MH7 | C5ORF34 | 62/114(54.39) | 0.4 | 1.71E-04 | 5.08E-04 | Yes | Up | - |
| P49279 | SLC11A1 | 66/114(57.89) | 0.79 | 1.09E-07 | 7.23E-07 | Yes | Up | - |
| P62937 | PPIA | 60/114(52.63) | 0.3 | 8.77E-06 | 3.57E-05 | Yes | Up | - |
| Q9P0L9 | PKD2L1 | 62/114(54.39) | 0.69 | 6.31E-04 | 1.64E-03 | Yes | Up | - |
| Q8NEP7 | KLHDC9 | 64/114(56.14) | 0.78 | 1.29E-08 | 1.11E-07 | Yes | Up | - |
| Q9P289 | MST4 | 65/114(57.02) | 0.35 | 8.48E-03 | 1.68E-02 | Yes | Up | - |
| Q12926 | ELAVL2 | 61/114(53.51) | 1.29 | 4.77E-12 | 1.22E-10 | Yes | Up | - |
| Q14469 | HES1 | 54/114(47.37) | 0.23 | 1.48E-02 | 2.75E-02 | Yes | Up | - |
| Q6T311 | ARL9 | 61/114(53.51) | 0.37 | 1.55E-02 | 2.86E-02 | Yes | Up | - |
| Q9Y3X0 | CCDC9 | 71/114(62.28) | 0.35 | 5.25E-06 | 2.24E-05 | Yes | Up | - |
| Q96A33 | CCDC47 | 49/114(42.98) | 0.21 | 5.77E-04 | 1.51E-03 | Yes | Up | - |
| P52888 | THOP1 | 57/114(50.0) | 0.32 | 8.10E-05 | 2.60E-04 | Yes | Up | - |
| Q9H3K6 | BOLA2 | 65/114(57.02) | 0.56 | 8.76E-11 | 1.45E-09 | Yes | Up | - |
| O14562 | UBFD1 | 56/114(49.12) | 0.27 | 3.31E-04 | 9.17E-04 | Yes | Up | - |
| Q9BPW5 | RASL11B | 58/114(50.88) | 1.13 | 1.19E-11 | 2.65E-10 | Yes | Up | - |
| Q5SQH8 | C6ORF136 | 57/114(50.0) | 0.15 | 1.93E-02 | 3.49E-02 | Yes | Up | - |
| Q9H0A8 | COMMD4 | 63/114(55.26) | 0.39 | 9.89E-09 | 8.80E-08 | Yes | Up | - |
| P23919 | DTYMK | 64/114(56.14) | 0.5 | 2.55E-09 | 2.68E-08 | Yes | Up | - |
| O15381 | NVL | 60/114(52.63) | 0.68 | 1.65E-10 | 2.51E-09 | Yes | Up | - |
| P43487 | RANBP1 | 59/114(51.75) | 0.29 | 3.60E-05 | 1.26E-04 | Yes | Up | - |
| Q96EP1 | CHFR | 69/114(60.53) | 0.25 | 2.09E-05 | 7.75E-05 | Yes | Up | - |
| Q6UW68 | TMEM205 | 60/114(52.63) | 0.35 | 3.97E-05 | 1.38E-04 | Yes | Up | - |
| Q9Y5Q8 | GTF3C5 | 62/114(54.39) | 0.19 | 2.71E-04 | 7.67E-04 | Yes | Up | - |
| - | LOC728606 | 61/114(53.51) | 1.03 | 8.22E-05 | 2.64E-04 | Yes | Up | - |
| Q9NY93 | DDX56 | 66/114(57.89) | 0.27 | 1.96E-06 | 9.32E-06 | Yes | Up | - |
| Q8WTT0 | CLEC4C | 41/114(35.96) | 0.92 | 1.23E-03 | 2.97E-03 | Yes | Up | - |
| Q4G0I0 | C16ORF91 | 64/114(56.14) | 0.47 | 1.98E-09 | 2.15E-08 | Yes | Up | - |
| Q86YJ7 | ANKRD13B | 59/114(51.75) | 0.25 | 9.69E-03 | 1.88E-02 | Yes | Up | - |
| Q9H9L4 | C12ORF41 | 56/114(49.12) | 0.12 | 2.42E-02 | 4.26E-02 | Yes | Up | - |
| - | LOC90784 | 55/114(48.25) | 0.22 | 9.50E-04 | 2.36E-03 | Yes | Up | - |
| Q969X5 | ERGIC1 | 59/114(51.75) | 0.32 | 4.71E-04 | 1.26E-03 | Yes | Up | - |
| Q9UDW3 | ZMAT5 | 60/114(52.63) | 0.25 | 9.33E-05 | 2.96E-04 | Yes | Up | - |
| Q49A26 | GLYR1 | 65/114(57.02) | 0.21 | 3.67E-04 | 1.01E-03 | Yes | Up | - |
| Q12824 | SMARCB1 | 55/114(48.25) | 0.32 | 3.62E-08 | 2.74E-07 | Yes | Up | - |
| Q66K74 | MAP1S | 70/114(61.4) | 0.3 | 4.03E-06 | 1.76E-05 | Yes | Up | - |
| P49721 | PSMB2 | 65/114(57.02) | 0.19 | 3.05E-03 | 6.67E-03 | Yes | Up | - |
| Q9NXG6 | P4HTM | 63/114(55.26) | 0.47 | 2.72E-07 | 1.62E-06 | Yes | Up | - |
| Q9BX79 | STRA6 | 63/114(55.26) | 0.64 | 8.95E-04 | 2.24E-03 | Yes | Up | - |
| Q9UF47 | DNAJC5B | 59/114(51.75) | 1.09 | 1.27E-07 | 8.28E-07 | Yes | Up | - |
| - | LOC387646 | 51/114(44.74) | 0.3 | 2.56E-02 | 4.48E-02 | Yes | Up | - |
| Q99259 | GAD1 | 61/114(53.51) | 1.13 | 4.08E-08 | 3.05E-07 | Yes | Up | - |
| P0C0S5 | H2AFZ | 61/114(53.51) | 0.65 | 1.45E-09 | 1.66E-08 | Yes | Up | - |
| Q8IWQ3 | BRSK2 | 61/114(53.51) | 1.48 | 1.13E-11 | 2.54E-10 | Yes | Up | - |
| - | FLJ45445 | 49/114(42.98) | 0.48 | 4.25E-04 | 1.15E-03 | Yes | Up | - |
| Q9P2I0 | CPSF2 | 59/114(51.75) | 0.19 | 3.44E-03 | 7.45E-03 | Yes | Up | - |
| Q9H4Q4 | PRDM12 | 60/114(52.63) | 0.45 | 2.34E-02 | 4.14E-02 | Yes | Up | - |
| P48556 | PSMD8 | 58/114(50.88) | 0.25 | 2.15E-04 | 6.23E-04 | Yes | Up | - |
| O95427 | PIGN | 62/114(54.39) | 0.15 | 1.93E-02 | 3.48E-02 | Yes | Up | - |
| Q92918 | MAP4K1 | 65/114(57.02) | 0.49 | 1.80E-04 | 5.31E-04 | Yes | Up | - |
| P28221 | HTR1D | 57/114(50.0) | 1.89 | 7.73E-18 | 1.92E-15 | Yes | Up | - |
| Q7Z769 | SLC35E3 | 48/114(42.11) | 0.29 | 8.15E-05 | 2.62E-04 | Yes | Up | - |
| Q96N66 | MBOAT7 | 66/114(57.89) | 0.74 | 2.06E-11 | 4.28E-10 | Yes | Up | - |
| P29274 | ADORA2A | 59/114(51.75) | 1.25 | 2.16E-17 | 4.95E-15 | Yes | Up | - |
| Q13145 | BAMBI | 53/114(46.49) | 0.8 | 3.91E-07 | 2.24E-06 | Yes | Up | - |
| Q16775 | HAGH | 61/114(53.51) | 0.34 | 1.03E-04 | 3.22E-04 | Yes | Up | - |
| Q12980 | NPRL3 | 66/114(57.89) | 0.18 | 3.10E-04 | 8.64E-04 | Yes | Up | - |
| O00237 | RNF103 | 62/114(54.39) | 0.23 | 1.01E-04 | 3.18E-04 | Yes | Up | - |
| O95208 | EPN2 | 61/114(53.51) | 0.14 | 1.77E-02 | 3.22E-02 | Yes | Up | - |
| Q9Y2Z4 | YARS2 | 62/114(54.39) | 0.22 | 3.75E-05 | 1.31E-04 | Yes | Up | - |
| Q8N9N5 | BANP | 65/114(57.02) | 0.18 | 2.26E-03 | 5.12E-03 | Yes | Up | - |
| Q9NSE7 | ABCC13 | 48/114(42.11) | 1.07 | 2.02E-03 | 4.64E-03 | Yes | Up | - |
| Q5VWP2 | FAM46C | 71/114(62.28) | 0.29 | 1.55E-02 | 2.87E-02 | Yes | Up | - |
| Q5GFL6 | VWA2 | 64/114(56.14) | 0.76 | 3.07E-05 | 1.09E-04 | Yes | Up | - |
| Q5VY80 | RAET1L | 54/114(47.37) | 0.9 | 6.06E-04 | 1.58E-03 | Yes | Up | - |
| Q9H993 | C6ORF211 | 59/114(51.75) | 0.52 | 2.04E-05 | 7.58E-05 | Yes | Up | - |
| Q9BRT9 | GINS4 | 60/114(52.63) | 0.91 | 4.13E-11 | 7.72E-10 | Yes | Up | - |
| Q9GZL7 | WDR12 | 54/114(47.37) | 0.17 | 1.17E-02 | 2.23E-02 | Yes | Up | - |
| P81605 | DCD | 46/114(40.35) | 0.88 | 2.40E-02 | 4.23E-02 | Yes | Up | - |
| O43255 | SIAH2 | 63/114(55.26) | 0.86 | 5.12E-13 | 1.84E-11 | Yes | Up | - |
| Q8WY54 | PPM1E | 58/114(50.88) | 0.71 | 1.35E-05 | 5.26E-05 | Yes | Up | - |
| Q53EV4 | LRRC23 | 61/114(53.51) | 0.32 | 3.68E-06 | 1.63E-05 | Yes | Up | - |
| Q96C00 | ZBTB9 | 54/114(47.37) | 0.33 | 3.18E-06 | 1.44E-05 | Yes | Up | - |
| Q99741 | CDC6 | 63/114(55.26) | 1.35 | 9.98E-14 | 4.84E-12 | Yes | Up | - |
| O15031 | PLXNB2 | 62/114(54.39) | 0.22 | 1.83E-03 | 4.24E-03 | Yes | Up | - |
| O15258 | RER1 | 57/114(50.0) | 0.2 | 1.62E-04 | 4.86E-04 | Yes | Up | - |
| Q8TAE8 | GADD45GIP1 | 66/114(57.89) | 0.47 | 1.94E-06 | 9.23E-06 | Yes | Up | - |
| Q86TP1 | PRUNE | 54/114(47.37) | 0.44 | 3.56E-09 | 3.61E-08 | Yes | Up | - |
| O15145 | ARPC3 | 65/114(57.02) | 0.41 | 2.23E-08 | 1.81E-07 | Yes | Up | - |
| P20813 | CYP2B6 | 51/114(44.74) | 1.82 | 2.08E-10 | 3.05E-09 | Yes | Up | - |
| Q96EG1 | ARSG | 53/114(46.49) | 0.34 | 6.71E-03 | 1.36E-02 | Yes | Up | - |
| P28325 | CST5 | 47/114(41.23) | 2.48 | 4.24E-10 | 5.68E-09 | Yes | Up | - |
| O43463 | SUV39H1 | 65/114(57.02) | 0.26 | 9.28E-05 | 2.94E-04 | Yes | Up | - |
| P30679 | GNA15 | 60/114(52.63) | 0.25 | 1.17E-02 | 2.22E-02 | Yes | Up | - |
| Q9NTZ6 | RBM12 | 59/114(51.75) | 0.11 | 9.67E-03 | 1.88E-02 | Yes | Up | - |
| O15427 | SLC16A3 | 63/114(55.26) | 1.18 | 1.33E-13 | 6.07E-12 | Yes | Up | - |
| P43897 | TSFM | 61/114(53.51) | 0.29 | 1.00E-06 | 5.15E-06 | Yes | Up | - |
| Q8WZ82 | OVCA2 | 62/114(54.39) | 0.27 | 3.45E-06 | 1.54E-05 | Yes | Up | - |
| Q9UKK9 | NUDT5 | 61/114(53.51) | 0.36 | 5.61E-06 | 2.38E-05 | Yes | Up | - |
| H0YL14 | C9ORF69 | 65/114(57.02) | 0.23 | 3.15E-04 | 8.78E-04 | Yes | Up | - |
| O60783 | MRPS14 | 53/114(46.49) | 0.21 | 1.83E-04 | 5.40E-04 | Yes | Up | - |
| Q9HB14 | KCNK13 | 63/114(55.26) | 0.41 | 2.49E-03 | 5.59E-03 | Yes | Up | - |
| P80294 | MT1H | 43/114(37.72) | 0.8 | 3.65E-03 | 7.85E-03 | Yes | Up | - |
| P08727 | KRT19 | 58/114(50.88) | 0.9 | 5.11E-06 | 2.19E-05 | Yes | Up | - |
| Q6P1M0 | SLC27A4 | 57/114(50.0) | 0.44 | 2.13E-07 | 1.30E-06 | Yes | Up | - |
| Q8NEQ6 | C1ORF64 | 52/114(45.61) | 0.81 | 2.34E-03 | 5.28E-03 | Yes | Up | - |
| Q9ULG3 | KIAA1257 | 60/114(52.63) | 0.94 | 1.00E-08 | 8.90E-08 | Yes | Up | - |
| Q969F9 | HPS3 | 60/114(52.63) | 0.31 | 9.03E-07 | 4.70E-06 | Yes | Up | - |
| P14174 | MIF | 61/114(53.51) | 0.83 | 3.05E-10 | 4.25E-09 | Yes | Up | - |
| Q9BYC9 | MRPL20 | 63/114(55.26) | 0.22 | 1.94E-04 | 5.68E-04 | Yes | Up | - |
| P78395 | PRAME | 58/114(50.88) | 2.4 | 3.33E-18 | 1.02E-15 | Yes | Up | - |
| - | LOC550643 | 61/114(53.51) | 0.25 | 1.23E-03 | 2.98E-03 | Yes | Up | - |
| Q8TD07 | RAET1E | 55/114(48.25) | 0.34 | 2.57E-02 | 4.50E-02 | Yes | Up | - |
| Q86WD7 | SERPINA9 | 51/114(44.74) | 1.37 | 4.82E-07 | 2.70E-06 | Yes | Up | - |
| P12109 | COL6A1 | 60/114(52.63) | 0.32 | 1.14E-02 | 2.19E-02 | Yes | Up | - |
| - | C19ORF23 | 55/114(48.25) | 0.38 | 2.79E-05 | 1.00E-04 | Yes | Up | - |
| P06702 | S100A9 | 59/114(51.75) | 0.47 | 2.46E-02 | 4.32E-02 | Yes | Up | - |
| Q9UIG0 | BAZ1B | 54/114(47.37) | 0.17 | 3.09E-03 | 6.75E-03 | Yes | Up | - |
| D3DPV3 | ZNF643 | 56/114(49.12) | 0.44 | 9.23E-05 | 2.93E-04 | Yes | Up | - |
| Q9NP92 | MRPS30 | 60/114(52.63) | 0.37 | 2.04E-03 | 4.68E-03 | Yes | Up | - |
| Q5SR53 | C1ORF200 | 53/114(46.49) | 0.81 | 4.74E-05 | 1.61E-04 | Yes | Up | - |
| P51610 | HCFC1 | 59/114(51.75) | 0.19 | 1.58E-04 | 4.75E-04 | Yes | Up | - |
| Q01201 | RELB | 75/114(65.79) | 0.41 | 8.72E-07 | 4.55E-06 | Yes | Up | - |
| Q16877 | PFKFB4 | 58/114(50.88) | 0.72 | 1.19E-09 | 1.39E-08 | Yes | Up | - |
| Q9NXW9 | ALKBH4 | 64/114(56.14) | 0.21 | 5.57E-05 | 1.86E-04 | Yes | Up | - |
| Q8N1B4 | VPS52 | 63/114(55.26) | 0.15 | 2.53E-03 | 5.65E-03 | Yes | Up | - |
| Q8NFZ8 | CADM4 | 63/114(55.26) | 0.4 | 5.66E-04 | 1.49E-03 | Yes | Up | - |
| Q9NQ36 | SCUBE2 | 55/114(48.25) | 0.58 | 3.78E-03 | 8.09E-03 | Yes | Up | - |
| A0PJW6 | TMEM223 | 66/114(57.89) | 0.3 | 5.77E-06 | 2.44E-05 | Yes | Up | - |
| Q9BTT0 | ANP32E | 54/114(47.37) | 0.25 | 1.50E-02 | 2.78E-02 | Yes | Up | - |
| Q14246 | EMR1 | 58/114(50.88) | 0.32 | 2.20E-02 | 3.92E-02 | Yes | Up | - |
| P35269 | GTF2F1 | 61/114(53.51) | 0.14 | 2.89E-03 | 6.37E-03 | Yes | Up | - |
| Q8TC29 | ENKUR | 49/114(42.98) | 0.68 | 3.98E-05 | 1.38E-04 | Yes | Up | - |
| O75596 | CLEC3A | 57/114(50.0) | 2.44 | 4.92E-11 | 8.90E-10 | Yes | Up | - |
| Q99661 | KIF2C | 61/114(53.51) | 1.61 | 2.47E-14 | 1.57E-12 | Yes | Up | - |
| Q6P444 | FAM54A | 61/114(53.51) | 1.41 | 5.37E-14 | 2.91E-12 | Yes | Up | - |
| Q86X10 | RALGAPB | 55/114(48.25) | 0.14 | 2.26E-02 | 4.01E-02 | Yes | Up | - |
| Q9UNY4 | TTF2 | 51/114(44.74) | 0.22 | 6.60E-03 | 1.34E-02 | Yes | Up | - |
| Q6UW63 | KDELC1 | 55/114(48.25) | 0.29 | 3.78E-04 | 1.03E-03 | Yes | Up | - |
| P51788 | CLCN2 | 57/114(50.0) | 0.73 | 6.64E-10 | 8.37E-09 | Yes | Up | - |
| P09001 | MRPL3 | 56/114(49.12) | 0.31 | 1.34E-05 | 5.23E-05 | Yes | Up | - |
| P30305 | CDC25B | 62/114(54.39) | 0.32 | 7.90E-04 | 2.00E-03 | Yes | Up | - |
| Q9BQ15 | OBFC2B | 65/114(57.02) | 0.43 | 6.86E-11 | 1.18E-09 | Yes | Up | - |
| Q9H9A7 | RMI1 | 58/114(50.88) | 0.61 | 2.28E-08 | 1.85E-07 | Yes | Up | - |
| Q96DN0 | ERP27 | 57/114(50.0) | 0.92 | 7.59E-08 | 5.27E-07 | Yes | Up | - |
| Q9BUN5 | CCDC28B | 62/114(54.39) | 0.44 | 7.25E-05 | 2.36E-04 | Yes | Up | - |
| Q6ZUI0 | TPRG1 | 64/114(56.14) | 0.58 | 2.46E-03 | 5.52E-03 | Yes | Up | - |
| P05362 | ICAM1 | 68/114(59.65) | 0.39 | 4.77E-04 | 1.27E-03 | Yes | Up | - |
| Q69YU5 | C12ORF73 | 53/114(46.49) | 0.21 | 3.66E-03 | 7.88E-03 | Yes | Up | - |
| Q96BH1 | RNF25 | 71/114(62.28) | 0.18 | 2.11E-04 | 6.14E-04 | Yes | Up | - |
| P16112 | ACAN | 58/114(50.88) | 1.51 | 1.71E-13 | 7.49E-12 | Yes | Up | - |
| P53004 | BLVRA | 56/114(49.12) | 0.28 | 2.03E-03 | 4.67E-03 | Yes | Up | - |
| O75844 | ZMPSTE24 | 54/114(47.37) | 0.29 | 1.05E-04 | 3.28E-04 | Yes | Up | - |
| Q9H040 | C1ORF124 | 52/114(45.61) | 0.23 | 3.26E-04 | 9.04E-04 | Yes | Up | - |
| P62873 | GNB1 | 51/114(44.74) | 0.12 | 2.32E-02 | 4.11E-02 | Yes | Up | - |
| O60888 | CUTA | 60/114(52.63) | 0.27 | 2.52E-05 | 9.15E-05 | Yes | Up | - |
| P46779 | RPL28 | 67/114(58.77) | 0.21 | 1.22E-02 | 2.31E-02 | Yes | Up | - |
| O75054 | IGSF3 | 52/114(45.61) | 0.64 | 4.12E-07 | 2.34E-06 | Yes | Up | - |
| Q8TEX9 | IPO4 | 56/114(49.12) | 0.34 | 5.46E-05 | 1.83E-04 | Yes | Up | - |
| Q3ZCT1 | ZNF260 | 54/114(47.37) | 0.24 | 8.10E-04 | 2.05E-03 | Yes | Up | - |
| O15511 | ARPC5 | 61/114(53.51) | 0.3 | 7.63E-06 | 3.14E-05 | Yes | Up | - |
| Q6UW78 | C11ORF83 | 67/114(58.77) | 0.51 | 2.72E-07 | 1.62E-06 | Yes | Up | - |
| P06753 | TPM3 | 60/114(52.63) | 0.67 | 3.30E-10 | 4.55E-09 | Yes | Up | - |
| Q9UKJ1 | PILRA | 64/114(56.14) | 0.6 | 2.78E-08 | 2.18E-07 | Yes | Up | - |
| O14579 | COPE | 66/114(57.89) | 0.52 | 5.78E-10 | 7.44E-09 | Yes | Up | - |
| Q9BR76 | CORO1B | 67/114(58.77) | 0.48 | 1.09E-08 | 9.57E-08 | Yes | Up | - |
| Q9GZW8 | MS4A7 | 51/114(44.74) | 0.38 | 4.51E-03 | 9.52E-03 | Yes | Up | - |
| O15444 | CCL25 | 53/114(46.49) | 0.84 | 1.09E-03 | 2.67E-03 | Yes | Up | - |
| Q09161 | NCBP1 | 55/114(48.25) | 0.18 | 4.45E-03 | 9.40E-03 | Yes | Up | - |
| - | OR7E91P | 57/114(50.0) | 0.65 | 2.22E-03 | 5.04E-03 | Yes | Up | - |
| Q9BYC5 | FUT8 | 56/114(49.12) | 0.57 | 1.95E-07 | 1.21E-06 | Yes | Up | - |
| O94972 | TRIM37 | 56/114(49.12) | 0.37 | 1.78E-05 | 6.72E-05 | Yes | Up | - |
| Q8WYP5 | AHCTF1 | 57/114(50.0) | 0.18 | 9.44E-03 | 1.84E-02 | Yes | Up | - |
| O15320 | CTAGE5 | 54/114(47.37) | 0.18 | 1.33E-02 | 2.50E-02 | Yes | Up | - |
| Q8N3J3 | C17ORF53 | 64/114(56.14) | 0.27 | 1.01E-02 | 1.96E-02 | Yes | Up | - |
| Q03252 | LMNB2 | 61/114(53.51) | 0.63 | 1.19E-09 | 1.39E-08 | Yes | Up | - |
| Q9H816 | DCLRE1B | 56/114(49.12) | 0.19 | 4.34E-04 | 1.17E-03 | Yes | Up | - |
| Q9Y493 | ZAN | 45/114(39.47) | 1.46 | 1.56E-06 | 7.62E-06 | Yes | Up | - |
| Q3B825 | C6ORF154 | 60/114(52.63) | 1.12 | 1.94E-10 | 2.88E-09 | Yes | Up | - |
| Q06945 | SOX4 | 56/114(49.12) | 0.42 | 1.55E-04 | 4.66E-04 | Yes | Up | - |
| P17039 | ZNF30 | 51/114(44.74) | 0.17 | 1.30E-02 | 2.46E-02 | Yes | Up | - |
| Q9Y237 | PIN4 | 66/114(57.89) | 0.2 | 4.77E-04 | 1.27E-03 | Yes | Up | - |
| O00231 | PSMD11 | 54/114(47.37) | 0.36 | 9.91E-06 | 3.99E-05 | Yes | Up | - |
| Q9NQW6 | ANLN | 57/114(50.0) | 1.6 | 1.99E-13 | 8.39E-12 | Yes | Up | - |
| P17038 | ZNF43 | 54/114(47.37) | 0.38 | 1.28E-04 | 3.94E-04 | Yes | Up | - |
| Q9H840 | GEMIN7 | 62/114(54.39) | 0.36 | 2.24E-08 | 1.82E-07 | Yes | Up | - |
| Q8WWI1 | LMO7 | 56/114(49.12) | 0.43 | 9.01E-04 | 2.25E-03 | Yes | Up | - |
| Q9NS68 | TNFRSF19 | 60/114(52.63) | 0.28 | 2.63E-02 | 4.59E-02 | Yes | Up | - |
| Q9H173 | SIL1 | 56/114(49.12) | 0.34 | 7.90E-05 | 2.55E-04 | Yes | Up | - |
| Q8IVA1 | PCP2 | 60/114(52.63) | 0.91 | 3.38E-06 | 1.52E-05 | Yes | Up | - |
| Q15428 | SF3A2 | 64/114(56.14) | 0.17 | 4.93E-03 | 1.03E-02 | Yes | Up | - |
| Q6P1M3 | LLGL2 | 55/114(48.25) | 1.01 | 8.59E-13 | 2.82E-11 | Yes | Up | - |
| Q86UK7 | ZNF598 | 69/114(60.53) | 0.35 | 1.47E-06 | 7.23E-06 | Yes | Up | - |
| Q8IZD6 | SLC22A15 | 57/114(50.0) | 0.75 | 2.61E-09 | 2.73E-08 | Yes | Up | - |
| Q9UJ42 | GPR160 | 60/114(52.63) | 0.8 | 4.63E-09 | 4.54E-08 | Yes | Up | - |
| Q9ULM2 | ZNF490 | 58/114(50.88) | 0.36 | 3.46E-07 | 2.01E-06 | Yes | Up | - |
| Q8N712 | C17ORF95 | 58/114(50.88) | 0.13 | 1.89E-02 | 3.42E-02 | Yes | Up | - |
| Q9UHX1 | PUF60 | 65/114(57.02) | 0.34 | 1.54E-05 | 5.91E-05 | Yes | Up | - |
| Q7Z7H3 | C2ORF62 | 58/114(50.88) | 0.82 | 4.15E-04 | 1.12E-03 | Yes | Up | - |
| Q5TA45 | CPSF3L | 62/114(54.39) | 0.18 | 1.57E-03 | 3.70E-03 | Yes | Up | - |
| Q8IZK6 | MCOLN2 | 66/114(57.89) | 0.64 | 6.53E-07 | 3.52E-06 | Yes | Up | - |
| Q86XJ0 | CALHM3 | 55/114(48.25) | 0.56 | 2.89E-02 | 4.99E-02 | Yes | Up | - |
| Q9Y543 | HES2 | 52/114(45.61) | 0.83 | 6.49E-06 | 2.71E-05 | Yes | Up | - |
| Q9Y6N5 | SQRDL | 68/114(59.65) | 0.28 | 2.74E-04 | 7.74E-04 | Yes | Up | - |
| Q9Y316 | MEMO1 | 54/114(47.37) | 0.21 | 9.06E-03 | 1.78E-02 | Yes | Up | - |
| G3V5G1 | C14ORF143 | 60/114(52.63) | 0.44 | 9.29E-08 | 6.28E-07 | Yes | Up | - |
| Q8WW33 | GTSF1 | 62/114(54.39) | 0.86 | 2.07E-04 | 6.03E-04 | Yes | Up | - |
| Q9NPB6 | PARD6A | 64/114(56.14) | 0.41 | 2.42E-05 | 8.83E-05 | Yes | Up | - |
| P47972 | NPTX2 | 63/114(55.26) | 0.55 | 4.36E-03 | 9.23E-03 | Yes | Up | - |
| P08910 | ABHD2 | 56/114(49.12) | 0.47 | 2.66E-06 | 1.22E-05 | Yes | Up | - |
| Q5T7M4 | FAM132A | 66/114(57.89) | 0.6 | 2.77E-03 | 6.14E-03 | Yes | Up | - |
| Q9UPY5 | SLC7A11 | 60/114(52.63) | 0.9 | 5.54E-08 | 3.98E-07 | Yes | Up | - |
| Q9Y343 | SNX24 | 62/114(54.39) | 0.35 | 2.39E-05 | 8.75E-05 | Yes | Up | - |
| P18405 | SRD5A1 | 60/114(52.63) | 0.31 | 1.68E-02 | 3.08E-02 | Yes | Up | - |
| Q8NFQ8 | TOR1AIP2 | 57/114(50.0) | 0.24 | 1.48E-03 | 3.51E-03 | Yes | Up | - |
| Q14674 | ESPL1 | 58/114(50.88) | 1.41 | 1.01E-12 | 3.26E-11 | Yes | Up | - |
| Q9H7B4 | SMYD3 | 66/114(57.89) | 0.64 | 7.91E-08 | 5.47E-07 | Yes | Up | - |
| Q9C0J1 | B3GNT4 | 67/114(58.77) | 0.62 | 1.07E-03 | 2.63E-03 | Yes | Up | - |
| Q8WXF1 | PSPC1 | 58/114(50.88) | 0.2 | 4.73E-04 | 1.26E-03 | Yes | Up | - |
| Q8N3S3 | PHTF2 | 55/114(48.25) | 0.33 | 1.51E-04 | 4.54E-04 | Yes | Up | - |
| P29460 | IL12B | 51/114(44.74) | 0.97 | 1.77E-05 | 6.68E-05 | Yes | Up | - |
| Q92698 | RAD54L | 62/114(54.39) | 1.36 | 2.12E-11 | 4.39E-10 | Yes | Up | - |
| Q0VD83 | APOB48R | 63/114(55.26) | 0.82 | 9.71E-11 | 1.59E-09 | Yes | Up | - |
| Q9H6Y2 | WDR55 | 68/114(59.65) | 0.2 | 8.11E-05 | 2.61E-04 | Yes | Up | - |
| O95630 | STAMBP | 54/114(47.37) | 0.13 | 9.76E-03 | 1.90E-02 | Yes | Up | - |
| Q8WU90 | ZC3H15 | 55/114(48.25) | 0.11 | 2.31E-02 | 4.08E-02 | Yes | Up | - |
| Q8IU81 | IRF2BP1 | 64/114(56.14) | 0.22 | 4.03E-04 | 1.09E-03 | Yes | Up | - |
| P01036 | CST4 | 59/114(51.75) | 1.92 | 2.50E-07 | 1.51E-06 | Yes | Up | - |
| Q9HCD6 | TANC2 | 60/114(52.63) | 0.56 | 6.53E-07 | 3.52E-06 | Yes | Up | - |
| O75223 | GGCT | 64/114(56.14) | 0.65 | 4.27E-09 | 4.22E-08 | Yes | Up | - |
| Q9BYN8 | MRPS26 | 64/114(56.14) | 0.26 | 2.49E-04 | 7.11E-04 | Yes | Up | - |
| Q5ZPR3 | CD276 | 60/114(52.63) | 0.55 | 1.31E-11 | 2.87E-10 | Yes | Up | - |
| - | LOC389458 | 64/114(56.14) | 0.9 | 7.23E-06 | 3.00E-05 | Yes | Up | - |
| Q16537 | PPP2R5E | 57/114(50.0) | 0.14 | 1.12E-02 | 2.14E-02 | Yes | Up | - |
| O00267 | SUPT5H | 65/114(57.02) | 0.2 | 2.20E-03 | 5.01E-03 | Yes | Up | - |
| P11142 | HSPA8 | 58/114(50.88) | 0.24 | 1.91E-03 | 4.42E-03 | Yes | Up | - |
| Q86U70 | LDB1 | 54/114(47.37) | 0.12 | 2.87E-02 | 4.96E-02 | Yes | Up | - |
| Q53GS7 | GLE1 | 58/114(50.88) | 0.24 | 1.62E-05 | 6.16E-05 | Yes | Up | - |
| O60291 | MGRN1 | 65/114(57.02) | 0.42 | 5.44E-10 | 7.07E-09 | Yes | Up | - |
| Q6ZV89 | SH2D5 | 60/114(52.63) | 0.4 | 2.87E-02 | 4.96E-02 | Yes | Up | - |
| O75602 | SPAG6 | 60/114(52.63) | 1.41 | 8.90E-08 | 6.06E-07 | Yes | Up | - |
| O00212 | RHOD | 59/114(51.75) | 0.5 | 1.34E-08 | 1.15E-07 | Yes | Up | - |
| P21741 | MDK | 62/114(54.39) | 0.69 | 7.35E-09 | 6.78E-08 | Yes | Up | - |
| Q8IY37 | DHX37 | 61/114(53.51) | 0.31 | 1.46E-06 | 7.16E-06 | Yes | Up | - |
| Q3SXR2 | C3ORF36 | 64/114(56.14) | 1.14 | 3.42E-11 | 6.57E-10 | Yes | Up | - |
| P30273 | FCER1G | 61/114(53.51) | 0.29 | 8.63E-03 | 1.70E-02 | Yes | Up | - |
| Q99755 | PIP5K1A | 56/114(49.12) | 0.36 | 5.04E-07 | 2.81E-06 | Yes | Up | - |
| Q02338 | BDH1 | 61/114(53.51) | 0.37 | 4.58E-04 | 1.23E-03 | Yes | Up | - |
| P07199 | CENPB | 57/114(50.0) | 0.14 | 1.68E-02 | 3.08E-02 | Yes | Up | - |
| O60844 | ZG16 | 26/114(22.81) | 0.81 | 2.05E-02 | 3.67E-02 | Yes | Up | - |
| Q8N2Z9 | APITD1 | 64/114(56.14) | 0.47 | 1.07E-10 | 1.73E-09 | Yes | Up | - |
| Q9UN88 | GABRQ | 41/114(35.96) | 1.93 | 2.60E-08 | 2.06E-07 | Yes | Up | - |
| P60468 | SEC61B | 60/114(52.63) | 0.19 | 1.82E-03 | 4.23E-03 | Yes | Up | - |
| Q7Z736 | PLEKHH3 | 63/114(55.26) | 0.24 | 8.58E-04 | 2.15E-03 | Yes | Up | - |
| Q6P1R4 | DUS1L | 61/114(53.51) | 0.37 | 5.27E-07 | 2.92E-06 | Yes | Up | - |
| Q49AM3 | TTC31 | 59/114(51.75) | 0.1 | 1.37E-02 | 2.57E-02 | Yes | Up | - |
| Q969Z4 | RELT | 70/114(61.4) | 0.79 | 4.06E-11 | 7.61E-10 | Yes | Up | - |
| P01112 | HRAS | 57/114(50.0) | 0.29 | 6.61E-04 | 1.71E-03 | Yes | Up | - |
| Q8N2M4 | TMEM86A | 58/114(50.88) | 0.4 | 3.50E-04 | 9.64E-04 | Yes | Up | - |
| Q15669 | RHOH | 55/114(48.25) | 0.68 | 6.95E-06 | 2.88E-05 | Yes | Up | - |
| O00194 | RAB27B | 59/114(51.75) | 0.37 | 2.59E-02 | 4.53E-02 | Yes | Up | - |
| Q5VT06 | CEP350 | 53/114(46.49) | 0.17 | 2.00E-02 | 3.59E-02 | Yes | Up | - |
| Q6ZVX7 | NCCRP1 | 66/114(57.89) | 0.54 | 1.32E-02 | 2.47E-02 | Yes | Up | - |
| Q9BT73 | PSMG3 | 64/114(56.14) | 0.51 | 8.70E-11 | 1.45E-09 | Yes | Up | - |
| Q9Y2X0 | MED16 | 69/114(60.53) | 0.22 | 2.83E-04 | 7.95E-04 | Yes | Up | - |
| P19971 | TYMP | 57/114(50.0) | 1.06 | 1.02E-15 | 1.12E-13 | Yes | Up | - |
| Q9H0B8 | CRISPLD2 | 59/114(51.75) | 0.24 | 2.71E-02 | 4.71E-02 | Yes | Up | - |
| Q96MR6 | WDR65 | 65/114(57.02) | 0.83 | 2.20E-05 | 8.13E-05 | Yes | Up | - |
| Q9UKI3 | VPREB3 | 56/114(49.12) | 0.43 | 1.47E-02 | 2.74E-02 | Yes | Up | - |
| O75343 | GUCY1B2 | 50/114(43.86) | 1.04 | 5.48E-06 | 2.33E-05 | Yes | Up | - |
| O43561 | LAT | 67/114(58.77) | 0.29 | 1.32E-02 | 2.48E-02 | Yes | Up | - |
| - | FLJ45983 | 57/114(50.0) | 1.13 | 3.36E-07 | 1.95E-06 | Yes | Up | - |
| O95402 | MED26 | 67/114(58.77) | 0.23 | 5.97E-06 | 2.52E-05 | Yes | Up | - |
| Q16763 | UBE2S | 66/114(57.89) | 1.24 | 3.39E-16 | 4.71E-14 | Yes | Up | - |
| O43765 | SGTA | 58/114(50.88) | 0.19 | 3.14E-04 | 8.74E-04 | Yes | Up | - |
| Q8IY33 | MICALL2 | 64/114(56.14) | 0.63 | 1.59E-09 | 1.79E-08 | Yes | Up | - |
| Q15697 | ZNF174 | 57/114(50.0) | 0.3 | 1.11E-08 | 9.73E-08 | Yes | Up | - |
| Q9H2J4 | PDCL3 | 59/114(51.75) | 0.42 | 9.47E-09 | 8.47E-08 | Yes | Up | - |
| Q13064 | MKRN3 | 51/114(44.74) | 1.2 | 1.09E-06 | 5.54E-06 | Yes | Up | - |
| O95864 | FADS2 | 49/114(42.98) | 0.52 | 3.81E-03 | 8.15E-03 | Yes | Up | - |
| P20336 | RAB3A | 64/114(56.14) | 0.75 | 8.48E-11 | 1.42E-09 | Yes | Up | - |
| Q3KQZ1 | SLC25A35 | 62/114(54.39) | 0.21 | 4.84E-03 | 1.01E-02 | Yes | Up | - |
| Q8IY67 | RAVER1 | 64/114(56.14) | 0.52 | 7.25E-13 | 2.48E-11 | Yes | Up | - |
| P84101 | SERF2 | 60/114(52.63) | 0.24 | 2.08E-03 | 4.77E-03 | Yes | Up | - |
| Q9BYB4 | GNB1L | 60/114(52.63) | 0.46 | 1.54E-06 | 7.50E-06 | Yes | Up | - |
| Q9Y285 | FARSA | 65/114(57.02) | 0.49 | 1.34E-10 | 2.09E-09 | Yes | Up | - |
| Q7L513 | FCRLA | 63/114(55.26) | 0.5 | 7.93E-03 | 1.58E-02 | Yes | Up | - |
| Q9GZR2 | REXO4 | 59/114(51.75) | 0.14 | 2.53E-02 | 4.42E-02 | Yes | Up | - |
| Q9Y446 | PKP3 | 56/114(49.12) | 0.67 | 1.18E-05 | 4.67E-05 | Yes | Up | - |
| Q6ZS82 | RGS9BP | 58/114(50.88) | 0.4 | 1.64E-02 | 3.01E-02 | Yes | Up | - |
| Q6W2J9 | BCOR | 63/114(55.26) | 0.26 | 2.47E-03 | 5.55E-03 | Yes | Up | - |
| Q96BD8 | SKA1 | 57/114(50.0) | 1.44 | 2.33E-12 | 6.61E-11 | Yes | Up | - |
| O43808 | SLC25A17 | 61/114(53.51) | 0.31 | 8.95E-06 | 3.64E-05 | Yes | Up | - |
| Q8N8G6 | C15ORF54 | 55/114(48.25) | 1.01 | 1.79E-05 | 6.75E-05 | Yes | Up | - |
| Q9UHD2 | TBK1 | 54/114(47.37) | 0.19 | 1.41E-05 | 5.44E-05 | Yes | Up | - |
| O43194 | GPR39 | 53/114(46.49) | 0.38 | 2.28E-02 | 4.05E-02 | Yes | Up | - |
| Q8IZ52 | CHPF | 64/114(56.14) | 0.67 | 5.04E-09 | 4.88E-08 | Yes | Up | - |
| P61165 | C11ORF10 | 67/114(58.77) | 0.24 | 4.82E-04 | 1.28E-03 | Yes | Up | - |
| Q5VWK0 | NBPF6 | 49/114(42.98) | 1.6 | 1.89E-07 | 1.18E-06 | Yes | Up | - |
| P11509 | CYP2A6 | 54/114(47.37) | 1.02 | 5.37E-04 | 1.42E-03 | Yes | Up | - |
| Q13459 | MYO9B | 62/114(54.39) | 0.24 | 4.57E-06 | 1.98E-05 | Yes | Up | - |
| A4D1S0 | KLRG2 | 56/114(49.12) | 1.01 | 1.98E-06 | 9.42E-06 | Yes | Up | - |
| - | NCRNA00181 | 60/114(52.63) | 0.37 | 1.15E-04 | 3.58E-04 | Yes | Up | - |
| Q96LY2 | CCDC74B | 63/114(55.26) | 0.69 | 6.33E-06 | 2.65E-05 | Yes | Up | - |
| P78527 | PRKDC | 61/114(53.51) | 0.28 | 2.22E-03 | 5.05E-03 | Yes | Up | - |
| Q9HBM0 | VEZT | 51/114(44.74) | 0.16 | 3.36E-03 | 7.28E-03 | Yes | Up | - |
| Q8IY17 | PNPLA6 | 69/114(60.53) | 0.18 | 4.18E-03 | 8.88E-03 | Yes | Up | - |
| P53667 | LIMK1 | 62/114(54.39) | 0.58 | 1.24E-10 | 1.96E-09 | Yes | Up | - |
| P52735 | VAV2 | 59/114(51.75) | 0.54 | 1.26E-08 | 1.08E-07 | Yes | Up | - |
| Q96BW1 | UPRT | 55/114(48.25) | 0.14 | 3.74E-03 | 8.02E-03 | Yes | Up | - |
| O75368 | SH3BGRL | 54/114(47.37) | 0.37 | 2.16E-03 | 4.92E-03 | Yes | Up | - |
| P05549 | TFAP2A | 56/114(49.12) | 0.61 | 3.66E-04 | 1.00E-03 | Yes | Up | - |
| Q9Y6I9 | TEX264 | 70/114(61.4) | 0.34 | 1.29E-07 | 8.40E-07 | Yes | Up | - |
| Q15785 | TOMM34 | 59/114(51.75) | 0.3 | 2.78E-04 | 7.83E-04 | Yes | Up | - |
| Q9HC21 | SLC25A19 | 57/114(50.0) | 0.42 | 4.70E-06 | 2.03E-05 | Yes | Up | - |
| P03971 | AMH | 60/114(52.63) | 0.95 | 1.71E-05 | 6.47E-05 | Yes | Up | - |
| Q9UL19 | RARRES3 | 68/114(59.65) | 0.68 | 1.27E-06 | 6.36E-06 | Yes | Up | - |
| Q9ULW0 | TPX2 | 58/114(50.88) | 1.64 | 5.97E-15 | 4.91E-13 | Yes | Up | - |
| P46926 | GNPDA1 | 56/114(49.12) | 0.24 | 8.55E-07 | 4.48E-06 | Yes | Up | - |
| Q8IY92 | BTBD12 | 63/114(55.26) | 0.46 | 7.24E-10 | 8.98E-09 | Yes | Up | - |
| O00254 | F2RL2 | 61/114(53.51) | 1.12 | 7.56E-08 | 5.25E-07 | Yes | Up | - |
| Q96AW1 | VOPP1 | 60/114(52.63) | 0.47 | 2.19E-06 | 1.03E-05 | Yes | Up | - |
| Q9H175 | CSRNP2 | 61/114(53.51) | 0.32 | 2.51E-06 | 1.17E-05 | Yes | Up | - |
| P46060 | RANGAP1 | 60/114(52.63) | 0.38 | 3.16E-06 | 1.43E-05 | Yes | Up | - |
| Q9HAB8 | PPCS | 60/114(52.63) | 0.19 | 1.60E-03 | 3.77E-03 | Yes | Up | - |
| Q9BTE3 | C10ORF119 | 59/114(51.75) | 0.12 | 6.52E-03 | 1.32E-02 | Yes | Up | - |
| P00751 | CFB | 61/114(53.51) | 1.42 | 8.98E-14 | 4.39E-12 | Yes | Up | - |
| P08240 | SRPR | 58/114(50.88) | 0.15 | 5.08E-03 | 1.06E-02 | Yes | Up | - |
| Q8TDZ2 | MICAL1 | 65/114(57.02) | 0.28 | 4.69E-04 | 1.25E-03 | Yes | Up | - |
| O15160 | POLR1C | 57/114(50.0) | 0.15 | 2.37E-02 | 4.18E-02 | Yes | Up | - |
| P32119 | PRDX2 | 62/114(54.39) | 0.37 | 1.66E-06 | 8.03E-06 | Yes | Up | - |
| Q96CG8 | CTHRC1 | 63/114(55.26) | 1.52 | 4.95E-16 | 6.33E-14 | Yes | Up | - |
| P57052 | RBM11 | 51/114(44.74) | 0.48 | 4.01E-03 | 8.55E-03 | Yes | Up | - |
| P56134 | ATP5J2 | 61/114(53.51) | 0.22 | 8.13E-03 | 1.61E-02 | Yes | Up | - |
| P99999 | CYCS | 57/114(50.0) | 0.27 | 8.13E-04 | 2.05E-03 | Yes | Up | - |
| Q96QF0 | RAB3IP | 57/114(50.0) | 0.53 | 1.38E-06 | 6.83E-06 | Yes | Up | - |
| P42701 | IL12RB1 | 57/114(50.0) | 0.36 | 3.08E-03 | 6.74E-03 | Yes | Up | - |
| Q9NPA1 | KCNMB3 | 56/114(49.12) | 0.18 | 6.39E-03 | 1.30E-02 | Yes | Up | - |
| Q9H6L4 | ARMC7 | 49/114(42.98) | 0.37 | 4.08E-08 | 3.05E-07 | Yes | Up | - |
| Q7L1Q6 | BZW1 | 58/114(50.88) | 0.26 | 3.36E-04 | 9.29E-04 | Yes | Up | - |
| P43358 | MAGEA4 | 35/114(30.7) | 1.48 | 1.18E-03 | 2.86E-03 | Yes | Up | - |
| Q9UKK6 | NXT1 | 61/114(53.51) | 0.27 | 1.92E-05 | 7.21E-05 | Yes | Up | - |
| P01909 | HLA-DQA1 | 64/114(56.14) | 0.49 | 2.71E-05 | 9.76E-05 | Yes | Up | - |
| Q702N8 | XIRP1 | 65/114(57.02) | 1.06 | 4.76E-06 | 2.06E-05 | Yes | Up | - |
| Q9BUN8 | DERL1 | 64/114(56.14) | 0.17 | 1.84E-02 | 3.34E-02 | Yes | Up | - |
| Q6PJ21 | SPSB3 | 62/114(54.39) | 0.17 | 7.93E-03 | 1.58E-02 | Yes | Up | - |
| Q6PL45 | C16ORF79 | 58/114(50.88) | 0.34 | 2.55E-03 | 5.71E-03 | Yes | Up | - |
| Q8TF61 | FBXO41 | 57/114(50.0) | 0.32 | 1.02E-02 | 1.97E-02 | Yes | Up | - |
| Q8N8Y5 | ZFP41 | 55/114(48.25) | 0.18 | 2.06E-02 | 3.69E-02 | Yes | Up | - |
| P78549 | NTHL1 | 65/114(57.02) | 0.57 | 4.73E-09 | 4.62E-08 | Yes | Up | - |
| Q04206 | RELA | 57/114(50.0) | 0.09 | 1.18E-02 | 2.25E-02 | Yes | Up | - |
| Q9C0I9 | LRRC27 | 57/114(50.0) | 0.19 | 6.77E-03 | 1.37E-02 | Yes | Up | - |
| P20645 | M6PR | 54/114(47.37) | 0.14 | 2.55E-04 | 7.27E-04 | Yes | Up | - |
| Q9UK23 | NAGPA | 62/114(54.39) | 0.22 | 8.93E-04 | 2.23E-03 | Yes | Up | - |
| Q15904 | ATP6AP1 | 57/114(50.0) | 0.64 | 5.30E-12 | 1.34E-10 | Yes | Up | - |
| O43504 | HBXIP | 60/114(52.63) | 0.17 | 1.64E-03 | 3.85E-03 | Yes | Up | - |
| Q9BX73 | TM2D2 | 61/114(53.51) | 0.18 | 1.53E-02 | 2.83E-02 | Yes | Up | - |
| Q8NC96 | NECAP1 | 51/114(44.74) | 0.18 | 2.84E-03 | 6.26E-03 | Yes | Up | - |
| Q9BW61 | DDA1 | 63/114(55.26) | 0.21 | 2.58E-05 | 9.34E-05 | Yes | Up | - |
| Q9UPQ8 | DOLK | 59/114(51.75) | 0.3 | 7.64E-07 | 4.05E-06 | Yes | Up | - |
| Q9C010 | PKIB | 50/114(43.86) | 0.99 | 4.91E-08 | 3.58E-07 | Yes | Up | - |
| P84090 | ERH | 65/114(57.02) | 0.35 | 1.14E-07 | 7.46E-07 | Yes | Up | - |
| Q9BTE1 | DCTN5 | 58/114(50.88) | 0.27 | 8.40E-06 | 3.43E-05 | Yes | Up | - |
| P60953 | CDC42 | 59/114(51.75) | 0.16 | 7.86E-04 | 1.99E-03 | Yes | Up | - |
| Q9Y664 | KPTN | 62/114(54.39) | 0.34 | 4.24E-06 | 1.85E-05 | Yes | Up | - |
| Q14444 | CAPRIN1 | 54/114(47.37) | 0.21 | 2.08E-03 | 4.77E-03 | Yes | Up | - |
| Q8N4A0 | GALNT4 | 54/114(47.37) | 0.22 | 2.06E-03 | 4.72E-03 | Yes | Up | - |
| U5GXR9 | MYCL1 | 59/114(51.75) | 0.48 | 5.03E-04 | 1.34E-03 | Yes | Up | - |
| Q9UNW1 | MINPP1 | 54/114(47.37) | 0.15 | 1.87E-02 | 3.38E-02 | Yes | Up | - |
| Q6PJ69 | TRIM65 | 48/114(42.11) | 0.19 | 3.61E-03 | 7.77E-03 | Yes | Up | - |
| Q9Y6J0 | CABIN1 | 60/114(52.63) | 0.18 | 1.17E-03 | 2.85E-03 | Yes | Up | - |
| Q9Y606 | PUS1 | 61/114(53.51) | 0.35 | 1.57E-06 | 7.66E-06 | Yes | Up | - |
| - | C8ORF51 | 60/114(52.63) | 0.83 | 1.20E-08 | 1.04E-07 | Yes | Up | - |
| Q99832 | CCT7 | 59/114(51.75) | 0.16 | 6.32E-03 | 1.29E-02 | Yes | Up | - |
| Q9BTL3 | FAM103A1 | 52/114(45.61) | 0.24 | 7.52E-05 | 2.43E-04 | Yes | Up | - |
| Q9BVM2 | DPCD | 68/114(59.65) | 0.52 | 1.79E-09 | 1.97E-08 | Yes | Up | - |
| O43353 | RIPK2 | 54/114(47.37) | 0.42 | 2.73E-06 | 1.26E-05 | Yes | Up | - |
| Q16531 | DDB1 | 51/114(44.74) | 0.12 | 2.59E-02 | 4.53E-02 | Yes | Up | - |
| Q8N5J2 | FAM63A | 53/114(46.49) | 0.29 | 5.94E-04 | 1.55E-03 | Yes | Up | - |
| O43291 | SPINT2 | 59/114(51.75) | 0.82 | 2.85E-07 | 1.69E-06 | Yes | Up | - |
| Q8NFA0 | USP32 | 58/114(50.88) | 0.29 | 1.90E-04 | 5.57E-04 | Yes | Up | - |
| Q6SJ93 | FAM111B | 63/114(55.26) | 1.24 | 1.02E-09 | 1.22E-08 | Yes | Up | - |
| O43427 | FIBP | 64/114(56.14) | 0.42 | 2.45E-09 | 2.58E-08 | Yes | Up | - |
| Q05048 | CSTF1 | 54/114(47.37) | 0.2 | 1.05E-03 | 2.59E-03 | Yes | Up | - |
| P30926 | CHRNB4 | 57/114(50.0) | 0.54 | 1.82E-02 | 3.31E-02 | Yes | Up | - |
| Q6ISS4 | LAIR2 | 54/114(47.37) | 1.22 | 2.31E-07 | 1.40E-06 | Yes | Up | - |
| P41250 | GARS | 59/114(51.75) | 0.41 | 1.04E-06 | 5.30E-06 | Yes | Up | - |
| Q9NPA8 | ENY2 | 67/114(58.77) | 0.33 | 2.44E-05 | 8.92E-05 | Yes | Up | - |
| Q567V2 | MPV17L2 | 63/114(55.26) | 0.49 | 1.05E-10 | 1.71E-09 | Yes | Up | - |
| Q9H0B6 | KLC2 | 63/114(55.26) | 0.48 | 4.37E-08 | 3.24E-07 | Yes | Up | - |
| Q9ULF5 | SLC39A10 | 50/114(43.86) | 0.16 | 1.31E-02 | 2.46E-02 | Yes | Up | - |
| Q9Y3L3 | SH3BP1 | 60/114(52.63) | 0.54 | 8.89E-10 | 1.08E-08 | Yes | Up | - |
| O94906 | PRPF6 | 63/114(55.26) | 0.19 | 2.08E-03 | 4.76E-03 | Yes | Up | - |
| Q6PGN9 | PSRC1 | 56/114(49.12) | 0.85 | 4.33E-11 | 8.02E-10 | Yes | Up | - |
| P02786 | TFRC | 58/114(50.88) | 0.34 | 5.43E-03 | 1.12E-02 | Yes | Up | - |
| Q9HC52 | CBX8 | 61/114(53.51) | 0.8 | 4.95E-12 | 1.26E-10 | Yes | Up | - |
| Q9BXH1 | BBC3 | 62/114(54.39) | 0.75 | 1.08E-10 | 1.74E-09 | Yes | Up | - |
| O14978 | ZNF263 | 54/114(47.37) | 0.24 | 3.63E-05 | 1.27E-04 | Yes | Up | - |
| B4DWL5 | ZNF434 | 57/114(50.0) | 0.26 | 2.11E-06 | 9.97E-06 | Yes | Up | - |
| Q96KN1 | FAM84B | 54/114(47.37) | 0.3 | 9.43E-03 | 1.84E-02 | Yes | Up | - |
| Q13356 | PPIL2 | 61/114(53.51) | 0.23 | 1.60E-05 | 6.13E-05 | Yes | Up | - |
| Q5T7B8 | KIF24 | 56/114(49.12) | 0.79 | 2.59E-09 | 2.71E-08 | Yes | Up | - |
| O00444 | PLK4 | 53/114(46.49) | 0.87 | 1.85E-09 | 2.02E-08 | Yes | Up | - |
| Q15116 | PDCD1 | 61/114(53.51) | 0.59 | 1.49E-04 | 4.49E-04 | Yes | Up | - |
| Q96Q04 | LMTK3 | 61/114(53.51) | 0.6 | 7.63E-04 | 1.94E-03 | Yes | Up | - |
| Q9H910 | HN1L | 59/114(51.75) | 0.65 | 7.79E-10 | 9.60E-09 | Yes | Up | - |
| Q5UCC4 | C19ORF63 | 66/114(57.89) | 0.38 | 1.59E-06 | 7.73E-06 | Yes | Up | - |
| Q59G12 | TMPO | 62/114(54.39) | 0.32 | 2.72E-04 | 7.70E-04 | Yes | Up | - |
| Q8IWR1 | TRIM59 | 59/114(51.75) | 1.17 | 1.27E-13 | 5.88E-12 | Yes | Up | - |
| Q96JF0 | ST6GAL2 | 60/114(52.63) | 0.92 | 1.45E-07 | 9.33E-07 | Yes | Up | - |
| P47756 | CAPZB | 62/114(54.39) | 0.2 | 4.82E-04 | 1.28E-03 | Yes | Up | - |
| Q5BKU9 | C17ORF90 | 57/114(50.0) | 0.37 | 6.08E-07 | 3.31E-06 | Yes | Up | - |
| P78368 | CSNK1G2 | 68/114(59.65) | 0.16 | 5.16E-03 | 1.07E-02 | Yes | Up | - |
| Q9UET6 | FTSJ1 | 62/114(54.39) | 0.25 | 3.44E-06 | 1.54E-05 | Yes | Up | - |
| Q9BT25 | HAUS8 | 65/114(57.02) | 0.5 | 1.43E-10 | 2.22E-09 | Yes | Up | - |
| Q92752 | TNR | 47/114(41.23) | 0.88 | 8.03E-04 | 2.03E-03 | Yes | Up | - |
| O95070 | YIF1A | 61/114(53.51) | 0.43 | 2.45E-07 | 1.48E-06 | Yes | Up | - |
| Q569K6 | CCDC157 | 62/114(54.39) | 0.51 | 4.07E-05 | 1.41E-04 | Yes | Up | - |
| Q8N6R0 | METTL13 | 59/114(51.75) | 0.32 | 3.48E-08 | 2.65E-07 | Yes | Up | - |
| Q96AV8 | E2F7 | 55/114(48.25) | 1.37 | 3.77E-11 | 7.14E-10 | Yes | Up | - |
| Q9Y2X9 | ZNF281 | 52/114(45.61) | 0.44 | 5.31E-06 | 2.27E-05 | Yes | Up | - |
| Q7Z7N9 | TMEM179B | 63/114(55.26) | 0.22 | 3.15E-04 | 8.77E-04 | Yes | Up | - |
| O75127 | PTCD1 | 62/114(54.39) | 0.31 | 7.31E-07 | 3.89E-06 | Yes | Up | - |
| - | CA5BP | 62/114(54.39) | 0.16 | 1.57E-02 | 2.90E-02 | Yes | Up | - |
| Q00973 | B4GALNT1 | 60/114(52.63) | 0.58 | 1.79E-05 | 6.75E-05 | Yes | Up | - |
| Q13972 | RASGRF1 | 60/114(52.63) | 0.81 | 6.45E-06 | 2.70E-05 | Yes | Up | - |
| Q6UXI9 | NPNT | 55/114(48.25) | 1.22 | 2.91E-14 | 1.79E-12 | Yes | Up | - |
| Q9ULP9 | TBC1D24 | 53/114(46.49) | 0.19 | 2.75E-02 | 4.77E-02 | Yes | Up | - |
| P83876 | TXNL4A | 61/114(53.51) | 0.26 | 1.78E-04 | 5.28E-04 | Yes | Up | - |
| Q9Y2I2 | NTNG1 | 59/114(51.75) | 0.53 | 1.28E-02 | 2.41E-02 | Yes | Up | - |
| Q9BPX1 | HSD17B14 | 69/114(60.53) | 0.45 | 4.89E-05 | 1.65E-04 | Yes | Up | - |
| Q8N594 | MPND | 73/114(64.04) | 0.36 | 1.20E-06 | 6.02E-06 | Yes | Up | - |
| Q99622 | C12ORF57 | 57/114(50.0) | 0.24 | 2.77E-04 | 7.81E-04 | Yes | Up | - |
| Q71RC2 | LARP4 | 56/114(49.12) | 0.28 | 2.23E-04 | 6.45E-04 | Yes | Up | - |
| O43924 | PDE6D | 62/114(54.39) | 0.21 | 2.86E-05 | 1.03E-04 | Yes | Up | - |
| Q8WU67 | ABHD3 | 56/114(49.12) | 0.58 | 2.60E-08 | 2.06E-07 | Yes | Up | - |
| Q969K3 | RNF34 | 59/114(51.75) | 0.26 | 3.15E-06 | 1.43E-05 | Yes | Up | - |
| Q969H4 | CNKSR1 | 61/114(53.51) | 0.59 | 1.69E-04 | 5.04E-04 | Yes | Up | - |
| Q8TED1 | GPX8 | 53/114(46.49) | 0.33 | 1.75E-03 | 4.08E-03 | Yes | Up | - |
| P21754 | ZP3 | 61/114(53.51) | 0.38 | 1.02E-04 | 3.21E-04 | Yes | Up | - |
| O75380 | NDUFS6 | 65/114(57.02) | 0.34 | 1.43E-04 | 4.35E-04 | Yes | Up | - |
| Q99595 | TIMM17A | 59/114(51.75) | 0.53 | 2.35E-08 | 1.89E-07 | Yes | Up | - |
| Q6P587 | FAHD1 | 60/114(52.63) | 0.34 | 4.41E-07 | 2.49E-06 | Yes | Up | - |
| Q9BZD4 | NUF2 | 59/114(51.75) | 1.87 | 1.27E-16 | 2.10E-14 | Yes | Up | - |
| Q8WWL2 | SPIRE2 | 62/114(54.39) | 0.53 | 2.75E-05 | 9.89E-05 | Yes | Up | - |
| O43278 | SPINT1 | 59/114(51.75) | 0.57 | 1.80E-04 | 5.31E-04 | Yes | Up | - |
| Q15369 | TCEB1 | 66/114(57.89) | 0.34 | 9.76E-05 | 3.08E-04 | Yes | Up | - |
| P27797 | CALR | 60/114(52.63) | 0.59 | 1.08E-11 | 2.46E-10 | Yes | Up | - |
| Q99638 | RAD9A | 62/114(54.39) | 0.26 | 1.46E-04 | 4.43E-04 | Yes | Up | - |
| Q96HA8 | WDYHV1 | 61/114(53.51) | 0.32 | 1.05E-04 | 3.28E-04 | Yes | Up | - |
| Q9BZR6 | RTN4R | 63/114(55.26) | 0.54 | 6.93E-06 | 2.88E-05 | Yes | Up | - |
| Q9UG63 | ABCF2 | 62/114(54.39) | 0.13 | 1.03E-02 | 1.98E-02 | Yes | Up | - |
| O43688 | PPAP2C | 59/114(51.75) | 0.58 | 8.42E-05 | 2.69E-04 | Yes | Up | - |
| Q96S55 | WRNIP1 | 59/114(51.75) | 0.3 | 1.07E-06 | 5.42E-06 | Yes | Up | - |
| Q562F6 | SGOL2 | 61/114(53.51) | 0.71 | 3.72E-09 | 3.74E-08 | Yes | Up | - |
| Q99807 | COQ7 | 57/114(50.0) | 0.14 | 1.70E-02 | 3.11E-02 | Yes | Up | - |
| Q15102 | PAFAH1B3 | 60/114(52.63) | 1.3 | 1.56E-15 | 1.60E-13 | Yes | Up | - |
| Q5VWN6 | C10ORF18 | 59/114(51.75) | 0.32 | 3.59E-04 | 9.88E-04 | Yes | Up | - |
| Q9H3S1 | SEMA4A | 52/114(45.61) | 0.42 | 8.19E-04 | 2.07E-03 | Yes | Up | - |
| P01266 | TG | 58/114(50.88) | 0.47 | 2.61E-03 | 5.83E-03 | Yes | Up | - |
| Q9NP87 | POLM | 59/114(51.75) | 0.19 | 4.83E-04 | 1.29E-03 | Yes | Up | - |
| Q8NEE8 | TTC16 | 64/114(56.14) | 0.81 | 2.38E-06 | 1.11E-05 | Yes | Up | - |
| Q9UNI6 | DUSP12 | 56/114(49.12) | 0.22 | 4.34E-05 | 1.49E-04 | Yes | Up | - |
| P14735 | IDE | 53/114(46.49) | 0.22 | 1.14E-02 | 2.17E-02 | Yes | Up | - |
| Q9Y282 | ERGIC3 | 65/114(57.02) | 0.25 | 1.19E-05 | 4.70E-05 | Yes | Up | - |
| P58340 | MLF1 | 67/114(58.77) | 0.33 | 4.16E-04 | 1.13E-03 | Yes | Up | - |
| O95400 | CD2BP2 | 67/114(58.77) | 0.28 | 2.05E-06 | 9.73E-06 | Yes | Up | - |
| Q86X02 | CDR2L | 58/114(50.88) | 0.36 | 2.14E-05 | 7.91E-05 | Yes | Up | - |
| Q92685 | ALG3 | 61/114(53.51) | 0.53 | 8.13E-09 | 7.41E-08 | Yes | Up | - |
| O60479 | DLX3 | 63/114(55.26) | 0.68 | 9.57E-05 | 3.02E-04 | Yes | Up | - |
| M0QX07 | C19ORF62 | 63/114(55.26) | 0.25 | 9.32E-06 | 3.77E-05 | Yes | Up | - |
| Q96T25 | ZIC5 | 45/114(39.47) | 0.64 | 2.02E-02 | 3.63E-02 | Yes | Up | - |
| Q7Z403 | TMC6 | 68/114(59.65) | 0.46 | 3.72E-06 | 1.65E-05 | Yes | Up | - |
| P51530 | DNA2 | 61/114(53.51) | 0.85 | 7.08E-09 | 6.56E-08 | Yes | Up | - |
| Q5JXA9 | SIRPB2 | 61/114(53.51) | 0.31 | 8.23E-03 | 1.63E-02 | Yes | Up | - |
| Q9NYU2 | UGGT1 | 51/114(44.74) | 0.18 | 3.23E-03 | 7.03E-03 | Yes | Up | - |
| P16435 | POR | 64/114(56.14) | 0.19 | 2.72E-02 | 4.73E-02 | Yes | Up | - |
| Q92963 | RIT1 | 54/114(47.37) | 0.23 | 4.77E-05 | 1.62E-04 | Yes | Up | - |
| O15027 | SEC16A | 59/114(51.75) | 0.43 | 2.95E-08 | 2.29E-07 | Yes | Up | - |
| Q15047 | SETDB1 | 54/114(47.37) | 0.28 | 2.21E-06 | 1.04E-05 | Yes | Up | - |
| Q15125 | EBP | 64/114(56.14) | 0.52 | 7.09E-08 | 4.97E-07 | Yes | Up | - |
| Q96DZ1 | ERLEC1 | 57/114(50.0) | 0.17 | 1.32E-03 | 3.18E-03 | Yes | Up | - |
| Q16540 | MRPL23 | 64/114(56.14) | 0.34 | 1.58E-05 | 6.05E-05 | Yes | Up | - |
| Q86X60 | FAM72B | 61/114(53.51) | 1.2 | 4.64E-12 | 1.19E-10 | Yes | Up | - |
| Q6PP77 | XKRX | 61/114(53.51) | 0.48 | 2.69E-03 | 5.99E-03 | Yes | Up | - |
| - | FLJ23867 | 64/114(56.14) | 0.44 | 2.94E-04 | 8.24E-04 | Yes | Up | - |
| Q96GA7 | SDSL | 64/114(56.14) | 0.73 | 4.28E-12 | 1.11E-10 | Yes | Up | - |
| Q15760 | GPR19 | 62/114(54.39) | 0.84 | 2.50E-07 | 1.51E-06 | Yes | Up | - |
| Q96AG4 | LRRC59 | 55/114(48.25) | 0.63 | 4.06E-09 | 4.04E-08 | Yes | Up | - |
| Q9H5K3 | SGK196 | 52/114(45.61) | 0.31 | 9.84E-03 | 1.91E-02 | Yes | Up | - |
| P26232 | CTNNA2 | 61/114(53.51) | 0.81 | 2.75E-04 | 7.76E-04 | Yes | Up | - |
| Q8NBT2 | SPC24 | 63/114(55.26) | 1.77 | 1.31E-13 | 6.01E-12 | Yes | Up | - |
| Q4KMP7 | TBC1D10B | 64/114(56.14) | 0.38 | 2.64E-10 | 3.74E-09 | Yes | Up | - |
| Q9BRJ6 | C7ORF50 | 70/114(61.4) | 0.35 | 1.63E-06 | 7.92E-06 | Yes | Up | - |
| P48735 | IDH2 | 64/114(56.14) | 0.69 | 1.23E-08 | 1.06E-07 | Yes | Up | - |
| Q96FQ6 | S100A16 | 61/114(53.51) | 0.4 | 4.51E-04 | 1.21E-03 | Yes | Up | - |
| P78508 | KCNJ10 | 66/114(57.89) | 1.07 | 1.62E-09 | 1.82E-08 | Yes | Up | - |
| P53365 | ARFIP2 | 59/114(51.75) | 0.33 | 1.05E-04 | 3.30E-04 | Yes | Up | - |
| Q9Y330 | ZBTB12 | 51/114(44.74) | 0.48 | 3.17E-06 | 1.43E-05 | Yes | Up | - |
| O95727 | CRTAM | 60/114(52.63) | 0.47 | 4.09E-04 | 1.11E-03 | Yes | Up | - |
| Q99795 | GPA33 | 49/114(42.98) | 0.51 | 5.38E-04 | 1.42E-03 | Yes | Up | - |
| Q6NTF7 | APOBEC3H | 64/114(56.14) | 0.68 | 2.57E-06 | 1.19E-05 | Yes | Up | - |
| P34059 | GALNS | 57/114(50.0) | 0.28 | 6.61E-04 | 1.71E-03 | Yes | Up | - |
| P61081 | UBE2M | 68/114(59.65) | 0.29 | 4.70E-05 | 1.60E-04 | Yes | Up | - |
| P83436 | COG7 | 63/114(55.26) | 0.34 | 6.16E-07 | 3.34E-06 | Yes | Up | - |
| Q8N427 | TXNDC3 | 63/114(55.26) | 0.95 | 4.15E-09 | 4.12E-08 | Yes | Up | - |
| Q15392 | DHCR24 | 59/114(51.75) | 0.41 | 1.30E-03 | 3.13E-03 | Yes | Up | - |
| Q6UX39 | AMTN | 36/114(31.58) | 1.66 | 3.97E-08 | 2.98E-07 | Yes | Up | - |
| Q7L3T8 | PARS2 | 61/114(53.51) | 0.19 | 4.22E-03 | 8.95E-03 | Yes | Up | - |
| O14917 | PCDH17 | 62/114(54.39) | 0.76 | 4.05E-08 | 3.03E-07 | Yes | Up | - |
| Q15435 | PPP1R7 | 57/114(50.0) | 0.12 | 9.97E-03 | 1.93E-02 | Yes | Up | - |
| Q6NS38 | ALKBH2 | 61/114(53.51) | 0.19 | 9.15E-03 | 1.79E-02 | Yes | Up | - |
| Q969M7 | UBE2F | 55/114(48.25) | 0.25 | 1.78E-03 | 4.14E-03 | Yes | Up | - |
| P24864 | CCNE1 | 58/114(50.88) | 0.9 | 2.44E-08 | 1.95E-07 | Yes | Up | - |
| Q9GZQ3 | COMMD5 | 64/114(56.14) | 0.42 | 6.94E-09 | 6.43E-08 | Yes | Up | - |
| B4DLH2 | C2ORF18 | 57/114(50.0) | 0.37 | 1.69E-06 | 8.20E-06 | Yes | Up | - |
| Q9BYN7 | ZNF341 | 61/114(53.51) | 0.25 | 3.37E-05 | 1.19E-04 | Yes | Up | - |
| P51397 | DAP | 66/114(57.89) | 0.28 | 6.33E-05 | 2.09E-04 | Yes | Up | - |
| Q99836 | MYD88 | 63/114(55.26) | 0.28 | 1.38E-05 | 5.34E-05 | Yes | Up | - |
| - | LOC644936 | 59/114(51.75) | 0.31 | 3.77E-04 | 1.03E-03 | Yes | Up | - |
| Q96A56 | TP53INP1 | 56/114(49.12) | 0.49 | 8.40E-06 | 3.43E-05 | Yes | Up | - |
| Q15053 | KIAA0040 | 56/114(49.12) | 0.32 | 9.91E-04 | 2.45E-03 | Yes | Up | - |
| Q13057 | COASY | 60/114(52.63) | 0.2 | 2.26E-03 | 5.14E-03 | Yes | Up | - |
| Q96S59 | RANBP9 | 58/114(50.88) | 0.22 | 8.35E-04 | 2.10E-03 | Yes | Up | - |
| Q86VQ3 | TXNDC2 | 53/114(46.49) | 0.85 | 3.72E-06 | 1.65E-05 | Yes | Up | - |
| P82673 | MRPS35 | 54/114(47.37) | 0.25 | 6.91E-04 | 1.78E-03 | Yes | Up | - |
| Q16612 | C5ORF13 | 59/114(51.75) | 0.74 | 1.47E-10 | 2.27E-09 | Yes | Up | - |
| Q8N1S5 | SLC39A11 | 60/114(52.63) | 0.82 | 4.73E-11 | 8.58E-10 | Yes | Up | - |
| P14859 | POU2F1 | 57/114(50.0) | 0.39 | 5.47E-10 | 7.09E-09 | Yes | Up | - |
| O60423 | ATP8B3 | 68/114(59.65) | 0.56 | 9.33E-06 | 3.77E-05 | Yes | Up | - |
| Q9BXM9 | FSD1L | 56/114(49.12) | 0.26 | 2.24E-02 | 3.97E-02 | Yes | Up | - |
| Q8IXM6 | NRM | 59/114(51.75) | 0.23 | 6.25E-03 | 1.28E-02 | Yes | Up | - |
| P00748 | F12 | 62/114(54.39) | 1.17 | 1.29E-08 | 1.11E-07 | Yes | Up | - |
| - | LOC100134713 | 58/114(50.88) | 0.42 | 4.66E-06 | 2.02E-05 | Yes | Up | - |
| O14920 | IKBKB | 62/114(54.39) | 0.24 | 1.19E-03 | 2.89E-03 | Yes | Up | - |
| P43694 | GATA4 | 54/114(47.37) | 1.67 | 9.18E-08 | 6.22E-07 | Yes | Up | - |
| O60732 | MAGEC1 | 42/114(36.84) | 1.44 | 1.36E-05 | 5.28E-05 | Yes | Up | - |
| Q9H3Y6 | SRMS | 58/114(50.88) | 1.24 | 1.14E-07 | 7.48E-07 | Yes | Up | - |
| P52292 | KPNA2 | 58/114(50.88) | 1.05 | 1.18E-12 | 3.73E-11 | Yes | Up | - |
| P30520 | ADSS | 62/114(54.39) | 0.49 | 4.40E-08 | 3.25E-07 | Yes | Up | - |
| Q6P2D8 | XRRA1 | 54/114(47.37) | 0.21 | 4.28E-04 | 1.15E-03 | Yes | Up | - |
| P18085 | ARF4 | 59/114(51.75) | 0.31 | 3.56E-05 | 1.25E-04 | Yes | Up | - |
| P35249 | RFC4 | 57/114(50.0) | 0.57 | 1.33E-09 | 1.53E-08 | Yes | Up | - |
| P09544 | WNT2 | 60/114(52.63) | 0.63 | 1.30E-04 | 3.98E-04 | Yes | Up | - |
| O00255 | MEN1 | 60/114(52.63) | 0.35 | 2.91E-07 | 1.73E-06 | Yes | Up | - |
| B4DNK4 | PKM2 | 63/114(55.26) | 0.62 | 4.54E-11 | 8.33E-10 | Yes | Up | - |
| Q99879 | HIST1H2BM | 33/114(28.95) | 1.37 | 3.18E-04 | 8.85E-04 | Yes | Up | - |
| P14780 | MMP9 | 65/114(57.02) | 1.55 | 1.45E-12 | 4.38E-11 | Yes | Up | - |
| Q5BLP8 | C4ORF48 | 65/114(57.02) | 0.84 | 6.09E-07 | 3.31E-06 | Yes | Up | - |
| Q14CZ0 | C16ORF72 | 54/114(47.37) | 0.14 | 6.78E-03 | 1.37E-02 | Yes | Up | - |
| Q8NI17 | IL31RA | 53/114(46.49) | 0.65 | 6.98E-03 | 1.41E-02 | Yes | Up | - |
| Q9NTJ3 | SMC4 | 54/114(47.37) | 0.75 | 1.48E-09 | 1.68E-08 | Yes | Up | - |
| Q9NPE6 | SPAG4 | 61/114(53.51) | 0.84 | 4.58E-09 | 4.49E-08 | Yes | Up | - |
| P05783 | KRT18 | 66/114(57.89) | 0.97 | 4.24E-08 | 3.16E-07 | Yes | Up | - |
| Q3KQV9 | UAP1L1 | 60/114(52.63) | 0.36 | 9.04E-08 | 6.14E-07 | Yes | Up | - |
| Q8WUH6 | C12ORF23 | 55/114(48.25) | 0.34 | 3.92E-06 | 1.72E-05 | Yes | Up | - |
| Q8TC76 | FAM110B | 58/114(50.88) | 0.27 | 1.02E-02 | 1.98E-02 | Yes | Up | - |
| Q96HZ4 | HES6 | 65/114(57.02) | 1.2 | 3.29E-13 | 1.28E-11 | Yes | Up | - |
| P08476 | INHBA | 61/114(53.51) | 2.15 | 3.06E-25 | 1.18E-21 | Yes | Up | - |
| Q75QN2 | INTS8 | 59/114(51.75) | 0.38 | 2.18E-06 | 1.03E-05 | Yes | Up | - |
| Q8N8W4 | PNPLA1 | 52/114(45.61) | 0.81 | 7.04E-04 | 1.81E-03 | Yes | Up | - |
| Q6P1L8 | MRPL14 | 65/114(57.02) | 0.56 | 4.83E-10 | 6.37E-09 | Yes | Up | - |
| - | MIR155HG | 61/114(53.51) | 0.38 | 4.66E-03 | 9.79E-03 | Yes | Up | - |
| Q9P0V3 | SH3BP4 | 57/114(50.0) | 0.23 | 1.40E-02 | 2.61E-02 | Yes | Up | - |
| Q16651 | PRSS8 | 60/114(52.63) | 0.85 | 8.04E-05 | 2.59E-04 | Yes | Up | - |
| P18577 | RHCE | 61/114(53.51) | 0.84 | 1.45E-07 | 9.33E-07 | Yes | Up | - |
| Q96K49 | TMEM87B | 53/114(46.49) | 0.3 | 5.76E-04 | 1.51E-03 | Yes | Up | - |
| Q7Z6M3 | C17ORF60 | 63/114(55.26) | 0.27 | 2.88E-02 | 4.98E-02 | Yes | Up | - |
| Q05D60 | CCDC67 | 50/114(43.86) | 0.61 | 2.55E-03 | 5.69E-03 | Yes | Up | - |
| Q96F63 | CCDC97 | 60/114(52.63) | 0.13 | 1.08E-03 | 2.64E-03 | Yes | Up | - |
| Q9BSH5 | HDHD3 | 65/114(57.02) | 0.16 | 1.73E-02 | 3.15E-02 | Yes | Up | - |
| U3KQE1 | DUSP13 | 59/114(51.75) | 0.81 | 1.65E-03 | 3.87E-03 | Yes | Up | - |
| P57057 | SLC37A1 | 63/114(55.26) | 0.71 | 4.73E-11 | 8.58E-10 | Yes | Up | - |
| O43805 | SSNA1 | 63/114(55.26) | 0.48 | 4.36E-10 | 5.82E-09 | Yes | Up | - |
| Q9P0J0 | NDUFA13 | 64/114(56.14) | 0.44 | 2.29E-08 | 1.85E-07 | Yes | Up | - |
| O75354 | ENTPD6 | 57/114(50.0) | 0.27 | 1.43E-04 | 4.34E-04 | Yes | Up | - |
| Q15506 | SPA17 | 60/114(52.63) | 0.42 | 1.81E-04 | 5.35E-04 | Yes | Up | - |
| O43914 | TYROBP | 67/114(58.77) | 0.38 | 6.49E-04 | 1.68E-03 | Yes | Up | - |
| P57081 | WDR4 | 58/114(50.88) | 0.2 | 1.31E-02 | 2.46E-02 | Yes | Up | - |
| B5LMG6 | C13ORF34 | 52/114(45.61) | 0.25 | 5.74E-03 | 1.18E-02 | Yes | Up | - |
| O43261 | DLEU1 | 47/114(41.23) | 0.35 | 2.78E-04 | 7.83E-04 | Yes | Up | - |
| P22234 | PAICS | 54/114(47.37) | 0.45 | 3.72E-06 | 1.65E-05 | Yes | Up | - |
| Q9UKB3 | DNAJC12 | 61/114(53.51) | 0.84 | 9.30E-06 | 3.76E-05 | Yes | Up | - |
| Q6IBW4 | NCAPH2 | 66/114(57.89) | 0.16 | 1.53E-02 | 2.83E-02 | Yes | Up | - |
| P12544 | GZMA | 58/114(50.88) | 0.37 | 1.24E-02 | 2.35E-02 | Yes | Up | - |
| Q504T8 | MIDN | 72/114(63.16) | 0.38 | 1.48E-05 | 5.70E-05 | Yes | Up | - |
| Q9BZM6 | ULBP1 | 62/114(54.39) | 1.11 | 1.80E-08 | 1.50E-07 | Yes | Up | - |
| Q8N2C9 | C21ORF128 | 55/114(48.25) | 0.95 | 3.42E-05 | 1.20E-04 | Yes | Up | - |
| Q9Y6B6 | SAR1B | 56/114(49.12) | 0.26 | 2.79E-03 | 6.17E-03 | Yes | Up | - |
| - | TOP1P1 | 65/114(57.02) | 0.25 | 1.34E-05 | 5.23E-05 | Yes | Up | - |
| Q8WXK3 | ASB13 | 61/114(53.51) | 0.5 | 9.46E-08 | 6.39E-07 | Yes | Up | - |
| Q6NUK1 | SLC25A24 | 51/114(44.74) | 0.2 | 5.86E-03 | 1.21E-02 | Yes | Up | - |
| P01008 | SERPINC1 | 53/114(46.49) | 0.54 | 3.16E-03 | 6.90E-03 | Yes | Up | - |
| Q14320 | FAM50A | 58/114(50.88) | 0.36 | 2.07E-05 | 7.69E-05 | Yes | Up | - |
| P56381 | ATP5E | 60/114(52.63) | 0.2 | 8.49E-03 | 1.68E-02 | Yes | Up | - |
| P42262 | GRIA2 | 61/114(53.51) | 1.13 | 9.33E-05 | 2.96E-04 | Yes | Up | - |
| Q8N7L0 | C13ORF30 | 49/114(42.98) | 0.94 | 1.23E-03 | 2.97E-03 | Yes | Up | - |
| Q92813 | DIO2 | 61/114(53.51) | 0.58 | 4.34E-04 | 1.17E-03 | Yes | Up | - |
| Q99848 | EBNA1BP2 | 62/114(54.39) | 0.36 | 7.01E-07 | 3.75E-06 | Yes | Up | - |
| P62487 | POLR2G | 57/114(50.0) | 0.27 | 6.04E-06 | 2.55E-05 | Yes | Up | - |
| Q9NR99 | MXRA5 | 63/114(55.26) | 0.78 | 3.71E-09 | 3.73E-08 | Yes | Up | - |
| A1KZ92 | PXDNL | 58/114(50.88) | 0.51 | 7.47E-03 | 1.50E-02 | Yes | Up | - |
| O75175 | CNOT3 | 67/114(58.77) | 0.31 | 2.16E-09 | 2.32E-08 | Yes | Up | - |
| Q8IYM1 | Sep-12 | 48/114(42.11) | 1.34 | 6.34E-08 | 4.49E-07 | Yes | Up | - |
| Q8IZC4 | RTKN2 | 56/114(49.12) | 0.92 | 2.98E-08 | 2.31E-07 | Yes | Up | - |
| O95900 | TRUB2 | 60/114(52.63) | 0.31 | 1.59E-07 | 1.01E-06 | Yes | Up | - |
| O60216 | RAD21 | 61/114(53.51) | 0.31 | 3.60E-03 | 7.76E-03 | Yes | Up | - |
| Q8NEG7 | FAM116B | 62/114(54.39) | 0.34 | 1.33E-05 | 5.17E-05 | Yes | Up | - |
| Q6PCT2 | FBXL19 | 62/114(54.39) | 0.65 | 2.97E-11 | 5.85E-10 | Yes | Up | - |
| O14683 | TP53I11 | 57/114(50.0) | 0.49 | 5.50E-08 | 3.96E-07 | Yes | Up | - |
| Q01167 | FOXK2 | 50/114(43.86) | 0.27 | 1.85E-04 | 5.45E-04 | Yes | Up | - |
| P11473 | VDR | 63/114(55.26) | 0.47 | 7.19E-05 | 2.34E-04 | Yes | Up | - |
| Q9BQE5 | APOL2 | 58/114(50.88) | 0.19 | 1.09E-02 | 2.09E-02 | Yes | Up | - |
| P80303 | NUCB2 | 54/114(47.37) | 0.51 | 4.91E-06 | 2.11E-05 | Yes | Up | - |
| P55854 | SUMO3 | 60/114(52.63) | 0.22 | 4.25E-05 | 1.46E-04 | Yes | Up | - |
| Q15477 | SKIV2L | 61/114(53.51) | 0.22 | 9.54E-05 | 3.01E-04 | Yes | Up | - |
| O43520 | ATP8B1 | 59/114(51.75) | 0.57 | 1.21E-07 | 7.90E-07 | Yes | Up | - |
| Q9Y697 | NFS1 | 59/114(51.75) | 0.2 | 8.72E-04 | 2.18E-03 | Yes | Up | - |
| P43362 | MAGEA9B | 33/114(28.95) | 1.28 | 3.32E-04 | 9.18E-04 | Yes | Up | - |
| P55036 | PSMD4 | 58/114(50.88) | 0.4 | 1.54E-07 | 9.84E-07 | Yes | Up | - |
| Q8TAC2 | JOSD2 | 64/114(56.14) | 0.35 | 5.18E-04 | 1.37E-03 | Yes | Up | - |
| Q9Y4A8 | NFE2L3 | 58/114(50.88) | 0.72 | 2.38E-10 | 3.42E-09 | Yes | Up | - |
| Q6IN84 | MRM1 | 62/114(54.39) | 0.21 | 2.19E-02 | 3.90E-02 | Yes | Up | - |
| Q6ISU1 | PTCRA | 58/114(50.88) | 0.99 | 2.16E-06 | 1.02E-05 | Yes | Up | - |
| Q9NR71 | ASAH2 | 58/114(50.88) | 0.43 | 9.32E-03 | 1.82E-02 | Yes | Up | - |
| Q6P5W5 | SLC39A4 | 59/114(51.75) | 0.72 | 1.61E-07 | 1.02E-06 | Yes | Up | - |
| Q96B54 | ZNF428 | 70/114(61.4) | 0.31 | 1.28E-05 | 5.01E-05 | Yes | Up | - |
| P62306 | SNRPF | 64/114(56.14) | 0.43 | 1.58E-09 | 1.78E-08 | Yes | Up | - |
| P21926 | CD9 | 62/114(54.39) | 0.48 | 2.55E-06 | 1.18E-05 | Yes | Up | - |
| Q96NZ1 | FOXN4 | 54/114(47.37) | 1.23 | 2.68E-05 | 9.65E-05 | Yes | Up | - |
| Q5BIV9 | SPRN | 57/114(50.0) | 0.22 | 4.17E-03 | 8.86E-03 | Yes | Up | - |
| O95470 | SGPL1 | 56/114(49.12) | 0.4 | 2.60E-07 | 1.56E-06 | Yes | Up | - |
| Q13263 | TRIM28 | 67/114(58.77) | 0.34 | 6.17E-07 | 3.35E-06 | Yes | Up | - |
| - | LOC344967 | 59/114(51.75) | 0.6 | 2.65E-06 | 1.22E-05 | Yes | Up | - |
| Q6ZP65 | CCDC64 | 63/114(55.26) | 1.41 | 6.58E-13 | 2.28E-11 | Yes | Up | - |
| P31276 | HOXC13 | 63/114(55.26) | 1.1 | 2.45E-06 | 1.14E-05 | Yes | Up | - |
| P43004 | SLC1A2 | 55/114(48.25) | 0.39 | 1.98E-02 | 3.56E-02 | Yes | Up | - |
| Q8N6K0 | C13ORF16 | 54/114(47.37) | 0.67 | 4.63E-04 | 1.24E-03 | Yes | Up | - |
| O75899 | GABBR2 | 52/114(45.61) | 0.54 | 2.06E-02 | 3.68E-02 | Yes | Up | - |
| P32971 | TNFSF8 | 58/114(50.88) | 0.26 | 2.61E-02 | 4.56E-02 | Yes | Up | - |
| Q15437 | SEC23B | 49/114(42.98) | 0.33 | 1.16E-04 | 3.60E-04 | Yes | Up | - |
| P19440 | GGT1 | 54/114(47.37) | 0.4 | 1.30E-02 | 2.44E-02 | Yes | Up | - |
| P27707 | DCK | 56/114(49.12) | 0.31 | 3.52E-05 | 1.24E-04 | Yes | Up | - |
| Q400G9 | AMZ1 | 68/114(59.65) | 1.54 | 1.30E-15 | 1.38E-13 | Yes | Up | - |
| Q9Y3B2 | EXOSC1 | 61/114(53.51) | 0.29 | 4.99E-08 | 3.63E-07 | Yes | Up | - |
| P61073 | CXCR4 | 63/114(55.26) | 0.57 | 4.74E-06 | 2.05E-05 | Yes | Up | - |
| Q9UPR6 | ZFR2 | 58/114(50.88) | 0.66 | 4.30E-03 | 9.10E-03 | Yes | Up | - |
| Q8N9B4 | ANKRD42 | 59/114(51.75) | 0.19 | 1.04E-02 | 2.01E-02 | Yes | Up | - |
| Q6NSJ5 | LRRC8E | 63/114(55.26) | 0.72 | 3.05E-07 | 1.79E-06 | Yes | Up | - |
| Q8TCU6 | PREX1 | 57/114(50.0) | 0.65 | 3.18E-07 | 1.86E-06 | Yes | Up | - |
| Q8NEA6 | GLIS3 | 61/114(53.51) | 0.36 | 4.91E-03 | 1.03E-02 | Yes | Up | - |
| Q9P0K7 | RAI14 | 62/114(54.39) | 0.47 | 8.31E-07 | 4.36E-06 | Yes | Up | - |
| P50281 | MMP14 | 63/114(55.26) | 0.75 | 4.61E-11 | 8.40E-10 | Yes | Up | - |
| Q9UHJ3 | SFMBT1 | 53/114(46.49) | 0.17 | 2.32E-02 | 4.11E-02 | Yes | Up | - |
| Q9BZW4 | TM6SF2 | 61/114(53.51) | 0.8 | 8.57E-09 | 7.75E-08 | Yes | Up | - |
| P35606 | COPB2 | 57/114(50.0) | 0.43 | 1.18E-07 | 7.73E-07 | Yes | Up | - |
| Q15831 | STK11 | 69/114(60.53) | 0.19 | 3.62E-03 | 7.79E-03 | Yes | Up | - |
| Q14244 | MAP7 | 58/114(50.88) | 0.42 | 2.64E-04 | 7.51E-04 | Yes | Up | - |
| Q8N9I5 | FADS6 | 47/114(41.23) | 1.22 | 4.98E-05 | 1.68E-04 | Yes | Up | - |
| Q96LU5 | IMMP1L | 56/114(49.12) | 0.17 | 6.09E-03 | 1.25E-02 | Yes | Up | - |
| Q16610 | ECM1 | 61/114(53.51) | 0.72 | 1.96E-05 | 7.34E-05 | Yes | Up | - |
| Q8WWV6 | FCAMR | 55/114(48.25) | 0.79 | 6.18E-03 | 1.26E-02 | Yes | Up | - |
| Q9UH03 | Sep-03 | 61/114(53.51) | 0.49 | 5.93E-03 | 1.22E-02 | Yes | Up | - |
| Q08397 | LOXL1 | 62/114(54.39) | 0.9 | 6.67E-11 | 1.15E-09 | Yes | Up | - |
| O14503 | BHLHE40 | 66/114(57.89) | 0.37 | 1.64E-04 | 4.91E-04 | Yes | Up | - |
| P49760 | CLK2 | 57/114(50.0) | 0.22 | 3.48E-04 | 9.58E-04 | Yes | Up | - |
| P43681 | CHRNA4 | 41/114(35.96) | 1.08 | 6.12E-04 | 1.59E-03 | Yes | Up | - |
| Q13387 | MAPK8IP2 | 61/114(53.51) | 1.34 | 1.27E-12 | 3.93E-11 | Yes | Up | - |
| O96024 | B3GALT4 | 64/114(56.14) | 0.21 | 1.26E-02 | 2.38E-02 | Yes | Up | - |
| Q86WQ0 | NR2C2AP | 58/114(50.88) | 0.57 | 1.98E-13 | 8.38E-12 | Yes | Up | - |
| Q15013 | MAD2L1BP | 57/114(50.0) | 0.3 | 1.09E-07 | 7.22E-07 | Yes | Up | - |
| Q8N159 | NAGS | 60/114(52.63) | 0.42 | 2.47E-03 | 5.55E-03 | Yes | Up | - |
| P49643 | PRIM2 | 59/114(51.75) | 0.42 | 5.14E-07 | 2.85E-06 | Yes | Up | - |
| Q8IU80 | TMPRSS6 | 66/114(57.89) | 0.9 | 9.14E-05 | 2.90E-04 | Yes | Up | - |
| P57086 | SCAND1 | 76/114(66.67) | 0.44 | 6.78E-07 | 3.64E-06 | Yes | Up | - |
| Q86YV0 | RASAL3 | 65/114(57.02) | 0.32 | 1.79E-03 | 4.16E-03 | Yes | Up | - |
| Q9NP62 | GCM1 | 57/114(50.0) | 0.7 | 3.66E-04 | 1.00E-03 | Yes | Up | - |
| Q00987 | MDM2 | 50/114(43.86) | 0.22 | 2.09E-04 | 6.08E-04 | Yes | Up | - |
| A2IDD5 | CCDC78 | 55/114(48.25) | 1.92 | 6.68E-19 | 2.48E-16 | Yes | Up | - |
| Q8NBP7 | PCSK9 | 54/114(47.37) | 0.56 | 3.73E-03 | 7.99E-03 | Yes | Up | - |
| O95858 | TSPAN15 | 62/114(54.39) | 0.32 | 3.83E-03 | 8.19E-03 | Yes | Up | - |
| Q6P3W7 | SCYL2 | 56/114(49.12) | 0.16 | 1.97E-02 | 3.55E-02 | Yes | Up | - |
| Q16740 | CLPP | 67/114(58.77) | 0.33 | 6.97E-06 | 2.90E-05 | Yes | Up | - |
| Q5TA50 | GLTPD1 | 66/114(57.89) | 0.35 | 3.07E-05 | 1.09E-04 | Yes | Up | - |
| Q92621 | NUP205 | 59/114(51.75) | 0.18 | 1.57E-02 | 2.90E-02 | Yes | Up | - |
| Q8IXT5 | RBM12B | 51/114(44.74) | 0.19 | 6.91E-03 | 1.40E-02 | Yes | Up | - |
| Q5FWE3 | PRRT3 | 62/114(54.39) | 0.91 | 2.50E-10 | 3.58E-09 | Yes | Up | - |
| Q3SXP7 | KIAA1644 | 62/114(54.39) | 0.72 | 1.17E-06 | 5.91E-06 | Yes | Up | - |
| P49802 | RGS7 | 65/114(57.02) | 1.37 | 3.61E-08 | 2.74E-07 | Yes | Up | - |
| Q8NBZ0 | INO80E | 67/114(58.77) | 0.25 | 1.19E-06 | 6.00E-06 | Yes | Up | - |
| Q4LE39 | ARID4B | 52/114(45.61) | 0.16 | 1.07E-02 | 2.06E-02 | Yes | Up | - |
| Q7Z4P6 | C1ORF86 | 67/114(58.77) | 0.33 | 9.13E-05 | 2.90E-04 | Yes | Up | - |
| - | LOC285359 | 65/114(57.02) | 0.17 | 5.82E-03 | 1.20E-02 | Yes | Up | - |
| Q12905 | ILF2 | 61/114(53.51) | 0.42 | 9.76E-07 | 5.03E-06 | Yes | Up | - |
| Q7Z4K8 | TRIM46 | 57/114(50.0) | 0.69 | 5.80E-06 | 2.45E-05 | Yes | Up | - |
| Q6ZVW7 | IL17REL | 53/114(46.49) | 1.5 | 1.44E-10 | 2.23E-09 | Yes | Up | - |
| P0C025 | NUDT17 | 58/114(50.88) | 0.18 | 2.57E-02 | 4.49E-02 | Yes | Up | - |
| Q68DK7 | MSL1 | 54/114(47.37) | 0.17 | 6.15E-03 | 1.26E-02 | Yes | Up | - |
| P58107 | EPPK1 | 62/114(54.39) | 0.56 | 1.86E-03 | 4.31E-03 | Yes | Up | - |
| Q9HBG6 | IFT122 | 59/114(51.75) | 0.23 | 4.94E-04 | 1.31E-03 | Yes | Up | - |
| - | LOC100128788 | 59/114(51.75) | 0.61 | 9.74E-07 | 5.02E-06 | Yes | Up | - |
| Q9BV68 | RNF126 | 69/114(60.53) | 0.18 | 4.24E-03 | 9.00E-03 | Yes | Up | - |
| Q96HB5 | CCDC120 | 59/114(51.75) | 0.33 | 5.90E-04 | 1.54E-03 | Yes | Up | - |
| Q5JT82 | KLF17 | 64/114(56.14) | 1.06 | 3.90E-06 | 1.72E-05 | Yes | Up | - |
| Q96FM1 | PGAP3 | 65/114(57.02) | 0.28 | 8.18E-03 | 1.62E-02 | Yes | Up | - |
| Q8WXC3 | PYDC1 | 55/114(48.25) | 2.74 | 6.59E-22 | 7.95E-19 | Yes | Up | - |
| Q9BYG5 | PARD6B | 57/114(50.0) | 0.94 | 1.86E-08 | 1.54E-07 | Yes | Up | - |
| Q12789 | GTF3C1 | 63/114(55.26) | 0.47 | 1.54E-09 | 1.74E-08 | Yes | Up | - |
| Q92917 | GPKOW | 62/114(54.39) | 0.19 | 2.13E-04 | 6.19E-04 | Yes | Up | - |
| P10809 | HSPD1 | 53/114(46.49) | 0.19 | 1.15E-02 | 2.19E-02 | Yes | Up | - |
| P42695 | NCAPD3 | 57/114(50.0) | 0.22 | 6.11E-03 | 1.25E-02 | Yes | Up | - |
| Q86XN6 | ZNF761 | 50/114(43.86) | 0.17 | 1.51E-02 | 2.80E-02 | Yes | Up | - |
| Q9Y388 | RBMX2 | 63/114(55.26) | 0.17 | 1.95E-05 | 7.31E-05 | Yes | Up | - |
| Q9UI08 | EVL | 60/114(52.63) | 0.66 | 2.20E-07 | 1.34E-06 | Yes | Up | - |
| O60320 | FAM189A1 | 63/114(55.26) | 0.58 | 1.73E-03 | 4.04E-03 | Yes | Up | - |
| O43633 | CHMP2A | 66/114(57.89) | 0.31 | 1.37E-05 | 5.32E-05 | Yes | Up | - |
| P04183 | TK1 | 62/114(54.39) | 1.57 | 3.28E-17 | 6.81E-15 | Yes | Up | - |
| A6NEE1 | UPF0639 | 57/114(50.0) | 1.59 | 8.73E-11 | 1.45E-09 | Yes | Up | - |
| O14977 | AZIN1 | 63/114(55.26) | 0.17 | 2.35E-02 | 4.15E-02 | Yes | Up | - |
| Q5SXM8 | DNLZ | 68/114(59.65) | 0.38 | 2.78E-05 | 9.97E-05 | Yes | Up | - |
| Q8TBE1 | CNIH3 | 61/114(53.51) | 0.62 | 5.86E-09 | 5.56E-08 | Yes | Up | - |
| Q9UK53 | ING1 | 58/114(50.88) | 0.17 | 8.75E-03 | 1.72E-02 | Yes | Up | - |
| Q5BKX8 | MURC | 58/114(50.88) | 0.36 | 6.34E-03 | 1.29E-02 | Yes | Up | - |
| F8W7K4 | CCDC21 | 58/114(50.88) | 0.5 | 2.40E-06 | 1.12E-05 | Yes | Up | - |
| Q9Y256 | RCE1 | 63/114(55.26) | 0.34 | 9.73E-07 | 5.01E-06 | Yes | Up | - |
| Q9Y3C4 | TPRKB | 66/114(57.89) | 0.16 | 1.04E-02 | 2.02E-02 | Yes | Up | - |
| O00584 | RNASET2 | 66/114(57.89) | 0.37 | 2.01E-06 | 9.56E-06 | Yes | Up | - |
| Q6PEZ8 | PODNL1 | 68/114(59.65) | 0.67 | 6.29E-06 | 2.64E-05 | Yes | Up | - |
| P51810 | GPR143 | 58/114(50.88) | 0.8 | 2.79E-05 | 1.00E-04 | Yes | Up | - |
| O00311 | CDC7 | 64/114(56.14) | 0.89 | 6.56E-10 | 8.29E-09 | Yes | Up | - |
| Q9NYZ4 | SIGLEC8 | 57/114(50.0) | 0.4 | 2.24E-03 | 5.09E-03 | Yes | Up | - |
| Q9Y6H8 | GJA3 | 63/114(55.26) | 0.9 | 3.52E-05 | 1.24E-04 | Yes | Up | - |
| Q8N5F7 | NKAP | 61/114(53.51) | 0.17 | 5.97E-05 | 1.98E-04 | Yes | Up | - |
| P31689 | DNAJA1 | 59/114(51.75) | 0.28 | 1.12E-05 | 4.45E-05 | Yes | Up | - |
| Q9UQ10 | DHDH | 64/114(56.14) | 0.87 | 7.78E-07 | 4.11E-06 | Yes | Up | - |
| Q9HAF1 | MEAF6 | 57/114(50.0) | 0.28 | 4.44E-06 | 1.93E-05 | Yes | Up | - |
| Q9Y6G9 | DYNC1LI1 | 66/114(57.89) | 0.21 | 7.86E-04 | 1.99E-03 | Yes | Up | - |
| Q8TE59 | ADAMTS19 | 55/114(48.25) | 0.77 | 9.63E-04 | 2.39E-03 | Yes | Up | - |
| Q9NUT2 | ABCB8 | 63/114(55.26) | 0.5 | 1.29E-08 | 1.11E-07 | Yes | Up | - |
| B0I1T2 | MYO1G | 67/114(58.77) | 0.53 | 1.02E-05 | 4.08E-05 | Yes | Up | - |
| Q9Y597 | KCTD3 | 61/114(53.51) | 0.26 | 3.62E-03 | 7.79E-03 | Yes | Up | - |
| Q9UPN4 | AZI1 | 63/114(55.26) | 0.49 | 1.61E-06 | 7.80E-06 | Yes | Up | - |
| Q7Z7J9 | CAMK2N1 | 63/114(55.26) | 0.3 | 8.87E-03 | 1.74E-02 | Yes | Up | - |
| Q69YH5 | CDCA2 | 57/114(50.0) | 1.5 | 6.73E-14 | 3.45E-12 | Yes | Up | - |
| Q9H9Y2 | RPF1 | 54/114(47.37) | 0.13 | 9.24E-03 | 1.81E-02 | Yes | Up | - |
| Q9BSF4 | C19ORF52 | 60/114(52.63) | 0.31 | 1.27E-06 | 6.36E-06 | Yes | Up | - |
| O75531 | BANF1 | 62/114(54.39) | 0.38 | 4.20E-08 | 3.13E-07 | Yes | Up | - |
| Q6ZVT6 | C3ORF67 | 55/114(48.25) | 1.01 | 5.16E-10 | 6.76E-09 | Yes | Up | - |
| P14625 | HSP90B1 | 57/114(50.0) | 0.17 | 1.48E-03 | 3.51E-03 | Yes | Up | - |
| Q3LXA3 | DAK | 59/114(51.75) | 0.24 | 1.82E-03 | 4.24E-03 | Yes | Up | - |
| P83105 | HTRA4 | 59/114(51.75) | 0.58 | 2.62E-03 | 5.83E-03 | Yes | Up | - |
| Q9UBE8 | NLK | 57/114(50.0) | 0.33 | 4.30E-05 | 1.48E-04 | Yes | Up | - |
| Q9BRT2 | C6ORF125 | 67/114(58.77) | 0.47 | 2.79E-08 | 2.19E-07 | Yes | Up | - |
| O43638 | FOXS1 | 56/114(49.12) | 0.83 | 9.15E-09 | 8.22E-08 | Yes | Up | - |
| Q9H426 | RIMS4 | 63/114(55.26) | 1.74 | 3.89E-15 | 3.49E-13 | Yes | Up | - |
| P27658 | COL8A1 | 60/114(52.63) | 0.82 | 4.97E-07 | 2.77E-06 | Yes | Up | - |
| Q14653 | IRF3 | 64/114(56.14) | 0.21 | 9.37E-04 | 2.33E-03 | Yes | Up | - |
| Q96F44 | TRIM11 | 62/114(54.39) | 0.7 | 7.04E-13 | 2.42E-11 | Yes | Up | - |
| P04844 | RPN2 | 60/114(52.63) | 0.42 | 1.97E-07 | 1.22E-06 | Yes | Up | - |
| Q5JTZ9 | AARS2 | 63/114(55.26) | 0.16 | 5.95E-03 | 1.22E-02 | Yes | Up | - |
| Q01113 | IL9R | 64/114(56.14) | 1.22 | 7.54E-11 | 1.28E-09 | Yes | Up | - |
| Q8NBD8 | TMEM229B | 53/114(46.49) | 0.4 | 1.06E-03 | 2.60E-03 | Yes | Up | - |
| P35610 | SOAT1 | 53/114(46.49) | 0.27 | 3.42E-03 | 7.41E-03 | Yes | Up | - |
| P08134 | RHOC | 62/114(54.39) | 0.38 | 9.39E-08 | 6.34E-07 | Yes | Up | - |
| O15066 | KIF3B | 55/114(48.25) | 0.18 | 3.71E-03 | 7.96E-03 | Yes | Up | - |
| P10253 | GAA | 64/114(56.14) | 0.43 | 2.77E-08 | 2.18E-07 | Yes | Up | - |
| Q5JTJ3 | C1ORF31 | 62/114(54.39) | 0.59 | 1.76E-10 | 2.65E-09 | Yes | Up | - |
| P23511 | NFYA | 59/114(51.75) | 0.18 | 2.45E-03 | 5.50E-03 | Yes | Up | - |
| Q9BTE7 | DCUN1D5 | 62/114(54.39) | 0.24 | 1.46E-03 | 3.48E-03 | Yes | Up | - |
| Q9BXD5 | NPL | 52/114(45.61) | 0.27 | 9.40E-03 | 1.84E-02 | Yes | Up | - |
| Q96H40 | ZNF486 | 61/114(53.51) | 0.39 | 1.13E-02 | 2.16E-02 | Yes | Up | - |
| Q5JRK9 | PAGE2B | 33/114(28.95) | 1.98 | 1.30E-06 | 6.47E-06 | Yes | Up | - |
| Q9H0U4 | RAB1B | 58/114(50.88) | 0.22 | 2.49E-04 | 7.12E-04 | Yes | Up | - |
| O00453 | LST1 | 66/114(57.89) | 0.3 | 6.25E-03 | 1.28E-02 | Yes | Up | - |
| Q8N1L4 | CYP4Z2P | 55/114(48.25) | 0.6 | 1.77E-02 | 3.22E-02 | Yes | Up | - |
| Q92845 | KIFAP3 | 60/114(52.63) | 0.31 | 1.10E-07 | 7.29E-07 | Yes | Up | - |
| Q8N4L8 | CCDC24 | 59/114(51.75) | 0.59 | 5.52E-07 | 3.04E-06 | Yes | Up | - |
| P60002 | ELOF1 | 68/114(59.65) | 0.42 | 2.85E-11 | 5.68E-10 | Yes | Up | - |
| Q8WXD5 | GEMIN6 | 59/114(51.75) | 0.43 | 4.46E-09 | 4.39E-08 | Yes | Up | - |
| Q9BQ89 | FAM110A | 63/114(55.26) | 0.8 | 1.09E-11 | 2.48E-10 | Yes | Up | - |
| Q9UKM9 | RALY | 66/114(57.89) | 0.26 | 2.56E-05 | 9.27E-05 | Yes | Up | - |
| Q8IZT6 | ASPM | 56/114(49.12) | 1.51 | 9.53E-11 | 1.57E-09 | Yes | Up | - |
| Q8TD43 | TRPM4 | 62/114(54.39) | 0.47 | 2.38E-06 | 1.11E-05 | Yes | Up | - |
| Q9NZT2 | OGFR | 67/114(58.77) | 0.34 | 2.10E-06 | 9.96E-06 | Yes | Up | - |
| Q8NCD3 | HJURP | 58/114(50.88) | 1.6 | 1.36E-13 | 6.15E-12 | Yes | Up | - |
| O43447 | PPIH | 60/114(52.63) | 0.3 | 8.62E-06 | 3.51E-05 | Yes | Up | - |
| Q9H7S9 | ZNF703 | 57/114(50.0) | 0.68 | 6.70E-08 | 4.72E-07 | Yes | Up | - |
| Q9H6S3 | EPS8L2 | 62/114(54.39) | 0.41 | 2.86E-04 | 8.04E-04 | Yes | Up | - |
| Q14683 | SMC1A | 51/114(44.74) | 0.19 | 4.14E-03 | 8.80E-03 | Yes | Up | - |
| Q6PF15 | KLHL35 | 61/114(53.51) | 0.77 | 7.00E-07 | 3.74E-06 | Yes | Up | - |
| B4DWZ3 | C15ORF44 | 57/114(50.0) | 0.24 | 1.09E-05 | 4.34E-05 | Yes | Up | - |
| P48448 | ALDH3B2 | 58/114(50.88) | 0.52 | 2.44E-02 | 4.29E-02 | Yes | Up | - |
| O00204 | SULT2B1 | 63/114(55.26) | 0.71 | 1.66E-04 | 4.97E-04 | Yes | Up | - |
| P11166 | SLC2A1 | 59/114(51.75) | 0.87 | 3.11E-12 | 8.48E-11 | Yes | Up | - |
| Q9HCD5 | NCOA5 | 66/114(57.89) | 0.23 | 1.26E-06 | 6.28E-06 | Yes | Up | - |
| O14757 | CHEK1 | 62/114(54.39) | 0.59 | 9.94E-07 | 5.10E-06 | Yes | Up | - |
| O43525 | KCNQ3 | 61/114(53.51) | 0.3 | 2.35E-02 | 4.16E-02 | Yes | Up | - |
| O00160 | MYO1F | 62/114(54.39) | 0.24 | 1.34E-02 | 2.51E-02 | Yes | Up | - |
| Q5TC63 | GRTP1 | 61/114(53.51) | 0.55 | 3.15E-08 | 2.43E-07 | Yes | Up | - |
| - | TCAM1P | 63/114(55.26) | 0.78 | 2.62E-04 | 7.46E-04 | Yes | Up | - |
| Q96CW1 | AP2M1 | 51/114(44.74) | 0.26 | 1.38E-05 | 5.34E-05 | Yes | Up | - |
| Q9H3S7 | PTPN23 | 54/114(47.37) | 0.12 | 1.14E-02 | 2.17E-02 | Yes | Up | - |
| P56945 | BCAR1 | 72/114(63.16) | 0.17 | 2.21E-02 | 3.92E-02 | Yes | Up | - |
| P46459 | NSF | 58/114(50.88) | 0.45 | 8.81E-07 | 4.59E-06 | Yes | Up | - |
| Q96B49 | TOMM6 | 64/114(56.14) | 0.19 | 1.44E-03 | 3.42E-03 | Yes | Up | - |
| P00439 | PAH | 53/114(46.49) | 0.67 | 6.89E-03 | 1.39E-02 | Yes | Up | - |
| Q92796 | DLG3 | 62/114(54.39) | 0.6 | 1.81E-07 | 1.14E-06 | Yes | Up | - |
| Q9H2B4 | SLC26A1 | 62/114(54.39) | 0.33 | 7.90E-03 | 1.57E-02 | Yes | Up | - |
| Q9NRL2 | BAZ1A | 60/114(52.63) | 0.34 | 3.08E-06 | 1.40E-05 | Yes | Up | - |
| Q13686 | ALKBH1 | 59/114(51.75) | 0.18 | 1.45E-03 | 3.45E-03 | Yes | Up | - |
| Q9NYP3 | DONSON | 60/114(52.63) | 0.65 | 3.94E-09 | 3.93E-08 | Yes | Up | - |
| P41236 | PPP1R2 | 60/114(52.63) | 0.17 | 7.22E-03 | 1.45E-02 | Yes | Up | - |
| Q9UJH8 | METRN | 66/114(57.89) | 1.29 | 3.54E-14 | 2.09E-12 | Yes | Up | - |
| P61289 | PSME3 | 59/114(51.75) | 0.16 | 4.52E-03 | 9.53E-03 | Yes | Up | - |
| Q9NQU5 | PAK6 | 59/114(51.75) | 0.3 | 2.76E-02 | 4.79E-02 | Yes | Up | - |
| P26599 | PTBP1 | 60/114(52.63) | 0.33 | 2.56E-09 | 2.69E-08 | Yes | Up | - |
| - | LOC148413 | 59/114(51.75) | 0.31 | 5.13E-06 | 2.20E-05 | Yes | Up | - |
| O43379 | WDR62 | 60/114(52.63) | 1.43 | 1.56E-12 | 4.68E-11 | Yes | Up | - |
| Q9NZC2 | TREM2 | 61/114(53.51) | 0.58 | 8.02E-06 | 3.29E-05 | Yes | Up | - |
| Q96EZ8 | MCRS1 | 65/114(57.02) | 0.2 | 3.61E-06 | 1.60E-05 | Yes | Up | - |
| P09914 | IFIT1 | 53/114(46.49) | 0.46 | 1.81E-03 | 4.20E-03 | Yes | Up | - |
| - | RACGAP1P | 55/114(48.25) | 0.64 | 8.90E-04 | 2.23E-03 | Yes | Up | - |
| Q6NUJ5 | PWWP2B | 57/114(50.0) | 0.3 | 7.76E-05 | 2.51E-04 | Yes | Up | - |
| Q4V339 | CBWD6 | 67/114(58.77) | 0.23 | 1.70E-02 | 3.11E-02 | Yes | Up | - |
| Q96A28 | SLAMF9 | 58/114(50.88) | 0.82 | 1.72E-05 | 6.54E-05 | Yes | Up | - |
| O15228 | GNPAT | 58/114(50.88) | 0.2 | 3.72E-03 | 7.99E-03 | Yes | Up | - |
| P20591 | MX1 | 59/114(51.75) | 0.74 | 7.60E-08 | 5.28E-07 | Yes | Up | - |
| Q8NCC3 | PLA2G15 | 61/114(53.51) | 0.22 | 9.07E-04 | 2.26E-03 | Yes | Up | - |
| Q14249 | ENDOG | 63/114(55.26) | 0.32 | 2.78E-04 | 7.83E-04 | Yes | Up | - |
| O75452 | RDH16 | 61/114(53.51) | 1.12 | 3.73E-09 | 3.75E-08 | Yes | Up | - |
| Q4W5P6 | TMEM155 | 58/114(50.88) | 0.59 | 4.64E-03 | 9.75E-03 | Yes | Up | - |
| Q12849 | GRSF1 | 58/114(50.88) | 0.14 | 1.22E-02 | 2.32E-02 | Yes | Up | - |
| Q8IYR0 | C6ORF165 | 61/114(53.51) | 0.39 | 1.66E-02 | 3.05E-02 | Yes | Up | - |
| Q8IWU6 | SULF1 | 60/114(52.63) | 1.1 | 1.19E-12 | 3.74E-11 | Yes | Up | - |
| Q9UBZ4 | APEX2 | 64/114(56.14) | 0.48 | 1.12E-10 | 1.79E-09 | Yes | Up | - |
| Q9UBM7 | DHCR7 | 61/114(53.51) | 0.53 | 3.92E-05 | 1.36E-04 | Yes | Up | - |
| O43731 | KDELR3 | 60/114(52.63) | 0.6 | 1.68E-06 | 8.15E-06 | Yes | Up | - |
| Q9BYD3 | MRPL4 | 67/114(58.77) | 0.26 | 2.37E-04 | 6.79E-04 | Yes | Up | - |
| P08254 | MMP3 | 62/114(54.39) | 1.08 | 2.93E-06 | 1.34E-05 | Yes | Up | - |
| P42685 | FRK | 59/114(51.75) | 0.39 | 1.13E-03 | 2.76E-03 | Yes | Up | - |
| Q6NXR4 | C8ORF41 | 65/114(57.02) | 0.18 | 3.91E-03 | 8.35E-03 | Yes | Up | - |
| Q9NSU2 | TREX1 | 62/114(54.39) | 0.29 | 3.29E-08 | 2.53E-07 | Yes | Up | - |
| P61086 | UBE2K | 50/114(43.86) | 0.14 | 1.00E-02 | 1.94E-02 | Yes | Up | - |
| Q96AX1 | VPS33A | 62/114(54.39) | 0.2 | 1.20E-05 | 4.74E-05 | Yes | Up | - |
| Q8TB22 | SPATA20 | 58/114(50.88) | 0.28 | 2.07E-03 | 4.74E-03 | Yes | Up | - |
| Q96MH6 | TMEM68 | 59/114(51.75) | 0.22 | 2.49E-03 | 5.58E-03 | Yes | Up | - |
| Q8N1F7 | NUP93 | 63/114(55.26) | 0.22 | 5.25E-04 | 1.39E-03 | Yes | Up | - |
| Q8TD23 | ZNF675 | 55/114(48.25) | 0.48 | 4.18E-06 | 1.82E-05 | Yes | Up | - |
| Q9UKM7 | MAN1B1 | 66/114(57.89) | 0.28 | 4.35E-05 | 1.49E-04 | Yes | Up | - |
| Q17RS7 | GEN1 | 59/114(51.75) | 0.48 | 2.64E-06 | 1.22E-05 | Yes | Up | - |
| Q5XKR4 | OTP | 31/114(27.19) | 1.29 | 9.25E-04 | 2.30E-03 | Yes | Up | - |
| Q16099 | GRIK4 | 51/114(44.74) | 0.7 | 2.08E-04 | 6.06E-04 | Yes | Up | - |
| Q5T5A4 | C1ORF194 | 64/114(56.14) | 1.26 | 9.59E-08 | 6.47E-07 | Yes | Up | - |
| Q9Y4L5 | RNF115 | 55/114(48.25) | 0.25 | 2.98E-05 | 1.06E-04 | Yes | Up | - |
| P02654 | APOC1 | 66/114(57.89) | 0.71 | 3.62E-07 | 2.09E-06 | Yes | Up | - |
| - | LOC374443 | 59/114(51.75) | 0.28 | 2.62E-04 | 7.45E-04 | Yes | Up | - |
| P23769 | GATA2 | 60/114(52.63) | 0.37 | 1.16E-02 | 2.22E-02 | Yes | Up | - |
| O95881 | TXNDC12 | 62/114(54.39) | 0.12 | 7.98E-03 | 1.59E-02 | Yes | Up | - |
| - | SNHG9 | 61/114(53.51) | 0.24 | 1.92E-02 | 3.46E-02 | Yes | Up | - |
| O75431 | MTX2 | 60/114(52.63) | 0.25 | 3.91E-05 | 1.36E-04 | Yes | Up | - |
| Q9H6Q4 | NARFL | 64/114(56.14) | 0.46 | 2.34E-10 | 3.37E-09 | Yes | Up | - |
| - | LOC100131551 | 57/114(50.0) | 0.64 | 2.23E-04 | 6.45E-04 | Yes | Up | - |
| Q9BVI0 | PHF20 | 55/114(48.25) | 0.2 | 1.22E-03 | 2.95E-03 | Yes | Up | - |
| Q6UWN5 | LYPD5 | 66/114(57.89) | 0.33 | 7.36E-03 | 1.48E-02 | Yes | Up | - |
| P62314 | SNRPD1 | 60/114(52.63) | 0.44 | 1.69E-09 | 1.87E-08 | Yes | Up | - |
| Q8IWF2 | FOXRED2 | 57/114(50.0) | 0.47 | 2.26E-07 | 1.38E-06 | Yes | Up | - |
| Q9BV97 | ZNF747 | 62/114(54.39) | 0.18 | 1.73E-03 | 4.04E-03 | Yes | Up | - |
| Q86X45 | LRRC6 | 58/114(50.88) | 0.59 | 1.64E-05 | 6.26E-05 | Yes | Up | - |
| P51449 | RORC | 58/114(50.88) | 0.39 | 6.73E-03 | 1.36E-02 | Yes | Up | - |
| A6NNA2 | SRRM3 | 55/114(48.25) | 0.34 | 8.46E-03 | 1.67E-02 | Yes | Up | - |
| Q6ZRC1 | C4ORF50 | 45/114(39.47) | 1.27 | 4.44E-06 | 1.93E-05 | Yes | Up | - |
| O60241 | BAI2 | 52/114(45.61) | 0.88 | 1.82E-07 | 1.14E-06 | Yes | Up | - |
| Q14493 | SLBP | 57/114(50.0) | 0.22 | 5.40E-04 | 1.42E-03 | Yes | Up | - |
| P25789 | PSMA4 | 62/114(54.39) | 0.31 | 1.61E-05 | 6.13E-05 | Yes | Up | - |
| Q9HC84 | MUC5B | 57/114(50.0) | 1.28 | 9.13E-08 | 6.19E-07 | Yes | Up | - |
| O43818 | RRP9 | 61/114(53.51) | 0.18 | 1.19E-02 | 2.26E-02 | Yes | Up | - |
| Q4G0U5 | PCDP1 | 62/114(54.39) | 0.93 | 2.88E-05 | 1.03E-04 | Yes | Up | - |
| O43324 | EEF1E1 | 63/114(55.26) | 0.27 | 1.29E-04 | 3.97E-04 | Yes | Up | - |
| P54652 | HSPA2 | 69/114(60.53) | 0.35 | 6.63E-03 | 1.35E-02 | Yes | Up | - |
| Q13342 | SP140 | 60/114(52.63) | 0.51 | 1.08E-04 | 3.36E-04 | Yes | Up | - |
| Q9NQ76 | MEPE | 58/114(50.88) | 0.8 | 3.77E-03 | 8.08E-03 | Yes | Up | - |
| Q8IYJ1 | CPNE9 | 61/114(53.51) | 0.79 | 5.33E-05 | 1.79E-04 | Yes | Up | - |
| Q01850 | CDR2 | 67/114(58.77) | 0.46 | 8.63E-11 | 1.44E-09 | Yes | Up | - |
| Q9NNX1 | TUFT1 | 61/114(53.51) | 0.88 | 9.51E-13 | 3.09E-11 | Yes | Up | - |
| Q7Z429 | GRINA | 65/114(57.02) | 0.28 | 8.47E-04 | 2.13E-03 | Yes | Up | - |
| Q6ZP29 | PQLC2 | 61/114(53.51) | 0.38 | 2.29E-08 | 1.85E-07 | Yes | Up | - |
| Q15149 | PLEC | 60/114(52.63) | 0.23 | 1.66E-03 | 3.90E-03 | Yes | Up | - |
| Q7Z3Z2 | RD3 | 54/114(47.37) | 0.91 | 2.13E-04 | 6.18E-04 | Yes | Up | - |
| Q9HCN8 | SDF2L1 | 63/114(55.26) | 0.74 | 8.17E-12 | 1.94E-10 | Yes | Up | - |
| Q6UXX5 | ITIH5L | 55/114(48.25) | 0.81 | 2.02E-04 | 5.89E-04 | Yes | Up | - |
| Q8WV28 | BLNK | 60/114(52.63) | 0.58 | 1.50E-06 | 7.36E-06 | Yes | Up | - |
| Q9P1F3 | C6ORF115 | 63/114(55.26) | 0.56 | 9.71E-07 | 5.00E-06 | Yes | Up | - |
| P20337 | RAB3B | 55/114(48.25) | 0.81 | 1.82E-04 | 5.38E-04 | Yes | Up | - |
| Q99878 | HIST1H2AJ | 45/114(39.47) | 1.36 | 7.09E-05 | 2.31E-04 | Yes | Up | - |
| Q8N539 | FIBCD1 | 57/114(50.0) | 1.77 | 3.31E-13 | 1.28E-11 | Yes | Up | - |
| Q969G9 | NKD1 | 62/114(54.39) | 0.48 | 6.63E-04 | 1.71E-03 | Yes | Up | - |
| Q86TL0 | ATG4D | 59/114(51.75) | 0.5 | 6.75E-10 | 8.49E-09 | Yes | Up | - |
| P35998 | PSMC2 | 61/114(53.51) | 0.22 | 3.60E-04 | 9.90E-04 | Yes | Up | - |
| P61803 | DAD1 | 59/114(51.75) | 0.18 | 2.08E-03 | 4.76E-03 | Yes | Up | - |
| Q86U28 | ISCA2 | 67/114(58.77) | 0.12 | 1.83E-02 | 3.32E-02 | Yes | Up | - |
| P60006 | C11ORF51 | 57/114(50.0) | 0.17 | 5.80E-03 | 1.19E-02 | Yes | Up | - |
| - | LOC643387 | 57/114(50.0) | 0.19 | 1.05E-03 | 2.59E-03 | Yes | Up | - |
| Q6UYE1 | DLEU7 | 48/114(42.11) | 0.33 | 2.09E-02 | 3.73E-02 | Yes | Up | - |
| P08195 | SLC3A2 | 61/114(53.51) | 0.27 | 8.12E-05 | 2.61E-04 | Yes | Up | - |
| O94880 | PHF14 | 48/114(42.11) | 0.2 | 2.47E-05 | 9.01E-05 | Yes | Up | - |
| O60264 | SMARCA5 | 59/114(51.75) | 0.13 | 2.11E-02 | 3.77E-02 | Yes | Up | - |
| Q9HBH1 | PDF | 64/114(56.14) | 0.39 | 1.08E-05 | 4.31E-05 | Yes | Up | - |
| Q9NT62 | ATG3 | 60/114(52.63) | 0.1 | 2.45E-02 | 4.31E-02 | Yes | Up | - |
| Q9NPF8 | ADAP2 | 62/114(54.39) | 0.21 | 1.30E-02 | 2.44E-02 | Yes | Up | - |
| Q8NBJ5 | GLT25D1 | 55/114(48.25) | 0.43 | 4.47E-08 | 3.30E-07 | Yes | Up | - |
| Q9NXV2 | KCTD5 | 60/114(52.63) | 0.59 | 3.70E-11 | 7.02E-10 | Yes | Up | - |
| A6NDP7 | MYADML2 | 58/114(50.88) | 0.55 | 4.51E-03 | 9.51E-03 | Yes | Up | - |
| Q9P0U3 | SENP1 | 57/114(50.0) | 0.17 | 4.03E-03 | 8.60E-03 | Yes | Up | - |
| Q8N448 | LNX2 | 52/114(45.61) | 0.32 | 8.97E-05 | 2.85E-04 | Yes | Up | - |
| Q9UJG1 | MOSPD1 | 62/114(54.39) | 0.17 | 3.90E-03 | 8.33E-03 | Yes | Up | - |
| Q9BZ67 | FRMD8 | 59/114(51.75) | 0.28 | 1.02E-05 | 4.09E-05 | Yes | Up | - |
| O95620 | DUS4L | 60/114(52.63) | 0.19 | 4.59E-03 | 9.67E-03 | Yes | Up | - |
| Q15561 | TEAD4 | 69/114(60.53) | 0.53 | 4.37E-07 | 2.46E-06 | Yes | Up | - |
| Q96F83 | C14ORF79 | 59/114(51.75) | 0.36 | 7.99E-05 | 2.57E-04 | Yes | Up | - |
| P08069 | IGF1R | 55/114(48.25) | 0.41 | 1.34E-03 | 3.21E-03 | Yes | Up | - |
| O00299 | CLIC1 | 63/114(55.26) | 0.29 | 8.20E-06 | 3.36E-05 | Yes | Up | - |
| Q9BZE9 | ASPSCR1 | 66/114(57.89) | 0.52 | 1.26E-10 | 1.98E-09 | Yes | Up | - |
| O75600 | GCAT | 65/114(57.02) | 0.51 | 2.49E-06 | 1.16E-05 | Yes | Up | - |
| Q9BXK1 | KLF16 | 71/114(62.28) | 0.56 | 4.93E-09 | 4.79E-08 | Yes | Up | - |
| Q9HBF5 | ST20 | 59/114(51.75) | 0.38 | 5.34E-06 | 2.28E-05 | Yes | Up | - |
| P39656 | DDOST | 64/114(56.14) | 0.28 | 2.50E-06 | 1.16E-05 | Yes | Up | - |
| Q9NXH8 | C9ORF167 | 58/114(50.88) | 0.23 | 1.94E-02 | 3.50E-02 | Yes | Up | - |
| Q68DD2 | PLA2G4F | 62/114(54.39) | 0.74 | 4.00E-04 | 1.09E-03 | Yes | Up | - |
| Q9BPY8 | HOPX | 54/114(47.37) | 0.53 | 5.32E-05 | 1.79E-04 | Yes | Up | - |
| Q14849 | STARD3 | 65/114(57.02) | 0.23 | 1.79E-02 | 3.26E-02 | Yes | Up | - |
| Q9C0H2 | TTYH3 | 58/114(50.88) | 0.66 | 3.81E-09 | 3.81E-08 | Yes | Up | - |
| Q9BYM8 | RBCK1 | 68/114(59.65) | 0.14 | 1.27E-02 | 2.39E-02 | Yes | Up | - |
| P49366 | DHPS | 72/114(63.16) | 0.27 | 2.24E-05 | 8.23E-05 | Yes | Up | - |
| Q9H9S3 | SEC61A2 | 64/114(56.14) | 0.34 | 1.98E-04 | 5.80E-04 | Yes | Up | - |
| Q9UBB9 | TFIP11 | 61/114(53.51) | 0.13 | 2.42E-03 | 5.44E-03 | Yes | Up | - |
| Q9NZS9 | BFAR | 63/114(55.26) | 0.14 | 1.11E-03 | 2.71E-03 | Yes | Up | - |
| - | MGC4473 | 36/114(31.58) | 0.77 | 1.42E-02 | 2.65E-02 | Yes | Up | - |
| Q9H7N4 | SCAF1 | 66/114(57.89) | 0.29 | 3.47E-06 | 1.55E-05 | Yes | Up | - |
| Q9BSE5 | AGMAT | 60/114(52.63) | 0.93 | 1.67E-10 | 2.53E-09 | Yes | Up | - |
| Q12768 | KIAA0196 | 60/114(52.63) | 0.22 | 8.11E-03 | 1.61E-02 | Yes | Up | - |
| Q9NY59 | SMPD3 | 58/114(50.88) | 0.31 | 1.90E-02 | 3.43E-02 | Yes | Up | - |
| Q8NHU2 | C20ORF26 | 60/114(52.63) | 0.56 | 8.31E-04 | 2.09E-03 | Yes | Up | - |
| Q7L2J0 | MEPCE | 63/114(55.26) | 0.15 | 1.33E-03 | 3.20E-03 | Yes | Up | - |
| Q15691 | MAPRE1 | 58/114(50.88) | 0.14 | 5.39E-03 | 1.12E-02 | Yes | Up | - |
| Q8WTP8 | AEN | 57/114(50.0) | 0.22 | 8.71E-04 | 2.18E-03 | Yes | Up | - |
| P02818 | BGLAP | 62/114(54.39) | 0.29 | 1.43E-03 | 3.42E-03 | Yes | Up | - |
| Q9NW13 | RBM28 | 60/114(52.63) | 0.15 | 6.01E-03 | 1.23E-02 | Yes | Up | - |
| P41223 | BUD31 | 67/114(58.77) | 0.24 | 6.33E-06 | 2.65E-05 | Yes | Up | - |
| Q8NFV4 | ABHD11 | 61/114(53.51) | 0.73 | 1.55E-11 | 3.31E-10 | Yes | Up | - |
| Q96KJ4 | MSLNL | 37/114(32.46) | 1.46 | 8.96E-05 | 2.85E-04 | Yes | Up | - |
| Q13203 | MYBPH | 58/114(50.88) | 0.64 | 2.80E-03 | 6.20E-03 | Yes | Up | - |
| Q969R5 | L3MBTL2 | 55/114(48.25) | 0.16 | 5.50E-04 | 1.45E-03 | Yes | Up | - |
| Q96CT7 | CCDC124 | 65/114(57.02) | 0.32 | 1.79E-04 | 5.29E-04 | Yes | Up | - |
| Q5MJ68 | SPDYC | 40/114(35.09) | 1.7 | 4.83E-05 | 1.64E-04 | Yes | Up | - |
| P17036 | ZNF3 | 59/114(51.75) | 0.13 | 1.82E-02 | 3.31E-02 | Yes | Up | - |
| Q9Y320 | TMX2 | 56/114(49.12) | 0.15 | 1.90E-02 | 3.43E-02 | Yes | Up | - |
| Q96M27 | PRRC1 | 52/114(45.61) | 0.27 | 1.74E-04 | 5.17E-04 | Yes | Up | - |
| P28066 | PSMA5 | 67/114(58.77) | 0.42 | 2.90E-08 | 2.26E-07 | Yes | Up | - |
| Q96EY9 | ADAT3 | 62/114(54.39) | 0.37 | 4.19E-04 | 1.13E-03 | Yes | Up | - |
| Q8N1D0 | SLC22A18AS | 60/114(52.63) | 0.59 | 1.48E-04 | 4.47E-04 | Yes | Up | - |
| Q5JTN6 | WDR38 | 47/114(41.23) | 1.15 | 1.08E-05 | 4.28E-05 | Yes | Up | - |
| Q9Y4L1 | HYOU1 | 59/114(51.75) | 0.38 | 1.35E-05 | 5.26E-05 | Yes | Up | - |
| Q92982 | NINJ1 | 64/114(56.14) | 0.41 | 1.08E-07 | 7.14E-07 | Yes | Up | - |
| O15498 | YKT6 | 56/114(49.12) | 0.26 | 3.42E-04 | 9.43E-04 | Yes | Up | - |
| P38919 | EIF4A3 | 55/114(48.25) | 0.42 | 1.25E-07 | 8.17E-07 | Yes | Up | - |
| P85298 | ARHGAP8 | 54/114(47.37) | 0.47 | 6.57E-04 | 1.70E-03 | Yes | Up | - |
| Q5SWX8 | C1ORF27 | 52/114(45.61) | 0.31 | 9.81E-06 | 3.95E-05 | Yes | Up | - |
| Q15464 | SHB | 63/114(55.26) | 0.32 | 2.17E-04 | 6.30E-04 | Yes | Up | - |
| Q96EY1 | DNAJA3 | 56/114(49.12) | 0.39 | 4.73E-08 | 3.47E-07 | Yes | Up | - |
| O96000 | NDUFB10 | 62/114(54.39) | 0.35 | 1.11E-06 | 5.62E-06 | Yes | Up | - |
| Q12913 | PTPRJ | 55/114(48.25) | 0.23 | 1.25E-02 | 2.36E-02 | Yes | Up | - |
| Q68DV7 | RNF43 | 52/114(45.61) | 0.41 | 2.65E-02 | 4.62E-02 | Yes | Up | - |
| P39748 | FEN1 | 61/114(53.51) | 0.72 | 1.05E-10 | 1.70E-09 | Yes | Up | - |
| P05111 | INHA | 59/114(51.75) | 0.41 | 1.37E-02 | 2.57E-02 | Yes | Up | - |
| Q9HAY2 | MAGEF1 | 61/114(53.51) | 0.37 | 5.44E-07 | 3.00E-06 | Yes | Up | - |
| Q96Q77 | CIB3 | 56/114(49.12) | 2.09 | 6.59E-15 | 5.28E-13 | Yes | Up | - |
| Q9UJW9 | SERTAD3 | 67/114(58.77) | 0.28 | 7.29E-06 | 3.02E-05 | Yes | Up | - |
| Q2VYF4 | LETM2 | 58/114(50.88) | 0.49 | 1.60E-04 | 4.80E-04 | Yes | Up | - |
| Q93009 | USP7 | 65/114(57.02) | 0.21 | 3.51E-04 | 9.68E-04 | Yes | Up | - |
| Q96T88 | UHRF1 | 60/114(52.63) | 1.72 | 4.47E-15 | 3.90E-13 | Yes | Up | - |
| P61204 | ARF3 | 60/114(52.63) | 0.33 | 6.59E-07 | 3.55E-06 | Yes | Up | - |
| Q5EBM0 | CMPK2 | 56/114(49.12) | 0.57 | 1.04E-05 | 4.16E-05 | Yes | Up | - |
| Q15878 | CACNA1E | 59/114(51.75) | 0.72 | 1.68E-04 | 5.00E-04 | Yes | Up | - |
| Q9NWU1 | OXSM | 58/114(50.88) | 0.15 | 9.42E-03 | 1.84E-02 | Yes | Up | - |
| Q06547 | GABPB1 | 56/114(49.12) | 0.18 | 7.08E-04 | 1.81E-03 | Yes | Up | - |
| P26368 | U2AF2 | 62/114(54.39) | 0.35 | 2.76E-08 | 2.17E-07 | Yes | Up | - |
| Q02241 | KIF23 | 59/114(51.75) | 1.36 | 3.40E-13 | 1.31E-11 | Yes | Up | - |
| Q96QE3 | ATAD5 | 57/114(50.0) | 0.49 | 2.01E-04 | 5.88E-04 | Yes | Up | - |
| - | LOC100130776 | 64/114(56.14) | 0.31 | 4.86E-04 | 1.29E-03 | Yes | Up | - |
| Q8NBR0 | TP53I13 | 62/114(54.39) | 0.35 | 6.84E-05 | 2.24E-04 | Yes | Up | - |
| Q92874 | DNASE1L2 | 57/114(50.0) | 0.98 | 2.93E-09 | 3.04E-08 | Yes | Up | - |
| P52732 | KIF11 | 57/114(50.0) | 1.27 | 1.72E-12 | 5.12E-11 | Yes | Up | - |
| Q5QNW6 | HIST2H2BF | 58/114(50.88) | 0.66 | 1.10E-06 | 5.56E-06 | Yes | Up | - |
| P21283 | ATP6V1C1 | 58/114(50.88) | 0.16 | 1.99E-02 | 3.58E-02 | Yes | Up | - |
| Q8WZA1 | POMGNT1 | 62/114(54.39) | 0.12 | 1.72E-02 | 3.15E-02 | Yes | Up | - |
| A8MVW0 | FAM171A2 | 60/114(52.63) | 0.67 | 4.72E-06 | 2.04E-05 | Yes | Up | - |
| Q9BWD1 | ACAT2 | 56/114(49.12) | 0.33 | 1.09E-04 | 3.39E-04 | Yes | Up | - |
| A8TX70 | COL29A1 | 55/114(48.25) | 1.04 | 1.52E-03 | 3.59E-03 | Yes | Up | - |
| Q9UJA9 | ENPP5 | 59/114(51.75) | 0.72 | 6.47E-06 | 2.71E-05 | Yes | Up | - |
| Q9Y314 | NOSIP | 69/114(60.53) | 0.24 | 1.06E-03 | 2.61E-03 | Yes | Up | - |
| Q9Y6X2 | PIAS3 | 62/114(54.39) | 0.35 | 6.97E-07 | 3.73E-06 | Yes | Up | - |
| Q9UER7 | DAXX | 58/114(50.88) | 0.32 | 1.23E-08 | 1.06E-07 | Yes | Up | - |
| P11117 | ACP2 | 52/114(45.61) | 0.29 | 6.73E-05 | 2.21E-04 | Yes | Up | - |
| P12318 | FCGR2A | 64/114(56.14) | 0.31 | 2.20E-03 | 5.01E-03 | Yes | Up | - |
| Q5T4I8 | C6ORF52 | 58/114(50.88) | 0.48 | 8.08E-05 | 2.60E-04 | Yes | Up | - |
| Q5VT52 | RPRD2 | 61/114(53.51) | 0.19 | 1.32E-03 | 3.17E-03 | Yes | Up | - |
| Q9HCS7 | XAB2 | 64/114(56.14) | 0.16 | 6.49E-03 | 1.32E-02 | Yes | Up | - |
| Q99674 | CGREF1 | 54/114(47.37) | 0.32 | 1.19E-02 | 2.26E-02 | Yes | Up | - |
| P13805 | TNNT1 | 61/114(53.51) | 1.37 | 2.32E-06 | 1.09E-05 | Yes | Up | - |
| P30040 | ERP29 | 61/114(53.51) | 0.32 | 3.31E-07 | 1.93E-06 | Yes | Up | - |
| P54851 | EMP2 | 60/114(52.63) | 0.42 | 1.20E-07 | 7.83E-07 | Yes | Up | - |
| P07910 | HNRNPC | 57/114(50.0) | 0.29 | 1.01E-08 | 8.94E-08 | Yes | Up | - |
| Q96KC8 | DNAJC1 | 63/114(55.26) | 0.71 | 8.93E-11 | 1.48E-09 | Yes | Up | - |
| Q9BU76 | C1ORF35 | 70/114(61.4) | 0.53 | 6.07E-10 | 7.75E-09 | Yes | Up | - |
| Q6P1A2 | LPCAT3 | 54/114(47.37) | 0.16 | 1.98E-02 | 3.56E-02 | Yes | Up | - |
| Q9Y2G9 | SBNO2 | 64/114(56.14) | 0.35 | 1.24E-06 | 6.19E-06 | Yes | Up | - |
| Q6P7N7 | TMEM81 | 52/114(45.61) | 0.22 | 1.79E-03 | 4.16E-03 | Yes | Up | - |
| Q6ICB4 | FAM109B | 60/114(52.63) | 0.28 | 3.34E-04 | 9.24E-04 | Yes | Up | - |
| Q9Y5L2 | C7ORF68 | 60/114(52.63) | 0.33 | 5.94E-03 | 1.22E-02 | Yes | Up | - |
| P81408 | FAM189B | 62/114(54.39) | 0.47 | 1.83E-07 | 1.14E-06 | Yes | Up | - |
| Q9NZI8 | IGF2BP1 | 55/114(48.25) | 1.42 | 3.11E-09 | 3.21E-08 | Yes | Up | - |
| Q9Y6M9 | NDUFB9 | 62/114(54.39) | 0.33 | 1.46E-04 | 4.43E-04 | Yes | Up | - |
| P52597 | HNRNPF | 63/114(55.26) | 0.45 | 7.33E-11 | 1.24E-09 | Yes | Up | - |
| Q9BWE0 | REPIN1 | 61/114(53.51) | 0.18 | 2.59E-03 | 5.78E-03 | Yes | Up | - |
| P29083 | GTF2E1 | 57/114(50.0) | 0.28 | 1.03E-05 | 4.14E-05 | Yes | Up | - |
| Q9H936 | SLC25A22 | 62/114(54.39) | 0.81 | 2.02E-14 | 1.33E-12 | Yes | Up | - |
| Q8IX15 | HOMEZ | 56/114(49.12) | 0.15 | 1.04E-02 | 2.02E-02 | Yes | Up | - |
| Q5T0J3 | C1ORF220 | 53/114(46.49) | 0.54 | 1.13E-04 | 3.50E-04 | Yes | Up | - |
| Q14183 | DOC2A | 60/114(52.63) | 0.65 | 9.18E-04 | 2.29E-03 | Yes | Up | - |
| Q5T4W7 | ARTN | 63/114(55.26) | 1.36 | 6.53E-12 | 1.61E-10 | Yes | Up | - |
| Q2M3V2 | ANKRD43 | 54/114(47.37) | 1.09 | 8.45E-08 | 5.79E-07 | Yes | Up | - |
| B4E0Y6 | KIAA0146 | 56/114(49.12) | 0.2 | 5.04E-04 | 1.34E-03 | Yes | Up | - |
| Q9H2F3 | HSD3B7 | 65/114(57.02) | 0.29 | 8.25E-04 | 2.08E-03 | Yes | Up | - |
| Q96AB3 | ISOC2 | 64/114(56.14) | 0.46 | 9.65E-08 | 6.50E-07 | Yes | Up | - |
| Q7Z7K2 | ZNF467 | 59/114(51.75) | 0.79 | 6.17E-11 | 1.08E-09 | Yes | Up | - |
| O43889 | CREB3 | 60/114(52.63) | 0.21 | 2.76E-04 | 7.79E-04 | Yes | Up | - |
| O15551 | CLDN3 | 62/114(54.39) | 0.72 | 2.57E-04 | 7.31E-04 | Yes | Up | - |
| O14746 | TERT | 58/114(50.88) | 0.86 | 4.25E-04 | 1.15E-03 | Yes | Up | - |
| Q15185 | PTGES3 | 58/114(50.88) | 0.2 | 3.60E-04 | 9.88E-04 | Yes | Up | - |
| Q9HCP0 | CSNK1G1 | 54/114(47.37) | 0.21 | 3.48E-03 | 7.52E-03 | Yes | Up | - |
| Q96JJ3 | ELMO2 | 63/114(55.26) | 0.15 | 7.13E-03 | 1.43E-02 | Yes | Up | - |
| Q9HBI5 | C3ORF14 | 58/114(50.88) | 0.56 | 1.81E-04 | 5.35E-04 | Yes | Up | - |
| Q5T1C6 | THEM4 | 60/114(52.63) | 0.15 | 1.90E-02 | 3.43E-02 | Yes | Up | - |
| B4DXX1 | C1ORF25 | 52/114(45.61) | 0.15 | 1.68E-02 | 3.07E-02 | Yes | Up | - |
| Q9H9F9 | ACTR5 | 64/114(56.14) | 0.24 | 6.69E-06 | 2.79E-05 | Yes | Up | - |
| Q9UQB8 | BAIAP2 | 62/114(54.39) | 0.52 | 2.95E-09 | 3.05E-08 | Yes | Up | - |
| Q8WY22 | BRI3BP | 56/114(49.12) | 0.76 | 1.25E-10 | 1.98E-09 | Yes | Up | - |
| Q9H1C0 | LPAR5 | 65/114(57.02) | 0.37 | 3.06E-04 | 8.55E-04 | Yes | Up | - |
| P10915 | HAPLN1 | 63/114(55.26) | 1.84 | 3.24E-11 | 6.29E-10 | Yes | Up | - |
| P50613 | CDK7 | 56/114(49.12) | 0.35 | 1.65E-07 | 1.04E-06 | Yes | Up | - |
| Q15050 | RRS1 | 64/114(56.14) | 0.39 | 1.70E-05 | 6.46E-05 | Yes | Up | - |
| Q92541 | RTF1 | 57/114(50.0) | 0.13 | 7.93E-03 | 1.58E-02 | Yes | Up | - |
| Q8NCE2 | MTMR14 | 65/114(57.02) | 0.13 | 2.36E-03 | 5.33E-03 | Yes | Up | - |
| Q5JU69 | TOR2A | 64/114(56.14) | 0.61 | 8.19E-12 | 1.94E-10 | Yes | Up | - |
| Q5T9C2 | FAM102A | 64/114(56.14) | 0.75 | 2.07E-13 | 8.67E-12 | Yes | Up | - |
| A8MT69 | STRA13 | 66/114(57.89) | 0.63 | 2.09E-10 | 3.07E-09 | Yes | Up | - |
| Q9BRT6 | LLPH | 62/114(54.39) | 0.23 | 4.48E-05 | 1.53E-04 | Yes | Up | - |
| O00716 | E2F3 | 56/114(49.12) | 0.25 | 7.33E-03 | 1.47E-02 | Yes | Up | - |
| Q06323 | PSME1 | 65/114(57.02) | 0.38 | 7.12E-09 | 6.58E-08 | Yes | Up | - |
| Q701N4 | KRTAP5-2 | 53/114(46.49) | 0.83 | 5.98E-03 | 1.23E-02 | Yes | Up | - |
| Q96SN7 | ORAI2 | 65/114(57.02) | 0.45 | 5.89E-08 | 4.20E-07 | Yes | Up | - |
| P46736 | BRCC3 | 57/114(50.0) | 0.22 | 1.72E-03 | 4.03E-03 | Yes | Up | - |
| P31749 | AKT1 | 62/114(54.39) | 0.27 | 1.09E-04 | 3.39E-04 | Yes | Up | - |
| P15812 | CD1E | 56/114(49.12) | 0.4 | 1.68E-02 | 3.08E-02 | Yes | Up | - |
| P07814 | EPRS | 59/114(51.75) | 0.39 | 1.55E-05 | 5.95E-05 | Yes | Up | - |
| Q8NCH0 | CHST14 | 59/114(51.75) | 0.13 | 1.84E-02 | 3.34E-02 | Yes | Up | - |
| O75083 | WDR1 | 62/114(54.39) | 0.22 | 2.83E-06 | 1.30E-05 | Yes | Up | - |
| Q8IWE2 | FAM114A1 | 59/114(51.75) | 0.18 | 2.53E-02 | 4.44E-02 | Yes | Up | - |
| Q8NA72 | POC5 | 59/114(51.75) | 0.19 | 2.33E-03 | 5.27E-03 | Yes | Up | - |
| Q96SQ9 | CYP2S1 | 57/114(50.0) | 0.44 | 5.60E-05 | 1.87E-04 | Yes | Up | - |
| Q6P1N0 | CC2D1A | 66/114(57.89) | 0.19 | 1.35E-03 | 3.25E-03 | Yes | Up | - |
| P04424 | ASL | 66/114(57.89) | 0.29 | 1.46E-05 | 5.65E-05 | Yes | Up | - |
| Q8N8Y2 | ATP6V0D2 | 57/114(50.0) | 0.88 | 1.10E-04 | 3.42E-04 | Yes | Up | - |
| Q13009 | TIAM1 | 67/114(58.77) | 0.25 | 7.66E-03 | 1.53E-02 | Yes | Up | - |
| Q13823 | GNL2 | 60/114(52.63) | 0.18 | 4.75E-04 | 1.27E-03 | Yes | Up | - |
| Q96G75 | RMND5B | 67/114(58.77) | 0.21 | 8.20E-04 | 2.07E-03 | Yes | Up | - |
| Q9NQ30 | ESM1 | 59/114(51.75) | 1.34 | 3.59E-11 | 6.86E-10 | Yes | Up | - |
| Q9BWU0 | SLC4A1AP | 57/114(50.0) | 0.13 | 2.35E-03 | 5.30E-03 | Yes | Up | - |
| P23510 | TNFSF4 | 63/114(55.26) | 1.15 | 5.66E-12 | 1.42E-10 | Yes | Up | - |
| Q9UIB8 | CD84 | 59/114(51.75) | 0.32 | 1.48E-02 | 2.76E-02 | Yes | Up | - |
| Q01538 | MYT1 | 62/114(54.39) | 1.69 | 8.29E-12 | 1.96E-10 | Yes | Up | - |
| P06493 | CDK1 | 62/114(54.39) | 1.45 | 1.77E-13 | 7.67E-12 | Yes | Up | - |
| Q9Y6D6 | ARFGEF1 | 59/114(51.75) | 0.33 | 2.85E-04 | 8.00E-04 | Yes | Up | - |
| - | SPDYE8P | 53/114(46.49) | 0.19 | 1.11E-02 | 2.13E-02 | Yes | Up | - |
| Q9NY33 | DPP3 | 59/114(51.75) | 0.83 | 7.45E-13 | 2.53E-11 | Yes | Up | - |
| Q9H8S9 | MOBKL1B | 57/114(50.0) | 0.2 | 1.89E-03 | 4.37E-03 | Yes | Up | - |
| Q9UJC3 | HOOK1 | 59/114(51.75) | 0.46 | 1.58E-02 | 2.92E-02 | Yes | Up | - |
| Q96EK6 | GNPNAT1 | 55/114(48.25) | 0.37 | 1.28E-05 | 5.02E-05 | Yes | Up | - |
| P10153 | RNASE2 | 68/114(59.65) | 0.63 | 6.03E-05 | 2.00E-04 | Yes | Up | - |
| Q16864 | ATP6V1F | 63/114(55.26) | 0.33 | 3.93E-06 | 1.73E-05 | Yes | Up | - |
| Q9NZJ0 | DTL | 61/114(53.51) | 1.59 | 1.22E-15 | 1.30E-13 | Yes | Up | - |
| O95084 | PRSS23 | 57/114(50.0) | 0.51 | 2.82E-05 | 1.01E-04 | Yes | Up | - |
| Q9P003 | CNIH4 | 61/114(53.51) | 0.25 | 1.26E-03 | 3.05E-03 | Yes | Up | - |
| O15357 | INPPL1 | 60/114(52.63) | 0.21 | 1.03E-04 | 3.22E-04 | Yes | Up | - |
| P25098 | ADRBK1 | 65/114(57.02) | 0.27 | 1.44E-05 | 5.56E-05 | Yes | Up | - |
| Q96I13 | ABHD8 | 73/114(64.04) | 0.29 | 8.49E-05 | 2.71E-04 | Yes | Up | - |
| Q13277 | STX3 | 55/114(48.25) | 0.31 | 6.16E-05 | 2.04E-04 | Yes | Up | - |
| Q9BZE1 | MRPL37 | 55/114(48.25) | 0.17 | 1.27E-02 | 2.39E-02 | Yes | Up | - |
| P17643 | TYRP1 | 56/114(49.12) | 0.72 | 5.63E-03 | 1.16E-02 | Yes | Up | - |
| P26358 | DNMT1 | 57/114(50.0) | 0.6 | 4.43E-10 | 5.91E-09 | Yes | Up | - |
| Q8WW01 | TSEN15 | 56/114(49.12) | 0.43 | 3.47E-08 | 2.64E-07 | Yes | Up | - |
| A6NDG6 | PGP | 62/114(54.39) | 0.55 | 5.37E-09 | 5.15E-08 | Yes | Up | - |
| Q92945 | KHSRP | 65/114(57.02) | 0.17 | 8.84E-04 | 2.21E-03 | Yes | Up | - |
| Q8IYJ2 | C10ORF67 | 55/114(48.25) | 0.63 | 1.77E-02 | 3.22E-02 | Yes | Up | - |
| Q9Y5N6 | ORC6L | 59/114(51.75) | 1.19 | 2.65E-11 | 5.35E-10 | Yes | Up | - |
| Q5VVB8 | C6ORF191 | 36/114(31.58) | 1.54 | 1.24E-05 | 4.89E-05 | Yes | Up | - |
| O60664 | PLIN3 | 63/114(55.26) | 0.43 | 4.78E-09 | 4.67E-08 | Yes | Up | - |
| Q8WX77 | IGFBPL1 | 58/114(50.88) | 1.55 | 1.09E-10 | 1.75E-09 | Yes | Up | - |
| P78417 | GSTO1 | 65/114(57.02) | 0.2 | 6.27E-04 | 1.63E-03 | Yes | Up | - |
| Q8N4G2 | ARL14 | 36/114(31.58) | 0.93 | 8.45E-03 | 1.67E-02 | Yes | Up | - |
| Q8WVK7 | SKA2 | 56/114(49.12) | 0.5 | 3.62E-07 | 2.09E-06 | Yes | Up | - |
| P26842 | CD27 | 60/114(52.63) | 0.4 | 6.45E-03 | 1.31E-02 | Yes | Up | - |
| Q9HD33 | MRPL47 | 66/114(57.89) | 0.44 | 8.54E-09 | 7.73E-08 | Yes | Up | - |
| P51587 | BRCA2 | 58/114(50.88) | 0.75 | 7.77E-07 | 4.11E-06 | Yes | Up | - |
| Q5T1M5 | FKBP15 | 53/114(46.49) | 0.14 | 4.29E-04 | 1.16E-03 | Yes | Up | - |
| Q8N9P6 | C9ORF163 | 57/114(50.0) | 0.55 | 7.27E-05 | 2.36E-04 | Yes | Up | - |
| Q9HCE1 | MOV10 | 60/114(52.63) | 0.44 | 2.91E-11 | 5.76E-10 | Yes | Up | - |
| Q9H607 | OCEL1 | 67/114(58.77) | 0.28 | 4.42E-04 | 1.19E-03 | Yes | Up | - |
| Q08378 | GOLGA3 | 61/114(53.51) | 0.14 | 1.02E-03 | 2.50E-03 | Yes | Up | - |
| Q93099 | HGD | 63/114(55.26) | 0.75 | 1.73E-03 | 4.05E-03 | Yes | Up | - |
| O75306 | NDUFS2 | 61/114(53.51) | 0.46 | 3.06E-09 | 3.15E-08 | Yes | Up | - |
| Q9NWU2 | C20ORF11 | 58/114(50.88) | 0.22 | 7.53E-04 | 1.92E-03 | Yes | Up | - |
| Q9H089 | LSG1 | 57/114(50.0) | 0.29 | 9.26E-06 | 3.75E-05 | Yes | Up | - |
| Q14332 | FZD2 | 58/114(50.88) | 0.76 | 8.36E-11 | 1.40E-09 | Yes | Up | - |
| Q9UQ84 | EXO1 | 58/114(50.88) | 1.68 | 4.64E-14 | 2.60E-12 | Yes | Up | - |
| Q15532 | SS18 | 58/114(50.88) | 0.16 | 9.26E-05 | 2.94E-04 | Yes | Up | - |
| Q15078 | CDK5R1 | 62/114(54.39) | 0.56 | 1.29E-05 | 5.03E-05 | Yes | Up | - |
| Q8TAI7 | RHEBL1 | 66/114(57.89) | 0.67 | 7.12E-08 | 4.99E-07 | Yes | Up | - |
| Q8N0S6 | CENPL | 57/114(50.0) | 0.77 | 3.02E-10 | 4.21E-09 | Yes | Up | - |
| Q96N16 | JAKMIP1 | 63/114(55.26) | 1.63 | 7.04E-15 | 5.55E-13 | Yes | Up | - |
| Q08357 | SLC20A2 | 56/114(49.12) | 0.24 | 1.09E-03 | 2.66E-03 | Yes | Up | - |
| Q9Y6Z7 | COLEC10 | 57/114(50.0) | 0.85 | 3.74E-06 | 1.66E-05 | Yes | Up | - |
| O14682 | ENC1 | 63/114(55.26) | 0.49 | 1.27E-05 | 5.00E-05 | Yes | Up | - |
| Q9BR26 | C20ORF123 | 37/114(32.46) | 0.79 | 1.41E-02 | 2.63E-02 | Yes | Up | - |
| Q9Y5P6 | GMPPB | 63/114(55.26) | 0.44 | 3.88E-07 | 2.22E-06 | Yes | Up | - |
| O14618 | CCS | 62/114(54.39) | 0.25 | 1.36E-03 | 3.25E-03 | Yes | Up | - |
| O96018 | APBA3 | 71/114(62.28) | 0.27 | 1.27E-04 | 3.90E-04 | Yes | Up | - |
| Q9BW19 | KIFC1 | 61/114(53.51) | 1.49 | 9.63E-15 | 7.10E-13 | Yes | Up | - |
| Q08ET2 | SIGLEC14 | 59/114(51.75) | 0.56 | 2.20E-05 | 8.13E-05 | Yes | Up | - |
| Q5TB12 | C1ORF51 | 56/114(49.12) | 0.36 | 7.73E-03 | 1.54E-02 | Yes | Up | - |
| Q9H9L3 | ISG20L2 | 54/114(47.37) | 0.48 | 3.17E-10 | 4.39E-09 | Yes | Up | - |
| Q9HBU1 | BARX1 | 64/114(56.14) | 0.52 | 1.85E-02 | 3.35E-02 | Yes | Up | - |
| Q13323 | BIK | 59/114(51.75) | 0.9 | 4.59E-06 | 1.99E-05 | Yes | Up | - |
| P54578 | USP14 | 54/114(47.37) | 0.35 | 9.30E-05 | 2.95E-04 | Yes | Up | - |
| Q96HA4 | C1ORF159 | 66/114(57.89) | 0.39 | 1.49E-06 | 7.33E-06 | Yes | Up | - |
| Q86UX6 | STK32C | 64/114(56.14) | 0.34 | 9.42E-04 | 2.34E-03 | Yes | Up | - |
| Q12952 | FOXL1 | 57/114(50.0) | 0.36 | 3.43E-03 | 7.41E-03 | Yes | Up | - |
| Q9HB65 | ELL3 | 50/114(43.86) | 0.39 | 2.24E-04 | 6.46E-04 | Yes | Up | - |
| Q9UJV9 | DDX41 | 68/114(59.65) | 0.34 | 7.12E-08 | 4.99E-07 | Yes | Up | - |
| P11441 | UBL4A | 56/114(49.12) | 0.19 | 1.98E-03 | 4.56E-03 | Yes | Up | - |
| Q5JRA6 | MIA3 | 52/114(45.61) | 0.16 | 2.09E-02 | 3.74E-02 | Yes | Up | - |
| - | ABCA17P | 62/114(54.39) | 0.42 | 1.35E-03 | 3.23E-03 | Yes | Up | - |
| Q7Z6L0 | PRRT2 | 54/114(47.37) | 0.58 | 9.85E-06 | 3.97E-05 | Yes | Up | - |
| Q5T3I0 | GPATCH4 | 57/114(50.0) | 0.37 | 2.85E-06 | 1.30E-05 | Yes | Up | - |
| B4DZ31 | C12ORF48 | 57/114(50.0) | 0.82 | 1.90E-09 | 2.07E-08 | Yes | Up | - |
| O43293 | DAPK3 | 68/114(59.65) | 0.4 | 4.07E-08 | 3.04E-07 | Yes | Up | - |
| O76014 | KRT37 | 53/114(46.49) | 1.81 | 4.71E-10 | 6.25E-09 | Yes | Up | - |
| Q969L2 | MAL2 | 62/114(54.39) | 0.68 | 1.08E-05 | 4.28E-05 | Yes | Up | - |
| Q9NVF9 | ETNK2 | 56/114(49.12) | 0.75 | 1.39E-07 | 8.95E-07 | Yes | Up | - |
| Q9Y624 | F11R | 60/114(52.63) | 0.55 | 6.64E-11 | 1.15E-09 | Yes | Up | - |
| P36954 | POLR2I | 69/114(60.53) | 0.2 | 3.25E-03 | 7.07E-03 | Yes | Up | - |
| - | CLRN1OS | 43/114(37.72) | 1.29 | 1.80E-05 | 6.80E-05 | Yes | Up | - |
| Q9UJU2 | LEF1 | 62/114(54.39) | 1.22 | 2.89E-14 | 1.78E-12 | Yes | Up | - |
| Q00839 | HNRNPU | 60/114(52.63) | 0.3 | 7.73E-09 | 7.09E-08 | Yes | Up | - |
| O60830 | TIMM17B | 68/114(59.65) | 0.56 | 6.36E-11 | 1.11E-09 | Yes | Up | - |
| Q9BVK6 | TMED9 | 66/114(57.89) | 0.44 | 8.02E-08 | 5.54E-07 | Yes | Up | - |
| - | TUBBP5 | 52/114(45.61) | 0.53 | 6.73E-03 | 1.36E-02 | Yes | Up | - |
| P16471 | PRLR | 49/114(42.98) | 0.65 | 3.35E-04 | 9.27E-04 | Yes | Up | - |
| Q16222 | UAP1 | 59/114(51.75) | 0.22 | 7.14E-03 | 1.44E-02 | Yes | Up | - |
| P32248 | CCR7 | 61/114(53.51) | 0.62 | 1.12E-04 | 3.48E-04 | Yes | Up | - |
| Q8IXI1 | RHOT2 | 66/114(57.89) | 0.41 | 2.05E-08 | 1.69E-07 | Yes | Up | - |
| O95721 | SNAP29 | 56/114(49.12) | 0.17 | 1.06E-03 | 2.59E-03 | Yes | Up | - |
| Q12906 | ILF3 | 59/114(51.75) | 0.17 | 6.79E-04 | 1.75E-03 | Yes | Up | - |
| Q9BRQ0 | PYGO2 | 61/114(53.51) | 0.5 | 1.03E-12 | 3.31E-11 | Yes | Up | - |
| P54105 | CLNS1A | 59/114(51.75) | 0.22 | 4.87E-03 | 1.02E-02 | Yes | Up | - |
| Q9UJX6 | ANAPC2 | 69/114(60.53) | 0.15 | 7.68E-03 | 1.53E-02 | Yes | Up | - |
| Q9BVW5 | TIPIN | 63/114(55.26) | 0.32 | 7.86E-06 | 3.23E-05 | Yes | Up | - |
| Q9NXR1 | NDE1 | 61/114(53.51) | 0.52 | 7.12E-10 | 8.89E-09 | Yes | Up | - |
| P27708 | CAD | 56/114(49.12) | 0.2 | 4.70E-03 | 9.86E-03 | Yes | Up | - |
| Q4V328 | GRIPAP1 | 67/114(58.77) | 0.26 | 1.85E-05 | 6.96E-05 | Yes | Up | - |
| Q16739 | UGCG | 58/114(50.88) | 0.29 | 8.81E-03 | 1.73E-02 | Yes | Up | - |
| Q86Z20 | CCDC125 | 55/114(48.25) | 0.27 | 4.10E-03 | 8.72E-03 | Yes | Up | - |
| O96015 | DNAL4 | 63/114(55.26) | 0.32 | 4.04E-06 | 1.77E-05 | Yes | Up | - |
| Q9H8W4 | PLEKHF2 | 58/114(50.88) | 0.6 | 1.10E-07 | 7.29E-07 | Yes | Up | - |
| Q5BJD5 | TMEM41B | 52/114(45.61) | 0.3 | 1.16E-04 | 3.61E-04 | Yes | Up | - |
| O00411 | POLRMT | 63/114(55.26) | 0.2 | 1.51E-03 | 3.56E-03 | Yes | Up | - |
| P49720 | PSMB3 | 66/114(57.89) | 0.45 | 3.57E-08 | 2.72E-07 | Yes | Up | - |
| A8MT70 | ZBBX | 42/114(36.84) | 1.25 | 1.37E-05 | 5.32E-05 | Yes | Up | - |
| Q8TAF8 | LHFPL5 | 54/114(47.37) | 1 | 2.74E-04 | 7.75E-04 | Yes | Up | - |
| Q8N1D5 | C1ORF158 | 31/114(27.19) | 1.27 | 9.81E-04 | 2.43E-03 | Yes | Up | - |
| Q15126 | PMVK | 63/114(55.26) | 0.47 | 7.02E-09 | 6.51E-08 | Yes | Up | - |
| Q9UHR4 | BAIAP2L1 | 57/114(50.0) | 0.49 | 1.33E-05 | 5.19E-05 | Yes | Up | - |
| Q9UL68 | MYT1L | 40/114(35.09) | 0.75 | 1.07E-02 | 2.06E-02 | Yes | Up | - |
| Q99645 | EPYC | 60/114(52.63) | 2.52 | 2.31E-15 | 2.30E-13 | Yes | Up | - |
| P98066 | TNFAIP6 | 67/114(58.77) | 0.54 | 5.57E-05 | 1.86E-04 | Yes | Up | - |
| Q9H211 | CDT1 | 62/114(54.39) | 1.22 | 5.26E-12 | 1.33E-10 | Yes | Up | - |
| P10914 | IRF1 | 60/114(52.63) | 0.32 | 6.40E-04 | 1.66E-03 | Yes | Up | - |
| Q8N2F6 | ARMC10 | 59/114(51.75) | 0.14 | 9.15E-03 | 1.79E-02 | Yes | Up | - |
| B4DV54 | LASS5 | 72/114(63.16) | 0.19 | 3.45E-06 | 1.54E-05 | Yes | Up | - |
| P09601 | HMOX1 | 63/114(55.26) | 0.43 | 7.93E-04 | 2.01E-03 | Yes | Up | - |
| Q8NHR9 | PFN4 | 62/114(54.39) | 0.3 | 1.29E-02 | 2.43E-02 | Yes | Up | - |
| Q96MB7 | HARBI1 | 60/114(52.63) | 0.32 | 9.36E-06 | 3.78E-05 | Yes | Up | - |
| - | DLEU2 | 55/114(48.25) | 0.61 | 1.50E-06 | 7.34E-06 | Yes | Up | - |
| Q1X8D7 | LRRC36 | 65/114(57.02) | 0.54 | 1.12E-04 | 3.50E-04 | Yes | Up | - |
| Q6UXH1 | CRELD2 | 57/114(50.0) | 0.52 | 7.83E-09 | 7.16E-08 | Yes | Up | - |
| Q8N128 | FAM177A1 | 53/114(46.49) | 0.17 | 4.36E-03 | 9.24E-03 | Yes | Up | - |
| P18627 | LAG3 | 69/114(60.53) | 0.71 | 6.21E-06 | 2.61E-05 | Yes | Up | - |
| Q8NF64 | ZMIZ2 | 60/114(52.63) | 0.21 | 7.93E-04 | 2.01E-03 | Yes | Up | - |
| Q9Y534 | CSDC2 | 66/114(57.89) | 0.45 | 4.77E-03 | 1.00E-02 | Yes | Up | - |
| Q13569 | TDG | 58/114(50.88) | 0.27 | 8.22E-05 | 2.64E-04 | Yes | Up | - |
| Q6P3X8 | PGBD2 | 55/114(48.25) | 0.31 | 3.36E-05 | 1.19E-04 | Yes | Up | - |
| Q8N158 | GPC2 | 65/114(57.02) | 0.57 | 3.47E-04 | 9.57E-04 | Yes | Up | - |
| Q8WVN6 | SECTM1 | 68/114(59.65) | 0.64 | 1.06E-06 | 5.42E-06 | Yes | Up | - |
| O00398 | P2RY10 | 57/114(50.0) | 0.52 | 4.48E-04 | 1.20E-03 | Yes | Up | - |
| Q9Y4P9 | SPEF1 | 60/114(52.63) | 1.16 | 3.83E-09 | 3.83E-08 | Yes | Up | - |
| Q8TBZ6 | RG9MTD2 | 53/114(46.49) | 0.17 | 9.39E-03 | 1.83E-02 | Yes | Up | - |
| Q6WKZ4 | RAB11FIP1 | 57/114(50.0) | 0.54 | 1.31E-05 | 5.10E-05 | Yes | Up | - |
| Q5XUX1 | FBXW9 | 60/114(52.63) | 0.41 | 9.15E-06 | 3.71E-05 | Yes | Up | - |
| Q15291 | RBBP5 | 55/114(48.25) | 0.28 | 9.74E-05 | 3.07E-04 | Yes | Up | - |
| Q92771 | DDX12 | 60/114(52.63) | 0.25 | 1.40E-02 | 2.62E-02 | Yes | Up | - |
| - | PMS2L1 | 60/114(52.63) | 0.14 | 6.63E-03 | 1.34E-02 | Yes | Up | - |
| Q9NPA2 | MMP25 | 67/114(58.77) | 0.49 | 1.79E-04 | 5.31E-04 | Yes | Up | - |
| P11413 | G6PD | 59/114(51.75) | 0.34 | 1.38E-03 | 3.29E-03 | Yes | Up | - |
| Q50LG9 | LRRC24 | 66/114(57.89) | 0.42 | 1.43E-02 | 2.67E-02 | Yes | Up | - |
| P29728 | OAS2 | 61/114(53.51) | 0.88 | 2.71E-10 | 3.83E-09 | Yes | Up | - |
| Q12846 | STX4 | 67/114(58.77) | 0.27 | 3.70E-05 | 1.29E-04 | Yes | Up | - |
| P68133 | ACTA1 | 60/114(52.63) | 0.57 | 1.60E-02 | 2.94E-02 | Yes | Up | - |
| Q9UGL1 | KDM5B | 58/114(50.88) | 0.58 | 5.93E-09 | 5.61E-08 | Yes | Up | - |
| O75616 | ERAL1 | 55/114(48.25) | 0.32 | 8.23E-07 | 4.33E-06 | Yes | Up | - |
| Q9BZQ6 | EDEM3 | 48/114(42.11) | 0.32 | 1.42E-04 | 4.31E-04 | Yes | Up | - |
| O75808 | SOLH | 67/114(58.77) | 0.36 | 8.93E-07 | 4.65E-06 | Yes | Up | - |
| Q00653 | NFKB2 | 72/114(63.16) | 0.32 | 6.43E-07 | 3.47E-06 | Yes | Up | - |
| Q92828 | CORO2A | 58/114(50.88) | 0.72 | 9.37E-08 | 6.33E-07 | Yes | Up | - |
| O60499 | STX10 | 65/114(57.02) | 0.32 | 1.57E-05 | 6.02E-05 | Yes | Up | - |
| Q8N729 | NPW | 64/114(56.14) | 1.56 | 1.09E-10 | 1.74E-09 | Yes | Up | - |
| Q96T51 | RUFY1 | 58/114(50.88) | 0.09 | 1.93E-02 | 3.49E-02 | Yes | Up | - |
| Q6ZVE7 | GOLT1A | 59/114(51.75) | 0.79 | 4.22E-06 | 1.84E-05 | Yes | Up | - |
| P49257 | LMAN1 | 58/114(50.88) | 0.18 | 3.78E-03 | 8.09E-03 | Yes | Up | - |
| A6NK53 | ZNF233 | 57/114(50.0) | 0.28 | 1.49E-02 | 2.77E-02 | Yes | Up | - |
| Q9HAC7 | C7ORF10 | 63/114(55.26) | 0.43 | 5.26E-04 | 1.39E-03 | Yes | Up | - |
| Q8N8I0 | SAMD12 | 58/114(50.88) | 0.39 | 5.04E-03 | 1.05E-02 | Yes | Up | - |
| Q9BW91 | NUDT9 | 57/114(50.0) | 0.17 | 2.71E-03 | 6.02E-03 | Yes | Up | - |
| O60762 | DPM1 | 58/114(50.88) | 0.15 | 8.49E-03 | 1.68E-02 | Yes | Up | - |
| Q9BVV2 | C20ORF195 | 59/114(51.75) | 0.38 | 6.96E-03 | 1.40E-02 | Yes | Up | - |
| Q9H1M0 | NUP62CL | 54/114(47.37) | 0.51 | 9.53E-04 | 2.36E-03 | Yes | Up | - |
| Q9H269 | VPS16 | 62/114(54.39) | 0.2 | 9.98E-04 | 2.46E-03 | Yes | Up | - |
| P43686 | PSMC4 | 60/114(52.63) | 0.43 | 2.67E-07 | 1.60E-06 | Yes | Up | - |
| - | C2ORF14 | 50/114(43.86) | 1.23 | 1.55E-04 | 4.66E-04 | Yes | Up | - |
| P35789 | ZNF93 | 59/114(51.75) | 0.56 | 2.41E-06 | 1.13E-05 | Yes | Up | - |
| Q92922 | SMARCC1 | 51/114(44.74) | 0.19 | 1.56E-02 | 2.89E-02 | Yes | Up | - |
| Q8NG31 | CASC5 | 59/114(51.75) | 1.25 | 1.62E-09 | 1.81E-08 | Yes | Up | - |
| Q96SB3 | PPP1R9B | 59/114(51.75) | 0.36 | 2.09E-06 | 9.91E-06 | Yes | Up | - |
| P78325 | ADAM8 | 60/114(52.63) | 1.35 | 3.36E-16 | 4.70E-14 | Yes | Up | - |
| P21757 | MSR1 | 59/114(51.75) | 0.68 | 2.36E-07 | 1.43E-06 | Yes | Up | - |
| A7KAX9 | ARHGAP32 | 49/114(42.98) | 0.34 | 4.85E-04 | 1.29E-03 | Yes | Up | - |
| O14684 | PTGES | 61/114(53.51) | 0.36 | 4.60E-03 | 9.70E-03 | Yes | Up | - |
| P56556 | NDUFA6 | 61/114(53.51) | 0.16 | 1.76E-02 | 3.22E-02 | Yes | Up | - |
| Q9NZW4 | DSPP | 36/114(31.58) | 0.87 | 2.15E-02 | 3.84E-02 | Yes | Up | - |
| Q8N6N2 | TTC9B | 41/114(35.96) | 0.97 | 2.05E-03 | 4.71E-03 | Yes | Up | - |
| P62166 | NCS1 | 53/114(46.49) | 0.28 | 6.06E-03 | 1.24E-02 | Yes | Up | - |
| Q9UEG4 | ZNF629 | 62/114(54.39) | 0.16 | 1.25E-02 | 2.36E-02 | Yes | Up | - |
| Q00535 | CDK5 | 65/114(57.02) | 0.7 | 3.49E-12 | 9.35E-11 | Yes | Up | - |
| Q8TBA6 | GOLGA5 | 54/114(47.37) | 0.22 | 5.48E-05 | 1.84E-04 | Yes | Up | - |
| P10144 | GZMB | 61/114(53.51) | 0.44 | 2.23E-02 | 3.96E-02 | Yes | Up | - |
| P15531 | NME1 | 60/114(52.63) | 0.87 | 5.51E-13 | 1.96E-11 | Yes | Up | - |
| Q13634 | CDH18 | 40/114(35.09) | 1.2 | 1.16E-05 | 4.58E-05 | Yes | Up | - |
| P43119 | PTGIR | 58/114(50.88) | 0.45 | 1.16E-03 | 2.82E-03 | Yes | Up | - |
| O75678 | RFPL2 | 57/114(50.0) | 0.61 | 6.27E-04 | 1.63E-03 | Yes | Up | - |
| Q13724 | MOGS | 55/114(48.25) | 0.32 | 4.22E-07 | 2.39E-06 | Yes | Up | - |
| P59046 | NLRP12 | 61/114(53.51) | 0.62 | 2.86E-05 | 1.02E-04 | Yes | Up | - |
| Q8WY21 | SORCS1 | 63/114(55.26) | 1 | 2.77E-05 | 9.96E-05 | Yes | Up | - |
| Q9UNM6 | PSMD13 | 57/114(50.0) | 0.15 | 2.78E-03 | 6.16E-03 | Yes | Up | - |
| O75626 | PRDM1 | 62/114(54.39) | 0.24 | 1.09E-02 | 2.09E-02 | Yes | Up | - |
| Q96S44 | TP53RK | 61/114(53.51) | 0.26 | 3.64E-05 | 1.27E-04 | Yes | Up | - |
| Q9H0X6 | RNF208 | 63/114(55.26) | 0.4 | 9.27E-05 | 2.94E-04 | Yes | Up | - |
| Q96EV8 | DTNBP1 | 63/114(55.26) | 0.13 | 1.26E-02 | 2.38E-02 | Yes | Up | - |
| Q0VG99 | MESP2 | 58/114(50.88) | 0.52 | 2.68E-04 | 7.60E-04 | Yes | Up | - |
| P55318 | FOXA3 | 68/114(59.65) | 1.17 | 7.36E-08 | 5.14E-07 | Yes | Up | - |
| P53350 | PLK1 | 60/114(52.63) | 1.67 | 1.32E-15 | 1.40E-13 | Yes | Up | - |
| O75506 | HSBP1 | 61/114(53.51) | 0.16 | 1.12E-02 | 2.14E-02 | Yes | Up | - |
| Q8WWM7 | ATXN2L | 58/114(50.88) | 0.28 | 3.72E-07 | 2.14E-06 | Yes | Up | - |
| - | LOC285548 | 58/114(50.88) | 1.41 | 1.38E-09 | 1.58E-08 | Yes | Up | - |
| Q07687 | DLX2 | 62/114(54.39) | 0.79 | 3.09E-03 | 6.75E-03 | Yes | Up | - |
| O95259 | KCNH1 | 54/114(47.37) | 1.86 | 8.05E-22 | 8.56E-19 | Yes | Up | - |
| Q9BV10 | ALG12 | 67/114(58.77) | 0.21 | 3.82E-04 | 1.04E-03 | Yes | Up | - |
| P37235 | HPCAL1 | 60/114(52.63) | 0.21 | 5.96E-03 | 1.22E-02 | Yes | Up | - |
| Q9NYL4 | FKBP11 | 61/114(53.51) | 0.43 | 1.83E-06 | 8.78E-06 | Yes | Up | - |
| P80404 | ABAT | 60/114(52.63) | 0.75 | 9.74E-07 | 5.02E-06 | Yes | Up | - |
| Q9BRL5 | CALM3 | 56/114(49.12) | 0.16 | 3.92E-03 | 8.38E-03 | Yes | Up | - |
| Q11130 | FUT7 | 66/114(57.89) | 0.89 | 1.07E-07 | 7.13E-07 | Yes | Up | - |
| Q9UNX3 | RPL26L1 | 67/114(58.77) | 0.48 | 2.38E-08 | 1.91E-07 | Yes | Up | - |
| O95711 | LY86 | 62/114(54.39) | 0.45 | 1.45E-05 | 5.60E-05 | Yes | Up | - |
| Q5JSZ5 | BAT2 | 63/114(55.26) | 0.14 | 1.18E-02 | 2.24E-02 | Yes | Up | - |
| Q96AY2 | EME1 | 61/114(53.51) | 1.34 | 1.64E-15 | 1.66E-13 | Yes | Up | - |
| Q8N2G4 | LYPD1 | 65/114(57.02) | 0.84 | 4.49E-08 | 3.31E-07 | Yes | Up | - |
| P0C2S0 | CTXN2 | 50/114(43.86) | 1.3 | 5.58E-06 | 2.37E-05 | Yes | Up | - |
| Q9NX61 | TMEM161A | 65/114(57.02) | 0.28 | 4.28E-05 | 1.47E-04 | Yes | Up | - |
| P26885 | FKBP2 | 61/114(53.51) | 0.43 | 3.25E-05 | 1.15E-04 | Yes | Up | - |
| Q9ULL5 | PRR12 | 60/114(52.63) | 0.25 | 1.67E-05 | 6.33E-05 | Yes | Up | - |
| Q8N5I4 | DHRSX | 56/114(49.12) | 0.17 | 5.69E-03 | 1.17E-02 | Yes | Up | - |
| P08709 | F7 | 50/114(43.86) | 1.57 | 1.04E-14 | 7.49E-13 | Yes | Up | - |
| Q9H611 | PIF1 | 55/114(48.25) | 0.89 | 4.29E-09 | 4.24E-08 | Yes | Up | - |
| Q7Z4H4 | ADM2 | 61/114(53.51) | 1.23 | 1.37E-12 | 4.20E-11 | Yes | Up | - |
| Q5QP82 | DCAF10 | 54/114(47.37) | 0.56 | 3.11E-08 | 2.41E-07 | Yes | Up | - |
| P21917 | DRD4 | 58/114(50.88) | 0.81 | 3.72E-06 | 1.65E-05 | Yes | Up | - |
| Q9NRC8 | SIRT7 | 58/114(50.88) | 0.47 | 5.64E-11 | 1.00E-09 | Yes | Up | - |
| Q93084 | ATP2A3 | 67/114(58.77) | 0.53 | 8.53E-05 | 2.73E-04 | Yes | Up | - |
| P22102 | GART | 52/114(45.61) | 0.13 | 2.42E-02 | 4.26E-02 | Yes | Up | - |
| Q9NZQ8 | TRPM5 | 40/114(35.09) | 1.08 | 2.05E-04 | 5.96E-04 | Yes | Up | - |
| Q9H5Y7 | SLITRK6 | 57/114(50.0) | 0.99 | 6.94E-06 | 2.88E-05 | Yes | Up | - |
| Q96PM9 | ZNF385A | 64/114(56.14) | 0.46 | 4.23E-06 | 1.84E-05 | Yes | Up | - |
| O43395 | PRPF3 | 60/114(52.63) | 0.25 | 2.42E-04 | 6.93E-04 | Yes | Up | - |
| P55072 | VCP | 61/114(53.51) | 0.15 | 1.13E-02 | 2.17E-02 | Yes | Up | - |
| Q8TD35 | C20ORF201 | 48/114(42.11) | 1.34 | 3.37E-06 | 1.51E-05 | Yes | Up | - |
| - | LOC100125556 | 54/114(47.37) | 0.3 | 2.28E-03 | 5.17E-03 | Yes | Up | - |
| Q9BZJ4 | SLC25A39 | 63/114(55.26) | 0.51 | 3.49E-09 | 3.54E-08 | Yes | Up | - |
| Q5JZY3 | EPHA10 | 64/114(56.14) | 0.66 | 6.09E-04 | 1.59E-03 | Yes | Up | - |
| Q8IZJ1 | UNC5B | 60/114(52.63) | 1.18 | 1.18E-17 | 2.88E-15 | Yes | Up | - |
| Q8TDL5 | C20ORF114 | 62/114(54.39) | 1.05 | 1.37E-04 | 4.18E-04 | Yes | Up | - |
| Q86UL3 | AGPAT6 | 64/114(56.14) | 0.22 | 2.17E-03 | 4.95E-03 | Yes | Up | - |
| Q6PEY2 | TUBA3E | 59/114(51.75) | 1.8 | 1.12E-13 | 5.33E-12 | Yes | Up | - |
| Q9NVP2 | ASF1B | 58/114(50.88) | 1.56 | 3.08E-16 | 4.38E-14 | Yes | Up | - |
| A6NMZ2 | SNTN | 49/114(42.98) | 1.48 | 3.59E-09 | 3.63E-08 | Yes | Up | - |
| P16410 | CTLA4 | 61/114(53.51) | 0.48 | 3.11E-03 | 6.80E-03 | Yes | Up | - |
| Q6NXE6 | ARMC6 | 59/114(51.75) | 0.36 | 2.89E-07 | 1.71E-06 | Yes | Up | - |
| Q96NN9 | AIFM3 | 60/114(52.63) | 0.68 | 3.74E-07 | 2.15E-06 | Yes | Up | - |
| Q8NAT2 | TDRD5 | 51/114(44.74) | 0.53 | 2.55E-03 | 5.70E-03 | Yes | Up | - |
| Q8IV63 | VRK3 | 64/114(56.14) | 0.17 | 2.54E-04 | 7.24E-04 | Yes | Up | - |
| P51878 | CASP5 | 62/114(54.39) | 0.7 | 4.36E-04 | 1.17E-03 | Yes | Up | - |
| O75340 | PDCD6 | 63/114(55.26) | 0.31 | 1.30E-07 | 8.42E-07 | Yes | Up | - |
| Q9BVK8 | TMEM147 | 61/114(53.51) | 0.37 | 1.80E-06 | 8.63E-06 | Yes | Up | - |
| Q9Y678 | COPG | 55/114(48.25) | 0.5 | 3.18E-10 | 4.40E-09 | Yes | Up | - |
| Q9H2A9 | CHST8 | 64/114(56.14) | 1.52 | 3.13E-13 | 1.22E-11 | Yes | Up | - |
| Q96EX3 | WDR34 | 64/114(56.14) | 0.85 | 3.57E-16 | 4.93E-14 | Yes | Up | - |
| - | FLJ46111 | 63/114(55.26) | 0.7 | 5.23E-04 | 1.38E-03 | Yes | Up | - |
| Q13177 | PAK2 | 57/114(50.0) | 0.23 | 3.10E-04 | 8.66E-04 | Yes | Up | - |
| P55017 | SLC12A3 | 64/114(56.14) | 0.53 | 1.18E-02 | 2.24E-02 | Yes | Up | - |
| Q07283 | TCHH | 64/114(56.14) | 0.41 | 3.79E-03 | 8.12E-03 | Yes | Up | - |
| P20749 | BCL3 | 69/114(60.53) | 0.6 | 5.20E-09 | 5.01E-08 | Yes | Up | - |
| Q6PCB0 | VWA1 | 63/114(55.26) | 0.52 | 5.94E-07 | 3.25E-06 | Yes | Up | - |
| P29122 | PCSK6 | 61/114(53.51) | 0.45 | 2.02E-03 | 4.64E-03 | Yes | Up | - |
| Q5T8A7 | KIAA0649 | 62/114(54.39) | 0.16 | 1.39E-02 | 2.60E-02 | Yes | Up | - |
| O75489 | NDUFS3 | 53/114(46.49) | 0.13 | 2.88E-02 | 4.98E-02 | Yes | Up | - |
| Q9NRR2 | TPSG1 | 55/114(48.25) | 1.35 | 9.60E-10 | 1.15E-08 | Yes | Up | - |
| Q8IUX7 | AEBP1 | 63/114(55.26) | 0.92 | 1.97E-11 | 4.11E-10 | Yes | Up | - |
| Q4VX71 | C1ORF66 | 64/114(56.14) | 0.24 | 1.48E-04 | 4.48E-04 | Yes | Up | - |
| Q8NHG8 | ZNRF2 | 59/114(51.75) | 0.41 | 4.63E-07 | 2.60E-06 | Yes | Up | - |
| Q04912 | MST1R | 62/114(54.39) | 0.83 | 7.76E-08 | 5.37E-07 | Yes | Up | - |
| Q8IUH4 | ZDHHC13 | 65/114(57.02) | 0.42 | 4.15E-05 | 1.43E-04 | Yes | Up | - |
| Q9NWU5 | MRPL22 | 59/114(51.75) | 0.16 | 1.70E-03 | 3.97E-03 | Yes | Up | - |
| P50897 | PPT1 | 60/114(52.63) | 0.38 | 3.24E-06 | 1.46E-05 | Yes | Up | - |
| Q86YC2 | PALB2 | 61/114(53.51) | 0.24 | 1.29E-03 | 3.10E-03 | Yes | Up | - |
| O75865 | TRAPPC6A | 71/114(62.28) | 0.38 | 7.16E-07 | 3.82E-06 | Yes | Up | - |
| P19634 | SLC9A1 | 60/114(52.63) | 0.28 | 5.02E-05 | 1.69E-04 | Yes | Up | - |
| P05787 | KRT8 | 67/114(58.77) | 1.03 | 1.14E-08 | 9.94E-08 | Yes | Up | - |
| Q8N111 | CEND1 | 62/114(54.39) | 0.79 | 1.08E-05 | 4.29E-05 | Yes | Up | - |
| A5D8T8 | CLEC18A | 57/114(50.0) | 0.44 | 2.23E-03 | 5.07E-03 | Yes | Up | - |
| Q5VV63 | ATRNL1 | 50/114(43.86) | 0.51 | 1.71E-02 | 3.13E-02 | Yes | Up | - |
| Q9BY19 | MS4A8B | 54/114(47.37) | 1.63 | 5.51E-09 | 5.26E-08 | Yes | Up | - |
| Q9Y255 | PRELID1 | 67/114(58.77) | 0.3 | 9.05E-06 | 3.67E-05 | Yes | Up | - |
| Q9BRR6 | ADPGK | 53/114(46.49) | 0.17 | 4.81E-05 | 1.63E-04 | Yes | Up | - |
| Q8WWF8 | CAPSL | 53/114(46.49) | 1.58 | 2.86E-08 | 2.23E-07 | Yes | Up | - |
| Q9UQ53 | MGAT4B | 61/114(53.51) | 0.22 | 1.61E-03 | 3.80E-03 | Yes | Up | - |
| Q15814 | TBCC | 66/114(57.89) | 0.17 | 3.16E-03 | 6.89E-03 | Yes | Up | - |
| Q8TBR7 | FAM57A | 57/114(50.0) | 0.19 | 1.31E-02 | 2.47E-02 | Yes | Up | - |
| P35609 | ACTN2 | 65/114(57.02) | 0.53 | 2.38E-02 | 4.19E-02 | Yes | Up | - |
| O14771 | ZNF213 | 63/114(55.26) | 0.44 | 2.11E-10 | 3.09E-09 | Yes | Up | - |
| P13995 | MTHFD2 | 58/114(50.88) | 0.77 | 3.33E-10 | 4.59E-09 | Yes | Up | - |
| O14530 | TXNDC9 | 57/114(50.0) | 0.21 | 1.26E-03 | 3.04E-03 | Yes | Up | - |
| Q99877 | HIST1H2BN | 60/114(52.63) | 0.71 | 5.20E-06 | 2.22E-05 | Yes | Up | - |
| Q9HB71 | CACYBP | 57/114(50.0) | 0.43 | 1.97E-07 | 1.22E-06 | Yes | Up | - |
| Q6P3R8 | NEK5 | 56/114(49.12) | 0.77 | 9.39E-05 | 2.97E-04 | Yes | Up | - |
| P35858 | IGFALS | 60/114(52.63) | 1.21 | 3.79E-09 | 3.80E-08 | Yes | Up | - |
| Q9NRZ9 | HELLS | 57/114(50.0) | 0.95 | 2.13E-08 | 1.75E-07 | Yes | Up | - |
| Q96KQ7 | EHMT2 | 62/114(54.39) | 0.29 | 2.18E-06 | 1.03E-05 | Yes | Up | - |
| Q14686 | NCOA6 | 57/114(50.0) | 0.2 | 2.88E-04 | 8.08E-04 | Yes | Up | - |
| Q96H22 | CENPN | 60/114(52.63) | 0.52 | 1.28E-05 | 5.03E-05 | Yes | Up | - |
| P30307 | CDC25C | 61/114(53.51) | 1.71 | 2.29E-13 | 9.34E-12 | Yes | Up | - |
| Q7L0R7 | RNF44 | 59/114(51.75) | 0.15 | 7.46E-03 | 1.50E-02 | Yes | Up | - |
| Q8IZA0 | KIAA0319L | 58/114(50.88) | 0.21 | 6.63E-03 | 1.35E-02 | Yes | Up | - |
| - | LOC653566 | 56/114(49.12) | 0.25 | 7.80E-05 | 2.52E-04 | Yes | Up | - |
| Q9Y3D3 | MRPS16 | 63/114(55.26) | 0.2 | 2.08E-03 | 4.77E-03 | Yes | Up | - |
| P07437 | TUBB | 57/114(50.0) | 0.35 | 2.66E-06 | 1.22E-05 | Yes | Up | - |
| Q8TE96 | DQX1 | 62/114(54.39) | 1.6 | 9.20E-10 | 1.11E-08 | Yes | Up | - |
| Q8N2W9 | PIAS4 | 64/114(56.14) | 0.42 | 9.08E-11 | 1.50E-09 | Yes | Up | - |
| Q9BW83 | IFT27 | 68/114(59.65) | 0.13 | 2.37E-02 | 4.18E-02 | Yes | Up | - |
| Q7RTN6 | STRADA | 53/114(46.49) | 0.15 | 1.05E-03 | 2.58E-03 | Yes | Up | - |
| Q9BT49 | THAP7 | 69/114(60.53) | 0.23 | 4.39E-04 | 1.18E-03 | Yes | Up | - |
| Q0D2K0 | NIPAL4 | 66/114(57.89) | 0.42 | 2.21E-02 | 3.92E-02 | Yes | Up | - |
| Q9UMR7 | CLEC4A | 59/114(51.75) | 0.23 | 2.33E-02 | 4.12E-02 | Yes | Up | - |
| Q9BVC3 | DSCC1 | 55/114(48.25) | 0.56 | 1.90E-05 | 7.12E-05 | Yes | Up | - |
| P14866 | HNRNPL | 63/114(55.26) | 0.24 | 6.30E-08 | 4.47E-07 | Yes | Up | - |
| Q7Z3S9 | NOTCH2NL | 52/114(45.61) | 0.19 | 5.05E-03 | 1.05E-02 | Yes | Up | - |
| P31949 | S100A11 | 68/114(59.65) | 0.56 | 9.20E-07 | 4.77E-06 | Yes | Up | - |
| Q9H5I1 | SUV39H2 | 57/114(50.0) | 0.25 | 3.36E-03 | 7.29E-03 | Yes | Up | - |
| Q8TC84 | FANK1 | 68/114(59.65) | 0.46 | 1.29E-03 | 3.11E-03 | Yes | Up | - |
| Q8IZQ5 | C11ORF31 | 59/114(51.75) | 0.42 | 9.64E-09 | 8.61E-08 | Yes | Up | - |
| Q8TCG1 | KIAA1524 | 56/114(49.12) | 1 | 6.09E-11 | 1.07E-09 | Yes | Up | - |
| Q9NPQ8 | RIC8A | 55/114(48.25) | 0.19 | 6.74E-05 | 2.21E-04 | Yes | Up | - |
| Q01970 | PLCB3 | 64/114(56.14) | 0.38 | 1.94E-07 | 1.21E-06 | Yes | Up | - |
| Q9HAC8 | UBTD1 | 66/114(57.89) | 0.23 | 1.39E-02 | 2.60E-02 | Yes | Up | - |
| O14617 | AP3D1 | 59/114(51.75) | 0.21 | 3.03E-05 | 1.08E-04 | Yes | Up | - |
| O94953 | KDM4B | 62/114(54.39) | 0.61 | 9.41E-09 | 8.43E-08 | Yes | Up | - |
| Q9H0M5 | ZNF700 | 62/114(54.39) | 0.18 | 7.84E-03 | 1.56E-02 | Yes | Up | - |
| Q9H5N1 | RABEP2 | 68/114(59.65) | 0.62 | 2.66E-10 | 3.77E-09 | Yes | Up | - |
| Q9P1W8 | SIRPG | 58/114(50.88) | 0.64 | 2.97E-04 | 8.32E-04 | Yes | Up | - |
| O15533 | TAPBP | 67/114(58.77) | 0.41 | 1.10E-07 | 7.25E-07 | Yes | Up | - |
| Q9UJ37 | ST6GALNAC2 | 58/114(50.88) | 0.42 | 3.39E-04 | 9.36E-04 | Yes | Up | - |
| Q1ZZU3 | C9ORF119 | 66/114(57.89) | 0.33 | 1.23E-07 | 8.01E-07 | Yes | Up | - |
| Q9UHA2 | SS18L2 | 61/114(53.51) | 0.21 | 1.03E-04 | 3.24E-04 | Yes | Up | - |
| Q9Y3C1 | NOP16 | 61/114(53.51) | 0.32 | 1.72E-04 | 5.10E-04 | Yes | Up | - |
| Q2M3D2 | EXOC3L2 | 62/114(54.39) | 0.36 | 5.85E-04 | 1.53E-03 | Yes | Up | - |
| Q96HS1 | PGAM5 | 57/114(50.0) | 0.46 | 3.33E-08 | 2.56E-07 | Yes | Up | - |
| Q9BWC9 | CCDC106 | 66/114(57.89) | 0.27 | 2.08E-03 | 4.76E-03 | Yes | Up | - |
| - | LOC100130932 | 64/114(56.14) | 0.41 | 1.77E-08 | 1.47E-07 | Yes | Up | - |
| Q5TYM5 | FAM72A | 60/114(52.63) | 1.41 | 3.04E-12 | 8.36E-11 | Yes | Up | - |
| P29033 | GJB2 | 60/114(52.63) | 2.08 | 3.53E-18 | 1.05E-15 | Yes | Up | - |
| O60832 | DKC1 | 56/114(49.12) | 0.26 | 5.09E-04 | 1.35E-03 | Yes | Up | - |
| O95619 | YEATS4 | 60/114(52.63) | 0.3 | 3.42E-05 | 1.20E-04 | Yes | Up | - |
| Q9UHR6 | ZNHIT2 | 67/114(58.77) | 0.59 | 1.64E-04 | 4.90E-04 | Yes | Up | - |
| Q86UE4 | MTDH | 59/114(51.75) | 0.19 | 9.85E-03 | 1.91E-02 | Yes | Up | - |
| P56282 | POLE2 | 62/114(54.39) | 0.99 | 1.30E-11 | 2.86E-10 | Yes | Up | - |
| Q86X27 | RALGPS2 | 51/114(44.74) | 0.73 | 5.95E-08 | 4.24E-07 | Yes | Up | - |
| Q9H6J7 | C11ORF49 | 57/114(50.0) | 0.28 | 3.15E-05 | 1.12E-04 | Yes | Up | - |
| P20853 | CYP2A7 | 62/114(54.39) | 1.03 | 1.90E-04 | 5.57E-04 | Yes | Up | - |
| B3KNX9 | BAT5 | 61/114(53.51) | 0.32 | 3.64E-07 | 2.10E-06 | Yes | Up | - |
| Q6P4F2 | FDX1L | 65/114(57.02) | 0.41 | 1.20E-08 | 1.04E-07 | Yes | Up | - |
| Q24JQ0 | C18ORF45 | 56/114(49.12) | 0.52 | 2.79E-07 | 1.66E-06 | Yes | Up | - |
| Q5VWT5 | C1ORF168 | 60/114(52.63) | 0.59 | 1.22E-02 | 2.32E-02 | Yes | Up | - |
| Q8IX19 | C19ORF59 | 62/114(54.39) | 0.67 | 1.35E-03 | 3.23E-03 | Yes | Up | - |
| Q15649 | ZNHIT3 | 55/114(48.25) | 0.17 | 7.07E-03 | 1.42E-02 | Yes | Up | - |
| O15360 | FANCA | 64/114(56.14) | 1.02 | 4.22E-10 | 5.66E-09 | Yes | Up | - |
| Q5R3K3 | FAM26F | 63/114(55.26) | 0.43 | 5.09E-04 | 1.35E-03 | Yes | Up | - |
| P19397 | CD53 | 59/114(51.75) | 0.23 | 2.49E-02 | 4.36E-02 | Yes | Up | - |
| O43414 | ERI3 | 61/114(53.51) | 0.17 | 2.61E-03 | 5.81E-03 | Yes | Up | - |
| P40616 | ARL1 | 54/114(47.37) | 0.22 | 9.94E-05 | 3.13E-04 | Yes | Up | - |
| Q6P3S1 | DENND1B | 55/114(48.25) | 0.44 | 4.57E-03 | 9.62E-03 | Yes | Up | - |
| Q13239 | SLA | 57/114(50.0) | 0.32 | 2.64E-03 | 5.88E-03 | Yes | Up | - |
| Q9Y2X3 | NOP58 | 62/114(54.39) | 0.28 | 1.36E-04 | 4.14E-04 | Yes | Up | - |
| Q8N461 | FBXL16 | 60/114(52.63) | 1.16 | 2.00E-09 | 2.17E-08 | Yes | Up | - |
| O75663 | TIPRL | 56/114(49.12) | 0.31 | 2.11E-06 | 9.99E-06 | Yes | Up | - |
| Q13636 | RAB31 | 67/114(58.77) | 0.85 | 1.16E-09 | 1.36E-08 | Yes | Up | - |
| P21579 | SYT1 | 62/114(54.39) | 1.03 | 4.68E-08 | 3.44E-07 | Yes | Up | - |
| Q14839 | CHD4 | 55/114(48.25) | 0.28 | 7.94E-08 | 5.49E-07 | Yes | Up | - |
| Q10589 | BST2 | 64/114(56.14) | 0.62 | 5.06E-06 | 2.17E-05 | Yes | Up | - |
| Q9NRC9 | OTOR | 58/114(50.88) | 0.78 | 8.35E-03 | 1.65E-02 | Yes | Up | - |
| Q5VYV7 | C20ORF94 | 57/114(50.0) | 0.29 | 1.67E-02 | 3.06E-02 | Yes | Up | - |
| Q5BN46 | C9ORF116 | 65/114(57.02) | 1.06 | 8.46E-13 | 2.80E-11 | Yes | Up | - |
| Q30154 | HLA-DRB5 | 58/114(50.88) | 0.27 | 2.08E-02 | 3.73E-02 | Yes | Up | - |
| Q8NHQ9 | DDX55 | 58/114(50.88) | 0.15 | 5.34E-03 | 1.11E-02 | Yes | Up | - |
| P30304 | CDC25A | 61/114(53.51) | 0.91 | 4.87E-08 | 3.56E-07 | Yes | Up | - |
| Q96CK0 | ZNF653 | 66/114(57.89) | 0.41 | 9.91E-08 | 6.66E-07 | Yes | Up | - |
| - | LOC400940 | 43/114(37.72) | 0.78 | 1.23E-02 | 2.34E-02 | Yes | Up | - |
| Q9BTW9 | TBCD | 58/114(50.88) | 0.27 | 6.10E-06 | 2.57E-05 | Yes | Up | - |
| Q8N9F7 | GDPD1 | 55/114(48.25) | 0.42 | 1.02E-03 | 2.51E-03 | Yes | Up | - |
| Q9C0H5 | ARHGAP39 | 60/114(52.63) | 0.79 | 2.74E-09 | 2.86E-08 | Yes | Up | - |
| O15164 | TRIM24 | 59/114(51.75) | 0.25 | 7.02E-04 | 1.80E-03 | Yes | Up | - |
| B0FP48 | UPK3BL | 65/114(57.02) | 0.4 | 9.72E-03 | 1.89E-02 | Yes | Up | - |
| Q86Y91 | KIF18B | 59/114(51.75) | 1.57 | 2.20E-12 | 6.28E-11 | Yes | Up | - |
| Q99742 | NPAS1 | 64/114(56.14) | 0.71 | 6.35E-06 | 2.66E-05 | Yes | Up | - |
| B3KSP0 | WHSC2 | 62/114(54.39) | 0.18 | 1.47E-03 | 3.49E-03 | Yes | Up | - |
| Q07812 | BAX | 63/114(55.26) | 0.61 | 1.55E-14 | 1.05E-12 | Yes | Up | - |
| Q96CF2 | CHMP4C | 52/114(45.61) | 0.31 | 2.17E-02 | 3.86E-02 | Yes | Up | - |
| O00244 | ATOX1 | 67/114(58.77) | 0.43 | 2.97E-07 | 1.75E-06 | Yes | Up | - |
| Q9HBI0 | PARVG | 62/114(54.39) | 0.29 | 3.75E-03 | 8.04E-03 | Yes | Up | - |
| P82921 | MRPS21 | 64/114(56.14) | 0.25 | 1.03E-03 | 2.53E-03 | Yes | Up | - |
| Q9BQE4 | SELS | 65/114(57.02) | 0.26 | 7.37E-05 | 2.39E-04 | Yes | Up | - |
| Q9NSE4 | IARS2 | 56/114(49.12) | 0.26 | 7.64E-04 | 1.94E-03 | Yes | Up | - |
| Q8N130 | SLC34A3 | 57/114(50.0) | 0.93 | 2.62E-07 | 1.57E-06 | Yes | Up | - |
| Q969G2 | LHX4 | 53/114(46.49) | 0.47 | 2.18E-03 | 4.97E-03 | Yes | Up | - |
| - | LOC341056 | 62/114(54.39) | 0.49 | 1.78E-07 | 1.12E-06 | Yes | Up | - |
| Q8N485 | LIX1 | 58/114(50.88) | 1.34 | 1.02E-07 | 6.83E-07 | Yes | Up | - |
| P61599 | NAA20 | 58/114(50.88) | 0.29 | 8.62E-05 | 2.75E-04 | Yes | Up | - |
| Q9NQ87 | HEYL | 58/114(50.88) | 0.61 | 7.10E-07 | 3.79E-06 | Yes | Up | - |
| Q9BQI5 | SGIP1 | 60/114(52.63) | 0.45 | 2.79E-04 | 7.86E-04 | Yes | Up | - |
| O14908 | GIPC1 | 64/114(56.14) | 0.48 | 1.69E-08 | 1.42E-07 | Yes | Up | - |
| Q9ULU4 | ZMYND8 | 55/114(48.25) | 0.28 | 3.24E-04 | 8.99E-04 | Yes | Up | - |
| Q8NFH8 | REPS2 | 62/114(54.39) | 0.66 | 8.22E-06 | 3.37E-05 | Yes | Up | - |
| P20396 | TRH | 59/114(51.75) | 1.48 | 2.67E-10 | 3.77E-09 | Yes | Up | - |
| Q96FC9 | DDX11 | 56/114(49.12) | 0.36 | 1.09E-04 | 3.40E-04 | Yes | Up | - |
| Q9NX14 | NDUFB11 | 66/114(57.89) | 0.26 | 2.09E-04 | 6.08E-04 | Yes | Up | - |
| - | DGCR5 | 60/114(52.63) | 0.46 | 6.51E-03 | 1.32E-02 | Yes | Up | - |
| P61088 | UBE2N | 62/114(54.39) | 0.25 | 2.36E-05 | 8.65E-05 | Yes | Up | - |
| P39060 | COL18A1 | 63/114(55.26) | 0.25 | 9.46E-03 | 1.84E-02 | Yes | Up | - |
| P55317 | FOXA1 | 58/114(50.88) | 1.01 | 2.54E-05 | 9.23E-05 | Yes | Up | - |
| P10155 | TROVE2 | 50/114(43.86) | 0.15 | 7.56E-03 | 1.51E-02 | Yes | Up | - |
| Q86X29 | LSR | 60/114(52.63) | 0.86 | 8.91E-12 | 2.09E-10 | Yes | Up | - |
| Q8IW35 | CEP97 | 55/114(48.25) | 0.29 | 3.12E-03 | 6.81E-03 | Yes | Up | - |
| Q8N1F8 | STK11IP | 62/114(54.39) | 0.28 | 4.35E-05 | 1.49E-04 | Yes | Up | - |
| Q9P0W2 | HMG20B | 64/114(56.14) | 0.47 | 2.46E-09 | 2.59E-08 | Yes | Up | - |
| Q7LFX5 | CHST15 | 55/114(48.25) | 0.5 | 2.05E-07 | 1.26E-06 | Yes | Up | - |
| P05538 | HLA-DQB2 | 60/114(52.63) | 0.57 | 2.04E-04 | 5.94E-04 | Yes | Up | - |
| P23677 | ITPKA | 66/114(57.89) | 2.08 | 2.19E-20 | 1.14E-17 | Yes | Up | - |
| Q69YN4 | KIAA1429 | 54/114(47.37) | 0.2 | 8.63E-03 | 1.70E-02 | Yes | Up | - |
| P17861 | XBP1 | 59/114(51.75) | 0.65 | 8.72E-06 | 3.55E-05 | Yes | Up | - |
| P05141 | SLC25A5 | 60/114(52.63) | 0.21 | 4.19E-03 | 8.91E-03 | Yes | Up | - |
| Q494R4 | CCDC153 | 70/114(61.4) | 0.76 | 6.02E-07 | 3.28E-06 | Yes | Up | - |
| Q6ZV73 | FGD6 | 60/114(52.63) | 0.51 | 1.57E-07 | 1.00E-06 | Yes | Up | - |
| O76095 | JTB | 61/114(53.51) | 0.57 | 7.79E-12 | 1.87E-10 | Yes | Up | - |
| Q86UP0 | CDH24 | 65/114(57.02) | 0.33 | 7.38E-05 | 2.39E-04 | Yes | Up | - |
| P33552 | CKS2 | 62/114(54.39) | 1.23 | 6.94E-14 | 3.54E-12 | Yes | Up | - |
| Q99929 | ASCL2 | 66/114(57.89) | 0.97 | 1.84E-08 | 1.53E-07 | Yes | Up | - |
| Q9BYJ9 | YTHDF1 | 67/114(58.77) | 0.27 | 5.65E-06 | 2.40E-05 | Yes | Up | - |
| Q02224 | CENPE | 59/114(51.75) | 1.3 | 3.74E-12 | 9.97E-11 | Yes | Up | - |
| Q5PRF9 | SAMD4B | 63/114(55.26) | 0.16 | 7.60E-03 | 1.52E-02 | Yes | Up | - |
| O60870 | KIN | 56/114(49.12) | 0.09 | 2.59E-02 | 4.53E-02 | Yes | Up | - |
| Q9BQD7 | FAM173A | 69/114(60.53) | 0.78 | 1.05E-11 | 2.41E-10 | Yes | Up | - |
| P24385 | CCND1 | 52/114(45.61) | 0.56 | 5.51E-07 | 3.03E-06 | Yes | Up | - |
| P08754 | GNAI3 | 55/114(48.25) | 0.19 | 4.65E-04 | 1.25E-03 | Yes | Up | - |
| Q96KP1 | EXOC2 | 60/114(52.63) | 0.2 | 2.10E-02 | 3.76E-02 | Yes | Up | - |
| Q9BQE6 | C11ORF48 | 63/114(55.26) | 0.49 | 4.89E-10 | 6.44E-09 | Yes | Up | - |
| P26639 | TARS | 55/114(48.25) | 0.26 | 7.73E-04 | 1.96E-03 | Yes | Up | - |
| Q96KS0 | EGLN2 | 66/114(57.89) | 0.28 | 1.21E-03 | 2.93E-03 | Yes | Up | - |
| Q9NYP9 | C21ORF45 | 63/114(55.26) | 0.52 | 6.15E-08 | 4.38E-07 | Yes | Up | - |
| Q8IWF9 | CCDC83 | 50/114(43.86) | 0.99 | 3.73E-04 | 1.02E-03 | Yes | Up | - |
| O95500 | CLDN14 | 63/114(55.26) | 1.17 | 8.17E-09 | 7.44E-08 | Yes | Up | - |
| P15391 | CD19 | 62/114(54.39) | 0.89 | 1.05E-04 | 3.29E-04 | Yes | Up | - |
| Q00610 | CLTC | 57/114(50.0) | 0.43 | 6.78E-06 | 2.82E-05 | Yes | Up | - |
| O43653 | PSCA | 60/114(52.63) | 0.6 | 2.40E-03 | 5.42E-03 | Yes | Up | - |
| Q9ULW8 | PADI3 | 67/114(58.77) | 0.69 | 1.76E-03 | 4.09E-03 | Yes | Up | - |
| Q96N06 | C16ORF55 | 63/114(55.26) | 0.38 | 1.11E-07 | 7.31E-07 | Yes | Up | - |
| P29016 | CD1B | 50/114(43.86) | 0.61 | 5.81E-03 | 1.20E-02 | Yes | Up | - |
| Q9Y5Q5 | CORIN | 63/114(55.26) | 1.4 | 6.62E-13 | 2.29E-11 | Yes | Up | - |
| P49795 | RGS19 | 65/114(57.02) | 0.52 | 5.72E-10 | 7.37E-09 | Yes | Up | - |
| P49450 | CENPA | 61/114(53.51) | 1.4 | 6.72E-11 | 1.16E-09 | Yes | Up | - |
| Q5SXM2 | SNAPC4 | 71/114(62.28) | 0.18 | 9.20E-03 | 1.80E-02 | Yes | Up | - |
| Q9P0N9 | TBC1D7 | 60/114(52.63) | 0.39 | 1.59E-05 | 6.06E-05 | Yes | Up | - |
| A6NL82 | FAM183A | 58/114(50.88) | 1.04 | 4.82E-07 | 2.70E-06 | Yes | Up | - |
| Q6ZU35 | KIAA1211 | 61/114(53.51) | 1.63 | 4.90E-16 | 6.31E-14 | Yes | Up | - |
| O95780 | ZNF682 | 62/114(54.39) | 0.36 | 1.36E-04 | 4.16E-04 | Yes | Up | - |
| Q13796 | SHROOM2 | 59/114(51.75) | 0.64 | 2.52E-06 | 1.17E-05 | Yes | Up | - |
| Q969E2 | SCAMP4 | 60/114(52.63) | 0.32 | 6.00E-07 | 3.27E-06 | Yes | Up | - |
| A6NHZ5 | LRRC14B | 60/114(52.63) | 0.81 | 3.90E-04 | 1.06E-03 | Yes | Up | - |
| Q6PJW8 | CNST | 58/114(50.88) | 0.17 | 5.97E-03 | 1.23E-02 | Yes | Up | - |
| Q8N782 | ZNF525 | 52/114(45.61) | 0.31 | 4.05E-03 | 8.62E-03 | Yes | Up | - |
| Q96GQ5 | C16ORF58 | 61/114(53.51) | 0.3 | 3.40E-06 | 1.52E-05 | Yes | Up | - |
| O75716 | STK16 | 63/114(55.26) | 0.17 | 1.10E-03 | 2.69E-03 | Yes | Up | - |
| Q8TDM5 | SPACA4 | 49/114(42.98) | 0.5 | 1.13E-02 | 2.16E-02 | Yes | Up | - |
| Q9Y663 | HS3ST3A1 | 65/114(57.02) | 0.82 | 2.61E-08 | 2.06E-07 | Yes | Up | - |
| Q86Y39 | NDUFA11 | 62/114(54.39) | 0.2 | 2.85E-03 | 6.30E-03 | Yes | Up | - |
| Q9BWJ5 | SF3B5 | 54/114(47.37) | 0.14 | 1.88E-02 | 3.40E-02 | Yes | Up | - |
| Q8N1B3 | FAM58A | 64/114(56.14) | 0.19 | 3.35E-03 | 7.26E-03 | Yes | Up | - |
| P17858 | PFKL | 65/114(57.02) | 0.27 | 1.26E-04 | 3.87E-04 | Yes | Up | - |
| Q9BVX2 | TMEM106C | 63/114(55.26) | 0.35 | 1.69E-05 | 6.40E-05 | Yes | Up | - |
| Q9Y6K1 | DNMT3A | 54/114(47.37) | 0.45 | 3.18E-07 | 1.86E-06 | Yes | Up | - |
| Q8IUC6 | TICAM1 | 69/114(60.53) | 0.28 | 1.28E-06 | 6.40E-06 | Yes | Up | - |
| Q15573 | TAF1A | 55/114(48.25) | 0.16 | 2.10E-02 | 3.74E-02 | Yes | Up | - |
| P29350 | PTPN6 | 64/114(56.14) | 0.59 | 3.50E-13 | 1.34E-11 | Yes | Up | - |
| Q96R06 | SPAG5 | 57/114(50.0) | 1.45 | 2.52E-16 | 3.78E-14 | Yes | Up | - |
| Q86SU0 | ILDR1 | 57/114(50.0) | 0.68 | 1.65E-04 | 4.93E-04 | Yes | Up | - |
| Q8TE82 | SH3TC1 | 60/114(52.63) | 0.34 | 7.50E-05 | 2.43E-04 | Yes | Up | - |
| Q9BQ83 | GIYD2 | 65/114(57.02) | 0.69 | 2.21E-11 | 4.54E-10 | Yes | Up | - |
| Q8NET5 | NFAM1 | 58/114(50.88) | 0.45 | 6.69E-05 | 2.19E-04 | Yes | Up | - |
| P32302 | CXCR5 | 72/114(63.16) | 0.72 | 7.23E-05 | 2.35E-04 | Yes | Up | - |
| Q9Y265 | RUVBL1 | 63/114(55.26) | 0.49 | 6.64E-09 | 6.19E-08 | Yes | Up | - |
| P17066 | HSPA6 | 61/114(53.51) | 0.57 | 1.11E-05 | 4.40E-05 | Yes | Up | - |
| P09467 | FBP1 | 56/114(49.12) | 0.59 | 1.05E-05 | 4.21E-05 | Yes | Up | - |
| Q49MI3 | CERKL | 56/114(49.12) | 0.63 | 2.10E-07 | 1.29E-06 | Yes | Up | - |
| Q9Y4B5 | KIAA0802 | 62/114(54.39) | 0.68 | 2.40E-07 | 1.45E-06 | Yes | Up | - |
| Q8TAA5 | GRPEL2 | 55/114(48.25) | 0.18 | 3.37E-03 | 7.29E-03 | Yes | Up | - |
| O60341 | KDM1A | 55/114(48.25) | 0.21 | 1.84E-04 | 5.41E-04 | Yes | Up | - |
| Q96TA1 | FAM129B | 66/114(57.89) | 0.31 | 2.69E-05 | 9.70E-05 | Yes | Up | - |
| Q8TAE7 | KCNG3 | 73/114(64.04) | 1.08 | 2.12E-07 | 1.30E-06 | Yes | Up | - |
| Q6NUT3 | C19ORF28 | 59/114(51.75) | 0.55 | 3.03E-11 | 5.95E-10 | Yes | Up | - |
| P43250 | GRK6 | 65/114(57.02) | 0.22 | 9.56E-04 | 2.37E-03 | Yes | Up | - |
| Q5TGZ0 | C1ORF151 | 66/114(57.89) | 0.19 | 3.96E-03 | 8.46E-03 | Yes | Up | - |
| Q14061 | COX17 | 66/114(57.89) | 0.39 | 1.89E-06 | 9.04E-06 | Yes | Up | - |
| Q8N0W5 | IQCK | 63/114(55.26) | 0.25 | 7.44E-04 | 1.90E-03 | Yes | Up | - |
| Q96HF1 | SFRP2 | 59/114(51.75) | 0.81 | 8.98E-07 | 4.67E-06 | Yes | Up | - |
| P14373 | TRIM27 | 52/114(45.61) | 0.11 | 2.79E-02 | 4.83E-02 | Yes | Up | - |
| Q8N5M1 | ATPAF2 | 63/114(55.26) | 0.18 | 1.05E-03 | 2.58E-03 | Yes | Up | - |
| A1L0T0 | ILVBL | 61/114(53.51) | 0.23 | 2.96E-03 | 6.51E-03 | Yes | Up | - |
| Q9BQA9 | C17ORF62 | 61/114(53.51) | 0.46 | 2.96E-11 | 5.83E-10 | Yes | Up | - |
| Q04741 | EMX1 | 67/114(58.77) | 2.27 | 7.09E-18 | 1.83E-15 | Yes | Up | - |
| Q8N398 | VWA5B2 | 60/114(52.63) | 0.73 | 1.99E-04 | 5.83E-04 | Yes | Up | - |
| Q9NYH9 | UTP6 | 49/114(42.98) | 0.11 | 2.16E-02 | 3.86E-02 | Yes | Up | - |
| Q9BVJ7 | DUSP23 | 68/114(59.65) | 0.33 | 4.42E-05 | 1.51E-04 | Yes | Up | - |
| P49459 | UBE2A | 58/114(50.88) | 0.19 | 1.99E-03 | 4.59E-03 | Yes | Up | - |
| - | PDIA3P | 59/114(51.75) | 0.44 | 1.04E-07 | 6.94E-07 | Yes | Up | - |
| Q5JXC2 | MIIP | 62/114(54.39) | 0.29 | 1.33E-04 | 4.07E-04 | Yes | Up | - |
| P78380 | OLR1 | 63/114(55.26) | 1.3 | 2.83E-11 | 5.66E-10 | Yes | Up | - |
| Q96BR6 | ZNF669 | 52/114(45.61) | 0.16 | 2.46E-02 | 4.32E-02 | Yes | Up | - |
| O75909 | CCNK | 55/114(48.25) | 0.15 | 1.03E-02 | 1.99E-02 | Yes | Up | - |
| Q9H5S1 | C6ORF103 | 49/114(42.98) | 0.63 | 1.58E-02 | 2.92E-02 | Yes | Up | - |
| Q8TCU3 | SLC7A13 | 55/114(48.25) | 1.19 | 4.96E-08 | 3.61E-07 | Yes | Up | - |
| Q401N2 | ZACN | 40/114(35.09) | 0.68 | 1.06E-02 | 2.04E-02 | Yes | Up | - |
| Q92905 | COPS5 | 57/114(50.0) | 0.22 | 3.43E-04 | 9.46E-04 | Yes | Up | - |
| Q8IVF4 | DNAH10 | 53/114(46.49) | 0.26 | 2.22E-02 | 3.95E-02 | Yes | Up | - |
| O75417 | POLQ | 57/114(50.0) | 1.31 | 6.42E-12 | 1.59E-10 | Yes | Up | - |
| P22314 | UBA1 | 63/114(55.26) | 0.25 | 4.46E-05 | 1.53E-04 | Yes | Up | - |
| P59942 | MCCD1 | 51/114(44.74) | 0.66 | 2.84E-02 | 4.92E-02 | Yes | Up | - |
| Q9BWN1 | PRR14 | 66/114(57.89) | 0.37 | 4.55E-09 | 4.46E-08 | Yes | Up | - |
| - | HOTAIR | 63/114(55.26) | 0.78 | 2.34E-03 | 5.28E-03 | Yes | Up | - |
| Q9BU68 | PRR15L | 58/114(50.88) | 0.79 | 9.46E-05 | 2.99E-04 | Yes | Up | - |
| Q9Y4X0 | AMMECR1 | 58/114(50.88) | 0.49 | 2.52E-06 | 1.17E-05 | Yes | Up | - |
| C9JFL3 | PHGR1 | 52/114(45.61) | 0.64 | 1.16E-02 | 2.22E-02 | Yes | Up | - |
| Q05655 | PRKCD | 54/114(47.37) | 0.27 | 2.43E-03 | 5.45E-03 | Yes | Up | - |
| - | WBSCR26 | 62/114(54.39) | 0.75 | 3.73E-07 | 2.15E-06 | Yes | Up | - |
| Q9UJ96 | KCNG2 | 58/114(50.88) | 0.59 | 2.29E-03 | 5.18E-03 | Yes | Up | - |
| Q5VV67 | PPRC1 | 62/114(54.39) | 0.18 | 4.69E-03 | 9.86E-03 | Yes | Up | - |
| Q12933 | TRAF2 | 62/114(54.39) | 0.64 | 2.69E-13 | 1.08E-11 | Yes | Up | - |
| Q7Z2Z1 | C15ORF42 | 58/114(50.88) | 0.94 | 3.99E-09 | 3.97E-08 | Yes | Up | - |
| Q9HBL8 | NMRAL1 | 69/114(60.53) | 0.66 | 1.41E-13 | 6.36E-12 | Yes | Up | - |
| Q8TB61 | SLC35B2 | 50/114(43.86) | 0.28 | 5.25E-06 | 2.24E-05 | Yes | Up | - |
| Q9H147 | DNTTIP1 | 65/114(57.02) | 0.31 | 1.08E-06 | 5.49E-06 | Yes | Up | - |
| O43390 | HNRNPR | 54/114(47.37) | 0.17 | 3.34E-04 | 9.23E-04 | Yes | Up | - |
| Q7Z3T1 | OR2W3 | 57/114(50.0) | 0.39 | 2.43E-02 | 4.28E-02 | Yes | Up | - |
| Q03936 | ZNF92 | 58/114(50.88) | 0.49 | 7.54E-05 | 2.44E-04 | Yes | Up | - |
| Q96QC0 | PPP1R10 | 65/114(57.02) | 0.24 | 7.94E-06 | 3.26E-05 | Yes | Up | - |
| Q13472 | TOP3A | 56/114(49.12) | 0.13 | 2.75E-02 | 4.77E-02 | Yes | Up | - |
| Q16566 | CAMK4 | 56/114(49.12) | 0.61 | 1.47E-05 | 5.68E-05 | Yes | Up | - |
| Q6N063 | OGFOD2 | 71/114(62.28) | 0.33 | 5.17E-07 | 2.87E-06 | Yes | Up | - |
| Q9Y6X9 | MORC2 | 56/114(49.12) | 0.25 | 3.39E-05 | 1.19E-04 | Yes | Up | - |
| A8MPS7 | YDJC | 63/114(55.26) | 0.67 | 5.85E-09 | 5.56E-08 | Yes | Up | - |
| O60667 | FAIM3 | 60/114(52.63) | 0.63 | 7.74E-07 | 4.10E-06 | Yes | Up | - |
| Q7Z5H3 | ARHGAP22 | 64/114(56.14) | 0.29 | 9.17E-03 | 1.80E-02 | Yes | Up | - |
| Q8NBP0 | TTC13 | 55/114(48.25) | 0.32 | 2.45E-05 | 8.92E-05 | Yes | Up | - |
| P14550 | AKR1A1 | 64/114(56.14) | 0.29 | 5.85E-05 | 1.94E-04 | Yes | Up | - |
| Q14807 | KIF22 | 65/114(57.02) | 0.75 | 1.33E-13 | 6.07E-12 | Yes | Up | - |
| Q12788 | TBL3 | 68/114(59.65) | 0.39 | 7.92E-09 | 7.23E-08 | Yes | Up | - |
| O75817 | POP7 | 67/114(58.77) | 0.42 | 7.78E-09 | 7.13E-08 | Yes | Up | - |
| O94855 | SEC24D | 59/114(51.75) | 0.54 | 3.30E-08 | 2.54E-07 | Yes | Up | - |
| O60309 | LRRC37A3 | 55/114(48.25) | 0.37 | 2.10E-05 | 7.78E-05 | Yes | Up | - |
| Q8WU43 | C2ORF15 | 53/114(46.49) | 0.41 | 1.64E-03 | 3.85E-03 | Yes | Up | - |
| O00238 | BMPR1B | 55/114(48.25) | 1.75 | 3.90E-13 | 1.47E-11 | Yes | Up | - |
| - | LOC92659 | 55/114(48.25) | 0.94 | 5.80E-13 | 2.05E-11 | Yes | Up | - |
| Q6AI08 | HEATR6 | 55/114(48.25) | 0.34 | 1.64E-05 | 6.26E-05 | Yes | Up | - |
| Q9UHJ9 | PGAP2 | 61/114(53.51) | 0.24 | 1.41E-03 | 3.36E-03 | Yes | Up | - |
| P35670 | ATP7B | 57/114(50.0) | 0.48 | 1.48E-04 | 4.48E-04 | Yes | Up | - |
| O95433 | AHSA1 | 63/114(55.26) | 0.24 | 4.00E-05 | 1.39E-04 | Yes | Up | - |
| P98175 | RBM10 | 65/114(57.02) | 0.23 | 3.15E-06 | 1.42E-05 | Yes | Up | - |
| Q8N6F8 | WBSCR27 | 68/114(59.65) | 0.4 | 3.52E-04 | 9.69E-04 | Yes | Up | - |
| Q86WB7 | UNC93A | 27/114(23.68) | 2.03 | 1.32E-06 | 6.57E-06 | Yes | Up | - |
| Q13200 | PSMD2 | 55/114(48.25) | 0.32 | 3.34E-05 | 1.18E-04 | Yes | Up | - |
| Q15833 | STXBP2 | 60/114(52.63) | 0.89 | 5.37E-12 | 1.35E-10 | Yes | Up | - |
| Q15813 | TBCE | 57/114(50.0) | 0.45 | 2.06E-07 | 1.27E-06 | Yes | Up | - |
| Q92734 | TFG | 60/114(52.63) | 0.32 | 5.19E-06 | 2.22E-05 | Yes | Up | - |
| Q9UJJ7 | RPUSD1 | 62/114(54.39) | 0.64 | 1.23E-12 | 3.85E-11 | Yes | Up | - |
| Q9BR61 | ACBD6 | 63/114(55.26) | 0.42 | 1.48E-10 | 2.29E-09 | Yes | Up | - |
| A1L020 | MEX3A | 53/114(46.49) | 0.82 | 2.20E-06 | 1.04E-05 | Yes | Up | - |
| Q9NPF7 | IL23A | 61/114(53.51) | 0.34 | 3.28E-04 | 9.09E-04 | Yes | Up | - |
| Q92696 | RABGGTA | 59/114(51.75) | 0.14 | 3.46E-03 | 7.47E-03 | Yes | Up | - |
| Q8N8S7 | ENAH | 61/114(53.51) | 0.41 | 9.44E-06 | 3.81E-05 | Yes | Up | - |
| Q8IW40 | CCDC103 | 63/114(55.26) | 0.28 | 1.18E-02 | 2.24E-02 | Yes | Up | - |
| Q53H54 | TRMT12 | 63/114(55.26) | 0.29 | 8.08E-05 | 2.60E-04 | Yes | Up | - |
| Q7Z6M1 | RABEPK | 60/114(52.63) | 0.26 | 5.23E-05 | 1.76E-04 | Yes | Up | - |
| Q9BQB6 | VKORC1 | 68/114(59.65) | 0.26 | 3.72E-04 | 1.02E-03 | Yes | Up | - |
| Q9Y227 | ENTPD4 | 60/114(52.63) | 0.22 | 1.60E-03 | 3.77E-03 | Yes | Up | - |
| Q12756 | KIF1A | 64/114(56.14) | 0.88 | 4.91E-04 | 1.31E-03 | Yes | Up | - |
| P61970 | NUTF2 | 64/114(56.14) | 0.21 | 2.00E-03 | 4.60E-03 | Yes | Up | - |
| Q96FT9 | C14ORF179 | 62/114(54.39) | 0.22 | 3.33E-04 | 9.22E-04 | Yes | Up | - |
| Q6ZWT7 | MBOAT2 | 54/114(47.37) | 0.97 | 7.00E-11 | 1.20E-09 | Yes | Up | - |
| Q8IXZ2 | ZC3H3 | 73/114(64.04) | 0.45 | 2.79E-07 | 1.66E-06 | Yes | Up | - |
| Q7RTX0 | TAS1R3 | 55/114(48.25) | 0.78 | 4.81E-06 | 2.08E-05 | Yes | Up | - |
| Q3KP66 | C1ORF106 | 53/114(46.49) | 0.47 | 2.37E-02 | 4.18E-02 | Yes | Up | - |
| Q96EU6 | C6ORF153 | 61/114(53.51) | 0.27 | 7.33E-05 | 2.38E-04 | Yes | Up | - |
| Q96JY0 | MAEL | 62/114(54.39) | 1.27 | 2.91E-10 | 4.08E-09 | Yes | Up | - |
| P01920 | HLA-DQB1 | 61/114(53.51) | 0.45 | 4.55E-05 | 1.55E-04 | Yes | Up | - |
| Q96BI3 | APH1A | 62/114(54.39) | 0.48 | 6.18E-11 | 1.08E-09 | Yes | Up | - |
| P31944 | CASP14 | 46/114(40.35) | 1.81 | 3.95E-06 | 1.74E-05 | Yes | Up | - |
| Q8NHL6 | LILRB1 | 63/114(55.26) | 0.43 | 4.74E-05 | 1.61E-04 | Yes | Up | - |
| - | GBAP1 | 59/114(51.75) | 0.65 | 1.12E-13 | 5.33E-12 | Yes | Up | - |
| Q15233 | NONO | 56/114(49.12) | 0.17 | 1.15E-03 | 2.79E-03 | Yes | Up | - |
| Q7Z4V5 | HDGFRP2 | 74/114(64.91) | 0.36 | 7.40E-06 | 3.06E-05 | Yes | Up | - |
| - | CXCR2P1 | 60/114(52.63) | 1.34 | 2.38E-09 | 2.51E-08 | Yes | Up | - |
| Q9H490 | PIGU | 59/114(51.75) | 0.44 | 4.75E-08 | 3.48E-07 | Yes | Up | - |
| Q9BXN1 | ASPN | 64/114(56.14) | 1.2 | 2.07E-10 | 3.04E-09 | Yes | Up | - |
| Q01518 | CAP1 | 57/114(50.0) | 0.17 | 3.38E-03 | 7.31E-03 | Yes | Up | - |
| P20962 | PTMS | 60/114(52.63) | 0.35 | 1.60E-04 | 4.80E-04 | Yes | Up | - |
| Q96EP0 | RNF31 | 65/114(57.02) | 0.13 | 8.22E-03 | 1.63E-02 | Yes | Up | - |
| Q13433 | SLC39A6 | 57/114(50.0) | 0.95 | 3.22E-09 | 3.30E-08 | Yes | Up | - |
| O95803 | NDST3 | 57/114(50.0) | 0.53 | 2.16E-03 | 4.93E-03 | Yes | Up | - |
| Q14680 | MELK | 58/114(50.88) | 1.63 | 8.22E-13 | 2.75E-11 | Yes | Up | - |
| O14879 | IFIT3 | 58/114(50.88) | 0.34 | 1.06E-02 | 2.04E-02 | Yes | Up | - |
| Q9BW11 | MXD3 | 64/114(56.14) | 0.52 | 7.75E-08 | 5.37E-07 | Yes | Up | - |
| Q2I0M4 | LRRC26 | 60/114(52.63) | 0.72 | 1.19E-03 | 2.89E-03 | Yes | Up | - |
| - | SLC6A10P | 55/114(48.25) | 0.49 | 1.59E-03 | 3.74E-03 | Yes | Up | - |
| P09543 | CNP | 66/114(57.89) | 0.18 | 1.77E-03 | 4.13E-03 | Yes | Up | - |
| Q8TAG9 | EXOC6 | 60/114(52.63) | 0.32 | 1.92E-04 | 5.63E-04 | Yes | Up | - |
| Q6UWE3 | C6ORF126 | 59/114(51.75) | 1.26 | 6.09E-06 | 2.56E-05 | Yes | Up | - |
| - | AURKAPS1 | 51/114(44.74) | 0.42 | 2.87E-04 | 8.07E-04 | Yes | Up | - |
| Q495A1 | TIGIT | 64/114(56.14) | 1 | 1.04E-08 | 9.22E-08 | Yes | Up | - |
| Q9Y4R8 | TELO2 | 64/114(56.14) | 0.4 | 1.17E-06 | 5.89E-06 | Yes | Up | - |
| O43704 | SULT1B1 | 57/114(50.0) | 0.54 | 1.68E-02 | 3.07E-02 | Yes | Up | - |
| P22392 | NME2 | 57/114(50.0) | 0.3 | 6.74E-05 | 2.21E-04 | Yes | Up | - |
| O43852 | CALU | 61/114(53.51) | 0.34 | 9.46E-05 | 2.99E-04 | Yes | Up | - |
| A6NFE3 | EFCAB10 | 49/114(42.98) | 0.42 | 3.66E-03 | 7.87E-03 | Yes | Up | - |
| Q16822 | PCK2 | 53/114(46.49) | 0.43 | 5.44E-05 | 1.82E-04 | Yes | Up | - |
| P58876 | HIST1H2BD | 61/114(53.51) | 1.58 | 9.64E-19 | 3.45E-16 | Yes | Up | - |
| Q9BT09 | CNPY3 | 61/114(53.51) | 0.39 | 5.04E-09 | 4.88E-08 | Yes | Up | - |
| Q9H7V2 | TMEM90B | 63/114(55.26) | 1.76 | 3.97E-17 | 7.96E-15 | Yes | Up | - |
| Q14008 | CKAP5 | 56/114(49.12) | 0.29 | 5.47E-04 | 1.44E-03 | Yes | Up | - |
| Q9P2M7 | CGN | 52/114(45.61) | 0.54 | 4.69E-03 | 9.85E-03 | Yes | Up | - |
| O15232 | MATN3 | 59/114(51.75) | 1.73 | 6.14E-20 | 2.89E-17 | Yes | Up | - |
| Q13939 | CCIN | 64/114(56.14) | 0.53 | 5.58E-05 | 1.86E-04 | Yes | Up | - |
| O95243 | MBD4 | 57/114(50.0) | 0.22 | 1.31E-06 | 6.51E-06 | Yes | Up | - |
| Q9NS87 | KIF15 | 60/114(52.63) | 1.32 | 2.88E-11 | 5.73E-10 | Yes | Up | - |
| P55061 | TMBIM6 | 53/114(46.49) | 0.18 | 1.41E-03 | 3.37E-03 | Yes | Up | - |
| Q6TDP4 | KLHL17 | 59/114(51.75) | 0.43 | 4.59E-06 | 1.99E-05 | Yes | Up | - |
| Q92854 | SEMA4D | 63/114(55.26) | 0.23 | 3.92E-03 | 8.38E-03 | Yes | Up | - |
| Q15031 | LARS2 | 52/114(45.61) | 0.14 | 1.49E-02 | 2.76E-02 | Yes | Up | - |
| Q9Y421 | FAM32A | 60/114(52.63) | 0.15 | 2.68E-04 | 7.61E-04 | Yes | Up | - |
| Q9NYF0 | DACT1 | 61/114(53.51) | 0.42 | 5.45E-04 | 1.44E-03 | Yes | Up | - |
| P02452 | COL1A1 | 63/114(55.26) | 1.5 | 5.83E-15 | 4.85E-13 | Yes | Up | - |
| Q86YV9 | HPS6 | 62/114(54.39) | 0.15 | 1.79E-03 | 4.16E-03 | Yes | Up | - |
| P02751 | FN1 | 61/114(53.51) | 1.72 | 2.20E-17 | 4.95E-15 | Yes | Up | - |
| Q9UHC3 | ACCN3 | 62/114(54.39) | 0.56 | 1.89E-04 | 5.57E-04 | Yes | Up | - |
| Q99435 | NELL2 | 64/114(56.14) | 1.52 | 1.17E-12 | 3.69E-11 | Yes | Up | - |
| P56937 | HSD17B7 | 57/114(50.0) | 0.69 | 1.38E-10 | 2.15E-09 | Yes | Up | - |
| Q8IZ63 | PRR22 | 57/114(50.0) | 0.43 | 2.00E-04 | 5.85E-04 | Yes | Up | - |
| Q12809 | KCNH2 | 62/114(54.39) | 0.38 | 2.56E-02 | 4.48E-02 | Yes | Up | - |
| Q9Y2B0 | CNPY2 | 64/114(56.14) | 0.33 | 4.00E-08 | 2.99E-07 | Yes | Up | - |
| Q49AR2 | C5ORF22 | 61/114(53.51) | 0.17 | 1.76E-02 | 3.21E-02 | Yes | Up | - |
| Q5TCY1 | TTBK1 | 64/114(56.14) | 0.64 | 1.17E-04 | 3.62E-04 | Yes | Up | - |
| Q99941 | ATF6B | 62/114(54.39) | 0.33 | 1.29E-07 | 8.42E-07 | Yes | Up | - |
| Q99470 | SDF2 | 61/114(53.51) | 0.34 | 1.46E-08 | 1.24E-07 | Yes | Up | - |
| Q16690 | DUSP5 | 59/114(51.75) | 0.45 | 5.89E-05 | 1.96E-04 | Yes | Up | - |
| Q9UJ71 | CD207 | 54/114(47.37) | 0.5 | 6.17E-03 | 1.26E-02 | Yes | Up | - |
| Q9P1U0 | ZNRD1 | 63/114(55.26) | 0.18 | 1.06E-03 | 2.60E-03 | Yes | Up | - |
| Q92925 | SMARCD2 | 55/114(48.25) | 0.21 | 5.91E-04 | 1.54E-03 | Yes | Up | - |
| Q96I15 | SCLY | 63/114(55.26) | 0.23 | 1.74E-04 | 5.17E-04 | Yes | Up | - |
| Q8N8Z6 | DCBLD1 | 56/114(49.12) | 0.22 | 9.69E-03 | 1.88E-02 | Yes | Up | - |
| O95484 | CLDN9 | 63/114(55.26) | 0.64 | 4.85E-05 | 1.64E-04 | Yes | Up | - |
| Q9Y5V3 | MAGED1 | 63/114(55.26) | 0.56 | 7.99E-08 | 5.52E-07 | Yes | Up | - |
| Q96FV0 | LRRC46 | 61/114(53.51) | 1.25 | 5.59E-13 | 1.98E-11 | Yes | Up | - |
| Q9H6T3 | RPAP3 | 53/114(46.49) | 0.19 | 2.47E-03 | 5.54E-03 | Yes | Up | - |
| Q2KHT3 | CLEC16A | 59/114(51.75) | 0.14 | 1.60E-02 | 2.95E-02 | Yes | Up | - |
| Q8TCT9 | HM13 | 59/114(51.75) | 0.48 | 1.69E-08 | 1.42E-07 | Yes | Up | - |
| Q96N19 | GPR137 | 60/114(52.63) | 0.39 | 8.25E-07 | 4.34E-06 | Yes | Up | - |
| P14314 | PRKCSH | 65/114(57.02) | 0.31 | 2.43E-06 | 1.13E-05 | Yes | Up | - |
| Q86UW9 | DTX2 | 60/114(52.63) | 0.43 | 7.54E-08 | 5.24E-07 | Yes | Up | - |
| Q8NAC3 | IL17RC | 67/114(58.77) | 0.17 | 1.71E-02 | 3.13E-02 | Yes | Up | - |
| Q6NT04 | TIGD7 | 60/114(52.63) | 0.15 | 2.67E-02 | 4.65E-02 | Yes | Up | - |
| Q9HBE1 | PATZ1 | 54/114(47.37) | 0.29 | 2.13E-04 | 6.18E-04 | Yes | Up | - |
| Q9BTK6 | C16ORF53 | 62/114(54.39) | 0.65 | 1.41E-13 | 6.36E-12 | Yes | Up | - |
| P01574 | IFNB1 | 39/114(34.21) | 1.01 | 2.23E-02 | 3.97E-02 | Yes | Up | - |
| Q96L58 | B3GALT6 | 56/114(49.12) | 0.24 | 4.94E-05 | 1.67E-04 | Yes | Up | - |
| P07954 | FH | 58/114(50.88) | 0.27 | 7.05E-04 | 1.81E-03 | Yes | Up | - |
| O43482 | OIP5 | 61/114(53.51) | 1.37 | 1.24E-12 | 3.86E-11 | Yes | Up | - |
| P56705 | WNT4 | 69/114(60.53) | 0.38 | 8.46E-03 | 1.67E-02 | Yes | Up | - |
| P62820 | RAB1A | 62/114(54.39) | 0.13 | 1.33E-02 | 2.49E-02 | Yes | Up | - |
| P09913 | IFIT2 | 58/114(50.88) | 0.3 | 1.44E-02 | 2.69E-02 | Yes | Up | - |
| Q6ZMU5 | TRIM72 | 50/114(43.86) | 1.46 | 7.42E-08 | 5.17E-07 | Yes | Up | - |
| O95758 | ROD1 | 59/114(51.75) | 0.3 | 3.82E-04 | 1.04E-03 | Yes | Up | - |
| Q9H7M6 | ZSWIM4 | 63/114(55.26) | 0.5 | 2.47E-07 | 1.49E-06 | Yes | Up | - |
| O95168 | NDUFB4 | 62/114(54.39) | 0.33 | 3.14E-06 | 1.42E-05 | Yes | Up | - |
| Q16520 | BATF | 66/114(57.89) | 0.76 | 8.26E-09 | 7.51E-08 | Yes | Up | - |
| Q8WUU4 | ZNF296 | 62/114(54.39) | 0.91 | 2.80E-09 | 2.92E-08 | Yes | Up | - |
| O95994 | AGR2 | 63/114(55.26) | 1.32 | 9.79E-08 | 6.59E-07 | Yes | Up | - |
| Q18PE1 | DOK7 | 63/114(55.26) | 1.19 | 7.65E-09 | 7.04E-08 | Yes | Up | - |
| Q96IR7 | HPDL | 57/114(50.0) | 0.39 | 2.87E-02 | 4.96E-02 | Yes | Up | - |
| Q7Z570 | ZNF804A | 66/114(57.89) | 0.36 | 6.14E-03 | 1.26E-02 | Yes | Up | - |
| Q9NUC0 | SERTAD4 | 55/114(48.25) | 0.27 | 2.87E-02 | 4.96E-02 | Yes | Up | - |
| Q9UL54 | TAOK2 | 60/114(52.63) | 0.18 | 2.91E-03 | 6.40E-03 | Yes | Up | - |
| O15014 | ZNF609 | 57/114(50.0) | 0.14 | 1.83E-02 | 3.32E-02 | Yes | Up | - |
| P13798 | APEH | 64/114(56.14) | 0.31 | 1.23E-04 | 3.78E-04 | Yes | Up | - |
| P57735 | RAB25 | 59/114(51.75) | 0.85 | 9.51E-06 | 3.84E-05 | Yes | Up | - |
| P48723 | HSPA13 | 59/114(51.75) | 0.26 | 1.20E-03 | 2.91E-03 | Yes | Up | - |
| Q5T6F0 | DCAF12 | 54/114(47.37) | 0.15 | 8.90E-03 | 1.75E-02 | Yes | Up | - |
| Q9NYT0 | PLEK2 | 61/114(53.51) | 0.79 | 1.44E-06 | 7.11E-06 | Yes | Up | - |
| Q13442 | PDAP1 | 67/114(58.77) | 0.22 | 9.00E-04 | 2.25E-03 | Yes | Up | - |
| Q9Y4Z0 | LSM4 | 63/114(55.26) | 0.63 | 4.67E-11 | 8.49E-10 | Yes | Up | - |
| Q9NP99 | TREM1 | 66/114(57.89) | 0.91 | 2.65E-06 | 1.22E-05 | Yes | Up | - |
| O60282 | KIF5C | 56/114(49.12) | 0.47 | 5.40E-03 | 1.12E-02 | Yes | Up | - |
| O95196 | CSPG5 | 63/114(55.26) | 0.44 | 1.54E-03 | 3.64E-03 | Yes | Up | - |
| Q8N5Z5 | KCTD17 | 65/114(57.02) | 0.37 | 5.25E-05 | 1.76E-04 | Yes | Up | - |
| Q6ZTR5 | CXORF22 | 56/114(49.12) | 1.32 | 1.68E-07 | 1.06E-06 | Yes | Up | - |
| P30405 | PPIF | 64/114(56.14) | 0.27 | 9.22E-03 | 1.81E-02 | Yes | Up | - |
| B4DU43 | C10ORF57 | 58/114(50.88) | 0.37 | 1.44E-05 | 5.55E-05 | Yes | Up | - |
| O43709 | WBSCR22 | 67/114(58.77) | 0.4 | 2.48E-08 | 1.98E-07 | Yes | Up | - |
| Q9BWL3 | C1ORF43 | 60/114(52.63) | 0.42 | 9.52E-08 | 6.42E-07 | Yes | Up | - |
| P40938 | RFC3 | 59/114(51.75) | 0.34 | 1.66E-04 | 4.95E-04 | Yes | Up | - |
| Q9UK41 | VPS28 | 64/114(56.14) | 0.37 | 6.60E-07 | 3.56E-06 | Yes | Up | - |
| Q9UQM7 | CAMK2A | 57/114(50.0) | 0.37 | 2.44E-02 | 4.29E-02 | Yes | Up | - |
| Q9BTE0 | NAT9 | 54/114(47.37) | 0.3 | 2.45E-05 | 8.92E-05 | Yes | Up | - |
| Q15800 | SC4MOL | 58/114(50.88) | 0.3 | 8.22E-03 | 1.63E-02 | Yes | Up | - |
| Q5JR12 | PPM1J | 68/114(59.65) | 1.1 | 9.91E-12 | 2.30E-10 | Yes | Up | - |
| O43299 | KIAA0415 | 60/114(52.63) | 0.26 | 1.93E-04 | 5.65E-04 | Yes | Up | - |
| Q96MX3 | ZNF48 | 60/114(52.63) | 0.51 | 9.42E-09 | 8.43E-08 | Yes | Up | - |
| Q53HV7 | SMUG1 | 59/114(51.75) | 0.24 | 1.07E-05 | 4.27E-05 | Yes | Up | - |
| P51679 | CCR4 | 58/114(50.88) | 0.91 | 4.39E-07 | 2.47E-06 | Yes | Up | - |
| Q96HR9 | REEP6 | 61/114(53.51) | 0.84 | 6.27E-07 | 3.40E-06 | Yes | Up | - |
| Q96S15 | WDR24 | 64/114(56.14) | 0.4 | 1.83E-07 | 1.15E-06 | Yes | Up | - |
| Q86UY5 | FAM83A | 58/114(50.88) | 0.95 | 9.33E-06 | 3.77E-05 | Yes | Up | - |
| Q8N7C0 | LRRC52 | 48/114(42.11) | 0.96 | 1.41E-03 | 3.37E-03 | Yes | Up | - |
| Q8IYA6 | CKAP2L | 57/114(50.0) | 1.49 | 9.97E-12 | 2.31E-10 | Yes | Up | - |
| Q13286 | CLN3 | 56/114(49.12) | 0.56 | 6.22E-11 | 1.09E-09 | Yes | Up | - |
| O14561 | NDUFAB1 | 62/114(54.39) | 0.3 | 3.60E-05 | 1.26E-04 | Yes | Up | - |
| Q9NRR8 | CDC42SE1 | 55/114(48.25) | 0.37 | 5.50E-07 | 3.03E-06 | Yes | Up | - |
| Q9NPC8 | SIX2 | 54/114(47.37) | 0.54 | 3.13E-03 | 6.84E-03 | Yes | Up | - |
| P54920 | NAPA | 60/114(52.63) | 0.27 | 1.82E-05 | 6.86E-05 | Yes | Up | - |
| A6NHQ4 | C17ORF96 | 62/114(54.39) | 0.46 | 3.91E-04 | 1.06E-03 | Yes | Up | - |
| Q92968 | PEX13 | 62/114(54.39) | 0.28 | 8.68E-05 | 2.77E-04 | Yes | Up | - |
| Q9BRK5 | SDF4 | 61/114(53.51) | 0.25 | 6.50E-04 | 1.68E-03 | Yes | Up | - |
| Q8WTW4 | NPRL2 | 64/114(56.14) | 0.26 | 7.21E-05 | 2.34E-04 | Yes | Up | - |
| Q86VI3 | IQGAP3 | 61/114(53.51) | 1.79 | 3.88E-15 | 3.49E-13 | Yes | Up | - |
| A5D8V6 | VPS37C | 59/114(51.75) | 0.45 | 1.51E-10 | 2.32E-09 | Yes | Up | - |
| P19022 | CDH2 | 60/114(52.63) | 1.04 | 2.83E-09 | 2.95E-08 | Yes | Up | - |
| Q8NBZ7 | UXS1 | 58/114(50.88) | 0.13 | 8.76E-03 | 1.72E-02 | Yes | Up | - |
| P58417 | NXPH1 | 56/114(49.12) | 1.69 | 1.73E-08 | 1.45E-07 | Yes | Up | - |
| P61966 | AP1S1 | 63/114(55.26) | 0.56 | 3.73E-09 | 3.74E-08 | Yes | Up | - |
| Q8WZ71 | TMEM158 | 58/114(50.88) | 0.55 | 5.56E-04 | 1.46E-03 | Yes | Up | - |
| Q9NVJ2 | ARL8B | 51/114(44.74) | 0.16 | 4.09E-03 | 8.71E-03 | Yes | Up | - |
| P02655 | APOC2 | 66/114(57.89) | 1.09 | 2.30E-08 | 1.85E-07 | Yes | Up | - |
| P41227 | NAA10 | 63/114(55.26) | 0.25 | 8.46E-05 | 2.71E-04 | Yes | Up | - |
| O00442 | RTCD1 | 53/114(46.49) | 0.14 | 2.34E-02 | 4.13E-02 | Yes | Up | - |
| Q9BWX5 | GATA5 | 60/114(52.63) | 2.62 | 5.51E-19 | 2.09E-16 | Yes | Up | - |
| P53680 | AP2S1 | 65/114(57.02) | 0.38 | 3.62E-06 | 1.61E-05 | Yes | Up | - |
| O75618 | DEDD | 58/114(50.88) | 0.25 | 1.82E-05 | 6.86E-05 | Yes | Up | - |
| - | LOC284441 | 61/114(53.51) | 0.19 | 2.41E-02 | 4.24E-02 | Yes | Up | - |
| O14949 | UQCRQ | 61/114(53.51) | 0.37 | 9.43E-05 | 2.99E-04 | Yes | Up | - |
| Q8N967 | LRTM2 | 52/114(45.61) | 1.48 | 6.04E-07 | 3.29E-06 | Yes | Up | - |
| Q969S8 | HDAC10 | 63/114(55.26) | 0.24 | 9.12E-04 | 2.27E-03 | Yes | Up | - |
| - | LOC152225 | 56/114(49.12) | 1.91 | 8.22E-17 | 1.46E-14 | Yes | Up | - |
| Q8WWY3 | PRPF31 | 72/114(63.16) | 0.21 | 1.54E-04 | 4.63E-04 | Yes | Up | - |
| P52943 | CRIP2 | 66/114(57.89) | 0.42 | 1.34E-05 | 5.21E-05 | Yes | Up | - |
| O95793 | STAU1 | 58/114(50.88) | 0.23 | 3.61E-04 | 9.92E-04 | Yes | Up | - |
| - | C8ORF75 | 52/114(45.61) | 0.93 | 2.11E-03 | 4.83E-03 | Yes | Up | - |
| Q9ULH4 | LRFN2 | 59/114(51.75) | 1.66 | 2.85E-13 | 1.13E-11 | Yes | Up | - |
| Q9BUI4 | POLR3C | 56/114(49.12) | 0.19 | 7.58E-05 | 2.45E-04 | Yes | Up | - |
| Q5SRE5 | NUP188 | 52/114(45.61) | 0.12 | 2.77E-02 | 4.80E-02 | Yes | Up | - |
| P21860 | ERBB3 | 55/114(48.25) | 0.76 | 1.31E-05 | 5.13E-05 | Yes | Up | - |
| Q9UGV2 | NDRG3 | 61/114(53.51) | 0.16 | 4.68E-03 | 9.83E-03 | Yes | Up | - |
| - | DGCR11 | 54/114(47.37) | 0.18 | 2.09E-02 | 3.74E-02 | Yes | Up | - |
| P31641 | SLC6A6 | 64/114(56.14) | 0.23 | 1.61E-03 | 3.80E-03 | Yes | Up | - |
| P31946 | YWHAB | 54/114(47.37) | 0.18 | 1.71E-03 | 4.01E-03 | Yes | Up | - |
| O00519 | FAAH | 61/114(53.51) | 0.36 | 3.04E-04 | 8.50E-04 | Yes | Up | - |
| P48549 | KCNJ3 | 52/114(45.61) | 1.55 | 2.35E-08 | 1.89E-07 | Yes | Up | - |
| - | LOC113230 | 62/114(54.39) | 0.87 | 3.75E-12 | 9.99E-11 | Yes | Up | - |
| A6NJZ7 | RIMBP3C | 55/114(48.25) | 0.91 | 3.54E-10 | 4.85E-09 | Yes | Up | - |
| Q9GZT8 | NIF3L1 | 65/114(57.02) | 0.11 | 1.61E-02 | 2.96E-02 | Yes | Up | - |
| Q86V81 | THOC4 | 64/114(56.14) | 0.48 | 8.35E-08 | 5.73E-07 | Yes | Up | - |
| Q9BRP8 | WIBG | 72/114(63.16) | 0.36 | 1.03E-08 | 9.09E-08 | Yes | Up | - |
| Q9HBW0 | LPAR2 | 61/114(53.51) | 0.77 | 4.21E-11 | 7.83E-10 | Yes | Up | - |
| P23284 | PPIB | 61/114(53.51) | 0.39 | 5.50E-07 | 3.03E-06 | Yes | Up | - |
| Q9NUW8 | TDP1 | 58/114(50.88) | 0.21 | 1.45E-03 | 3.44E-03 | Yes | Up | - |
| Q8ND04 | C17ORF71 | 55/114(48.25) | 0.23 | 6.89E-04 | 1.77E-03 | Yes | Up | - |
| Q8IXM3 | MRPL41 | 61/114(53.51) | 0.36 | 7.91E-05 | 2.55E-04 | Yes | Up | - |
| B7WNH4 | C19ORF42 | 56/114(49.12) | 0.17 | 6.79E-04 | 1.75E-03 | Yes | Up | - |
| P17035 | ZNF28 | 51/114(44.74) | 0.36 | 9.13E-05 | 2.90E-04 | Yes | Up | - |
| Q14566 | MCM6 | 59/114(51.75) | 0.42 | 7.47E-06 | 3.09E-05 | Yes | Up | - |
| Q99437 | ATP6V0B | 63/114(55.26) | 0.68 | 5.01E-12 | 1.27E-10 | Yes | Up | - |
| - | FLJ16779 | 61/114(53.51) | 0.68 | 1.19E-03 | 2.88E-03 | Yes | Up | - |
| Q9H7D7 | WDR26 | 52/114(45.61) | 0.19 | 1.64E-03 | 3.86E-03 | Yes | Up | - |
| Q24JP5 | TMEM132A | 63/114(55.26) | 0.92 | 1.83E-09 | 2.00E-08 | Yes | Up | - |
| Q8N6Q3 | CD177 | 62/114(54.39) | 2.32 | 9.39E-25 | 2.59E-21 | Yes | Up | - |
| O60841 | EIF5B | 56/114(49.12) | 0.18 | 5.73E-04 | 1.50E-03 | Yes | Up | - |
| Q12996 | CSTF3 | 53/114(46.49) | 0.26 | 7.36E-05 | 2.39E-04 | Yes | Up | - |
| Q13015 | MLLT11 | 60/114(52.63) | 0.51 | 9.40E-05 | 2.98E-04 | Yes | Up | - |
| O60513 | B4GALT4 | 58/114(50.88) | 0.3 | 7.86E-06 | 3.23E-05 | Yes | Up | - |
| O95861 | BPNT1 | 61/114(53.51) | 0.42 | 9.36E-07 | 4.85E-06 | Yes | Up | - |
| P06732 | CKM | 62/114(54.39) | 0.56 | 1.80E-02 | 3.27E-02 | Yes | Up | - |
| Q7Z5W3 | BCDIN3D | 62/114(54.39) | 0.23 | 2.81E-04 | 7.91E-04 | Yes | Up | - |
| Q9UMY1 | NOL7 | 64/114(56.14) | 0.14 | 7.61E-03 | 1.52E-02 | Yes | Up | - |
| Q12974 | PTP4A2 | 51/114(44.74) | 0.28 | 1.23E-03 | 2.97E-03 | Yes | Up | - |
| H0UI80 | TH1L | 55/114(48.25) | 0.24 | 1.49E-03 | 3.54E-03 | Yes | Up | - |
| O14492 | SH2B2 | 67/114(58.77) | 0.43 | 2.98E-04 | 8.35E-04 | Yes | Up | - |
| P49913 | CAMP | 59/114(51.75) | 1.48 | 1.08E-07 | 7.16E-07 | Yes | Up | - |
| P67936 | TPM4 | 67/114(58.77) | 0.31 | 8.40E-07 | 4.41E-06 | Yes | Up | - |
| D6W4K3 | KIAA0406 | 57/114(50.0) | 0.24 | 5.51E-04 | 1.45E-03 | Yes | Up | - |
| Q6ZTR7 | FAM92B | 54/114(47.37) | 0.67 | 2.48E-03 | 5.57E-03 | Yes | Up | - |
| Q96EA4 | CCDC99 | 60/114(52.63) | 0.38 | 1.74E-05 | 6.57E-05 | Yes | Up | - |
| Q6GQQ9 | OTUD7B | 57/114(50.0) | 0.21 | 1.13E-03 | 2.75E-03 | Yes | Up | - |
| Q8IUG5 | MYO18B | 57/114(50.0) | 0.52 | 1.23E-02 | 2.32E-02 | Yes | Up | - |
| A6NN90 | C2ORF81 | 64/114(56.14) | 0.47 | 4.62E-06 | 2.00E-05 | Yes | Up | - |
| Q5W0U4 | BSPRY | 63/114(55.26) | 0.53 | 2.12E-03 | 4.84E-03 | Yes | Up | - |
| A5PLL7 | TMEM189 | 58/114(50.88) | 0.18 | 2.41E-02 | 4.25E-02 | Yes | Up | - |
| Q15743 | GPR68 | 64/114(56.14) | 1.13 | 8.63E-17 | 1.52E-14 | Yes | Up | - |
| Q7RTY0 | SLC16A13 | 59/114(51.75) | 0.22 | 8.87E-03 | 1.74E-02 | Yes | Up | - |
| Q8NCR0 | B3GALNT2 | 57/114(50.0) | 0.19 | 9.58E-03 | 1.86E-02 | Yes | Up | - |
| O14929 | HAT1 | 58/114(50.88) | 0.17 | 3.96E-03 | 8.44E-03 | Yes | Up | - |
| Q6ZVK8 | NUDT18 | 59/114(51.75) | 0.32 | 1.90E-04 | 5.57E-04 | Yes | Up | - |
| P25788 | PSMA3 | 61/114(53.51) | 0.22 | 1.69E-03 | 3.95E-03 | Yes | Up | - |
| O75365 | PTP4A3 | 63/114(55.26) | 0.49 | 2.98E-07 | 1.76E-06 | Yes | Up | - |
| Q8NFT6 | DBF4B | 60/114(52.63) | 0.19 | 1.10E-02 | 2.12E-02 | Yes | Up | - |
| Q96GX8 | C16ORF74 | 58/114(50.88) | 0.46 | 1.41E-04 | 4.28E-04 | Yes | Up | - |
| Q6Y2X3 | DNAJC14 | 56/114(49.12) | 0.27 | 5.71E-10 | 7.36E-09 | Yes | Up | - |
| Q8TDX5 | ACMSD | 59/114(51.75) | 1.23 | 2.08E-05 | 7.74E-05 | Yes | Up | - |
| Q8NHJ6 | LILRB4 | 60/114(52.63) | 0.6 | 4.97E-06 | 2.14E-05 | Yes | Up | - |
| P05997 | COL5A2 | 60/114(52.63) | 1.13 | 1.70E-11 | 3.61E-10 | Yes | Up | - |
| P63218 | GNG5 | 64/114(56.14) | 0.31 | 6.28E-05 | 2.07E-04 | Yes | Up | - |
| Q9P202 | DFNB31 | 62/114(54.39) | 0.24 | 2.56E-02 | 4.48E-02 | Yes | Up | - |
| P51580 | TPMT | 59/114(51.75) | 0.33 | 6.90E-05 | 2.25E-04 | Yes | Up | - |
| Q96MF7 | NSMCE2 | 63/114(55.26) | 0.18 | 1.51E-02 | 2.79E-02 | Yes | Up | - |
| Q9UGT4 | SUSD2 | 63/114(55.26) | 0.55 | 3.72E-06 | 1.65E-05 | Yes | Up | - |
| Q9BZV3 | IMPG2 | 67/114(58.77) | 0.33 | 1.06E-02 | 2.04E-02 | Yes | Up | - |
| O15382 | BCAT2 | 64/114(56.14) | 0.39 | 6.71E-07 | 3.60E-06 | Yes | Up | - |
| Q7Z4W1 | DCXR | 60/114(52.63) | 0.25 | 6.89E-03 | 1.39E-02 | Yes | Up | - |
| Q9BV36 | MLPH | 56/114(49.12) | 0.87 | 1.61E-06 | 7.82E-06 | Yes | Up | - |
| Q330K2 | C8ORF38 | 59/114(51.75) | 0.67 | 9.78E-10 | 1.17E-08 | Yes | Up | - |
| Q8IVL6 | LEPREL2 | 59/114(51.75) | 0.53 | 6.54E-06 | 2.73E-05 | Yes | Up | - |
| Q6HA08 | ASTL | 59/114(51.75) | 0.58 | 7.44E-03 | 1.49E-02 | Yes | Up | - |
| P58397 | ADAMTS12 | 53/114(46.49) | 0.41 | 3.34E-03 | 7.24E-03 | Yes | Up | - |
| Q9NUX5 | POT1 | 59/114(51.75) | 0.16 | 1.72E-02 | 3.14E-02 | Yes | Up | - |
| O95685 | PPP1R3D | 50/114(43.86) | 0.2 | 6.55E-03 | 1.33E-02 | Yes | Up | - |
| P42768 | WAS | 64/114(56.14) | 0.29 | 5.81E-03 | 1.20E-02 | Yes | Up | - |
| Q5VU69 | C1ORF189 | 31/114(27.19) | 0.94 | 2.74E-03 | 6.08E-03 | Yes | Up | - |
| Q5T953 | IER5L | 66/114(57.89) | 0.7 | 1.91E-08 | 1.58E-07 | Yes | Up | - |
| Q8NAX2 | C1ORF172 | 57/114(50.0) | 0.5 | 6.05E-03 | 1.24E-02 | Yes | Up | - |
| Q9H9V9 | JMJD4 | 64/114(56.14) | 0.43 | 4.35E-08 | 3.23E-07 | Yes | Up | - |
| Q08AG7 | C13ORF37 | 58/114(50.88) | 0.24 | 6.20E-03 | 1.27E-02 | Yes | Up | - |
| - | LOC389033 | 62/114(54.39) | 0.61 | 1.57E-02 | 2.90E-02 | Yes | Up | - |
| A0MZ66 | KIAA1598 | 56/114(49.12) | 0.59 | 6.67E-08 | 4.70E-07 | Yes | Up | - |
| Q9H1C4 | UNC93B1 | 62/114(54.39) | 0.47 | 1.12E-07 | 7.39E-07 | Yes | Up | - |
| Q14982 | OPCML | 64/114(56.14) | 0.41 | 1.93E-02 | 3.48E-02 | Yes | Up | - |
| P60606 | CTXN1 | 68/114(59.65) | 1.3 | 7.56E-14 | 3.80E-12 | Yes | Up | - |
| Q86WA9 | SLC26A11 | 62/114(54.39) | 0.31 | 3.87E-06 | 1.71E-05 | Yes | Up | - |
| O95563 | BRP44 | 58/114(50.88) | 0.37 | 1.07E-05 | 4.28E-05 | Yes | Up | - |
| P68371 | TUBB2C | 68/114(59.65) | 0.49 | 7.58E-10 | 9.37E-09 | Yes | Up | - |
| Q8IZE3 | SCYL3 | 51/114(44.74) | 0.26 | 3.76E-04 | 1.03E-03 | Yes | Up | - |
| Q9ULU8 | CADPS | 64/114(56.14) | 0.96 | 1.43E-07 | 9.20E-07 | Yes | Up | - |
| P13010 | XRCC5 | 59/114(51.75) | 0.16 | 4.36E-04 | 1.17E-03 | Yes | Up | - |
| Q8WXR4 | MYO3B | 50/114(43.86) | 0.5 | 1.59E-02 | 2.94E-02 | Yes | Up | - |
| Q9BTM1 | H2AFJ | 60/114(52.63) | 0.41 | 3.10E-05 | 1.10E-04 | Yes | Up | - |
| Q9UBF6 | RNF7 | 61/114(53.51) | 0.22 | 2.95E-05 | 1.05E-04 | Yes | Up | - |
| O75608 | LYPLA1 | 60/114(52.63) | 0.46 | 2.43E-05 | 8.88E-05 | Yes | Up | - |
| Q9BY41 | HDAC8 | 66/114(57.89) | 0.21 | 2.03E-06 | 9.63E-06 | Yes | Up | - |
| Q4ZG55 | GREB1 | 57/114(50.0) | 0.54 | 1.77E-03 | 4.12E-03 | Yes | Up | - |
| Q460N5 | PARP14 | 52/114(45.61) | 0.32 | 2.63E-04 | 7.46E-04 | Yes | Up | - |
| Q9Y242 | TCF19 | 58/114(50.88) | 0.48 | 3.99E-06 | 1.75E-05 | Yes | Up | - |
| Q92542 | NCSTN | 57/114(50.0) | 0.29 | 1.47E-05 | 5.67E-05 | Yes | Up | - |
| P35052 | GPC1 | 68/114(59.65) | 0.21 | 1.52E-02 | 2.82E-02 | Yes | Up | - |
| P62072 | TIMM10 | 60/114(52.63) | 0.35 | 6.43E-07 | 3.47E-06 | Yes | Up | - |
| P09525 | ANXA4 | 62/114(54.39) | 0.12 | 1.50E-02 | 2.79E-02 | Yes | Up | - |
| Q9NWW9 | HRASLS2 | 57/114(50.0) | 0.97 | 8.78E-07 | 4.58E-06 | Yes | Up | - |
| Q10713 | PMPCA | 63/114(55.26) | 0.15 | 5.40E-03 | 1.12E-02 | Yes | Up | - |
| P25089 | FPR3 | 60/114(52.63) | 0.56 | 8.04E-05 | 2.59E-04 | Yes | Up | - |
| P55259 | GP2 | 57/114(50.0) | 1.52 | 2.48E-07 | 1.50E-06 | Yes | Up | - |
| O14874 | BCKDK | 62/114(54.39) | 0.31 | 2.95E-06 | 1.34E-05 | Yes | Up | - |
| Q9HAH7 | FBRS | 63/114(55.26) | 0.37 | 5.90E-09 | 5.59E-08 | Yes | Up | - |
| O75132 | ZBED4 | 53/114(46.49) | 0.24 | 1.86E-03 | 4.32E-03 | Yes | Up | - |
| P10599 | TXN | 61/114(53.51) | 0.38 | 7.47E-06 | 3.09E-05 | Yes | Up | - |
| Q8NES3 | LFNG | 59/114(51.75) | 0.31 | 1.21E-02 | 2.30E-02 | Yes | Up | - |
| Q9BUQ8 | DDX23 | 63/114(55.26) | 0.22 | 9.30E-06 | 3.76E-05 | Yes | Up | - |
| Q9H3R5 | CENPH | 65/114(57.02) | 0.42 | 1.67E-05 | 6.34E-05 | Yes | Up | - |
| P11388 | TOP2A | 60/114(52.63) | 1.67 | 1.21E-13 | 5.66E-12 | Yes | Up | - |
| Q969V4 | TEKT1 | 43/114(37.72) | 1.12 | 8.27E-04 | 2.09E-03 | Yes | Up | - |
| O14756 | HSD17B6 | 59/114(51.75) | 1.91 | 8.25E-21 | 5.31E-18 | Yes | Up | - |
| Q01650 | SLC7A5 | 60/114(52.63) | 1.12 | 6.32E-10 | 8.02E-09 | Yes | Up | - |
| Q9NS18 | GLRX2 | 57/114(50.0) | 0.41 | 6.94E-05 | 2.27E-04 | Yes | Up | - |
| Q12914 | C11ORF41 | 58/114(50.88) | 0.49 | 4.37E-04 | 1.18E-03 | Yes | Up | - |
| Q9HB75 | LRDD | 58/114(50.88) | 0.26 | 7.87E-04 | 2.00E-03 | Yes | Up | - |
| Q16514 | TAF12 | 64/114(56.14) | 0.15 | 2.86E-03 | 6.31E-03 | Yes | Up | - |
| Q8N5H3 | FAM89B | 60/114(52.63) | 0.32 | 3.37E-05 | 1.19E-04 | Yes | Up | - |
| Q7Z7G2 | CPLX4 | 45/114(39.47) | 0.78 | 7.10E-03 | 1.43E-02 | Yes | Up | - |
| Q9NXE4 | SMPD4 | 62/114(54.39) | 0.27 | 5.33E-07 | 2.95E-06 | Yes | Up | - |
| Q16667 | CDKN3 | 62/114(54.39) | 1.47 | 4.78E-14 | 2.65E-12 | Yes | Up | - |
| Q3KRB8 | ARHGAP11B | 56/114(49.12) | 0.78 | 6.00E-06 | 2.53E-05 | Yes | Up | - |
| O75173 | ADAMTS4 | 66/114(57.89) | 0.66 | 4.49E-05 | 1.53E-04 | Yes | Up | - |
| Q03426 | MVK | 59/114(51.75) | 0.2 | 1.21E-02 | 2.29E-02 | Yes | Up | - |
| Q9Y6U7 | RNF215 | 68/114(59.65) | 0.16 | 9.65E-03 | 1.88E-02 | Yes | Up | - |
| Q969F8 | KISS1R | 56/114(49.12) | 1.24 | 8.99E-07 | 4.68E-06 | Yes | Up | - |
| O75800 | ZMYND10 | 62/114(54.39) | 1.45 | 1.84E-13 | 7.93E-12 | Yes | Up | - |
| Q9Y3L5 | RAP2C | 50/114(43.86) | 0.45 | 4.15E-06 | 1.81E-05 | Yes | Up | - |
| Q08AI8 | C2ORF54 | 65/114(57.02) | 1.35 | 1.97E-07 | 1.22E-06 | Yes | Up | - |
| Q14409 | GK3P | 57/114(50.0) | 0.53 | 2.04E-06 | 9.69E-06 | Yes | Up | - |
| Q9NQ29 | LUC7L | 63/114(55.26) | 0.14 | 2.51E-02 | 4.40E-02 | Yes | Up | - |
| Q96BZ8 | LENG1 | 62/114(54.39) | 0.23 | 1.88E-02 | 3.41E-02 | Yes | Up | - |
| P10276 | RARA | 66/114(57.89) | 0.64 | 2.38E-09 | 2.52E-08 | Yes | Up | - |
| Q86W10 | CYP4Z1 | 60/114(52.63) | 0.75 | 8.47E-03 | 1.67E-02 | Yes | Up | - |
| Q96J94 | PIWIL1 | 45/114(39.47) | 0.62 | 2.25E-02 | 3.99E-02 | Yes | Up | - |
| Q8TAF5 | LQK1 | 64/114(56.14) | 0.33 | 1.57E-04 | 4.72E-04 | Yes | Up | - |
| Q8IXU6 | SLC35F2 | 49/114(42.98) | 0.45 | 7.26E-05 | 2.36E-04 | Yes | Up | - |
| Q95460 | MR1 | 48/114(42.11) | 0.3 | 1.89E-04 | 5.56E-04 | Yes | Up | - |
| P19544 | WT1 | 65/114(57.02) | 1.8 | 4.97E-09 | 4.82E-08 | Yes | Up | - |
| P61923 | COPZ1 | 65/114(57.02) | 0.29 | 6.01E-07 | 3.28E-06 | Yes | Up | - |
| P33992 | MCM5 | 64/114(56.14) | 0.26 | 5.69E-04 | 1.49E-03 | Yes | Up | - |
| Q99728 | BARD1 | 58/114(50.88) | 0.58 | 6.26E-06 | 2.63E-05 | Yes | Up | - |
| Q96GX5 | MASTL | 62/114(54.39) | 0.27 | 1.44E-03 | 3.43E-03 | Yes | Up | - |
| Q14190 | SIM2 | 56/114(49.12) | 0.57 | 4.16E-04 | 1.13E-03 | Yes | Up | - |
| Q92535 | PIGC | 55/114(48.25) | 0.36 | 4.22E-08 | 3.14E-07 | Yes | Up | - |
| Q8N6T7 | SIRT6 | 69/114(60.53) | 0.46 | 2.51E-10 | 3.59E-09 | Yes | Up | - |
| Q96MT3 | PRICKLE1 | 63/114(55.26) | 0.26 | 7.60E-03 | 1.52E-02 | Yes | Up | - |
| Q8IY82 | CCDC135 | 51/114(44.74) | 1.05 | 1.65E-05 | 6.29E-05 | Yes | Up | - |
| P52566 | ARHGDIB | 65/114(57.02) | 0.39 | 1.24E-05 | 4.89E-05 | Yes | Up | - |
| P55210 | CASP7 | 63/114(55.26) | 0.16 | 9.71E-03 | 1.89E-02 | Yes | Up | - |
| O76094 | SRP72 | 54/114(47.37) | 0.16 | 2.92E-03 | 6.42E-03 | Yes | Up | - |
| O75325 | LRRN2 | 57/114(50.0) | 0.71 | 9.97E-07 | 5.12E-06 | Yes | Up | - |
| Q49AJ0 | FAM135B | 67/114(58.77) | 0.79 | 1.11E-04 | 3.44E-04 | Yes | Up | - |
| Q6S9Z5 | ZNF474 | 64/114(56.14) | 0.69 | 9.97E-08 | 6.69E-07 | Yes | Up | - |
| Q07817 | BCL2L1 | 63/114(55.26) | 0.26 | 1.73E-05 | 6.55E-05 | Yes | Up | - |
| Q9NX74 | DUS2L | 60/114(52.63) | 0.15 | 9.18E-03 | 1.80E-02 | Yes | Up | - |
| Q32MZ4 | LRRFIP1 | 57/114(50.0) | 0.21 | 1.03E-03 | 2.54E-03 | Yes | Up | - |
| Q9UGN4 | CD300A | 62/114(54.39) | 0.21 | 2.05E-02 | 3.67E-02 | Yes | Up | - |
| P56703 | WNT3 | 52/114(45.61) | 0.34 | 2.52E-02 | 4.42E-02 | Yes | Up | - |
| Q12893 | TMEM115 | 63/114(55.26) | 0.12 | 2.11E-02 | 3.76E-02 | Yes | Up | - |
| Q5T6C5 | ATXN7L2 | 63/114(55.26) | 0.24 | 2.47E-03 | 5.54E-03 | Yes | Up | - |
| Q9NQ31 | C11ORF17 | 62/114(54.39) | 0.19 | 1.46E-03 | 3.47E-03 | Yes | Up | - |
| Q96HT8 | MRFAP1L1 | 58/114(50.88) | 0.16 | 2.30E-03 | 5.21E-03 | Yes | Up | - |
| P68363 | TUBA1B | 60/114(52.63) | 0.43 | 8.97E-09 | 8.08E-08 | Yes | Up | - |
| O60575 | SPINK4 | 32/114(28.07) | 1.98 | 2.30E-06 | 1.08E-05 | Yes | Up | - |
| Q9BY27 | DGCR6L | 69/114(60.53) | 0.23 | 1.52E-03 | 3.59E-03 | Yes | Up | - |
| O75474 | FRAT2 | 53/114(46.49) | 0.34 | 1.82E-04 | 5.37E-04 | Yes | Up | - |
| Q8WZ59 | TMEM190 | 45/114(39.47) | 1.09 | 1.65E-04 | 4.93E-04 | Yes | Up | - |
| P31314 | TLX1 | 53/114(46.49) | 2.22 | 1.92E-13 | 8.18E-12 | Yes | Up | - |
| P16444 | DPEP1 | 65/114(57.02) | 1.67 | 1.06E-15 | 1.15E-13 | Yes | Up | - |
| Q15544 | TAF11 | 65/114(57.02) | 0.17 | 8.07E-04 | 2.04E-03 | Yes | Up | - |
| P19429 | TNNI3 | 54/114(47.37) | 0.72 | 1.57E-02 | 2.90E-02 | Yes | Up | - |
| Q8NGT2 | OR13J1 | 60/114(52.63) | 0.87 | 8.84E-04 | 2.21E-03 | Yes | Up | - |
| P09132 | SRP19 | 60/114(52.63) | 0.19 | 1.79E-04 | 5.30E-04 | Yes | Up | - |
| Q9UHD8 | Sep-09 | 59/114(51.75) | 0.37 | 8.49E-08 | 5.82E-07 | Yes | Up | - |
| P56182 | RRP1 | 63/114(55.26) | 0.33 | 2.01E-05 | 7.49E-05 | Yes | Up | - |
| Q6UWX4 | HHIPL2 | 59/114(51.75) | 1.13 | 1.94E-07 | 1.21E-06 | Yes | Up | - |
| Q8NHQ1 | CEP70 | 60/114(52.63) | 0.43 | 1.14E-07 | 7.46E-07 | Yes | Up | - |
| Q14576 | ELAVL3 | 44/114(38.6) | 0.71 | 1.83E-02 | 3.32E-02 | Yes | Up | - |
| Q8IZT8 | HS3ST5 | 46/114(40.35) | 0.97 | 6.60E-05 | 2.17E-04 | Yes | Up | - |
| Q9H9H5 | MAP6D1 | 61/114(53.51) | 0.5 | 2.70E-05 | 9.74E-05 | Yes | Up | - |
| Q9H8S5 | CNTD2 | 64/114(56.14) | 1.47 | 5.73E-14 | 3.05E-12 | Yes | Up | - |
| Q969W1 | ZDHHC16 | 66/114(57.89) | 0.44 | 5.43E-09 | 5.19E-08 | Yes | Up | - |
| Q15717 | ELAVL1 | 61/114(53.51) | 0.29 | 4.84E-08 | 3.54E-07 | Yes | Up | - |
| O14782 | KIF3C | 61/114(53.51) | 0.27 | 7.13E-04 | 1.83E-03 | Yes | Up | - |
| Q99640 | PKMYT1 | 62/114(54.39) | 1.83 | 3.08E-14 | 1.87E-12 | Yes | Up | - |
| Q96S95 | CAMK2N2 | 59/114(51.75) | 1.18 | 8.84E-10 | 1.07E-08 | Yes | Up | - |
| P0CAT3 | TLX1NB | 32/114(28.07) | 2.13 | 3.50E-08 | 2.67E-07 | Yes | Up | - |
| O43272 | PRODH | 63/114(55.26) | 0.49 | 1.16E-02 | 2.21E-02 | Yes | Up | - |
| P49736 | MCM2 | 58/114(50.88) | 0.86 | 1.33E-10 | 2.09E-09 | Yes | Up | - |
| Q86YW9 | MED12L | 49/114(42.98) | 0.44 | 6.16E-03 | 1.26E-02 | Yes | Up | - |
| Q9NZ20 | PLA2G3 | 56/114(49.12) | 0.73 | 6.45E-03 | 1.31E-02 | Yes | Up | - |
| Q15256 | PTPRR | 62/114(54.39) | 0.37 | 5.01E-03 | 1.05E-02 | Yes | Up | - |
| Q96RQ9 | IL4I1 | 62/114(54.39) | 1.23 | 7.12E-15 | 5.59E-13 | Yes | Up | - |
| Q68E01 | INTS3 | 58/114(50.88) | 0.13 | 1.30E-02 | 2.45E-02 | Yes | Up | - |
| Q5JTH9 | RRP12 | 59/114(51.75) | 0.41 | 1.23E-06 | 6.14E-06 | Yes | Up | - |
| Q6NV74 | C2ORF55 | 65/114(57.02) | 0.27 | 4.50E-03 | 9.51E-03 | Yes | Up | - |
| Q53FC8 | ORC5L | 56/114(49.12) | 0.18 | 7.88E-03 | 1.57E-02 | Yes | Up | - |
| Q13641 | TPBG | 55/114(48.25) | 0.64 | 2.19E-09 | 2.34E-08 | Yes | Up | - |
| Q16854 | DGUOK | 57/114(50.0) | 0.18 | 1.18E-03 | 2.86E-03 | Yes | Up | - |
| P07093 | SERPINE2 | 57/114(50.0) | 0.37 | 1.83E-03 | 4.24E-03 | Yes | Up | - |
| Q9NYQ6 | CELSR1 | 58/114(50.88) | 0.87 | 8.12E-08 | 5.60E-07 | Yes | Up | - |
| O95568 | C1ORF156 | 50/114(43.86) | 0.38 | 1.17E-07 | 7.69E-07 | Yes | Up | - |
| P07477 | PRSS1 | 40/114(35.09) | 2.76 | 5.27E-16 | 6.65E-14 | Yes | Up | - |
| P13686 | ACP5 | 61/114(53.51) | 0.64 | 1.07E-07 | 7.13E-07 | Yes | Up | - |
| O75367 | H2AFY | 60/114(52.63) | 0.56 | 3.65E-10 | 4.99E-09 | Yes | Up | - |
| O75689 | ADAP1 | 64/114(56.14) | 0.51 | 2.29E-05 | 8.41E-05 | Yes | Up | - |
| P52630 | STAT2 | 66/114(57.89) | 0.11 | 9.51E-03 | 1.85E-02 | Yes | Up | - |
| Q9P0X4 | CACNA1I | 66/114(57.89) | 0.67 | 2.36E-04 | 6.78E-04 | Yes | Up | - |
| Q8N9S7 | CXORF59 | 44/114(38.6) | 1.17 | 2.43E-04 | 6.96E-04 | Yes | Up | - |
| Q9BVV8 | C19ORF24 | 65/114(57.02) | 0.5 | 1.04E-09 | 1.23E-08 | Yes | Up | - |
| Q96NR8 | RDH12 | 59/114(51.75) | 0.73 | 3.60E-05 | 1.26E-04 | Yes | Up | - |
| Q9Y3Y2 | C1ORF77 | 61/114(53.51) | 0.22 | 4.13E-05 | 1.43E-04 | Yes | Up | - |
| - | NBPF22P | 55/114(48.25) | 1.24 | 5.38E-06 | 2.29E-05 | Yes | Up | - |
| Q96CW9 | NTNG2 | 61/114(53.51) | 0.5 | 2.33E-04 | 6.69E-04 | Yes | Up | - |
| P48067 | SLC6A9 | 65/114(57.02) | 0.87 | 8.66E-11 | 1.44E-09 | Yes | Up | - |
| Q9Y6W8 | ICOS | 60/114(52.63) | 0.8 | 1.59E-06 | 7.72E-06 | Yes | Up | - |
| P62341 | SELT | 59/114(51.75) | 0.26 | 1.25E-04 | 3.84E-04 | Yes | Up | - |
| Q96RP9 | GFM1 | 57/114(50.0) | 0.14 | 1.99E-02 | 3.58E-02 | Yes | Up | - |
| Q13867 | BLMH | 63/114(55.26) | 0.22 | 6.18E-03 | 1.26E-02 | Yes | Up | - |
| Q5JU85 | IQSEC2 | 62/114(54.39) | 0.19 | 2.11E-03 | 4.82E-03 | Yes | Up | - |
| Q6P1K2 | PMF1 | 62/114(54.39) | 0.39 | 7.24E-10 | 8.98E-09 | Yes | Up | - |
| Q16512 | PKN1 | 58/114(50.88) | 0.36 | 2.41E-05 | 8.80E-05 | Yes | Up | - |
| Q9NW97 | TMEM51 | 64/114(56.14) | 0.49 | 5.23E-08 | 3.79E-07 | Yes | Up | - |
| Q9NUM3 | SLC39A9 | 55/114(48.25) | 0.22 | 4.09E-04 | 1.11E-03 | Yes | Up | - |
| O00182 | LGALS9 | 60/114(52.63) | 0.44 | 1.78E-05 | 6.74E-05 | Yes | Up | - |
| A4GXA9 | EME2 | 60/114(52.63) | 0.62 | 4.13E-05 | 1.43E-04 | Yes | Up | - |
| P48729 | CSNK1A1 | 66/114(57.89) | 0.13 | 2.09E-02 | 3.74E-02 | Yes | Up | - |
| O00233 | PSMD9 | 60/114(52.63) | 0.18 | 1.87E-04 | 5.49E-04 | Yes | Up | - |
| P22626 | HNRNPA2B1 | 59/114(51.75) | 0.29 | 2.73E-07 | 1.63E-06 | Yes | Up | - |
| O75949 | FAM155B | 59/114(51.75) | 0.44 | 1.05E-02 | 2.02E-02 | Yes | Up | - |
| Q86XT2 | VPS37D | 67/114(58.77) | 0.52 | 9.19E-07 | 4.77E-06 | Yes | Up | - |
| P50990 | CCT8 | 60/114(52.63) | 0.21 | 1.17E-03 | 2.84E-03 | Yes | Up | - |
| Q9NUQ8 | ABCF3 | 53/114(46.49) | 0.33 | 6.44E-09 | 6.04E-08 | Yes | Up | - |
| Q9H2C2 | ARV1 | 55/114(48.25) | 0.41 | 1.46E-06 | 7.20E-06 | Yes | Up | - |
| Q96B70 | LENG9 | 65/114(57.02) | 0.2 | 6.43E-03 | 1.31E-02 | Yes | Up | - |
| Q8NE71 | ABCF1 | 63/114(55.26) | 0.14 | 1.13E-02 | 2.16E-02 | Yes | Up | - |
| Q6PCB6 | FAM108C1 | 61/114(53.51) | 0.63 | 1.49E-06 | 7.33E-06 | Yes | Up | - |
| P12004 | PCNA | 60/114(52.63) | 0.63 | 3.44E-09 | 3.50E-08 | Yes | Up | - |
| Q9Y286 | SIGLEC7 | 63/114(55.26) | 0.71 | 1.05E-08 | 9.25E-08 | Yes | Up | - |
| O15342 | ATP6V0E1 | 58/114(50.88) | 0.17 | 1.44E-02 | 2.69E-02 | Yes | Up | - |
| O75382 | TRIM3 | 56/114(49.12) | 0.34 | 4.67E-04 | 1.25E-03 | Yes | Up | - |
| P22891 | PROZ | 57/114(50.0) | 0.88 | 2.31E-05 | 8.49E-05 | Yes | Up | - |
| O43678 | NDUFA2 | 67/114(58.77) | 0.25 | 6.84E-04 | 1.76E-03 | Yes | Up | - |
| P0C671 | C6ORF222 | 40/114(35.09) | 1.48 | 5.37E-06 | 2.29E-05 | Yes | Up | - |
| Q96PD5 | PGLYRP2 | 57/114(50.0) | 0.91 | 2.83E-04 | 7.95E-04 | Yes | Up | - |
| P07339 | CTSD | 63/114(55.26) | 0.69 | 6.73E-09 | 6.26E-08 | Yes | Up | - |
| Q96CN4 | EVI5L | 66/114(57.89) | 0.2 | 1.80E-03 | 4.19E-03 | Yes | Up | - |
| Q9H267 | VPS33B | 51/114(44.74) | 0.13 | 2.02E-03 | 4.64E-03 | Yes | Up | - |
| Q6SPF0 | SAMD1 | 59/114(51.75) | 0.55 | 5.37E-11 | 9.60E-10 | Yes | Up | - |
| P11215 | ITGAM | 57/114(50.0) | 0.31 | 8.82E-03 | 1.73E-02 | Yes | Up | - |
| Q8WYA6 | CTNNBL1 | 60/114(52.63) | 0.18 | 3.62E-04 | 9.95E-04 | Yes | Up | - |
| Q9H2Y9 | SLCO5A1 | 65/114(57.02) | 0.73 | 3.09E-04 | 8.62E-04 | Yes | Up | - |
| Q9H5Q4 | TFB2M | 56/114(49.12) | 0.42 | 1.25E-06 | 6.25E-06 | Yes | Up | - |
| Q01081 | U2AF1 | 60/114(52.63) | 0.11 | 2.34E-02 | 4.14E-02 | Yes | Up | - |
| A2A288 | ZC3H12D | 63/114(55.26) | 0.36 | 1.24E-02 | 2.35E-02 | Yes | Up | - |
| P34897 | SHMT2 | 64/114(56.14) | 0.45 | 1.51E-07 | 9.64E-07 | Yes | Up | - |
| Q9BW27 | NUP85 | 52/114(45.61) | 0.24 | 9.16E-05 | 2.91E-04 | Yes | Up | - |
| Q8TD10 | MIPOL1 | 59/114(51.75) | 0.4 | 2.90E-05 | 1.04E-04 | Yes | Up | - |
| Q9NVL1 | FAM86C | 57/114(50.0) | 0.35 | 8.34E-06 | 3.41E-05 | Yes | Up | - |
| Q9P035 | PTPLAD1 | 52/114(45.61) | 0.42 | 1.77E-05 | 6.70E-05 | Yes | Up | - |
| Q14997 | PSME4 | 61/114(53.51) | 0.18 | 2.66E-02 | 4.64E-02 | Yes | Up | - |
| Q6PD74 | AAGAB | 61/114(53.51) | 0.44 | 3.89E-07 | 2.23E-06 | Yes | Up | - |
| Q8IY21 | DDX60 | 58/114(50.88) | 0.31 | 2.27E-03 | 5.15E-03 | Yes | Up | - |
| Q96CD2 | PPCDC | 58/114(50.88) | 0.27 | 4.74E-06 | 2.05E-05 | Yes | Up | - |
| Q9HCU5 | PREB | 58/114(50.88) | 0.13 | 1.76E-02 | 3.22E-02 | Yes | Up | - |
| P02647 | APOA1 | 58/114(50.88) | 0.72 | 1.21E-03 | 2.94E-03 | Yes | Up | - |
| P09912 | IFI6 | 62/114(54.39) | 1.27 | 8.58E-13 | 2.82E-11 | Yes | Up | - |
| Q7L2R6 | ZNF765 | 54/114(47.37) | 0.2 | 6.16E-03 | 1.26E-02 | Yes | Up | - |
| Q96JB1 | DNAH8 | 59/114(51.75) | 0.66 | 3.22E-03 | 7.02E-03 | Yes | Up | - |
| Q9BXJ4 | C1QTNF3 | 57/114(50.0) | 0.74 | 2.47E-06 | 1.15E-05 | Yes | Up | - |
| Q96SK2 | TMEM209 | 61/114(53.51) | 0.27 | 3.16E-04 | 8.80E-04 | Yes | Up | - |
| P30837 | ALDH1B1 | 60/114(52.63) | 0.47 | 2.57E-07 | 1.54E-06 | Yes | Up | - |
| Q96F05 | C11ORF24 | 56/114(49.12) | 0.43 | 1.83E-07 | 1.14E-06 | Yes | Up | - |
| P07305 | H1F0 | 61/114(53.51) | 0.4 | 2.92E-06 | 1.33E-05 | Yes | Up | - |
| Q96AP4 | ZUFSP | 55/114(48.25) | 0.15 | 9.94E-03 | 1.93E-02 | Yes | Up | - |
| Q8TE77 | SSH3 | 68/114(59.65) | 0.39 | 5.08E-07 | 2.83E-06 | Yes | Up | - |
| Q9BRY0 | SLC39A3 | 61/114(53.51) | 0.38 | 9.31E-09 | 8.34E-08 | Yes | Up | - |
| P54278 | PMS2 | 51/114(44.74) | 0.15 | 3.62E-03 | 7.79E-03 | Yes | Up | - |
| Q9H1N7 | SLC35B3 | 57/114(50.0) | 0.11 | 2.88E-02 | 4.98E-02 | Yes | Up | - |
| P11245 | NAT2 | 57/114(50.0) | 0.5 | 1.89E-02 | 3.42E-02 | Yes | Up | - |
| Q9HAS0 | C17ORF75 | 53/114(46.49) | 0.19 | 9.10E-03 | 1.78E-02 | Yes | Up | - |
| P25205 | MCM3 | 61/114(53.51) | 0.36 | 9.28E-07 | 4.81E-06 | Yes | Up | - |
| P78362 | SRPK2 | 52/114(45.61) | 0.27 | 9.17E-05 | 2.91E-04 | Yes | Up | - |
| Q06210 | GFPT1 | 54/114(47.37) | 0.17 | 2.35E-02 | 4.15E-02 | Yes | Up | - |
| Q969V3 | NCLN | 59/114(51.75) | 0.31 | 9.92E-06 | 3.99E-05 | Yes | Up | - |
| Q96GQ7 | DDX27 | 64/114(56.14) | 0.27 | 1.02E-04 | 3.21E-04 | Yes | Up | - |
| Q9UJU5 | FOXD3 | 63/114(55.26) | 1.2 | 2.26E-06 | 1.06E-05 | Yes | Up | - |
| Q06055 | ATP5G2 | 58/114(50.88) | 0.21 | 5.85E-04 | 1.53E-03 | Yes | Up | - |
| P13637 | ATP1A3 | 60/114(52.63) | 1.17 | 2.60E-07 | 1.56E-06 | Yes | Up | - |
| Q96HJ3 | CCDC34 | 54/114(47.37) | 0.17 | 2.37E-02 | 4.19E-02 | Yes | Up | - |
| Q5UE93 | PIK3R6 | 65/114(57.02) | 0.49 | 1.85E-05 | 6.96E-05 | Yes | Up | - |
| Q04917 | YWHAH | 65/114(57.02) | 0.34 | 4.87E-07 | 2.72E-06 | Yes | Up | - |
| P59894 | DCDC1 | 55/114(48.25) | 0.85 | 9.31E-05 | 2.95E-04 | Yes | Up | - |
| Q9P2H5 | USP35 | 55/114(48.25) | 0.24 | 7.46E-03 | 1.49E-02 | Yes | Up | - |
| Q6ZTN6 | ANKRD13D | 60/114(52.63) | 0.4 | 1.58E-08 | 1.33E-07 | Yes | Up | - |
| Q9H2H9 | SLC38A1 | 58/114(50.88) | 0.5 | 5.79E-04 | 1.51E-03 | Yes | Up | - |
| Q9UK80 | USP21 | 61/114(53.51) | 0.37 | 4.68E-08 | 3.44E-07 | Yes | Up | - |
| P35232 | PHB | 58/114(50.88) | 0.17 | 1.55E-02 | 2.86E-02 | Yes | Up | - |
| P07741 | APRT | 68/114(59.65) | 0.24 | 8.99E-04 | 2.25E-03 | Yes | Up | - |
| P50238 | CRIP1 | 62/114(54.39) | 1.34 | 2.78E-15 | 2.63E-13 | Yes | Up | - |
| Q96DH6 | MSI2 | 56/114(49.12) | 0.62 | 1.01E-06 | 5.17E-06 | Yes | Up | - |
| Q0VGL1 | C7ORF59 | 61/114(53.51) | 0.24 | 1.91E-04 | 5.62E-04 | Yes | Up | - |
| Q9C005 | DPY30 | 65/114(57.02) | 0.36 | 2.11E-06 | 9.99E-06 | Yes | Up | - |
| P62195 | PSMC5 | 63/114(55.26) | 0.27 | 4.57E-05 | 1.56E-04 | Yes | Up | - |
| Q96EH5 | RPL39L | 66/114(57.89) | 0.61 | 6.35E-08 | 4.50E-07 | Yes | Up | - |
| Q9BUH6 | C9ORF142 | 65/114(57.02) | 0.57 | 2.59E-10 | 3.69E-09 | Yes | Up | - |
| Q96G04 | FAM86A | 65/114(57.02) | 0.39 | 2.78E-08 | 2.18E-07 | Yes | Up | - |
| P78337 | PITX1 | 61/114(53.51) | 1.97 | 1.06E-15 | 1.15E-13 | Yes | Up | - |
| Q16559 | TAL2 | 57/114(50.0) | 0.6 | 2.22E-03 | 5.04E-03 | Yes | Up | - |
| P63104 | YWHAZ | 57/114(50.0) | 0.33 | 2.93E-04 | 8.22E-04 | Yes | Up | - |
| O95154 | AKR7A3 | 57/114(50.0) | 1.29 | 1.57E-09 | 1.77E-08 | Yes | Up | - |
| Q8IYS2 | KIAA2013 | 61/114(53.51) | 0.2 | 4.72E-04 | 1.26E-03 | Yes | Up | - |
| A0AVF1 | TTC26 | 55/114(48.25) | 0.42 | 2.30E-05 | 8.43E-05 | Yes | Up | - |
| Q96FV3 | TSPAN17 | 66/114(57.89) | 0.37 | 8.35E-06 | 3.41E-05 | Yes | Up | - |
| P31151 | S100A7 | 58/114(50.88) | 2.51 | 1.51E-14 | 1.04E-12 | Yes | Up | - |
| O95267 | RASGRP1 | 67/114(58.77) | 0.8 | 1.20E-06 | 6.03E-06 | Yes | Up | - |
| Q08380 | LGALS3BP | 56/114(49.12) | 0.25 | 2.51E-02 | 4.40E-02 | Yes | Up | - |
| Q9NVX7 | KBTBD4 | 51/114(44.74) | 0.14 | 1.90E-02 | 3.43E-02 | Yes | Up | - |
| Q9BSC4 | NOL10 | 56/114(49.12) | 0.13 | 1.48E-02 | 2.75E-02 | Yes | Up | - |
| Q96C12 | ARMC5 | 69/114(60.53) | 0.22 | 8.24E-04 | 2.08E-03 | Yes | Up | - |
| Q14527 | HLTF | 59/114(51.75) | 0.19 | 2.04E-02 | 3.66E-02 | Yes | Up | - |
| Q8IV36 | C17ORF28 | 59/114(51.75) | 0.71 | 2.48E-07 | 1.50E-06 | Yes | Up | - |
| Q9NZ01 | TECR | 64/114(56.14) | 0.19 | 5.68E-04 | 1.49E-03 | Yes | Up | - |
| Q86TS9 | MRPL52 | 63/114(55.26) | 0.29 | 4.90E-06 | 2.11E-05 | Yes | Up | - |
| O95996 | APC2 | 48/114(42.11) | 0.38 | 5.98E-03 | 1.23E-02 | Yes | Up | - |
| Q13190 | STX5 | 61/114(53.51) | 0.11 | 5.97E-03 | 1.23E-02 | Yes | Up | - |
| Q00536 | CDK16 | 62/114(54.39) | 0.22 | 3.72E-03 | 7.98E-03 | Yes | Up | - |
| P61024 | CKS1B | 61/114(53.51) | 0.34 | 5.41E-04 | 1.43E-03 | Yes | Up | - |
| A0JLT2 | MED19 | 65/114(57.02) | 0.31 | 1.90E-06 | 9.09E-06 | Yes | Up | - |
| P62136 | PPP1CA | 65/114(57.02) | 0.57 | 3.92E-11 | 7.39E-10 | Yes | Up | - |
| Q96CS7 | PLEKHB2 | 57/114(50.0) | 0.2 | 2.79E-03 | 6.16E-03 | Yes | Up | - |
| Q96PP8 | GBP5 | 61/114(53.51) | 1.06 | 3.17E-10 | 4.39E-09 | Yes | Up | - |
| Q99496 | RNF2 | 47/114(41.23) | 0.22 | 6.20E-03 | 1.27E-02 | Yes | Up | - |
| Q9BUW7 | C9ORF16 | 58/114(50.88) | 0.23 | 4.93E-03 | 1.03E-02 | Yes | Up | - |
| A1A4V9 | C16ORF93 | 62/114(54.39) | 0.81 | 8.35E-09 | 7.58E-08 | Yes | Up | - |
| Q9Y5U9 | IER3IP1 | 63/114(55.26) | 0.32 | 5.24E-06 | 2.24E-05 | Yes | Up | - |
| Q6UX98 | ZDHHC24 | 59/114(51.75) | 0.52 | 3.82E-10 | 5.19E-09 | Yes | Up | - |
| Q16777 | HIST2H2AC | 61/114(53.51) | 0.74 | 1.39E-06 | 6.87E-06 | Yes | Up | - |
| O95396 | MOCS3 | 60/114(52.63) | 0.3 | 6.67E-05 | 2.19E-04 | Yes | Up | - |
| P08118 | MSMB | 57/114(50.0) | 2.38 | 6.08E-21 | 4.35E-18 | Yes | Up | - |
| Q9NRG9 | AAAS | 65/114(57.02) | 0.14 | 6.33E-04 | 1.64E-03 | Yes | Up | - |
| Q96QT6 | PHF12 | 53/114(46.49) | 0.2 | 9.58E-04 | 2.38E-03 | Yes | Up | - |
| P51685 | CCR8 | 63/114(55.26) | 1.53 | 2.61E-10 | 3.70E-09 | Yes | Up | - |
| O15212 | PFDN6 | 66/114(57.89) | 0.5 | 4.07E-09 | 4.05E-08 | Yes | Up | - |
| Q8IX29 | FBXO16 | 62/114(54.39) | 1.02 | 5.78E-09 | 5.50E-08 | Yes | Up | - |
| P52298 | NCBP2 | 54/114(47.37) | 0.16 | 1.86E-03 | 4.31E-03 | Yes | Up | - |
| Q9H609 | ZNF576 | 65/114(57.02) | 0.1 | 2.14E-02 | 3.82E-02 | Yes | Up | - |
| Q14CW9 | ATXN7L3 | 58/114(50.88) | 0.26 | 1.09E-07 | 7.25E-07 | Yes | Up | - |
| Q5JWF8 | C20ORF134 | 64/114(56.14) | 0.65 | 6.96E-08 | 4.88E-07 | Yes | Up | - |
| Q6IPX3 | TCEAL6 | 65/114(57.02) | 0.45 | 5.50E-05 | 1.84E-04 | Yes | Up | - |
| - | GEMIN8P4 | 60/114(52.63) | 0.32 | 9.19E-04 | 2.29E-03 | Yes | Up | - |
| Q9NVI1 | FANCI | 60/114(52.63) | 0.99 | 4.02E-12 | 1.05E-10 | Yes | Up | - |
| Q5VYY1 | ANKRD22 | 64/114(56.14) | 0.95 | 3.83E-07 | 2.20E-06 | Yes | Up | - |
| O75446 | SAP30 | 62/114(54.39) | 0.4 | 3.83E-05 | 1.33E-04 | Yes | Up | - |
| P14678 | SNRPB | 63/114(55.26) | 0.47 | 8.17E-08 | 5.62E-07 | Yes | Up | - |
| Q9BXS9 | SLC26A6 | 58/114(50.88) | 0.47 | 5.11E-06 | 2.19E-05 | Yes | Up | - |
| Q13352 | ITGB3BP | 66/114(57.89) | 0.17 | 7.71E-03 | 1.54E-02 | Yes | Up | - |
| O15304 | SIVA1 | 73/114(64.04) | 0.22 | 1.96E-03 | 4.53E-03 | Yes | Up | - |
| A8C4L5 | C20ORF20 | 62/114(54.39) | 0.5 | 1.64E-10 | 2.50E-09 | Yes | Up | - |
| O95684 | FGFR1OP | 54/114(47.37) | 0.29 | 8.61E-04 | 2.16E-03 | Yes | Up | - |
| Q68CZ1 | RPGRIP1L | 59/114(51.75) | 0.21 | 1.65E-02 | 3.03E-02 | Yes | Up | - |
| Q86TN4 | TRPT1 | 59/114(51.75) | 0.19 | 6.54E-03 | 1.33E-02 | Yes | Up | - |
| Q494U1 | PLEKHN1 | 65/114(57.02) | 0.9 | 3.40E-08 | 2.60E-07 | Yes | Up | - |
| Q13474 | DRP2 | 61/114(53.51) | 1.17 | 1.33E-09 | 1.53E-08 | Yes | Up | - |
| Q86YR5 | GPSM1 | 68/114(59.65) | 0.31 | 5.13E-04 | 1.36E-03 | Yes | Up | - |
| P11908 | PRPS2 | 55/114(48.25) | 0.34 | 7.52E-05 | 2.43E-04 | Yes | Up | - |
| Q9Y275 | TNFSF13B | 60/114(52.63) | 0.56 | 1.06E-05 | 4.23E-05 | Yes | Up | - |
| Q9BW71 | HIRIP3 | 64/114(56.14) | 0.31 | 6.46E-05 | 2.13E-04 | Yes | Up | - |
| Q8WV37 | ZNF480 | 51/114(44.74) | 0.33 | 4.92E-05 | 1.67E-04 | Yes | Up | - |
| Q11206 | ST3GAL4 | 56/114(49.12) | 0.38 | 2.64E-04 | 7.50E-04 | Yes | Up | - |
| Q86VP6 | CAND1 | 54/114(47.37) | 0.27 | 6.12E-05 | 2.03E-04 | Yes | Up | - |
| Q9BTC8 | MTA3 | 60/114(52.63) | 0.23 | 7.46E-05 | 2.42E-04 | Yes | Up | - |
| P30550 | GRPR | 59/114(51.75) | 0.85 | 2.80E-04 | 7.87E-04 | Yes | Up | - |
| Q96M69 | LRGUK | 68/114(59.65) | 0.56 | 5.64E-04 | 1.48E-03 | Yes | Up | - |
| P28799 | GRN | 67/114(58.77) | 0.43 | 1.63E-08 | 1.37E-07 | Yes | Up | - |
| Q8TF05 | PPP4R1 | 59/114(51.75) | 0.14 | 3.95E-03 | 8.44E-03 | Yes | Up | - |
| Q53ET0 | CRTC2 | 56/114(49.12) | 0.23 | 2.27E-05 | 8.36E-05 | Yes | Up | - |
| Q6UXP7 | FAM151B | 55/114(48.25) | 0.29 | 1.75E-03 | 4.09E-03 | Yes | Up | - |
| Q5M8T2 | SLC35D3 | 58/114(50.88) | 0.89 | 1.50E-03 | 3.56E-03 | Yes | Up | - |
| P24666 | ACP1 | 59/114(51.75) | 0.21 | 1.01E-03 | 2.48E-03 | Yes | Up | - |
| P54317 | PNLIPRP2 | 41/114(35.96) | 1.41 | 4.92E-06 | 2.12E-05 | Yes | Up | - |
| A6NKF1 | SAC3D1 | 60/114(52.63) | 0.72 | 8.13E-11 | 1.37E-09 | Yes | Up | - |
| Q9BRP1 | PDCD2L | 63/114(55.26) | 0.2 | 1.56E-02 | 2.88E-02 | Yes | Up | - |
| Q659C4 | LARP1B | 52/114(45.61) | 0.18 | 9.87E-03 | 1.92E-02 | Yes | Up | - |
| P15863 | PAX1 | 63/114(55.26) | 1.13 | 2.05E-05 | 7.63E-05 | Yes | Up | - |
| Q8NCX0 | CCDC150 | 62/114(54.39) | 0.55 | 8.41E-05 | 2.69E-04 | Yes | Up | - |
| Q9NQR7 | C14ORF162 | 54/114(47.37) | 0.47 | 1.66E-02 | 3.05E-02 | Yes | Up | - |
| Q9Y6D9 | MAD1L1 | 70/114(61.4) | 0.21 | 2.79E-03 | 6.17E-03 | Yes | Up | - |
| Q14160 | SCRIB | 65/114(57.02) | 0.36 | 2.25E-04 | 6.48E-04 | Yes | Up | - |
| Q96G42 | KLHDC7B | 67/114(58.77) | 1.03 | 1.38E-09 | 1.58E-08 | Yes | Up | - |
| Q9NYA1 | SPHK1 | 67/114(58.77) | 0.38 | 4.43E-04 | 1.19E-03 | Yes | Up | - |
| Q9NUQ9 | FAM49B | 62/114(54.39) | 0.41 | 1.51E-05 | 5.81E-05 | Yes | Up | - |
| Q92730 | RND1 | 64/114(56.14) | 1.12 | 5.72E-12 | 1.43E-10 | Yes | Up | - |
| Q8N4T0 | CPA6 | 54/114(47.37) | 1.63 | 2.12E-12 | 6.09E-11 | Yes | Up | - |
| Q9NU39 | FOXD4L1 | 62/114(54.39) | 0.75 | 1.13E-07 | 7.44E-07 | Yes | Up | - |
| Q71RC9 | LOC643008 | 63/114(55.26) | 0.37 | 8.20E-03 | 1.63E-02 | Yes | Up | - |
| P55795 | HNRNPH2 | 57/114(50.0) | 0.11 | 2.37E-02 | 4.18E-02 | Yes | Up | - |
| Q9Y3B6 | FAM158A | 64/114(56.14) | 0.53 | 4.01E-08 | 3.00E-07 | Yes | Up | - |
| Q8TE68 | EPS8L1 | 68/114(59.65) | 0.78 | 2.18E-06 | 1.03E-05 | Yes | Up | - |
| Q8IUL8 | CILP2 | 60/114(52.63) | 2.19 | 1.65E-28 | 3.20E-24 | Yes | Up | - |
| Q96A26 | FAM162A | 62/114(54.39) | 0.4 | 9.71E-07 | 5.01E-06 | Yes | Up | - |
| Q9NP97 | DYNLRB1 | 66/114(57.89) | 0.23 | 7.15E-04 | 1.83E-03 | Yes | Up | - |
| Q6P4I2 | WDR73 | 60/114(52.63) | 0.14 | 1.34E-02 | 2.51E-02 | Yes | Up | - |
| O96011 | PEX11B | 61/114(53.51) | 0.41 | 1.06E-08 | 9.36E-08 | Yes | Up | - |
| P46013 | MKI67 | 55/114(48.25) | 1.5 | 1.59E-12 | 4.76E-11 | Yes | Up | - |
| Q9NQ11 | ATP13A2 | 64/114(56.14) | 0.48 | 9.05E-08 | 6.14E-07 | Yes | Up | - |
| Q5JSP0 | FGD3 | 67/114(58.77) | 0.99 | 1.07E-11 | 2.46E-10 | Yes | Up | - |
| O43670 | ZNF207 | 58/114(50.88) | 0.11 | 4.91E-03 | 1.03E-02 | Yes | Up | - |
| - | EPR1 | 58/114(50.88) | 1.69 | 2.83E-13 | 1.13E-11 | Yes | Up | - |
| P20700 | LMNB1 | 57/114(50.0) | 1.25 | 2.11E-13 | 8.80E-12 | Yes | Up | - |
| Q53S58 | TMEM177 | 60/114(52.63) | 0.24 | 9.63E-04 | 2.39E-03 | Yes | Up | - |
| O95298 | NDUFC2 | 62/114(54.39) | 0.19 | 2.50E-02 | 4.38E-02 | Yes | Up | - |
| Q9NVH2 | INTS7 | 56/114(49.12) | 0.72 | 2.13E-11 | 4.40E-10 | Yes | Up | - |
| Q8IYL3 | C1ORF174 | 59/114(51.75) | 0.11 | 7.66E-03 | 1.53E-02 | Yes | Up | - |
| Q6ZT07 | TBC1D9 | 51/114(44.74) | 0.56 | 5.37E-05 | 1.80E-04 | Yes | Up | - |
| Q9UH17 | APOBEC3B | 62/114(54.39) | 1.23 | 1.49E-11 | 3.21E-10 | Yes | Up | - |
| Q8WUA4 | GTF3C2 | 50/114(43.86) | 0.21 | 2.29E-04 | 6.59E-04 | Yes | Up | - |
| Q96G03 | PGM2 | 57/114(50.0) | 0.19 | 2.81E-03 | 6.21E-03 | Yes | Up | - |
| Q6P9B9 | INTS5 | 63/114(55.26) | 0.21 | 4.24E-06 | 1.85E-05 | Yes | Up | - |
| Q8NC56 | LEMD2 | 67/114(58.77) | 0.23 | 3.27E-05 | 1.16E-04 | Yes | Up | - |
| Q8IWU5 | SULF2 | 65/114(57.02) | 0.56 | 1.90E-06 | 9.07E-06 | Yes | Up | - |
| B3KQX0 | ZFP64 | 52/114(45.61) | 0.31 | 5.59E-08 | 4.01E-07 | Yes | Up | - |
| Q9UHC7 | MKRN1 | 60/114(52.63) | 0.14 | 3.36E-03 | 7.28E-03 | Yes | Up | - |
| Q15003 | NCAPH | 56/114(49.12) | 1.41 | 1.49E-13 | 6.69E-12 | Yes | Up | - |
| Q8WXU2 | DYX1C1 | 58/114(50.88) | 0.5 | 1.45E-05 | 5.61E-05 | Yes | Up | - |
| P51693 | APLP1 | 60/114(52.63) | 0.66 | 5.51E-05 | 1.84E-04 | Yes | Up | - |
| Q6PII5 | HAGHL | 60/114(52.63) | 2.01 | 1.30E-21 | 1.19E-18 | Yes | Up | - |
| P52569 | SLC7A2 | 60/114(52.63) | 0.45 | 1.66E-02 | 3.05E-02 | Yes | Up | - |
| Q96J65 | ABCC12 | 53/114(46.49) | 0.77 | 9.96E-03 | 1.93E-02 | Yes | Up | - |
| Q03393 | PTS | 64/114(56.14) | 0.36 | 9.54E-06 | 3.85E-05 | Yes | Up | - |
| O15015 | ZNF646 | 55/114(48.25) | 0.24 | 1.07E-04 | 3.33E-04 | Yes | Up | - |
| Q9H1Z9 | TSPAN10 | 59/114(51.75) | 0.46 | 6.56E-04 | 1.70E-03 | Yes | Up | - |
| Q96BA8 | CREB3L1 | 56/114(49.12) | 1.21 | 2.87E-12 | 7.91E-11 | Yes | Up | - |
| P23771 | GATA3 | 52/114(45.61) | 1.05 | 2.64E-08 | 2.09E-07 | Yes | Up | - |
| O60243 | HS6ST1 | 65/114(57.02) | 0.33 | 1.10E-05 | 4.38E-05 | Yes | Up | - |
| Q32P28 | LEPRE1 | 58/114(50.88) | 0.42 | 9.23E-07 | 4.78E-06 | Yes | Up | - |
| Q96B77 | TMEM186 | 67/114(58.77) | 0.23 | 1.57E-04 | 4.73E-04 | Yes | Up | - |
| P15924 | DSP | 58/114(50.88) | 0.48 | 1.69E-03 | 3.97E-03 | Yes | Up | - |
| P38398 | BRCA1 | 61/114(53.51) | 0.49 | 2.04E-05 | 7.58E-05 | Yes | Up | - |
| Q96A72 | MAGOHB | 57/114(50.0) | 0.38 | 4.04E-07 | 2.30E-06 | Yes | Up | - |
| Q14554 | PDIA5 | 58/114(50.88) | 0.33 | 5.20E-05 | 1.75E-04 | Yes | Up | - |
| Q8NC24 | RELL2 | 61/114(53.51) | 0.61 | 6.01E-05 | 1.99E-04 | Yes | Up | - |
| O43615 | TIMM44 | 64/114(56.14) | 0.25 | 7.14E-04 | 1.83E-03 | Yes | Up | - |
| Q2NL68 | C19ORF55 | 58/114(50.88) | 0.27 | 1.62E-03 | 3.80E-03 | Yes | Up | - |
| Q9NYQ7 | CELSR3 | 55/114(48.25) | 1.1 | 1.29E-10 | 2.04E-09 | Yes | Up | - |
| O14559 | ARHGAP33 | 59/114(51.75) | 0.33 | 9.09E-04 | 2.27E-03 | Yes | Up | - |
| Q9C0D6 | FHDC1 | 53/114(46.49) | 0.44 | 1.03E-04 | 3.22E-04 | Yes | Up | - |
| Q9NQ34 | TMEM9B | 58/114(50.88) | 0.16 | 8.06E-03 | 1.60E-02 | Yes | Up | - |
| P07384 | CAPN1 | 58/114(50.88) | 0.3 | 2.02E-06 | 9.58E-06 | Yes | Up | - |
| - | C6ORF147 | 58/114(50.88) | 0.38 | 1.83E-02 | 3.32E-02 | Yes | Up | - |
| O43542 | XRCC3 | 60/114(52.63) | 0.23 | 3.00E-03 | 6.57E-03 | Yes | Up | - |
| Q9HBM1 | SPC25 | 60/114(52.63) | 1.42 | 8.88E-12 | 2.09E-10 | Yes | Up | - |
| J3KQD0 | C1ORF93 | 63/114(55.26) | 0.58 | 1.95E-10 | 2.89E-09 | Yes | Up | - |
| Q9H6Y5 | MAGIX | 57/114(50.0) | 0.59 | 8.29E-08 | 5.69E-07 | Yes | Up | - |
| P31645 | SLC6A4 | 56/114(49.12) | 1.64 | 2.16E-12 | 6.19E-11 | Yes | Up | - |
| Q99500 | S1PR3 | 56/114(49.12) | 0.39 | 6.05E-04 | 1.58E-03 | Yes | Up | - |
| Q58A44 | PCOTH | 58/114(50.88) | 0.49 | 1.42E-04 | 4.30E-04 | Yes | Up | - |
| Q96Q89 | KIF20B | 55/114(48.25) | 0.46 | 1.67E-05 | 6.34E-05 | Yes | Up | - |
| P52926 | HMGA2 | 57/114(50.0) | 0.59 | 1.17E-03 | 2.84E-03 | Yes | Up | - |
| Q9H977 | WDR54 | 62/114(54.39) | 0.5 | 1.02E-06 | 5.20E-06 | Yes | Up | - |
| Q04760 | GLO1 | 61/114(53.51) | 0.25 | 8.36E-04 | 2.11E-03 | Yes | Up | - |
| P51884 | LUM | 56/114(49.12) | 0.53 | 3.85E-05 | 1.34E-04 | Yes | Up | - |
| O95257 | GADD45G | 71/114(62.28) | 0.58 | 1.83E-05 | 6.88E-05 | Yes | Up | - |
| Q9NUD7 | C20ORF96 | 60/114(52.63) | 0.25 | 5.08E-03 | 1.06E-02 | Yes | Up | - |
| - | LOC221122 | 35/114(30.7) | 1.66 | 1.13E-05 | 4.48E-05 | Yes | Up | - |
| P06731 | CEACAM5 | 60/114(52.63) | 1.46 | 9.77E-07 | 5.03E-06 | Yes | Up | - |
| Q8TEK3 | DOT1L | 63/114(55.26) | 0.16 | 1.68E-02 | 3.08E-02 | Yes | Up | - |
| P40305 | IFI27 | 66/114(57.89) | 0.64 | 5.22E-05 | 1.76E-04 | Yes | Up | - |
| Q5VIY5 | ZNF468 | 52/114(45.61) | 0.43 | 2.94E-06 | 1.34E-05 | Yes | Up | - |
| P08631 | HCK | 59/114(51.75) | 0.38 | 3.27E-04 | 9.08E-04 | Yes | Up | - |
| Q9P0B6 | C6ORF129 | 63/114(55.26) | 0.93 | 1.78E-13 | 7.69E-12 | Yes | Up | - |
| A2RUU4 | C6ORF127 | 55/114(48.25) | 1.11 | 1.03E-05 | 4.12E-05 | Yes | Up | - |
| Q9BUX1 | CHAC1 | 69/114(60.53) | 0.55 | 1.27E-04 | 3.91E-04 | Yes | Up | - |
| Q9P2W3 | GNG13 | 53/114(46.49) | 2.57 | 1.26E-12 | 3.92E-11 | Yes | Up | - |
| Q9H5P4 | PDZD7 | 57/114(50.0) | 0.39 | 9.02E-03 | 1.77E-02 | Yes | Up | - |
| Q9BRV3 | RAG1AP1 | 58/114(50.88) | 1.1 | 2.65E-16 | 3.85E-14 | Yes | Up | - |
| P50748 | KNTC1 | 62/114(54.39) | 0.57 | 5.34E-08 | 3.86E-07 | Yes | Up | - |
| Q86SX3 | C14ORF80 | 64/114(56.14) | 0.86 | 7.61E-13 | 2.58E-11 | Yes | Up | - |
| Q00978 | IRF9 | 60/114(52.63) | 0.54 | 3.10E-08 | 2.40E-07 | Yes | Up | - |
| O96028 | WHSC1 | 55/114(48.25) | 0.32 | 1.74E-04 | 5.17E-04 | Yes | Up | - |
| P52272 | HNRNPM | 66/114(57.89) | 0.22 | 3.29E-07 | 1.92E-06 | Yes | Up | - |
| Q8NBI6 | C3ORF21 | 58/114(50.88) | 0.35 | 3.33E-07 | 1.94E-06 | Yes | Up | - |
| P49588 | AARS | 59/114(51.75) | 0.16 | 1.92E-02 | 3.46E-02 | Yes | Up | - |
| Q8TDQ1 | CD300LF | 59/114(51.75) | 0.75 | 3.45E-09 | 3.50E-08 | Yes | Up | - |
| Q9UMW8 | USP18 | 61/114(53.51) | 0.44 | 2.73E-04 | 7.72E-04 | Yes | Up | - |
| Q9Y603 | ETV7 | 60/114(52.63) | 0.96 | 1.08E-10 | 1.74E-09 | Yes | Up | - |
| Q15427 | SF3B4 | 59/114(51.75) | 0.52 | 7.82E-11 | 1.32E-09 | Yes | Up | - |
| Q9BXA9 | SALL3 | 40/114(35.09) | 1.23 | 1.38E-04 | 4.21E-04 | Yes | Up | - |
| Q9NWR8 | CCDC109B | 64/114(56.14) | 0.35 | 2.57E-04 | 7.33E-04 | Yes | Up | - |
| Q8NBB4 | ZSCAN1 | 63/114(55.26) | 0.61 | 4.39E-03 | 9.29E-03 | Yes | Up | - |
| Q53S33 | BOLA3 | 54/114(47.37) | 0.21 | 4.62E-03 | 9.72E-03 | Yes | Up | - |
| P14923 | JUP | 57/114(50.0) | 0.45 | 4.26E-07 | 2.42E-06 | Yes | Up | - |
| Q6ZNC8 | MBOAT1 | 60/114(52.63) | 0.37 | 2.69E-04 | 7.62E-04 | Yes | Up | - |
| Q9C091 | GREB1L | 57/114(50.0) | 0.94 | 1.58E-07 | 1.01E-06 | Yes | Up | - |
| P20273 | CD22 | 62/114(54.39) | 0.59 | 2.66E-04 | 7.56E-04 | Yes | Up | - |
| Q9Y2R9 | MRPS7 | 61/114(53.51) | 0.25 | 6.40E-04 | 1.66E-03 | Yes | Up | - |
| Q0VDD7 | C19ORF57 | 63/114(55.26) | 0.31 | 1.41E-02 | 2.63E-02 | Yes | Up | - |
| P57088 | TMEM33 | 56/114(49.12) | 0.25 | 1.71E-04 | 5.08E-04 | Yes | Up | - |
| P18669 | PGAM1 | 64/114(56.14) | 0.21 | 1.59E-03 | 3.74E-03 | Yes | Up | - |
| O15235 | MRPS12 | 68/114(59.65) | 0.56 | 1.81E-09 | 1.99E-08 | Yes | Up | - |
| P54368 | OAZ1 | 62/114(54.39) | 0.14 | 9.16E-03 | 1.80E-02 | Yes | Up | - |
| Q9NQ55 | PPAN | 66/114(57.89) | 0.27 | 2.78E-03 | 6.16E-03 | Yes | Up | - |
| Q14691 | GINS1 | 59/114(51.75) | 1.17 | 5.44E-12 | 1.37E-10 | Yes | Up | - |
| O94776 | MTA2 | 62/114(54.39) | 0.19 | 1.81E-04 | 5.35E-04 | Yes | Up | - |
| Q9HC29 | NOD2 | 61/114(53.51) | 0.68 | 5.29E-06 | 2.26E-05 | Yes | Up | - |
| P51161 | FABP6 | 40/114(35.09) | 1.42 | 1.81E-04 | 5.35E-04 | Yes | Up | - |
| P22570 | FDXR | 64/114(56.14) | 0.62 | 8.43E-08 | 5.78E-07 | Yes | Up | - |
| Q96AQ1 | CCDC74A | 64/114(56.14) | 0.72 | 3.43E-06 | 1.54E-05 | Yes | Up | - |
| P03956 | MMP1 | 61/114(53.51) | 1.32 | 2.33E-05 | 8.55E-05 | Yes | Up | - |
| Q8IY34 | SLC15A3 | 61/114(53.51) | 0.35 | 1.05E-03 | 2.58E-03 | Yes | Up | - |
| P37088 | SCNN1A | 61/114(53.51) | 0.44 | 1.04E-02 | 2.01E-02 | Yes | Up | - |
| Q8N511 | TMEM199 | 62/114(54.39) | 0.33 | 2.53E-07 | 1.53E-06 | Yes | Up | - |
| Q14586 | ZNF267 | 58/114(50.88) | 0.24 | 3.69E-04 | 1.01E-03 | Yes | Up | - |
| Q9NSE2 | CISH | 59/114(51.75) | 0.59 | 2.38E-06 | 1.11E-05 | Yes | Up | - |
| P86791 | C7ORF28A | 54/114(47.37) | 0.27 | 2.67E-05 | 9.65E-05 | Yes | Up | - |
| Q9HCY8 | S100A14 | 65/114(57.02) | 1.28 | 2.05E-09 | 2.21E-08 | Yes | Up | - |
| Q96BN2 | TADA1 | 49/114(42.98) | 0.38 | 1.13E-07 | 7.43E-07 | Yes | Up | - |
| Q15468 | STIL | 60/114(52.63) | 0.99 | 2.99E-10 | 4.18E-09 | Yes | Up | - |
| K7EKQ4 | C19ORF40 | 59/114(51.75) | 0.65 | 3.46E-11 | 6.65E-10 | Yes | Up | - |
| P20827 | EFNA1 | 60/114(52.63) | 0.59 | 4.44E-09 | 4.37E-08 | Yes | Up | - |
| P98174 | FGD1 | 60/114(52.63) | 0.36 | 5.76E-05 | 1.92E-04 | Yes | Up | - |
| Q96BP2 | CHCHD1 | 69/114(60.53) | 0.32 | 6.78E-06 | 2.82E-05 | Yes | Up | - |
| O75818 | RPP40 | 58/114(50.88) | 0.26 | 1.29E-03 | 3.11E-03 | Yes | Up | - |
| O43423 | ANP32C | 55/114(48.25) | 0.34 | 1.49E-02 | 2.77E-02 | Yes | Up | - |
| Q16676 | FOXD1 | 63/114(55.26) | 1.46 | 1.30E-13 | 5.97E-12 | Yes | Up | - |
| P15311 | EZR | 56/114(49.12) | 0.73 | 4.82E-10 | 6.36E-09 | Yes | Up | - |
| Q13547 | HDAC1 | 57/114(50.0) | 0.28 | 4.64E-06 | 2.00E-05 | Yes | Up | - |
| Q96DG6 | CMBL | 55/114(48.25) | 0.41 | 2.27E-03 | 5.15E-03 | Yes | Up | - |
| Q9Y570 | PPME1 | 60/114(52.63) | 0.26 | 1.11E-04 | 3.45E-04 | Yes | Up | - |
| Q15819 | UBE2V2 | 64/114(56.14) | 0.19 | 8.54E-03 | 1.69E-02 | Yes | Up | - |
| Q2WGJ6 | KLHL38 | 58/114(50.88) | 0.88 | 1.07E-06 | 5.44E-06 | Yes | Up | - |
| Q8NDX2 | SLC17A8 | 38/114(33.33) | 0.83 | 1.76E-02 | 3.21E-02 | Yes | Up | - |
| P35637 | FUS | 65/114(57.02) | 0.42 | 1.74E-09 | 1.91E-08 | Yes | Up | - |
| Q9BVK2 | ALG8 | 54/114(47.37) | 0.28 | 3.28E-03 | 7.13E-03 | Yes | Up | - |
| - | MIAT | 61/114(53.51) | 0.48 | 5.67E-05 | 1.89E-04 | Yes | Up | - |
| P30989 | NTSR1 | 63/114(55.26) | 1.5 | 2.83E-10 | 3.98E-09 | Yes | Up | - |
| Q8N4W6 | DNAJC22 | 58/114(50.88) | 0.92 | 7.53E-06 | 3.11E-05 | Yes | Up | - |
| Q6Q0C0 | TRAF7 | 60/114(52.63) | 0.47 | 1.74E-09 | 1.92E-08 | Yes | Up | - |
| Q5K130 | CLLU1OS | 50/114(43.86) | 0.7 | 8.68E-03 | 1.71E-02 | Yes | Up | - |
| P49768 | PSEN1 | 65/114(57.02) | 0.16 | 4.45E-04 | 1.19E-03 | Yes | Up | - |
| Q9UKY0 | PRND | 67/114(58.77) | 0.62 | 1.80E-04 | 5.32E-04 | Yes | Up | - |
| O00463 | TRAF5 | 62/114(54.39) | 0.47 | 4.86E-06 | 2.09E-05 | Yes | Up | - |
| Q16769 | QPCT | 59/114(51.75) | 0.32 | 3.11E-03 | 6.80E-03 | Yes | Up | - |
| O15117 | FYB | 59/114(51.75) | 0.27 | 8.86E-03 | 1.74E-02 | Yes | Up | - |
| Q9H2X0 | CHRD | 68/114(59.65) | 0.56 | 1.58E-04 | 4.74E-04 | Yes | Up | - |
| P41240 | CSK | 61/114(53.51) | 0.39 | 1.28E-09 | 1.48E-08 | Yes | Up | - |
| - | ZNF815 | 55/114(48.25) | 0.25 | 1.28E-03 | 3.09E-03 | Yes | Up | - |
| Q8TE56 | ADAMTS17 | 65/114(57.02) | 0.26 | 2.81E-02 | 4.86E-02 | Yes | Up | - |
| Q96RP7 | GAL3ST4 | 64/114(56.14) | 0.36 | 3.84E-05 | 1.34E-04 | Yes | Up | - |
| Q8NI99 | ANGPTL6 | 68/114(59.65) | 0.77 | 2.04E-05 | 7.59E-05 | Yes | Up | - |
| Q6IPT2 | FAM71E1 | 64/114(56.14) | 0.59 | 1.36E-05 | 5.29E-05 | Yes | Up | - |
| Q99618 | CDCA3 | 60/114(52.63) | 1.56 | 1.37E-16 | 2.25E-14 | Yes | Up | - |
| Q9Y566 | SHANK1 | 58/114(50.88) | 0.46 | 3.37E-03 | 7.29E-03 | Yes | Up | - |
| Q9Y2S6 | CCDC72 | 62/114(54.39) | 0.17 | 8.67E-03 | 1.71E-02 | Yes | Up | - |
| P61326 | MAGOH | 63/114(55.26) | 0.21 | 9.70E-05 | 3.06E-04 | Yes | Up | - |
| Q96GM1 | LPPR2 | 67/114(58.77) | 0.45 | 1.98E-07 | 1.23E-06 | Yes | Up | - |
| Q9NX24 | NHP2 | 65/114(57.02) | 0.35 | 2.68E-06 | 1.23E-05 | Yes | Up | - |
| P45844 | ABCG1 | 61/114(53.51) | 0.42 | 7.41E-06 | 3.07E-05 | Yes | Up | - |
| Q6UW60 | PCSK4 | 61/114(53.51) | 0.76 | 5.76E-07 | 3.16E-06 | Yes | Up | - |
| P00491 | PNP | 61/114(53.51) | 0.49 | 1.71E-06 | 8.26E-06 | Yes | Up | - |
| Q5FYB1 | ARSI | 67/114(58.77) | 0.88 | 1.12E-08 | 9.81E-08 | Yes | Up | - |
| Q6IS14 | EIF5AL1 | 64/114(56.14) | 0.2 | 1.75E-03 | 4.08E-03 | Yes | Up | - |
| Q15029 | EFTUD2 | 55/114(48.25) | 0.18 | 8.82E-04 | 2.21E-03 | Yes | Up | - |
| Q9BT23 | LIMD2 | 67/114(58.77) | 0.57 | 3.26E-07 | 1.90E-06 | Yes | Up | - |
| Q96DA0 | ZG16B | 63/114(55.26) | 1.11 | 1.97E-07 | 1.22E-06 | Yes | Up | - |
| Q9BXV9 | C14ORF142 | 58/114(50.88) | 0.32 | 1.13E-06 | 5.72E-06 | Yes | Up | - |
| B4DYM4 | C1ORF9 | 54/114(47.37) | 0.56 | 2.88E-08 | 2.25E-07 | Yes | Up | - |
| P78317 | RNF4 | 57/114(50.0) | 0.15 | 9.77E-04 | 2.42E-03 | Yes | Up | - |
| Q86UR1 | NOXA1 | 68/114(59.65) | 0.47 | 2.98E-04 | 8.35E-04 | Yes | Up | - |
| P61158 | ACTR3 | 58/114(50.88) | 0.27 | 2.23E-04 | 6.45E-04 | Yes | Up | - |
| P25063 | CD24 | 57/114(50.0) | 0.61 | 3.07E-03 | 6.72E-03 | Yes | Up | - |
| P13284 | IFI30 | 64/114(56.14) | 1.07 | 4.87E-15 | 4.16E-13 | Yes | Up | - |
| Q8N5V2 | NGEF | 58/114(50.88) | 0.34 | 1.80E-02 | 3.28E-02 | Yes | Up | - |
| A6NI56 | CCDC154 | 55/114(48.25) | 0.47 | 1.18E-02 | 2.25E-02 | Yes | Up | - |
| Q8IZ40 | RCOR2 | 57/114(50.0) | 0.55 | 4.27E-04 | 1.15E-03 | Yes | Up | - |
| Q5BJH7 | YIF1B | 62/114(54.39) | 0.59 | 5.46E-11 | 9.74E-10 | Yes | Up | - |
| Q96KP6 | TNIP3 | 59/114(51.75) | 0.88 | 3.15E-06 | 1.42E-05 | Yes | Up | - |
| P54577 | YARS | 61/114(53.51) | 0.31 | 9.17E-06 | 3.72E-05 | Yes | Up | - |
| O43663 | PRC1 | 59/114(51.75) | 1.29 | 5.72E-14 | 3.05E-12 | Yes | Up | - |
| Q9NP78 | ABCB9 | 68/114(59.65) | 0.55 | 4.73E-07 | 2.65E-06 | Yes | Up | - |
| Q6P589 | TNFAIP8L2 | 67/114(58.77) | 0.27 | 1.34E-02 | 2.52E-02 | Yes | Up | - |
| O94979 | SEC31A | 57/114(50.0) | 0.16 | 5.34E-03 | 1.11E-02 | Yes | Up | - |
| Q12792 | TWF1 | 54/114(47.37) | 0.22 | 1.59E-03 | 3.75E-03 | Yes | Up | - |
| - | LOC285954 | 59/114(51.75) | 0.59 | 3.70E-04 | 1.01E-03 | Yes | Up | - |
| B7ZAQ6 | GPR89A | 59/114(51.75) | 0.48 | 3.29E-09 | 3.36E-08 | Yes | Up | - |
| Q9BRG1 | VPS25 | 66/114(57.89) | 0.35 | 6.41E-09 | 6.01E-08 | Yes | Up | - |
| P40121 | CAPG | 66/114(57.89) | 0.71 | 5.03E-13 | 1.82E-11 | Yes | Up | - |
| Q2WGJ9 | FER1L6 | 51/114(44.74) | 0.85 | 3.20E-04 | 8.89E-04 | Yes | Up | - |
| Q9UKA8 | RCAN3 | 54/114(47.37) | 0.27 | 6.20E-03 | 1.27E-02 | Yes | Up | - |
| P01011 | SERPINA3 | 58/114(50.88) | 0.75 | 3.57E-05 | 1.25E-04 | Yes | Up | - |
| Q8WWX9 | SELM | 69/114(60.53) | 0.26 | 1.17E-02 | 2.24E-02 | Yes | Up | - |
| P38646 | HSPA9 | 54/114(47.37) | 0.2 | 1.94E-03 | 4.49E-03 | Yes | Up | - |
| Q9H6Z9 | EGLN3 | 62/114(54.39) | 0.77 | 1.10E-06 | 5.57E-06 | Yes | Up | - |
| O15353 | FOXN1 | 62/114(54.39) | 0.47 | 1.93E-02 | 3.48E-02 | Yes | Up | - |
| A1A5B4 | ANO9 | 59/114(51.75) | 0.72 | 6.06E-06 | 2.55E-05 | Yes | Up | - |
| Q6UXU6 | TMEM92 | 60/114(52.63) | 1.15 | 2.71E-08 | 2.13E-07 | Yes | Up | - |
| Q13470 | TNK1 | 60/114(52.63) | 0.33 | 3.31E-03 | 7.18E-03 | Yes | Up | - |
| P16401 | HIST1H1B | 50/114(43.86) | 0.98 | 1.19E-04 | 3.68E-04 | Yes | Up | - |
| P09234 | SNRPC | 68/114(59.65) | 0.32 | 2.91E-06 | 1.33E-05 | Yes | Up | - |
| P21796 | VDAC1 | 59/114(51.75) | 0.25 | 8.36E-04 | 2.11E-03 | Yes | Up | - |
| Q14CZ7 | FASTKD3 | 54/114(47.37) | 0.21 | 2.53E-03 | 5.66E-03 | Yes | Up | - |
| O60565 | GREM1 | 62/114(54.39) | 0.91 | 4.75E-08 | 3.48E-07 | Yes | Up | - |
| Q8WUM9 | SLC20A1 | 57/114(50.0) | 0.61 | 2.50E-09 | 2.62E-08 | Yes | Up | - |
| Q9Y519 | TMEM184B | 57/114(50.0) | 0.15 | 1.37E-02 | 2.57E-02 | Yes | Up | - |
| Q9BQY9 | DBNDD2 | 62/114(54.39) | 0.26 | 6.66E-03 | 1.35E-02 | Yes | Up | - |
| Q9UGQ3 | SLC2A6 | 57/114(50.0) | 0.63 | 1.94E-05 | 7.28E-05 | Yes | Up | - |
| P58418 | CLRN1 | 31/114(27.19) | 1 | 9.80E-03 | 1.90E-02 | Yes | Up | - |
| P35908 | KRT2 | 46/114(40.35) | 0.79 | 1.05E-02 | 2.03E-02 | Yes | Up | - |
| Q96BI1 | SLC22A18 | 64/114(56.14) | 0.55 | 1.86E-06 | 8.88E-06 | Yes | Up | - |
| O14545 | TRAFD1 | 61/114(53.51) | 0.4 | 2.41E-10 | 3.45E-09 | Yes | Up | - |
| Q96S82 | UBL7 | 57/114(50.0) | 0.13 | 1.24E-02 | 2.36E-02 | Yes | Up | - |
| P53618 | COPB1 | 51/114(44.74) | 0.23 | 3.96E-04 | 1.08E-03 | Yes | Up | - |
| O00754 | MAN2B1 | 62/114(54.39) | 0.35 | 4.05E-06 | 1.77E-05 | Yes | Up | - |
| Q86V88 | MDP1 | 59/114(51.75) | 0.13 | 2.69E-02 | 4.68E-02 | Yes | Up | - |
| Q01668 | CACNA1D | 59/114(51.75) | 0.7 | 3.84E-04 | 1.05E-03 | Yes | Up | - |
| P12830 | CDH1 | 57/114(50.0) | 0.51 | 8.78E-03 | 1.73E-02 | Yes | Up | - |
| Q9UBH6 | XPR1 | 58/114(50.88) | 0.31 | 1.87E-05 | 7.03E-05 | Yes | Up | - |
| Q7L5D6 | GET4 | 56/114(49.12) | 0.24 | 3.93E-04 | 1.07E-03 | Yes | Up | - |
| Q6PHW0 | IYD | 57/114(50.0) | 0.81 | 1.05E-03 | 2.59E-03 | Yes | Up | - |
| O15229 | KMO | 56/114(49.12) | 0.76 | 9.62E-05 | 3.04E-04 | Yes | Up | - |
| B1AK53 | ESPN | 63/114(55.26) | 1.25 | 1.21E-11 | 2.69E-10 | Yes | Up | - |
| O60885 | BRD4 | 59/114(51.75) | 0.2 | 2.87E-05 | 1.03E-04 | Yes | Up | - |
| Q4G163 | FBXO43 | 62/114(54.39) | 1.12 | 1.08E-11 | 2.46E-10 | Yes | Up | - |
| Q9NPF4 | OSGEP | 62/114(54.39) | 0.14 | 3.80E-03 | 8.14E-03 | Yes | Up | - |
| Q96DY2 | IQCD | 63/114(55.26) | 0.74 | 4.73E-10 | 6.26E-09 | Yes | Up | - |
| P43007 | SLC1A4 | 57/114(50.0) | 0.61 | 1.08E-07 | 7.18E-07 | Yes | Up | - |
| Q92522 | H1FX | 70/114(61.4) | 0.28 | 2.81E-05 | 1.01E-04 | Yes | Up | - |
| Q86Y56 | HEATR2 | 58/114(50.88) | 0.35 | 1.39E-06 | 6.86E-06 | Yes | Up | - |
| Q96M91 | CCDC11 | 59/114(51.75) | 0.61 | 2.15E-05 | 7.95E-05 | Yes | Up | - |
| Q9BYT1 | SLC17A9 | 63/114(55.26) | 0.87 | 6.87E-10 | 8.61E-09 | Yes | Up | - |
| Q14088 | RAB33A | 61/114(53.51) | 0.48 | 7.09E-05 | 2.31E-04 | Yes | Up | - |
| Q6UVJ0 | SASS6 | 56/114(49.12) | 0.32 | 4.70E-04 | 1.26E-03 | Yes | Up | - |
| Q9NQR4 | NIT2 | 53/114(46.49) | 0.21 | 3.70E-04 | 1.01E-03 | Yes | Up | - |
| Q96S21 | RAB40C | 64/114(56.14) | 0.53 | 1.76E-11 | 3.71E-10 | Yes | Up | - |
| Q96SI1 | KCTD15 | 64/114(56.14) | 0.24 | 1.10E-02 | 2.10E-02 | Yes | Up | - |
| Q01974 | ROR2 | 57/114(50.0) | 0.49 | 2.89E-04 | 8.11E-04 | Yes | Up | - |
| Q9UJQ4 | SALL4 | 57/114(50.0) | 1.67 | 5.90E-18 | 1.60E-15 | Yes | Up | - |
| P48643 | CCT5 | 60/114(52.63) | 0.3 | 6.52E-04 | 1.69E-03 | Yes | Up | - |
| Q5VTE6 | ANGEL2 | 56/114(49.12) | 0.18 | 2.55E-03 | 5.70E-03 | Yes | Up | - |
| Q9BV44 | THUMPD3 | 57/114(50.0) | 0.21 | 2.19E-04 | 6.35E-04 | Yes | Up | - |
| Q9H310 | RHBG | 58/114(50.88) | 1.49 | 1.11E-12 | 3.53E-11 | Yes | Up | - |
| P31930 | UQCRC1 | 56/114(49.12) | 0.18 | 7.34E-03 | 1.47E-02 | Yes | Up | - |
| Q9C0C4 | SEMA4C | 63/114(55.26) | 0.19 | 8.52E-03 | 1.68E-02 | Yes | Up | - |
| Q9P1W9 | PIM2 | 61/114(53.51) | 0.45 | 2.41E-06 | 1.12E-05 | Yes | Up | - |
| Q8NB14 | USP38 | 56/114(49.12) | 0.15 | 2.44E-02 | 4.29E-02 | Yes | Up | - |
| A2A3N6 | PIPSL | 61/114(53.51) | 0.31 | 5.81E-06 | 2.46E-05 | Yes | Up | - |
| Q9UHY7 | ENOPH1 | 52/114(45.61) | 0.32 | 8.02E-06 | 3.29E-05 | Yes | Up | - |
| Q8N806 | UBR7 | 54/114(47.37) | 0.18 | 3.31E-03 | 7.18E-03 | Yes | Up | - |
| Q17RN3 | FAM98C | 66/114(57.89) | 0.29 | 2.06E-04 | 5.99E-04 | Yes | Up | - |
| Q7L190 | DPPA4 | 38/114(33.33) | 0.63 | 2.11E-02 | 3.77E-02 | Yes | Up | - |
| Q06643 | LTB | 67/114(58.77) | 0.71 | 1.40E-04 | 4.25E-04 | Yes | Up | - |
| Q96H20 | SNF8 | 65/114(57.02) | 0.28 | 4.34E-04 | 1.17E-03 | Yes | Up | - |
| A4FU49 | C1ORF113 | 62/114(54.39) | 0.46 | 4.67E-04 | 1.25E-03 | Yes | Up | - |
| Q9Y333 | LSM2 | 59/114(51.75) | 0.2 | 9.52E-04 | 2.36E-03 | Yes | Up | - |
| O15127 | SCAMP2 | 58/114(50.88) | 0.15 | 1.58E-03 | 3.72E-03 | Yes | Up | - |
| Q93077 | HIST1H2AC | 59/114(51.75) | 0.8 | 1.05E-09 | 1.24E-08 | Yes | Up | - |
| Q99487 | PAFAH2 | 58/114(50.88) | 0.25 | 3.30E-05 | 1.17E-04 | Yes | Up | - |
| - | SNORD1C | 55/114(48.25) | 0.28 | 6.44E-03 | 1.31E-02 | Yes | Up | - |
| Q9BX63 | BRIP1 | 59/114(51.75) | 1.15 | 5.44E-11 | 9.72E-10 | Yes | Up | - |
| P49006 | MARCKSL1 | 62/114(54.39) | 0.71 | 3.65E-08 | 2.76E-07 | Yes | Up | - |
| Q9NWS0 | PIH1D1 | 63/114(55.26) | 0.2 | 4.90E-04 | 1.30E-03 | Yes | Up | - |
| P33240 | CSTF2 | 57/114(50.0) | 0.43 | 9.56E-08 | 6.45E-07 | Yes | Up | - |
| Q9BSE2 | TMEM79 | 63/114(55.26) | 0.73 | 2.94E-09 | 3.05E-08 | Yes | Up | - |
| Q9H4Z2 | ZNF335 | 63/114(55.26) | 0.12 | 1.34E-02 | 2.52E-02 | Yes | Up | - |
| P78345 | RPP38 | 61/114(53.51) | 0.17 | 1.36E-03 | 3.25E-03 | Yes | Up | - |
| P61026 | RAB10 | 53/114(46.49) | 0.17 | 7.38E-03 | 1.48E-02 | Yes | Up | - |
| Q9NRX2 | MRPL17 | 63/114(55.26) | 0.42 | 6.20E-08 | 4.40E-07 | Yes | Up | - |
| Q9BT17 | MTG1 | 67/114(58.77) | 0.17 | 6.39E-04 | 1.66E-03 | Yes | Up | - |
| P43489 | TNFRSF4 | 67/114(58.77) | 0.6 | 2.74E-05 | 9.84E-05 | Yes | Up | - |
| Q9NWT8 | AURKAIP1 | 64/114(56.14) | 0.43 | 1.06E-05 | 4.23E-05 | Yes | Up | - |
| Q86U17 | SERPINA11 | 55/114(48.25) | 1 | 2.44E-04 | 6.97E-04 | Yes | Up | - |
| - | HPYR1 | 35/114(30.7) | 0.98 | 1.03E-02 | 1.99E-02 | Yes | Up | - |
| Q9BYN0 | SRXN1 | 55/114(48.25) | 0.48 | 1.63E-07 | 1.03E-06 | Yes | Up | - |
| - | LOC400696 | 58/114(50.88) | 1 | 1.78E-04 | 5.26E-04 | Yes | Up | - |
| Q9H2V7 | SPNS1 | 63/114(55.26) | 0.36 | 7.46E-08 | 5.19E-07 | Yes | Up | - |
| O60303 | KIAA0556 | 59/114(51.75) | 0.29 | 3.45E-04 | 9.52E-04 | Yes | Up | - |
| Q16795 | NDUFA9 | 59/114(51.75) | 0.15 | 1.62E-02 | 2.98E-02 | Yes | Up | - |
| P55056 | APOC4 | 49/114(42.98) | 0.84 | 2.73E-04 | 7.71E-04 | Yes | Up | - |
| A8K8P3 | SFI1 | 58/114(50.88) | 0.38 | 2.75E-05 | 9.88E-05 | Yes | Up | - |
| Q13568 | IRF5 | 61/114(53.51) | 0.41 | 4.58E-06 | 1.99E-05 | Yes | Up | - |
| Q14116 | IL18 | 59/114(51.75) | 0.5 | 4.49E-05 | 1.53E-04 | Yes | Up | - |
| P0C6T2 | OST4 | 61/114(53.51) | 0.23 | 8.68E-04 | 2.18E-03 | Yes | Up | - |
| Q96FH0 | LOC729991 | 65/114(57.02) | 0.23 | 2.49E-04 | 7.11E-04 | Yes | Up | - |
| Q495T6 | MMEL1 | 62/114(54.39) | 1.09 | 3.59E-11 | 6.86E-10 | Yes | Up | - |
| Q86VI4 | LAPTM4B | 64/114(56.14) | 0.32 | 8.10E-03 | 1.61E-02 | Yes | Up | - |
| Q9BRP7 | FDXACB1 | 62/114(54.39) | 0.17 | 1.62E-02 | 2.98E-02 | Yes | Up | - |
| Q13573 | SNW1 | 63/114(55.26) | 0.08 | 2.68E-02 | 4.67E-02 | Yes | Up | - |
| Q17RW2 | COL24A1 | 54/114(47.37) | 0.89 | 5.71E-09 | 5.44E-08 | Yes | Up | - |
| Q16850 | CYP51A1 | 58/114(50.88) | 0.29 | 5.99E-04 | 1.56E-03 | Yes | Up | - |
| P04035 | HMGCR | 53/114(46.49) | 0.24 | 8.87E-03 | 1.74E-02 | Yes | Up | - |
| O95239 | KIF4A | 58/114(50.88) | 1.83 | 2.51E-14 | 1.59E-12 | Yes | Up | - |
| P62879 | GNB2 | 68/114(59.65) | 0.32 | 1.00E-06 | 5.14E-06 | Yes | Up | - |
| Q14197 | ICT1 | 59/114(51.75) | 0.38 | 1.85E-06 | 8.86E-06 | Yes | Up | - |
| Q14151 | SAFB2 | 70/114(61.4) | 0.18 | 5.17E-04 | 1.37E-03 | Yes | Up | - |
| Q5T1V6 | DDX59 | 54/114(47.37) | 0.12 | 2.21E-02 | 3.92E-02 | Yes | Up | - |
| Q99571 | P2RX4 | 59/114(51.75) | 0.54 | 7.69E-12 | 1.84E-10 | Yes | Up | - |
| O75348 | ATP6V1G1 | 58/114(50.88) | 0.42 | 2.04E-08 | 1.68E-07 | Yes | Up | - |
| Q86UN3 | RTN4RL2 | 58/114(50.88) | 0.8 | 2.28E-10 | 3.29E-09 | Yes | Up | - |
| Q96A49 | SYAP1 | 53/114(46.49) | 0.5 | 1.28E-07 | 8.31E-07 | Yes | Up | - |
| Q9UJK0 | C16ORF42 | 65/114(57.02) | 0.31 | 3.18E-05 | 1.13E-04 | Yes | Up | - |
| Q9BUL8 | PDCD10 | 61/114(53.51) | 0.3 | 3.48E-06 | 1.55E-05 | Yes | Up | - |
| Q9H720 | CWH43 | 63/114(55.26) | 1.12 | 2.64E-05 | 9.54E-05 | Yes | Up | - |
| S4R338 | METTL11A | 63/114(55.26) | 0.39 | 4.64E-07 | 2.61E-06 | Yes | Up | - |
| Q9H1V8 | SLC6A17 | 58/114(50.88) | 0.86 | 4.89E-07 | 2.73E-06 | Yes | Up | - |
| Q8NFW1 | COL22A1 | 65/114(57.02) | 1.48 | 1.01E-14 | 7.34E-13 | Yes | Up | - |
| Q96SL8 | FIZ1 | 68/114(59.65) | 0.21 | 2.35E-04 | 6.75E-04 | Yes | Up | - |
| O95988 | TCL1B | 40/114(35.09) | 1.35 | 9.62E-05 | 3.04E-04 | Yes | Up | - |
| Q96LB8 | PGLYRP4 | 44/114(38.6) | 0.72 | 1.64E-02 | 3.02E-02 | Yes | Up | - |
| Q9Y2R5 | MRPS17 | 65/114(57.02) | 0.26 | 3.10E-04 | 8.66E-04 | Yes | Up | - |
| Q14802 | FXYD3 | 51/114(44.74) | 0.81 | 9.45E-06 | 3.82E-05 | Yes | Up | - |
| A6H8M9 | CDHR4 | 54/114(47.37) | 1.11 | 1.23E-04 | 3.78E-04 | Yes | Up | - |
| P58173 | OR2B6 | 50/114(43.86) | 1.71 | 2.22E-08 | 1.80E-07 | Yes | Up | - |
| O95336 | PGLS | 68/114(59.65) | 0.35 | 7.73E-07 | 4.10E-06 | Yes | Up | - |
| Q13368 | MPP3 | 59/114(51.75) | 0.23 | 2.22E-02 | 3.95E-02 | Yes | Up | - |
| Q86X55 | CARM1 | 62/114(54.39) | 0.37 | 1.07E-07 | 7.11E-07 | Yes | Up | - |
| Q9Y244 | POMP | 53/114(46.49) | 0.2 | 8.64E-03 | 1.70E-02 | Yes | Up | - |
| - | TPI1P2 | 61/114(53.51) | 0.3 | 4.37E-03 | 9.24E-03 | Yes | Up | - |
| O43543 | XRCC2 | 54/114(47.37) | 0.8 | 3.20E-07 | 1.87E-06 | Yes | Up | - |
| P14635 | CCNB1 | 59/114(51.75) | 1.22 | 7.57E-14 | 3.80E-12 | Yes | Up | - |
| P23527 | HIST1H2BO | 56/114(49.12) | 1.74 | 1.06E-09 | 1.26E-08 | Yes | Up | - |
| Q9H5X1 | FAM96A | 59/114(51.75) | 0.45 | 1.15E-08 | 1.00E-07 | Yes | Up | - |
| Q9NR82 | KCNQ5 | 60/114(52.63) | 0.64 | 4.05E-04 | 1.10E-03 | Yes | Up | - |
| - | LOC728554 | 62/114(54.39) | 0.26 | 2.01E-02 | 3.61E-02 | Yes | Up | - |
| O60883 | GPR37L1 | 61/114(53.51) | 1.15 | 2.90E-11 | 5.75E-10 | Yes | Up | - |
| Q15646 | OASL | 60/114(52.63) | 1.16 | 1.06E-13 | 5.12E-12 | Yes | Up | - |
| P16402 | HIST1H1D | 58/114(50.88) | 1.34 | 6.19E-09 | 5.82E-08 | Yes | Up | - |
| Q3B8N2 | LGALS9B | 48/114(42.11) | 2.01 | 2.12E-09 | 2.28E-08 | Yes | Up | - |
| Q9UKR5 | C14ORF1 | 58/114(50.88) | 0.26 | 4.28E-05 | 1.47E-04 | Yes | Up | - |
| P13674 | P4HA1 | 60/114(52.63) | 0.41 | 5.49E-05 | 1.84E-04 | Yes | Up | - |
| Q04726 | TLE3 | 58/114(50.88) | 0.54 | 4.18E-10 | 5.61E-09 | Yes | Up | - |
| A4D2B0 | MBLAC1 | 67/114(58.77) | 0.24 | 6.89E-03 | 1.39E-02 | Yes | Up | - |
| Q96TA2 | YME1L1 | 54/114(47.37) | 0.13 | 2.05E-02 | 3.67E-02 | Yes | Up | - |
| Q9NRA0 | SPHK2 | 68/114(59.65) | 0.18 | 7.75E-03 | 1.55E-02 | Yes | Up | - |
| Q86XT9 | TMEM219 | 67/114(58.77) | 0.25 | 1.51E-05 | 5.80E-05 | Yes | Up | - |
| Q86UX7 | FERMT3 | 60/114(52.63) | 0.35 | 1.83E-03 | 4.26E-03 | Yes | Up | - |
| Q96JT2 | SLC45A3 | 58/114(50.88) | 0.28 | 2.66E-03 | 5.92E-03 | Yes | Up | - |
| Q9P0T7 | TMEM9 | 67/114(58.77) | 0.56 | 1.02E-10 | 1.67E-09 | Yes | Up | - |
| Q15726 | KISS1 | 47/114(41.23) | 1.19 | 2.94E-07 | 1.74E-06 | Yes | Up | - |
| Q92949 | FOXJ1 | 53/114(46.49) | 1.9 | 4.09E-15 | 3.62E-13 | Yes | Up | - |
| Q9BUA3 | C11ORF84 | 60/114(52.63) | 0.37 | 2.23E-06 | 1.05E-05 | Yes | Up | - |
| P41231 | P2RY2 | 60/114(52.63) | 0.58 | 4.19E-05 | 1.45E-04 | Yes | Up | - |
| O14524 | TMEM194A | 66/114(57.89) | 0.26 | 2.70E-04 | 7.66E-04 | Yes | Up | - |
| Q96I82 | KAZALD1 | 61/114(53.51) | 0.42 | 2.94E-03 | 6.46E-03 | Yes | Up | - |
| Q12888 | TP53BP1 | 52/114(45.61) | 0.14 | 2.35E-02 | 4.15E-02 | Yes | Up | - |
| P37198 | NUP62 | 60/114(52.63) | 0.33 | 2.95E-08 | 2.29E-07 | Yes | Up | - |
| Q8N4S7 | PAQR4 | 59/114(51.75) | 1.42 | 7.75E-18 | 1.92E-15 | Yes | Up | - |
| P67775 | PPP2CA | 58/114(50.88) | 0.21 | 1.28E-04 | 3.92E-04 | Yes | Up | - |
| Q9UIC8 | LCMT1 | 68/114(59.65) | 0.34 | 3.64E-07 | 2.10E-06 | Yes | Up | - |
| Q96G23 | LASS2 | 59/114(51.75) | 0.76 | 1.38E-12 | 4.23E-11 | Yes | Up | - |
| O76082 | SLC22A5 | 53/114(46.49) | 0.27 | 3.03E-03 | 6.64E-03 | Yes | Up | - |
| Q8N3Y7 | SDR16C5 | 58/114(50.88) | 1.05 | 3.13E-06 | 1.42E-05 | Yes | Up | - |
| Q86XQ3 | CATSPER3 | 62/114(54.39) | 0.37 | 5.69E-04 | 1.49E-03 | Yes | Up | - |
| Q96GD0 | PDXP | 60/114(52.63) | 0.3 | 3.70E-05 | 1.29E-04 | Yes | Up | - |
| Q96L03 | SPATA17 | 62/114(54.39) | 1.16 | 6.06E-12 | 1.51E-10 | Yes | Up | - |
| - | NCRNA00202 | 57/114(50.0) | 0.43 | 1.12E-02 | 2.14E-02 | Yes | Up | - |
| F8W6F3 | PRR5-ARHGAP8 | 64/114(56.14) | 0.55 | 1.25E-03 | 3.03E-03 | Yes | Up | - |
| P60709 | ACTB | 64/114(56.14) | 0.27 | 8.70E-05 | 2.77E-04 | Yes | Up | - |
| P25067 | COL8A2 | 60/114(52.63) | 0.54 | 1.56E-05 | 5.97E-05 | Yes | Up | - |
| P58512 | C21ORF67 | 55/114(48.25) | 0.27 | 5.26E-04 | 1.39E-03 | Yes | Up | - |
| P56178 | DLX5 | 59/114(51.75) | 0.91 | 3.39E-08 | 2.60E-07 | Yes | Up | - |
| Q49A17 | GALNTL6 | 52/114(45.61) | 0.58 | 1.97E-03 | 4.54E-03 | Yes | Up | - |
| Q6QHC5 | DEGS2 | 55/114(48.25) | 1.29 | 1.97E-09 | 2.14E-08 | Yes | Up | - |
| Q08J23 | NSUN2 | 60/114(52.63) | 0.2 | 5.68E-03 | 1.17E-02 | Yes | Up | - |
| Q6P4F7 | ARHGAP11A | 54/114(47.37) | 0.97 | 3.96E-09 | 3.94E-08 | Yes | Up | - |
| O14964 | HGS | 62/114(54.39) | 0.27 | 6.05E-06 | 2.55E-05 | Yes | Up | - |
| Q14974 | KPNB1 | 53/114(46.49) | 0.17 | 5.84E-03 | 1.20E-02 | Yes | Up | - |
| P14324 | FDPS | 59/114(51.75) | 0.31 | 1.53E-04 | 4.61E-04 | Yes | Up | - |
| Q9UNF1 | MAGED2 | 54/114(47.37) | 0.66 | 6.08E-08 | 4.32E-07 | Yes | Up | - |
| Q6ZNE9 | RUFY4 | 59/114(51.75) | 1.09 | 2.61E-08 | 2.06E-07 | Yes | Up | - |
| Q9UKX5 | ITGA11 | 64/114(56.14) | 0.64 | 1.60E-06 | 7.75E-06 | Yes | Up | - |
| Q9UDV7 | ZNF282 | 58/114(50.88) | 0.14 | 1.50E-02 | 2.79E-02 | Yes | Up | - |
| Q01892 | SPIB | 60/114(52.63) | 0.8 | 4.94E-05 | 1.67E-04 | Yes | Up | - |
| P18065 | IGFBP2 | 65/114(57.02) | 0.36 | 1.47E-02 | 2.73E-02 | Yes | Up | - |
| Q9H0A0 | NAT10 | 52/114(45.61) | 0.26 | 2.20E-04 | 6.36E-04 | Yes | Up | - |
| P31431 | SDC4 | 57/114(50.0) | 0.26 | 2.69E-02 | 4.67E-02 | Yes | Up | - |
| P48637 | GSS | 63/114(55.26) | 0.25 | 1.53E-04 | 4.61E-04 | Yes | Up | - |
| P41218 | MNDA | 57/114(50.0) | 0.41 | 1.18E-04 | 3.64E-04 | Yes | Up | - |
| Q14181 | POLA2 | 66/114(57.89) | 0.42 | 3.44E-07 | 2.00E-06 | Yes | Up | - |
| Q7L5N1 | COPS6 | 67/114(58.77) | 0.19 | 5.74E-04 | 1.50E-03 | Yes | Up | - |
| Q9BSY4 | CHCHD5 | 71/114(62.28) | 0.39 | 4.33E-07 | 2.45E-06 | Yes | Up | - |
| Q9UBI1 | COMMD3 | 59/114(51.75) | 0.38 | 4.22E-07 | 2.39E-06 | Yes | Up | - |
| O95235 | KIF20A | 58/114(50.88) | 1.64 | 4.08E-13 | 1.53E-11 | Yes | Up | - |
| O75541 | ZNF821 | 63/114(55.26) | 0.16 | 1.77E-02 | 3.22E-02 | Yes | Up | - |
| O15321 | TM9SF1 | 57/114(50.0) | 0.3 | 5.28E-07 | 2.92E-06 | Yes | Up | - |
| Q9BYD5 | CNFN | 72/114(63.16) | 0.47 | 4.80E-05 | 1.63E-04 | Yes | Up | - |
| Q9ULC6 | PADI1 | 48/114(42.11) | 0.91 | 1.66E-04 | 4.96E-04 | Yes | Up | - |
| Q9H3P7 | ACBD3 | 53/114(46.49) | 0.41 | 4.20E-07 | 2.38E-06 | Yes | Up | - |
| O95202 | LETM1 | 60/114(52.63) | 0.23 | 1.02E-03 | 2.52E-03 | Yes | Up | - |
| P98182 | ZNF200 | 58/114(50.88) | 0.31 | 7.72E-09 | 7.09E-08 | Yes | Up | - |
| Q5T4D3 | TMTC4 | 54/114(47.37) | 0.2 | 1.73E-02 | 3.15E-02 | Yes | Up | - |
| Q06250 | WIT1 | 61/114(53.51) | 1.27 | 3.13E-04 | 8.73E-04 | Yes | Up | - |
| Q9BXL6 | CARD14 | 57/114(50.0) | 0.59 | 1.67E-04 | 4.99E-04 | Yes | Up | - |
| Q9UMS5 | PHTF1 | 57/114(50.0) | 0.38 | 1.13E-07 | 7.43E-07 | Yes | Up | - |
| Q96D15 | RCN3 | 62/114(54.39) | 0.42 | 8.90E-04 | 2.23E-03 | Yes | Up | - |
| Q13316 | DMP1 | 54/114(47.37) | 1.19 | 3.22E-05 | 1.14E-04 | Yes | Up | - |
| Q8TAP8 | C7ORF47 | 58/114(50.88) | 0.5 | 1.71E-09 | 1.89E-08 | Yes | Up | - |
| Q9H7L9 | SUDS3 | 49/114(42.98) | 0.23 | 2.66E-04 | 7.54E-04 | Yes | Up | - |
| Q9BU89 | DOHH | 71/114(62.28) | 0.39 | 1.07E-05 | 4.26E-05 | Yes | Up | - |
| P19367 | HK1 | 59/114(51.75) | 0.22 | 7.21E-04 | 1.85E-03 | Yes | Up | - |
| Q8TCT7 | SPPL2B | 67/114(58.77) | 0.16 | 8.34E-03 | 1.65E-02 | Yes | Up | - |
| Q86VX2 | COMMD7 | 60/114(52.63) | 0.28 | 2.87E-06 | 1.31E-05 | Yes | Up | - |
| A6NF83 | LOC389493 | 61/114(53.51) | 1.11 | 1.64E-07 | 1.04E-06 | Yes | Up | - |
| Q6PIU2 | NCEH1 | 58/114(50.88) | 0.41 | 1.48E-05 | 5.69E-05 | Yes | Up | - |
| Q9Y5E7 | PCDHB2 | 54/114(47.37) | 0.45 | 1.33E-03 | 3.19E-03 | Yes | Up | - |
| Q13625 | TP53BP2 | 60/114(52.63) | 0.3 | 9.73E-05 | 3.07E-04 | Yes | Up | - |
| Q9Y2D9 | ZNF652 | 53/114(46.49) | 0.21 | 1.58E-02 | 2.91E-02 | Yes | Up | - |
| O43927 | CXCL13 | 64/114(56.14) | 0.69 | 3.89E-03 | 8.31E-03 | Yes | Up | - |
| P20908 | COL5A1 | 59/114(51.75) | 1.22 | 4.69E-13 | 1.73E-11 | Yes | Up | - |
| Q8IYN0 | ZNF100 | 48/114(42.11) | 0.26 | 1.66E-03 | 3.90E-03 | Yes | Up | - |
| Q96IQ9 | ZNF414 | 63/114(55.26) | 0.19 | 4.98E-03 | 1.04E-02 | Yes | Up | - |
| Q16774 | GUK1 | 62/114(54.39) | 0.38 | 1.77E-05 | 6.70E-05 | Yes | Up | - |
| Q5T124 | UBXN11 | 68/114(59.65) | 0.24 | 5.33E-03 | 1.10E-02 | Yes | Up | - |
| Q9Y2D8 | SSX2IP | 57/114(50.0) | 0.73 | 5.30E-09 | 5.10E-08 | Yes | Up | - |
| O95989 | NUDT3 | 58/114(50.88) | 0.25 | 1.55E-04 | 4.67E-04 | Yes | Up | - |
| P56385 | ATP5I | 63/114(55.26) | 0.24 | 1.33E-03 | 3.20E-03 | Yes | Up | - |
| Q92583 | CCL17 | 61/114(53.51) | 0.71 | 1.15E-04 | 3.55E-04 | Yes | Up | - |
| P24588 | AKAP5 | 58/114(50.88) | 0.9 | 2.09E-09 | 2.25E-08 | Yes | Up | - |
| P35520 | CBS | 62/114(54.39) | 0.76 | 5.63E-06 | 2.39E-05 | Yes | Up | - |
| - | C8ORF39 | 58/114(50.88) | 0.3 | 1.34E-02 | 2.52E-02 | Yes | Up | - |
| Q9GZM5 | YIPF3 | 58/114(50.88) | 0.22 | 1.74E-04 | 5.17E-04 | Yes | Up | - |
| P63165 | SUMO1 | 64/114(56.14) | 0.18 | 2.19E-04 | 6.33E-04 | Yes | Up | - |
| P28062 | PSMB8 | 66/114(57.89) | 0.26 | 4.57E-03 | 9.64E-03 | Yes | Up | - |
| Q9Y2T4 | PPP2R2C | 59/114(51.75) | 1.82 | 1.00E-14 | 7.34E-13 | Yes | Up | - |
| Q96CJ1 | EAF2 | 56/114(49.12) | 0.27 | 6.99E-03 | 1.41E-02 | Yes | Up | - |
| Q96KS9 | FAM167A | 57/114(50.0) | 0.41 | 1.84E-02 | 3.34E-02 | Yes | Up | - |
| Q6ZU64 | CCDC108 | 67/114(58.77) | 0.97 | 1.80E-05 | 6.79E-05 | Yes | Up | - |
| Q9UBX3 | SLC25A10 | 57/114(50.0) | 0.41 | 2.13E-05 | 7.87E-05 | Yes | Up | - |
| P37802 | TAGLN2 | 65/114(57.02) | 0.53 | 4.52E-09 | 4.44E-08 | Yes | Up | - |
| P41002 | CCNF | 56/114(49.12) | 1 | 1.30E-13 | 5.97E-12 | Yes | Up | - |
| P42574 | CASP3 | 61/114(53.51) | 0.39 | 1.32E-07 | 8.57E-07 | Yes | Up | - |
| P61160 | ACTR2 | 59/114(51.75) | 0.22 | 1.65E-03 | 3.87E-03 | Yes | Up | - |
| Q96P50 | ACAP3 | 57/114(50.0) | 0.21 | 3.93E-03 | 8.40E-03 | Yes | Up | - |
| P00749 | PLAU | 60/114(52.63) | 0.71 | 6.31E-08 | 4.48E-07 | Yes | Up | - |
| Q6FI13 | HIST2H2AA3 | 67/114(58.77) | 1.65 | 2.18E-21 | 1.91E-18 | Yes | Up | - |
| Q9H0K6 | PUS7L | 56/114(49.12) | 0.24 | 2.74E-03 | 6.07E-03 | Yes | Up | - |
| Q9BQC3 | DPH2 | 63/114(55.26) | 0.19 | 3.27E-03 | 7.11E-03 | Yes | Up | - |
| Q6PK04 | CCDC137 | 64/114(56.14) | 0.5 | 7.99E-09 | 7.28E-08 | Yes | Up | - |
| Q14156 | EFR3A | 58/114(50.88) | 0.27 | 1.24E-03 | 3.00E-03 | Yes | Up | - |
| Q9UHI7 | SLC23A1 | 59/114(51.75) | 0.87 | 4.25E-06 | 1.85E-05 | Yes | Up | - |
| O15264 | MAPK13 | 56/114(49.12) | 0.64 | 2.61E-07 | 1.57E-06 | Yes | Up | - |
| O14965 | AURKA | 59/114(51.75) | 1.44 | 3.13E-14 | 1.90E-12 | Yes | Up | - |
| Q9BR11 | ZSWIM1 | 66/114(57.89) | 0.14 | 3.33E-03 | 7.23E-03 | Yes | Up | - |
| P31644 | GABRA5 | 50/114(43.86) | 0.95 | 6.92E-03 | 1.40E-02 | Yes | Up | - |
| O60671 | RAD1 | 53/114(46.49) | 0.19 | 3.52E-03 | 7.59E-03 | Yes | Up | - |
[truncated: 63,573 more chars]
